# Supplementary material for: Catalytic asymmetric radical aminoperfluoroalkylation and aminodifluoromethylation of alkenes to versatile enantioenriched-fluoroalkyl amines
Source: Nat Commun. 2017 Mar 23;8:14841. doi: 10.1038/ncomms14841 (PMC5376653; doi:10.1038/ncomms14841)
Supplement: Supplementary Information — Supplementary Figures, Supplementary Methods and Supplementary References. [file ncomms14841-s1.pdf]

Supplementary Figures

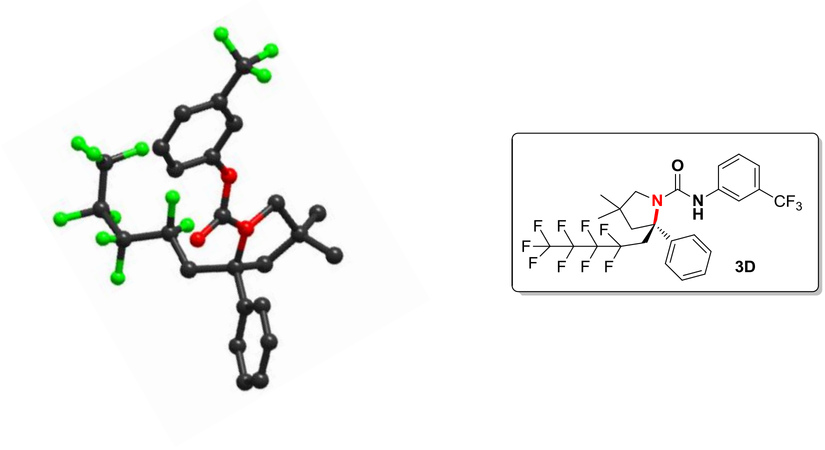

Supplementary Figure 1. X-ray of chiral compound **3D**

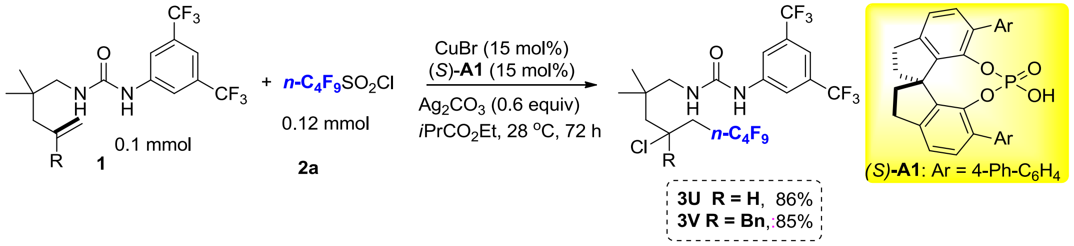

Supplementary Figure 2. substrates **1U** and **1V** is under the identical reaction conditions

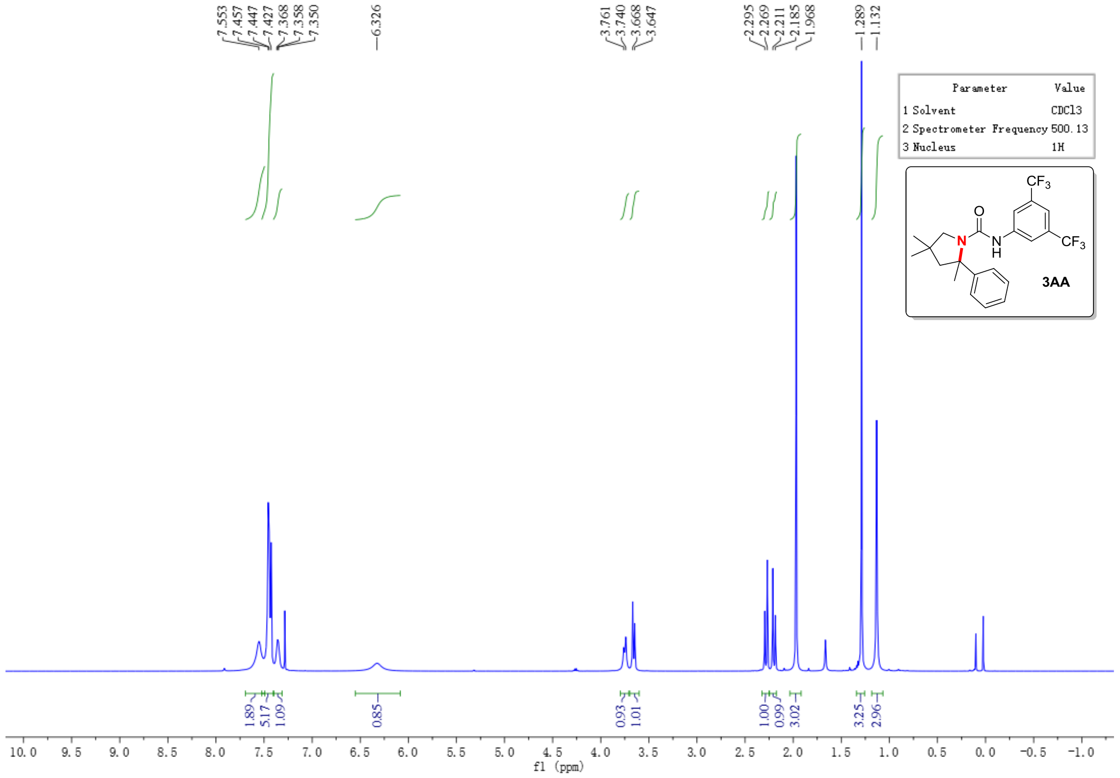

Supplementary Figure 3. ^{1}H NMR of **3AA**

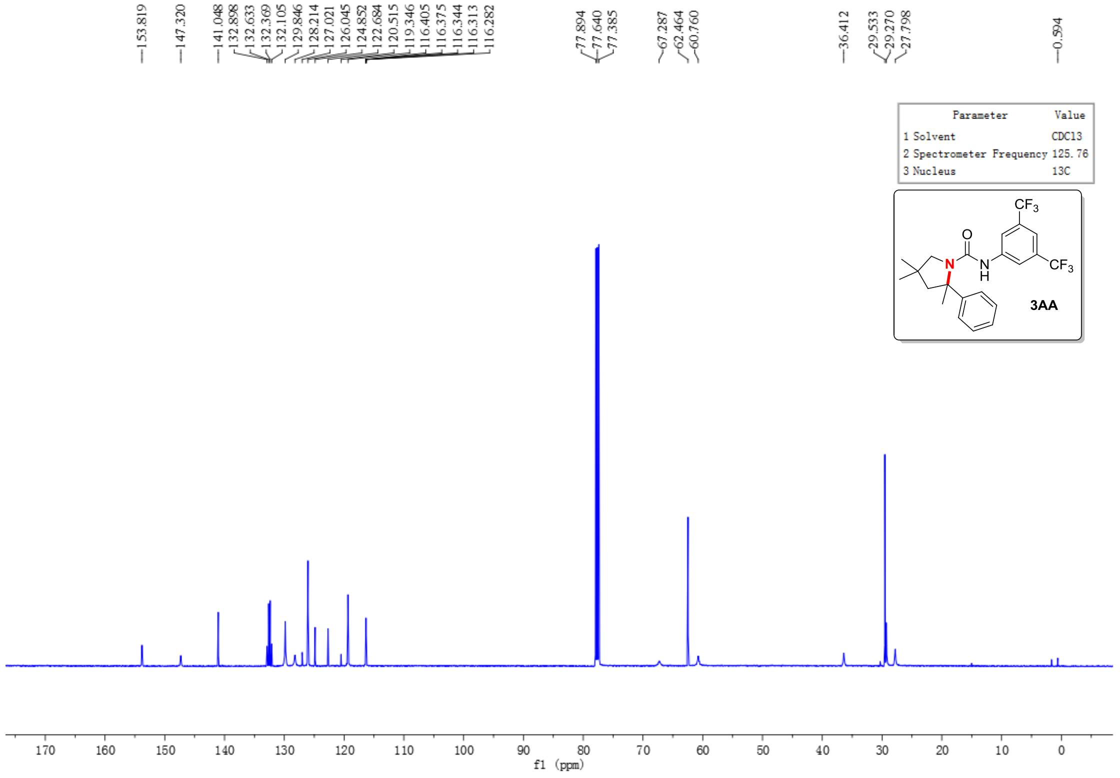

Supplementary Figure 4. ^{13}C NMR of **3AA**

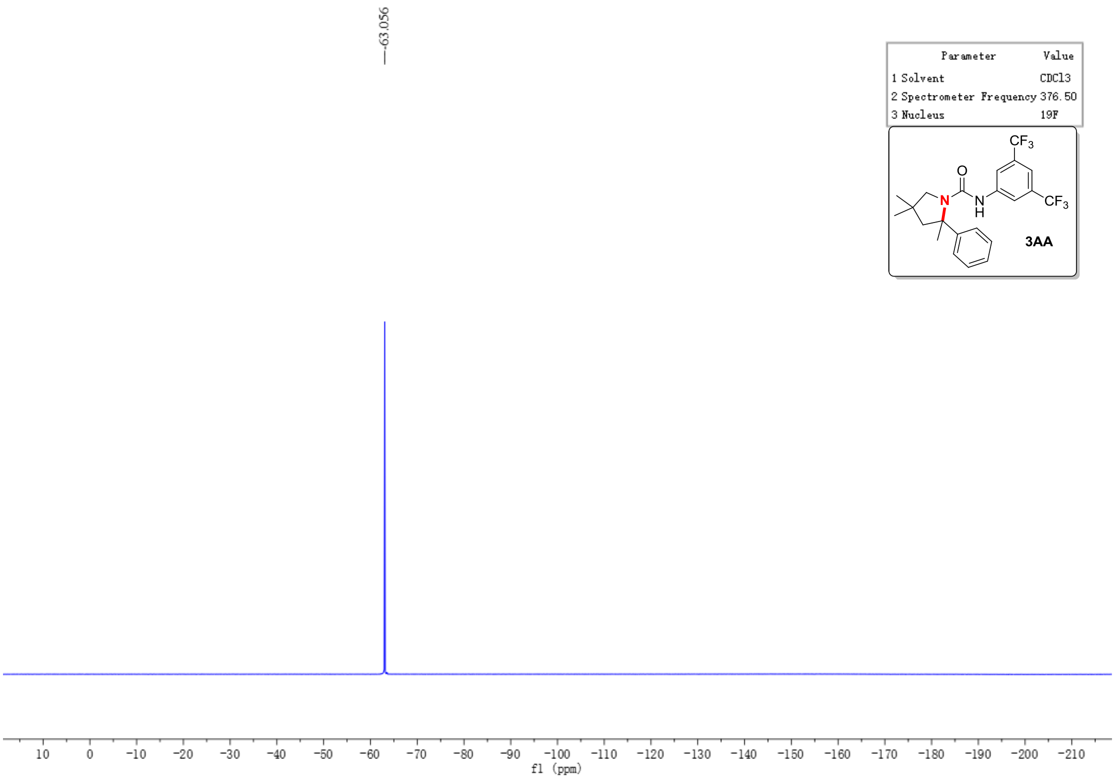

Supplementary Figure 5. ^{19}F NMR of **3AA**

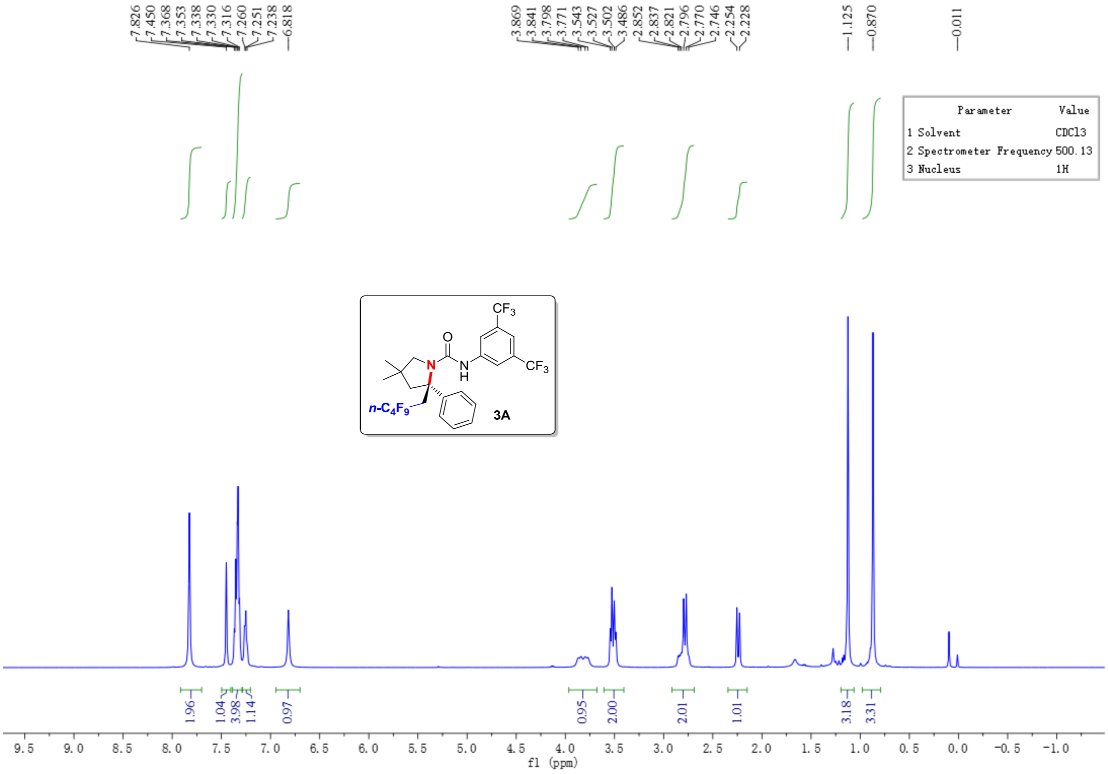

Supplementary Figure 6. ^{1}H NMR of **3A**

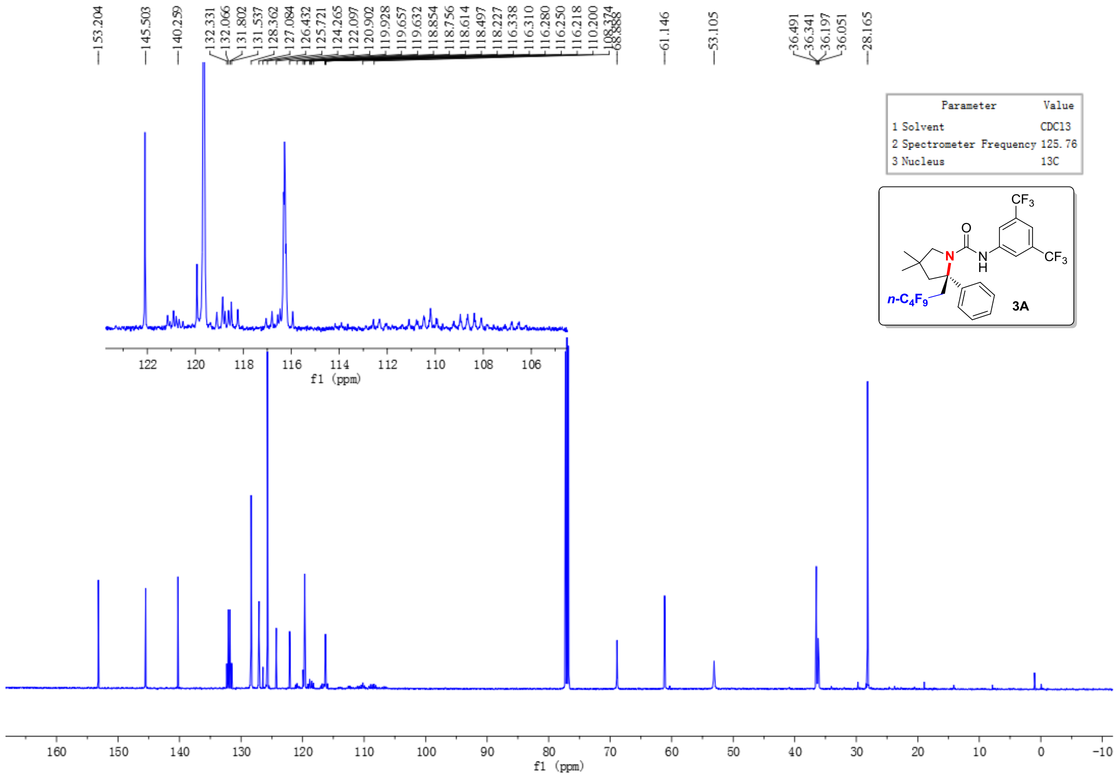

Supplementary Figure 7. ^{13}C NMR of **3A**

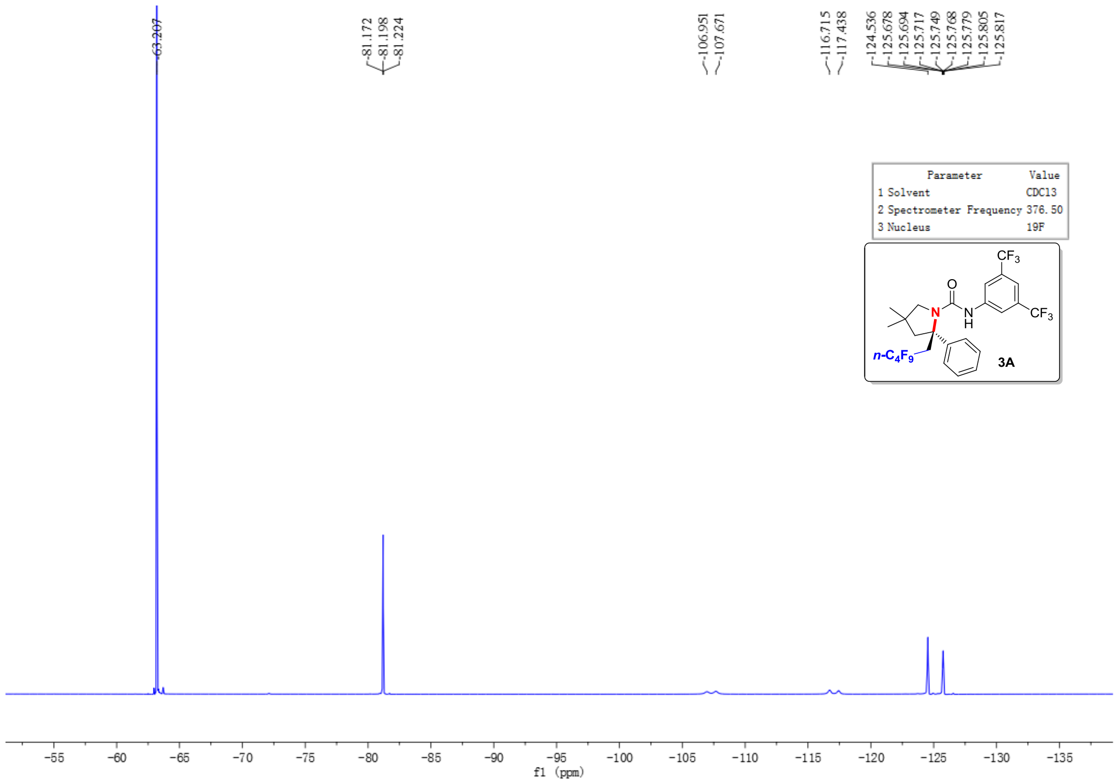

Supplementary Figure 8. ^{19}F NMR of **3A**

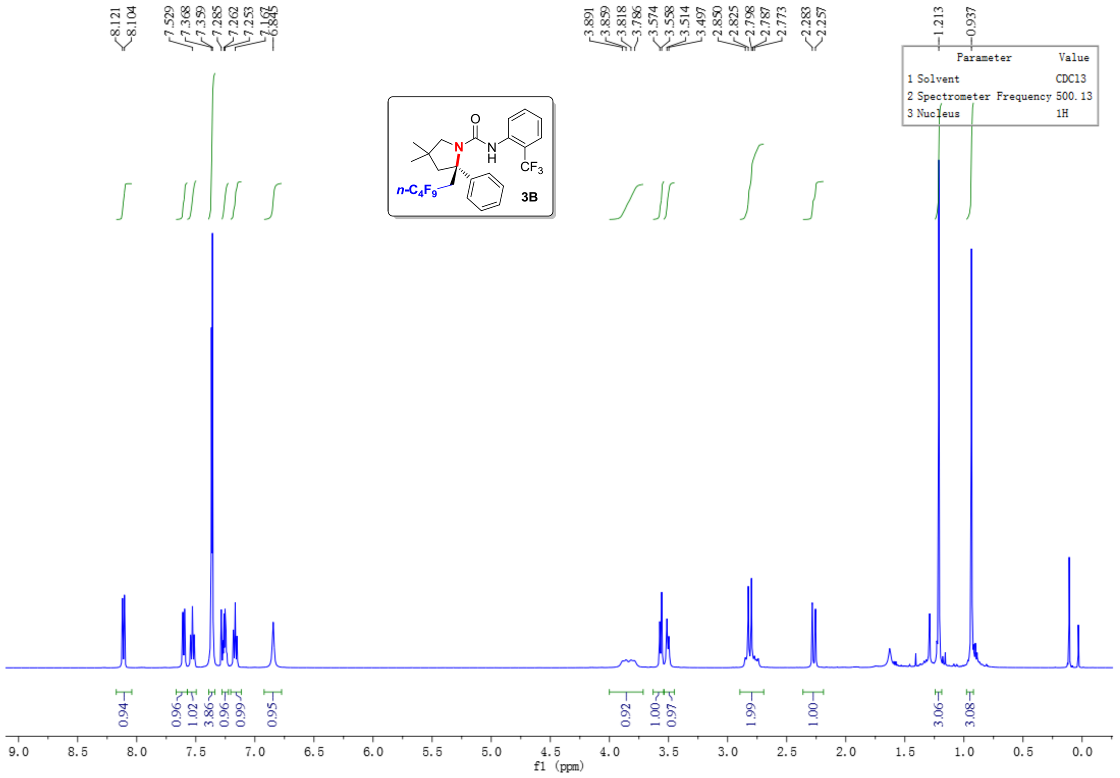

Supplementary Figure 9. ^{1}H NMR of **3B**

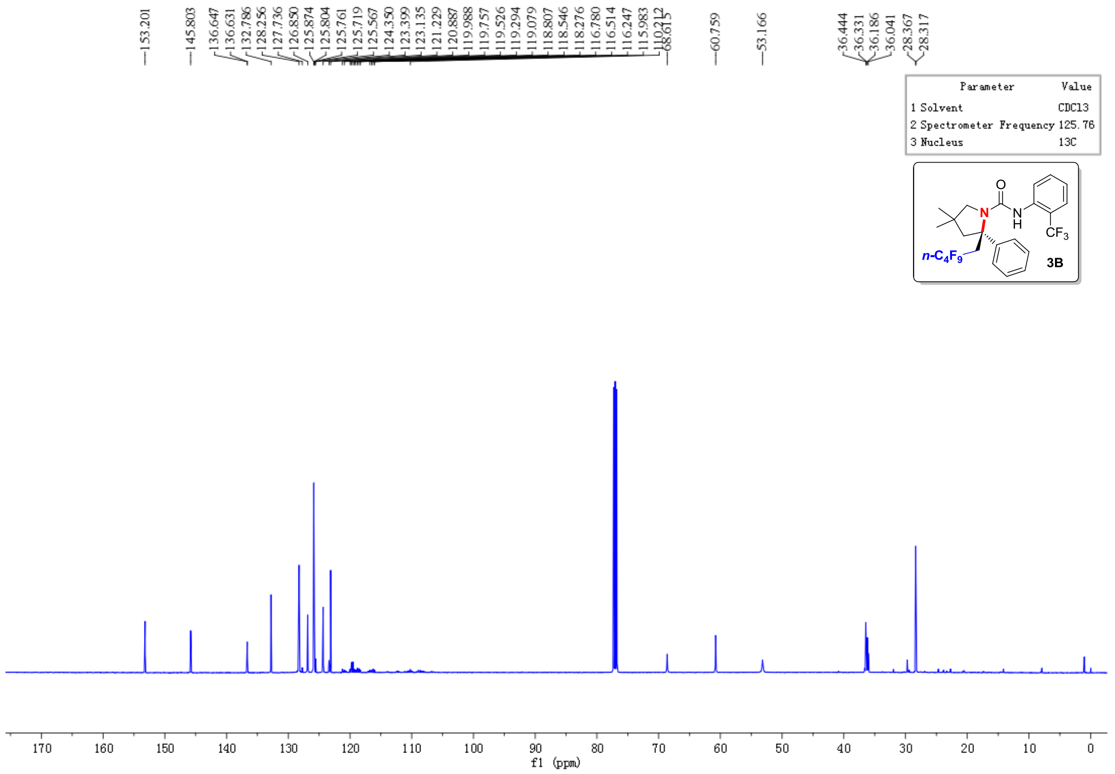

Supplementary Figure 10. ^{13}C NMR of **3B**

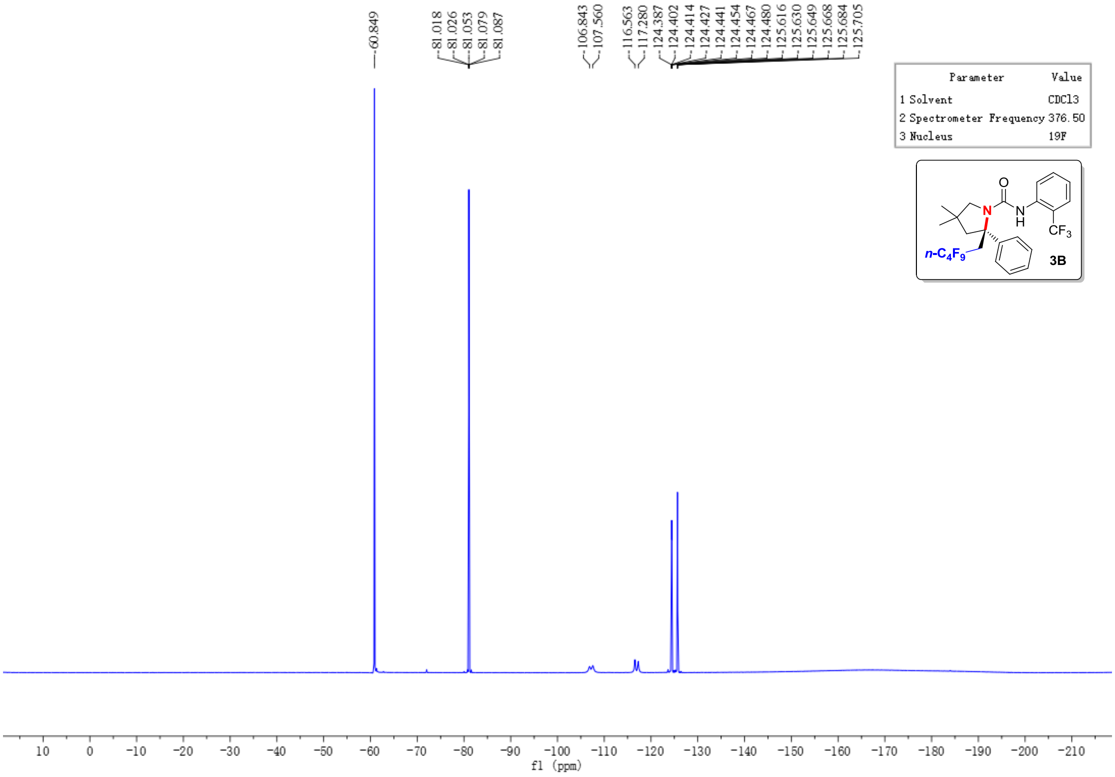

Supplementary Figure 11. ^{19}F NMR of **3B**

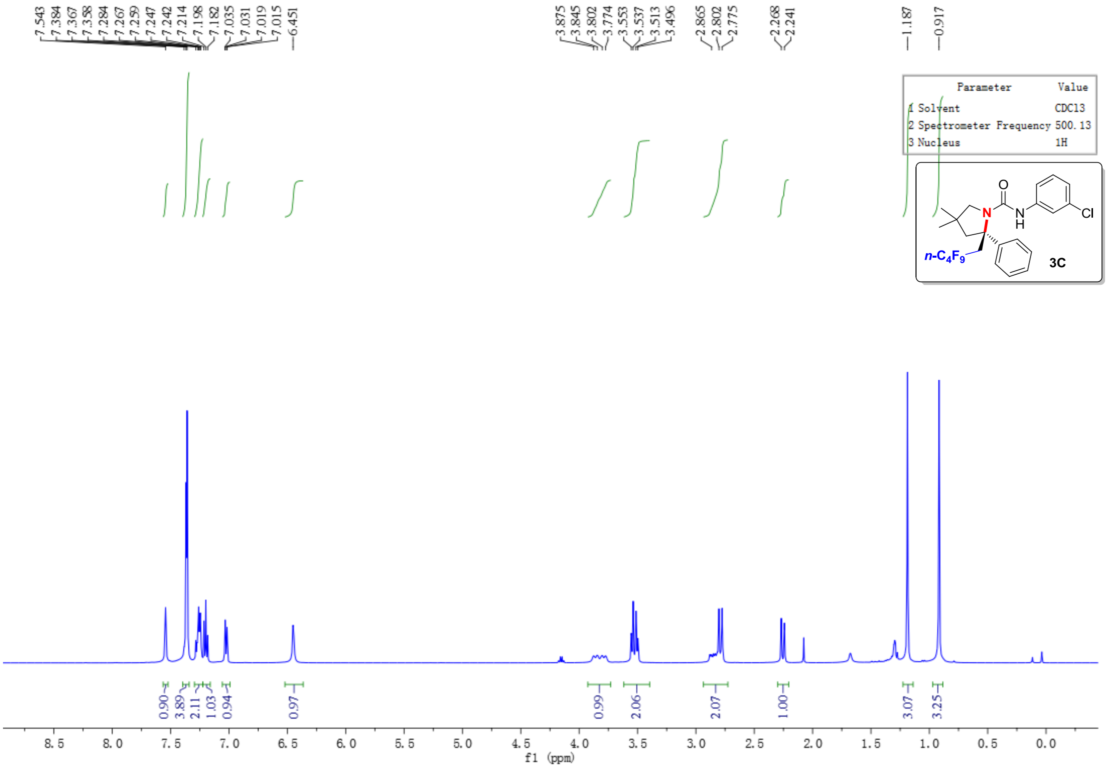

Supplementary Figure 12. ^{1}H NMR of **3C**

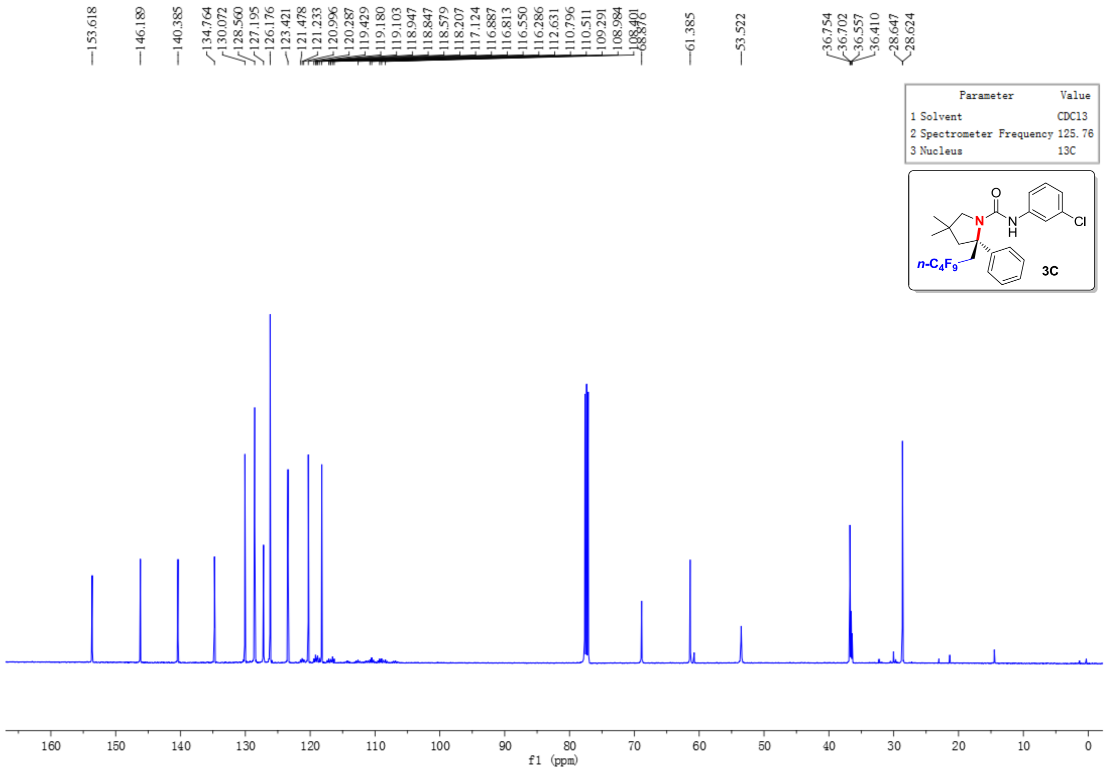

Supplementary Figure 13. ^{13}C NMR of **3C**

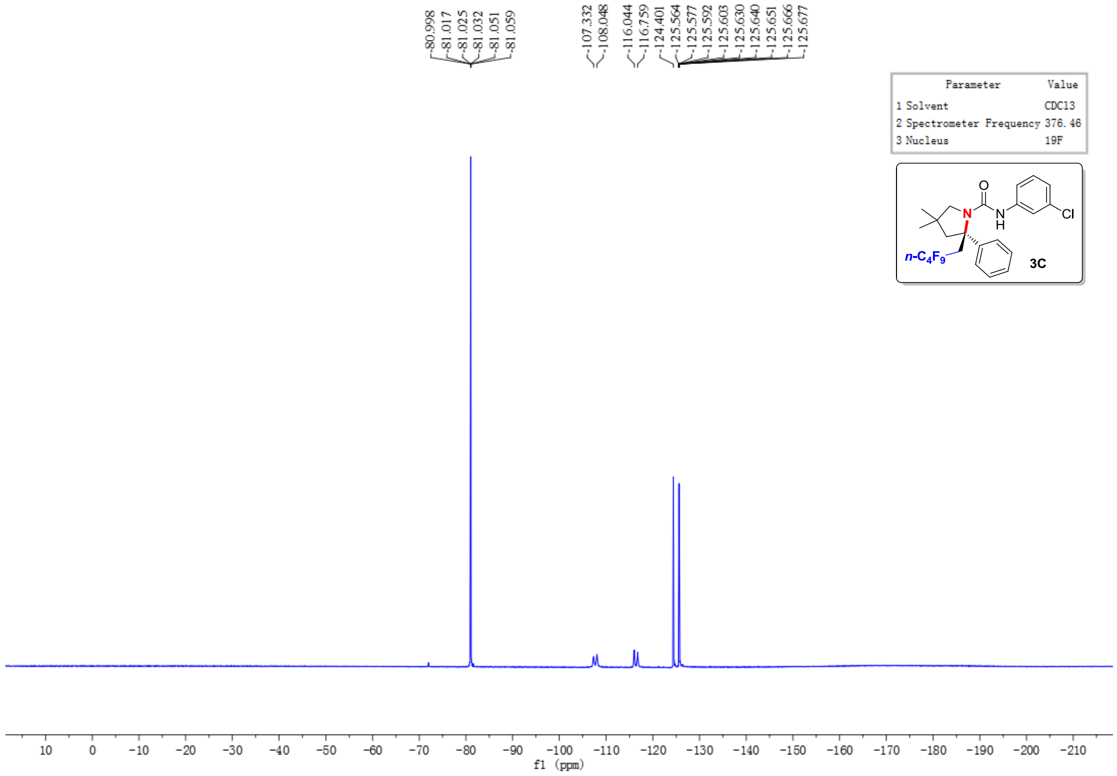

Supplementary Figure 14. ^{19}F NMR of **3C**

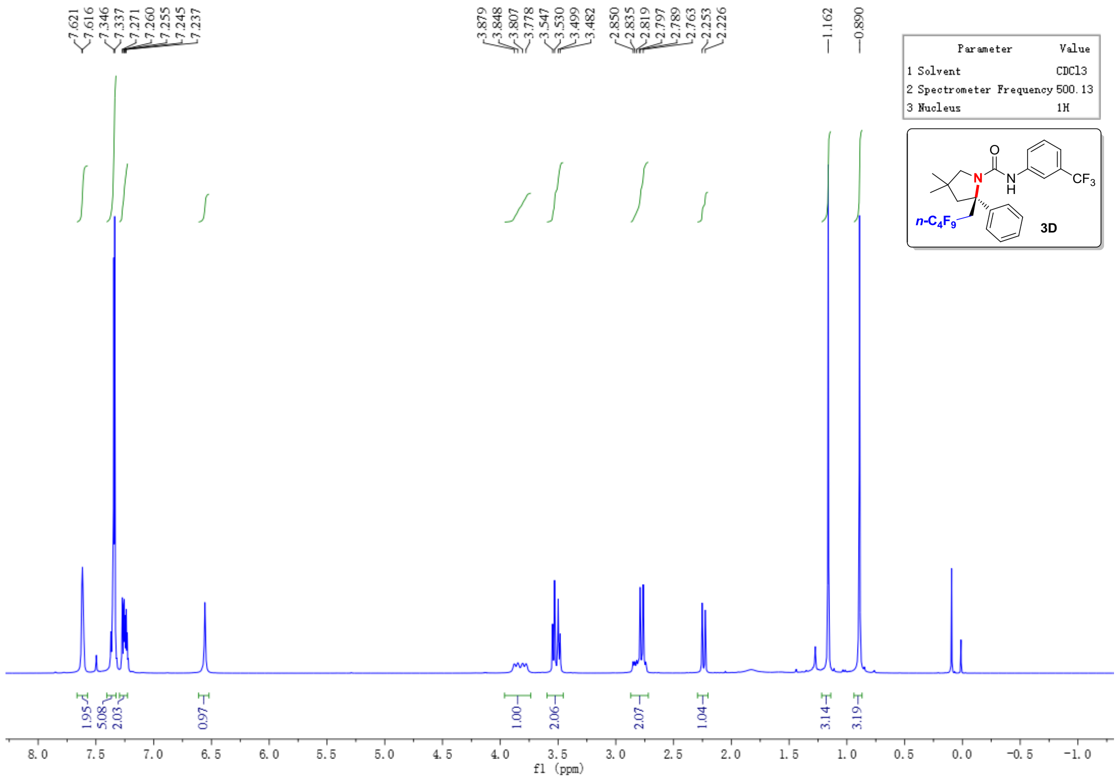

Supplementary Figure 15. ^{1}H NMR of **3D**

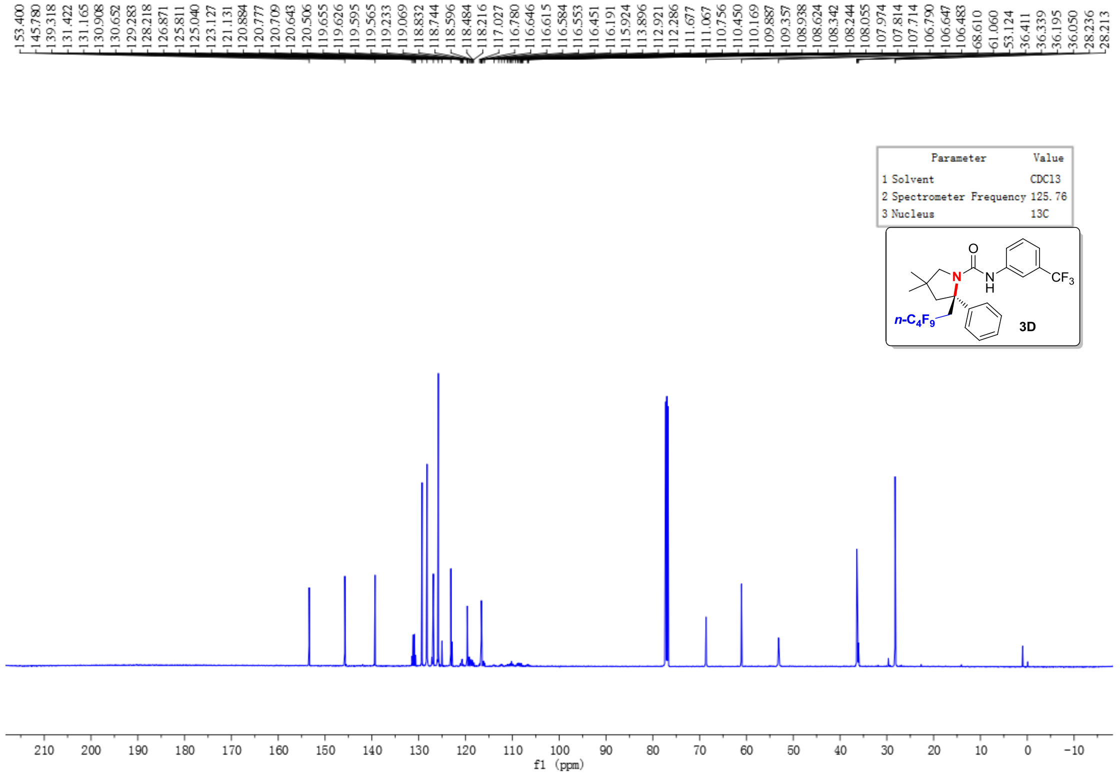

Supplementary Figure 16. ^{13}C NMR of **3D**

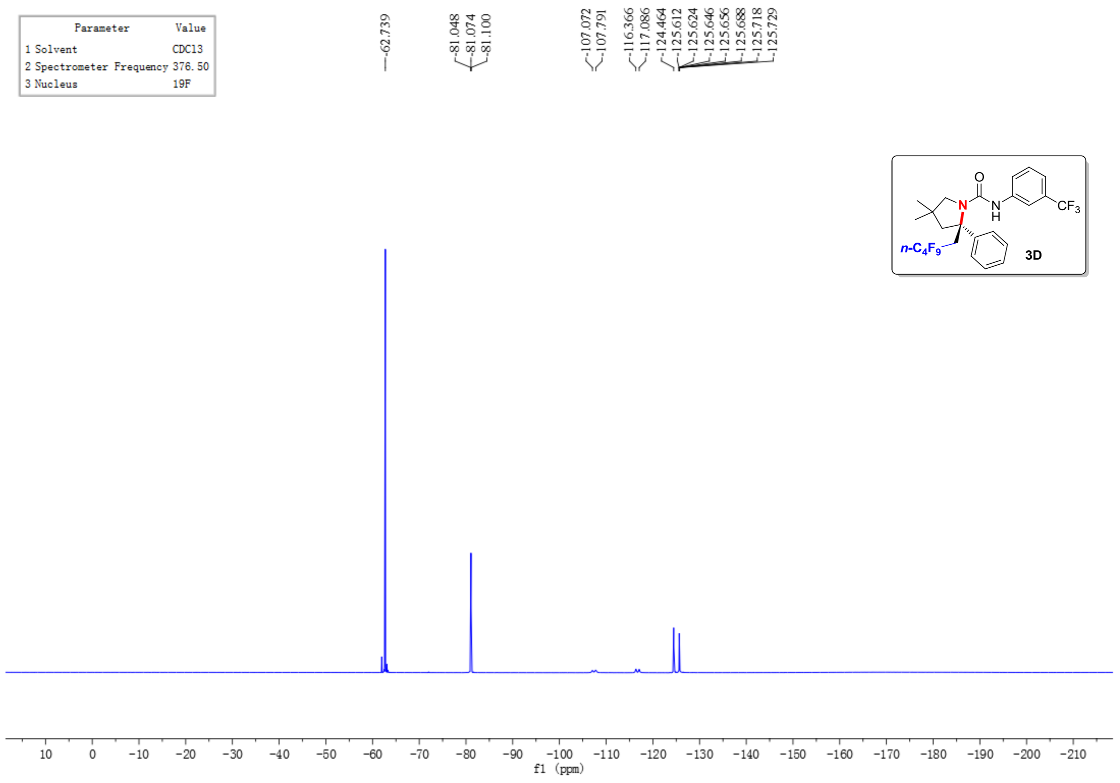

Supplementary Figure 17. ^{19}F NMR of **3D**

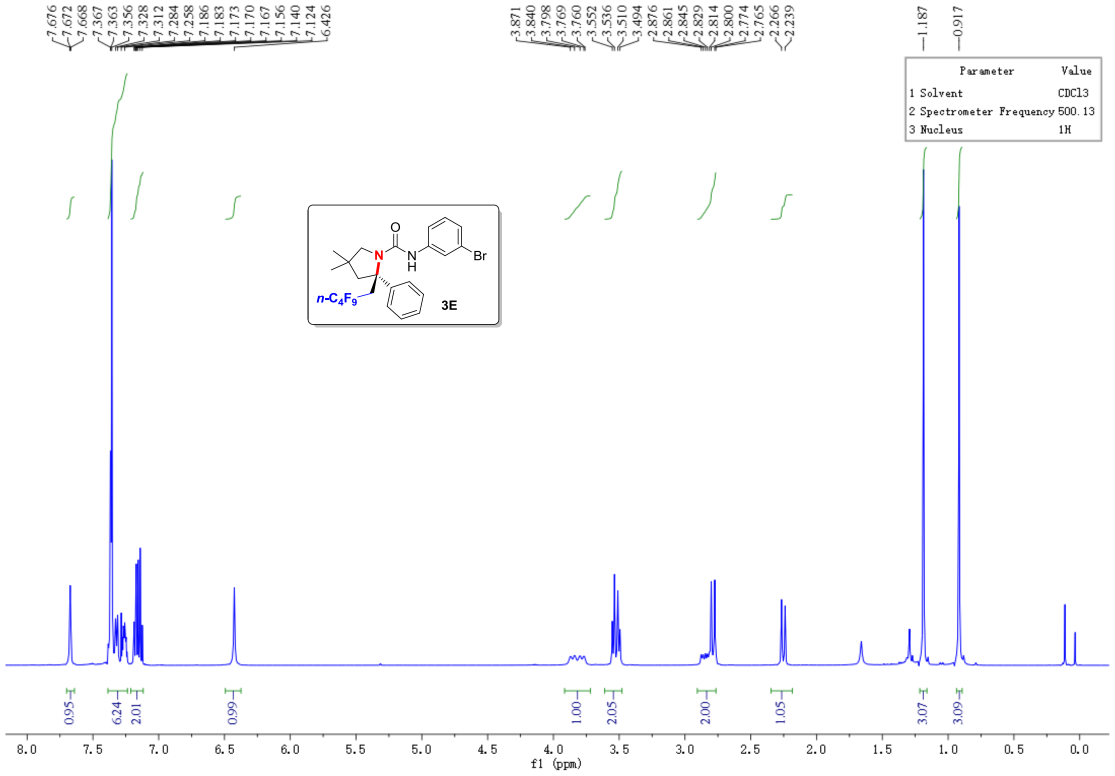

Supplementary Figure 18. ^{1}H NMR of **3E**

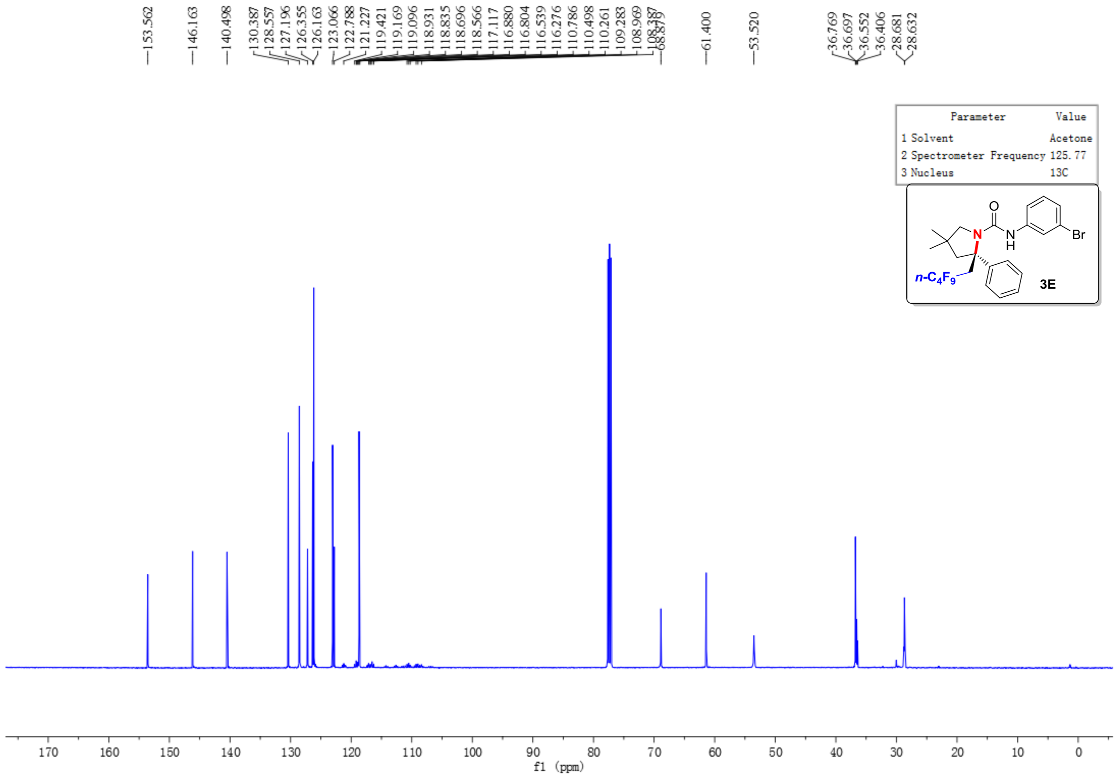

Supplementary Figure 19. ^{13}C NMR of **3E**

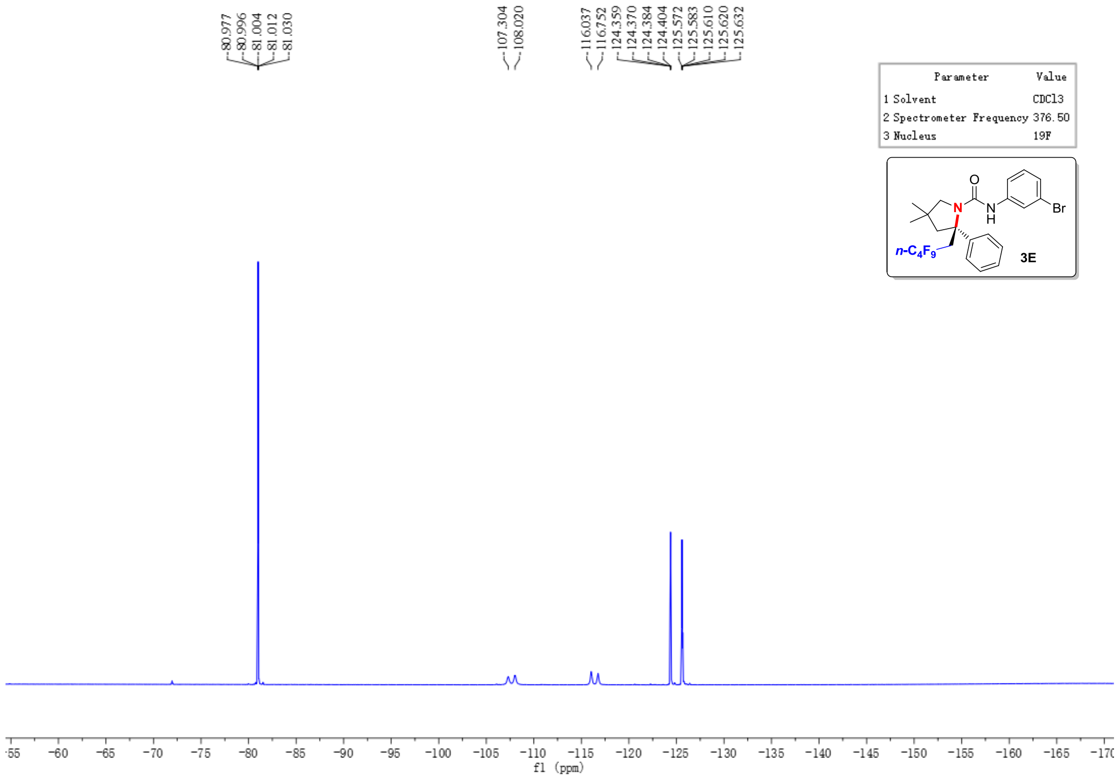

Supplementary Figure 20. ^{19}F NMR of **3E**

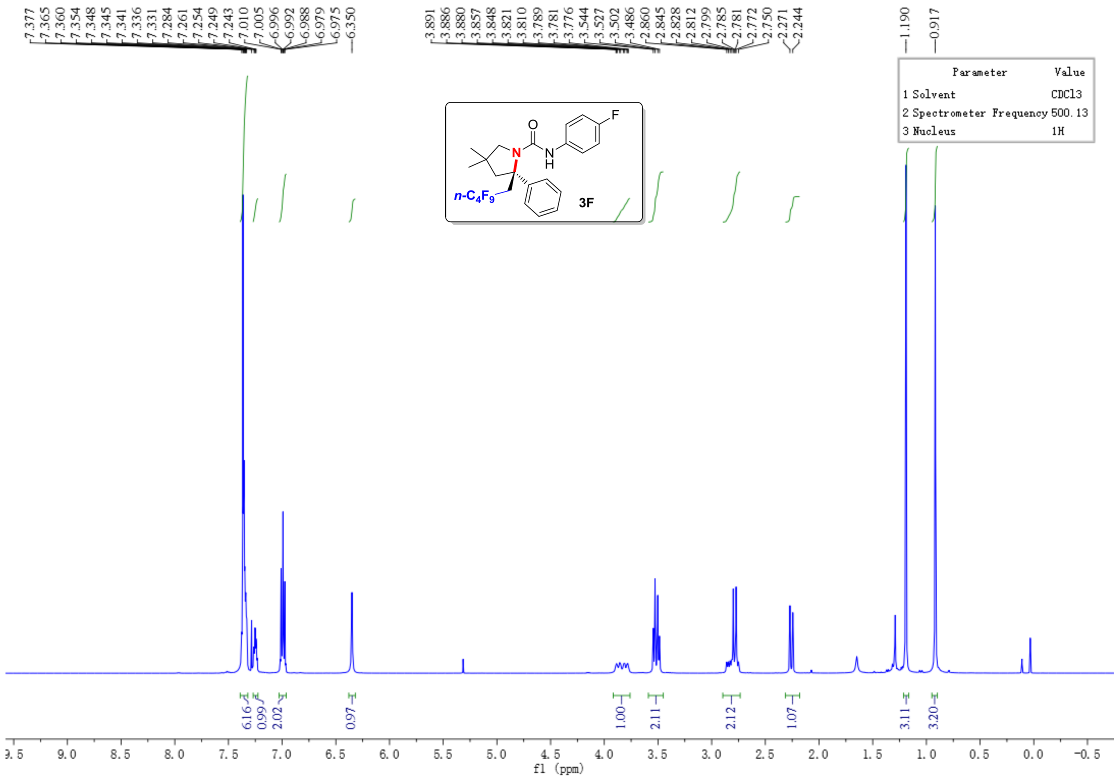

Supplementary Figure 21. ^{1}H NMR of **3F**

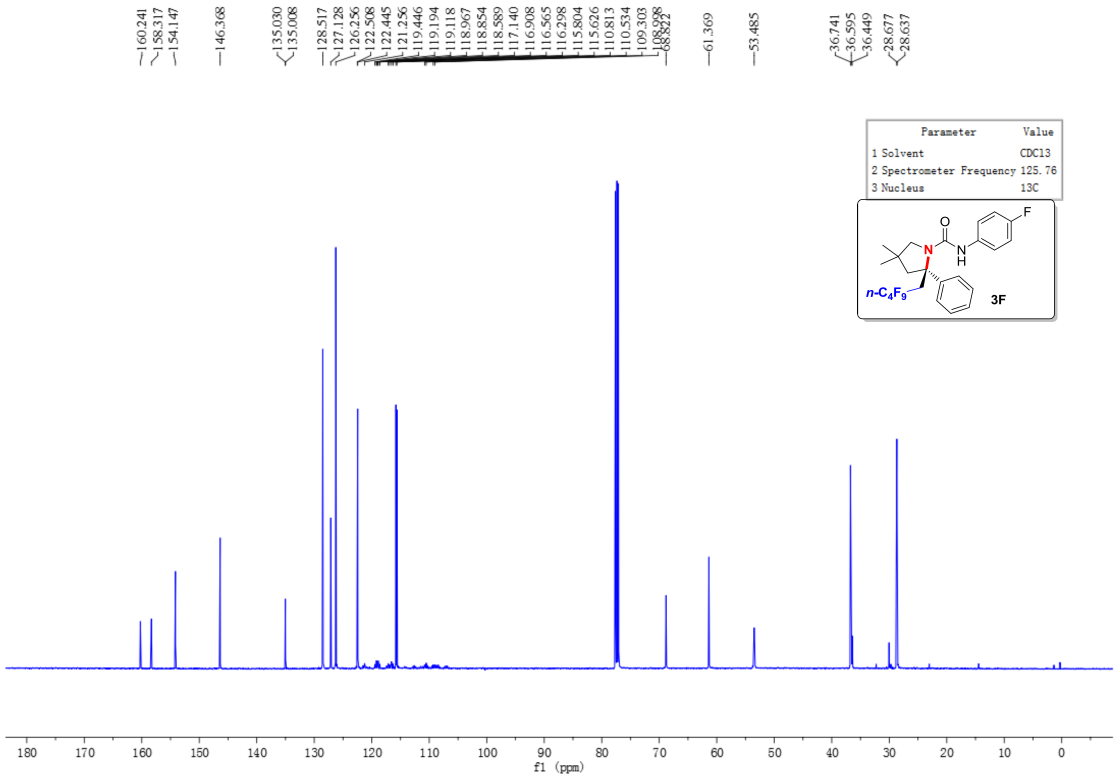

Supplementary Figure 22. ^{13}C NMR of **3F**

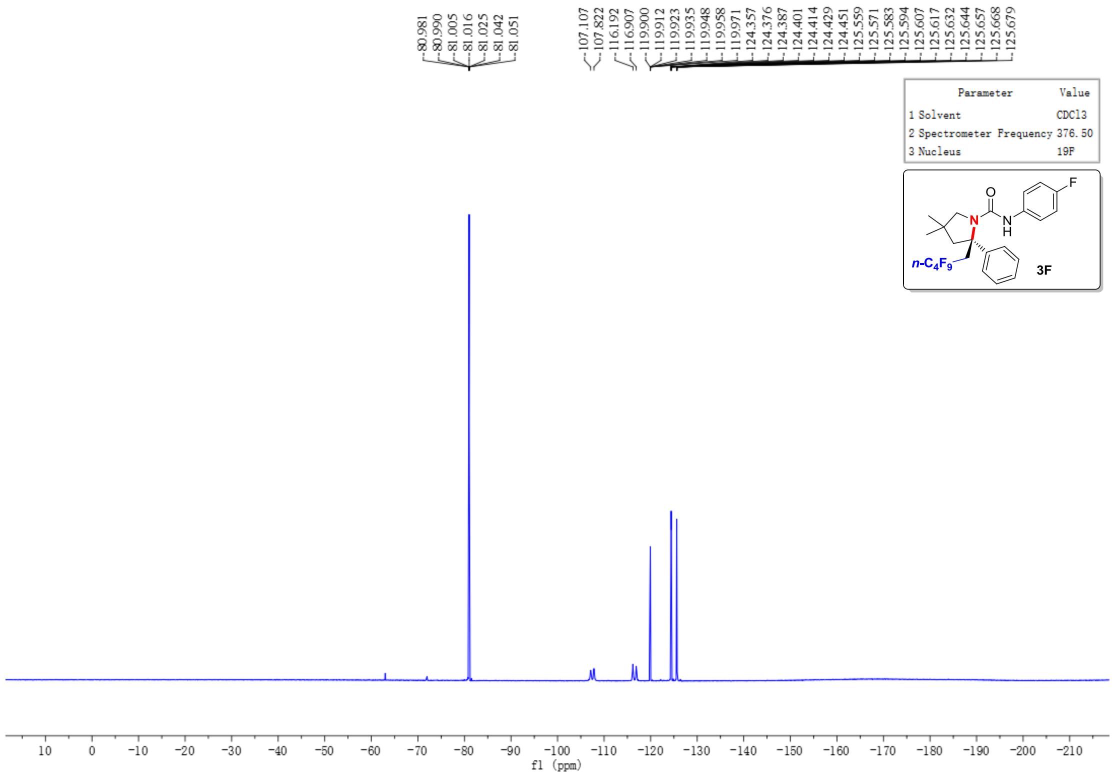

Supplementary Figure 23. ^{19}F NMR of **3F**

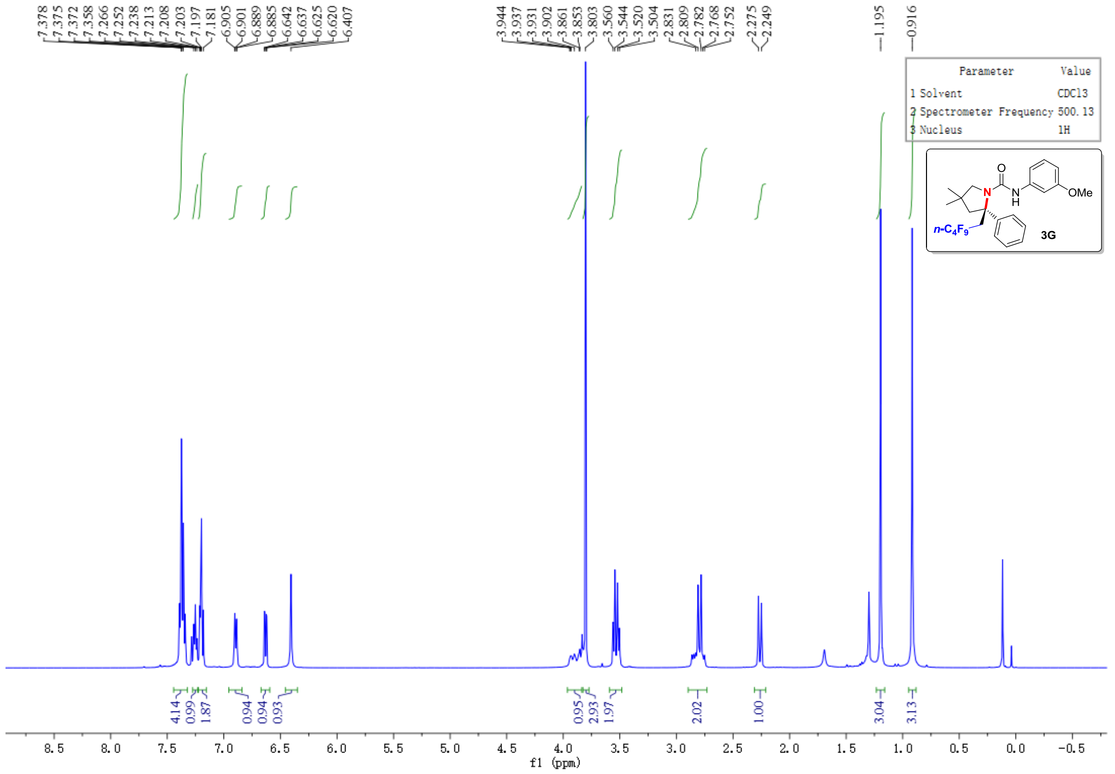

Supplementary Figure 24. ^{1}H NMR of **3G**

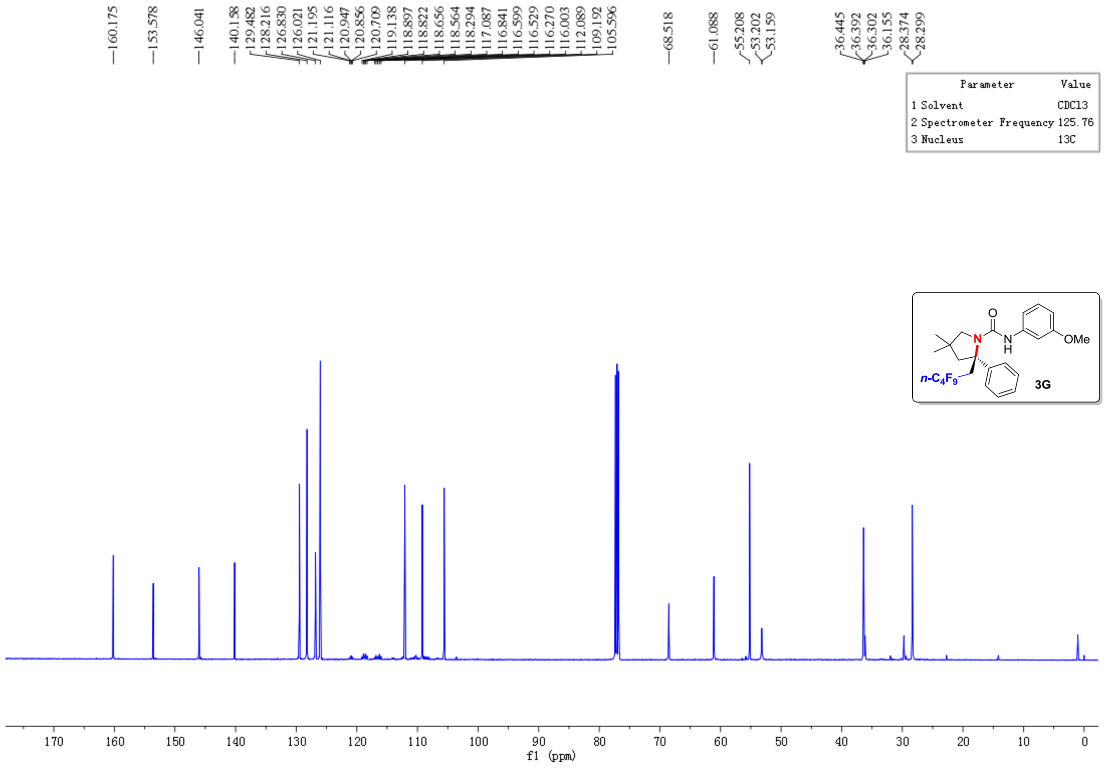

Supplementary Figure 25. ^{13}C NMR of **3G**

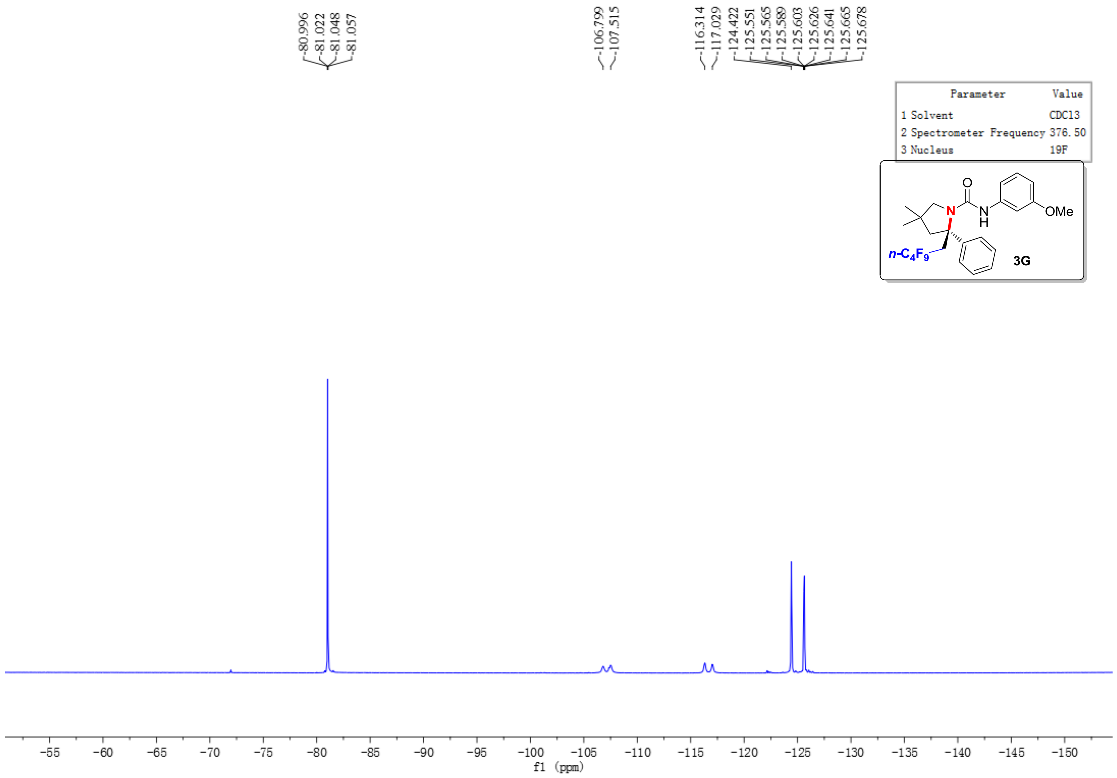

Supplementary Figure 26. ^{19}F NMR of **3G**

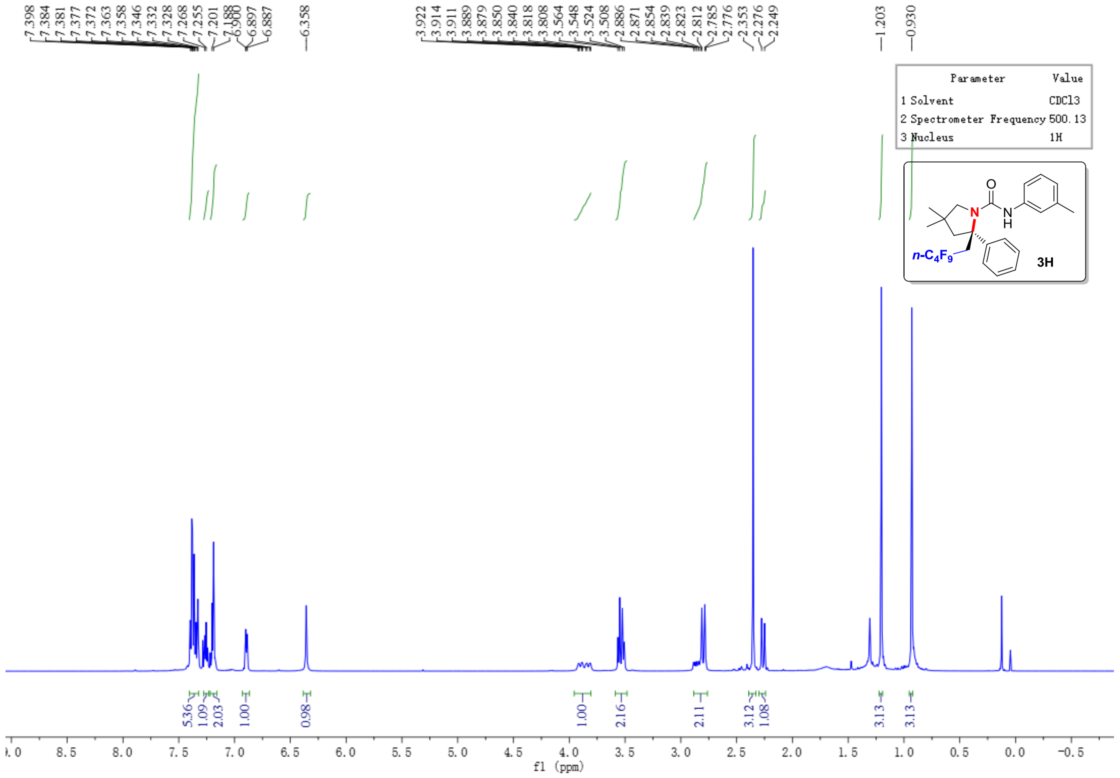

Supplementary Figure 27. ^{1}H NMR of **3H**

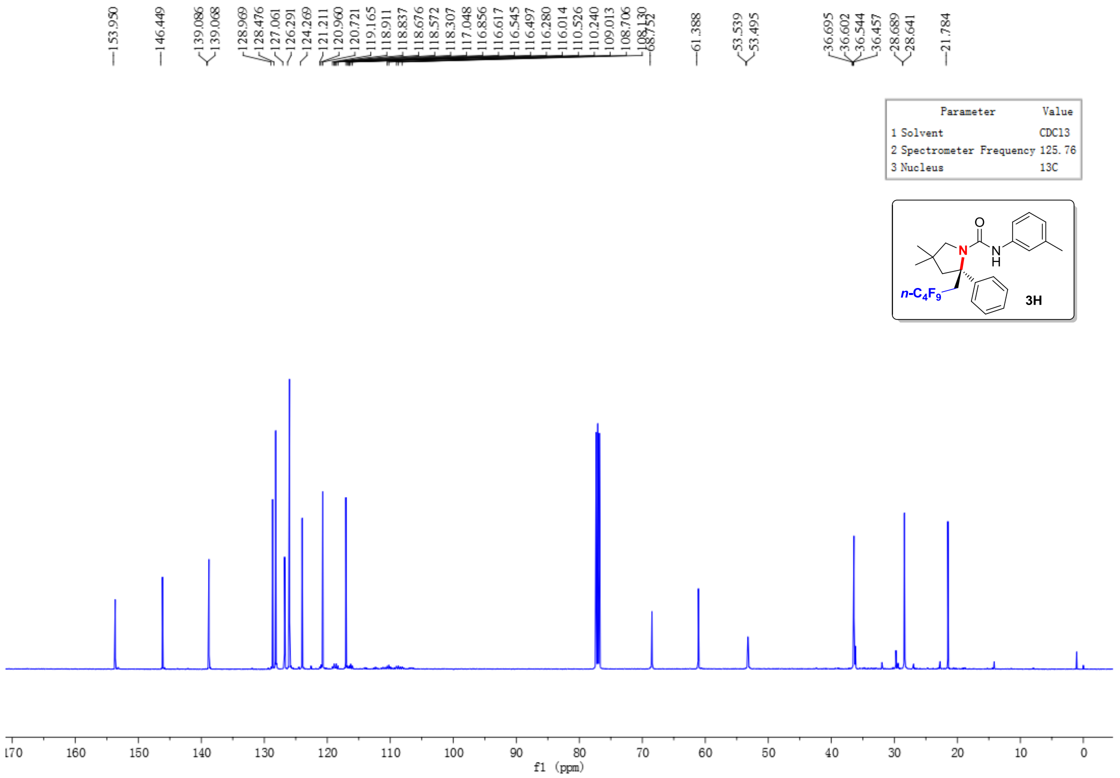

Supplementary Figure 28. ^{13}C NMR of **3H**

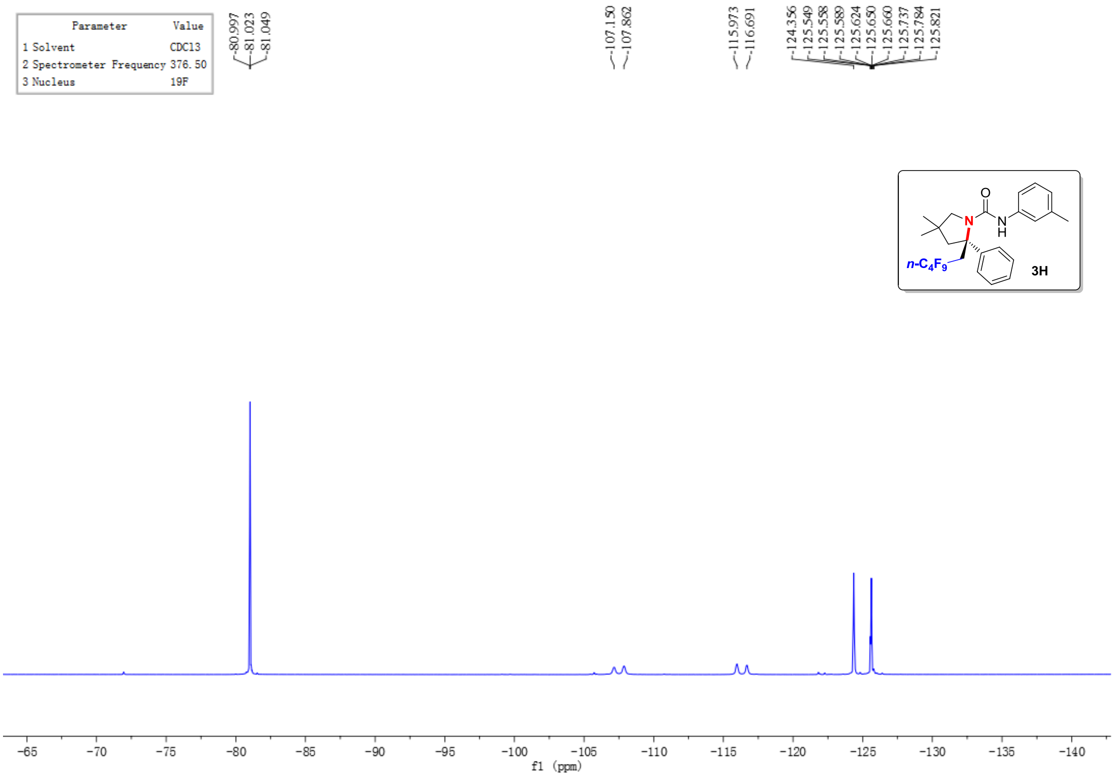

Supplementary Figure 29. ^{19}F NMR of **3H**

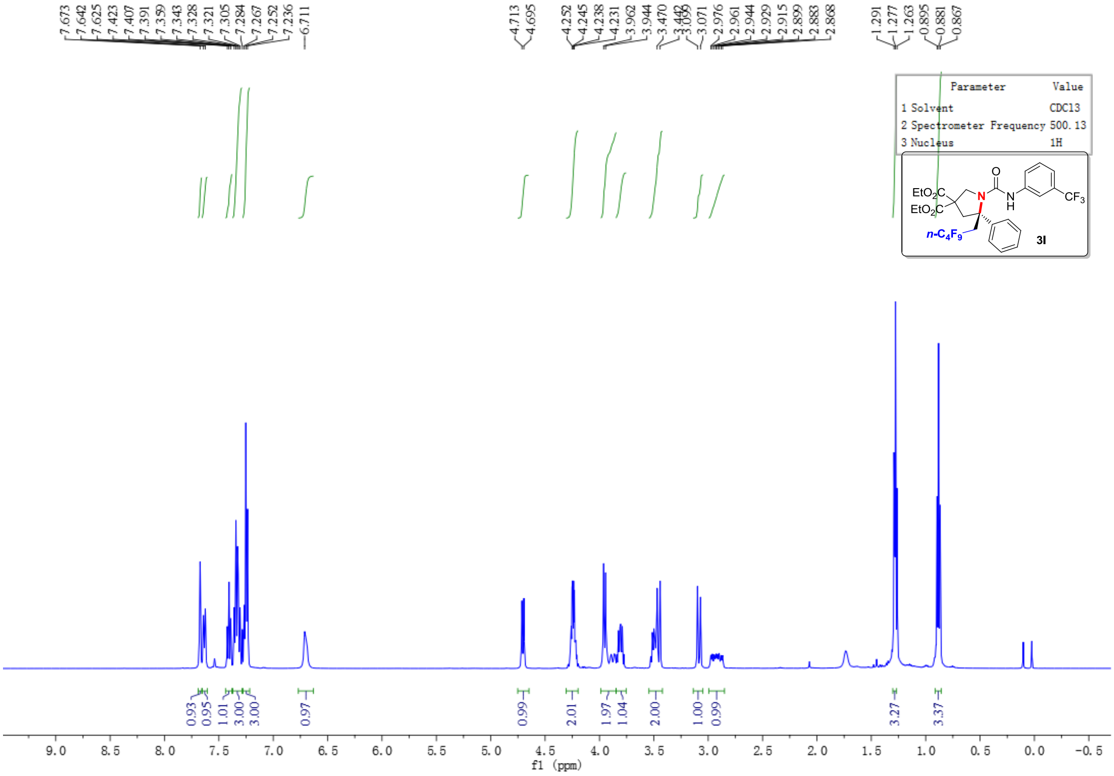

Supplementary Figure 30. ^{1}H NMR of **3I**

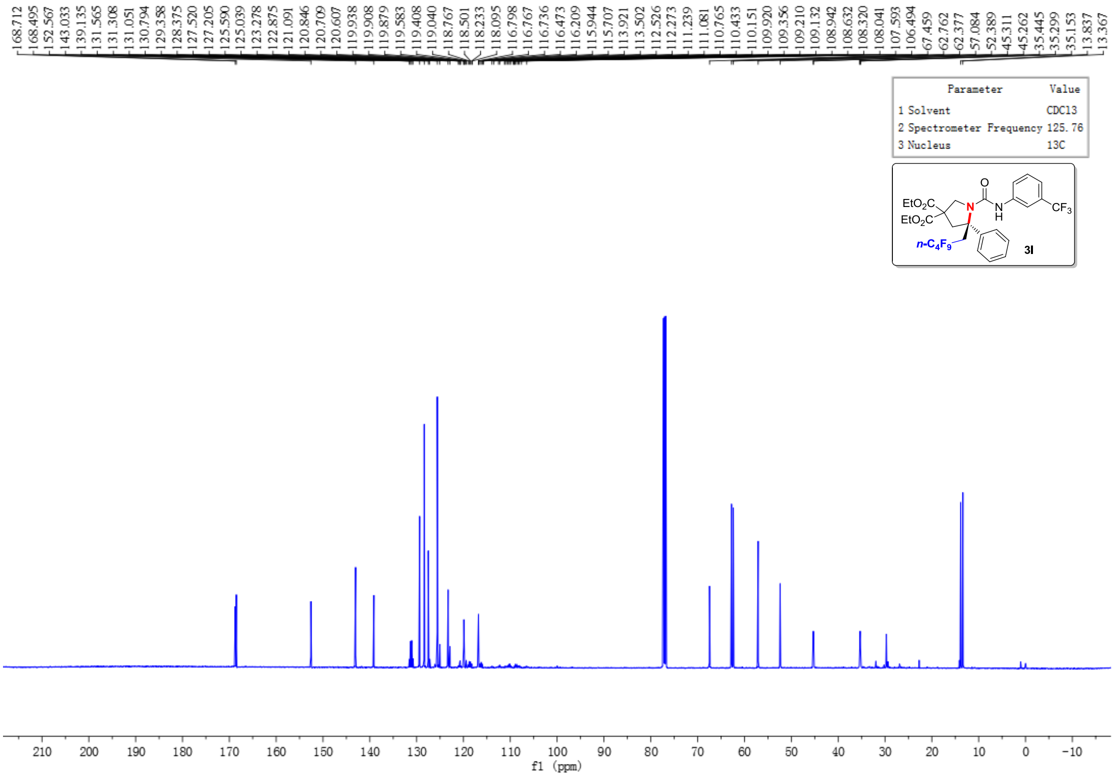

Supplementary Figure 31. ^{13}C NMR of **3l**

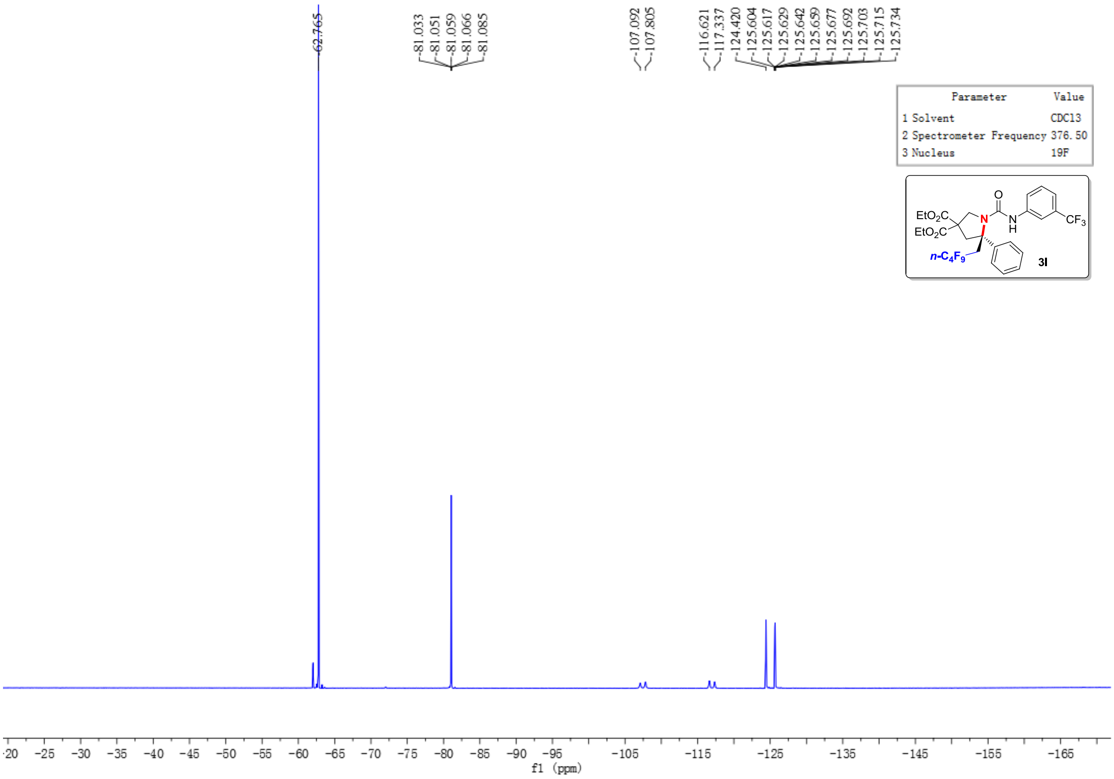

Supplementary Figure 32. ^{19}F NMR of **3l**

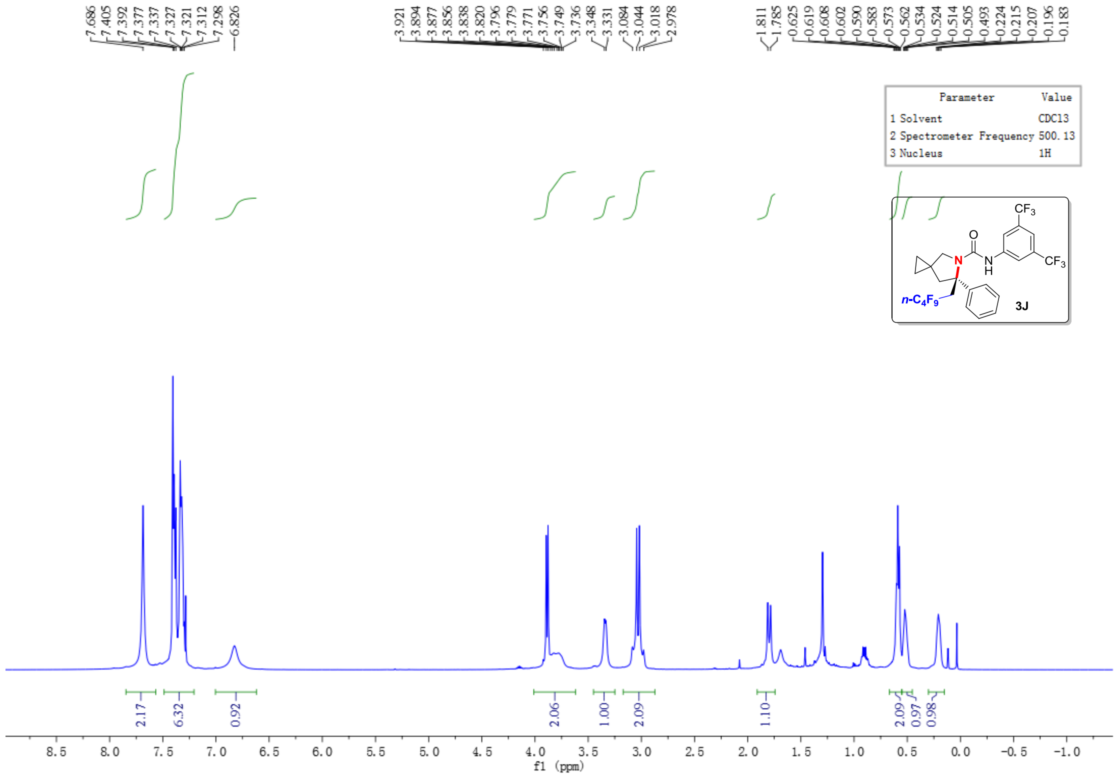

Supplementary Figure 33. ^{1}H NMR of **3J**

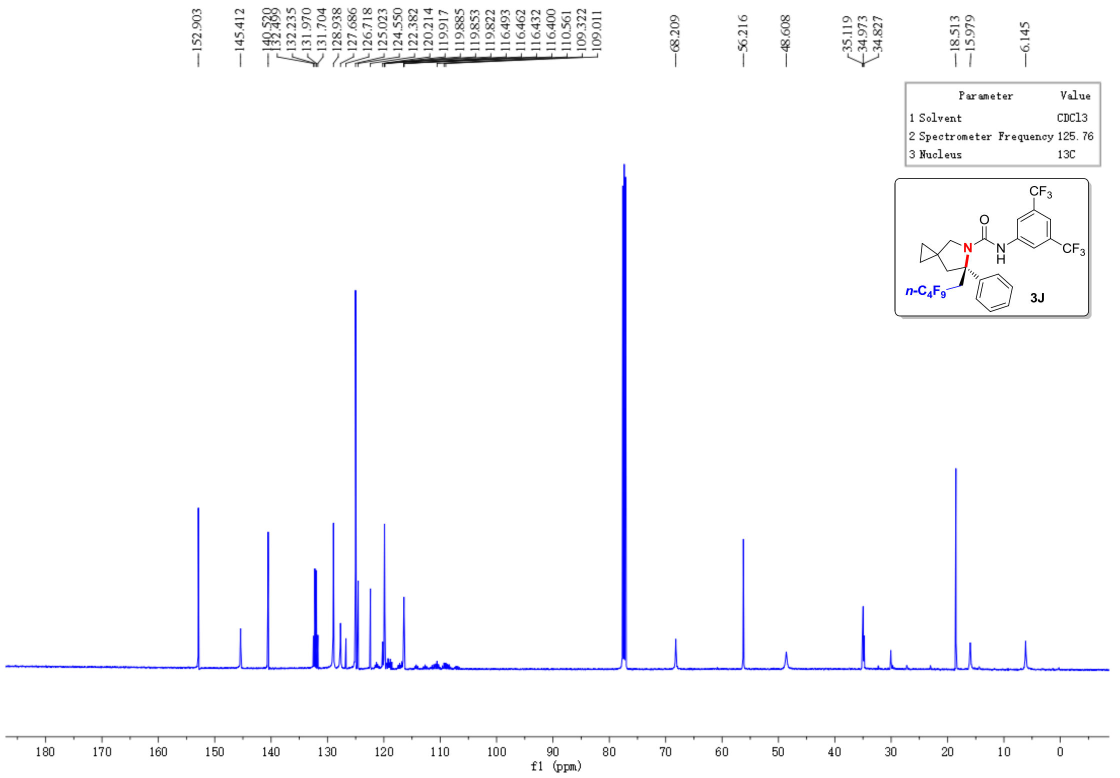

Supplementary Figure 34. ^{13}C NMR of **3J**

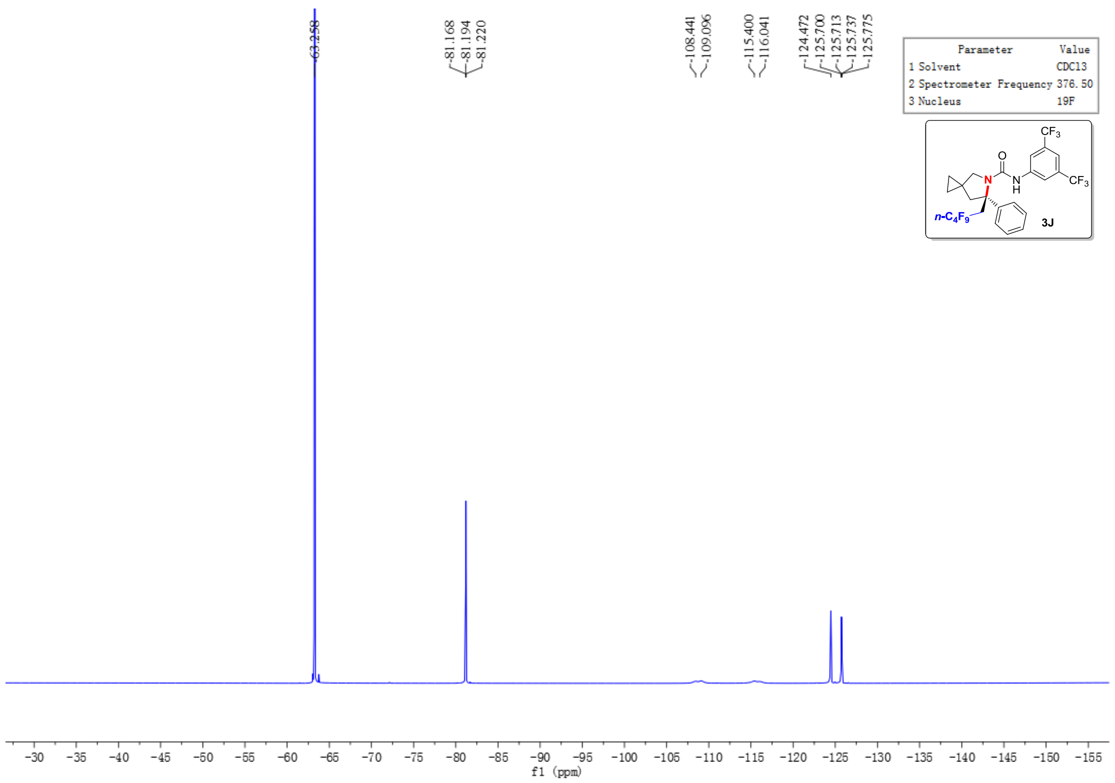

Supplementary Figure 35. ^{19}F NMR of **3J**

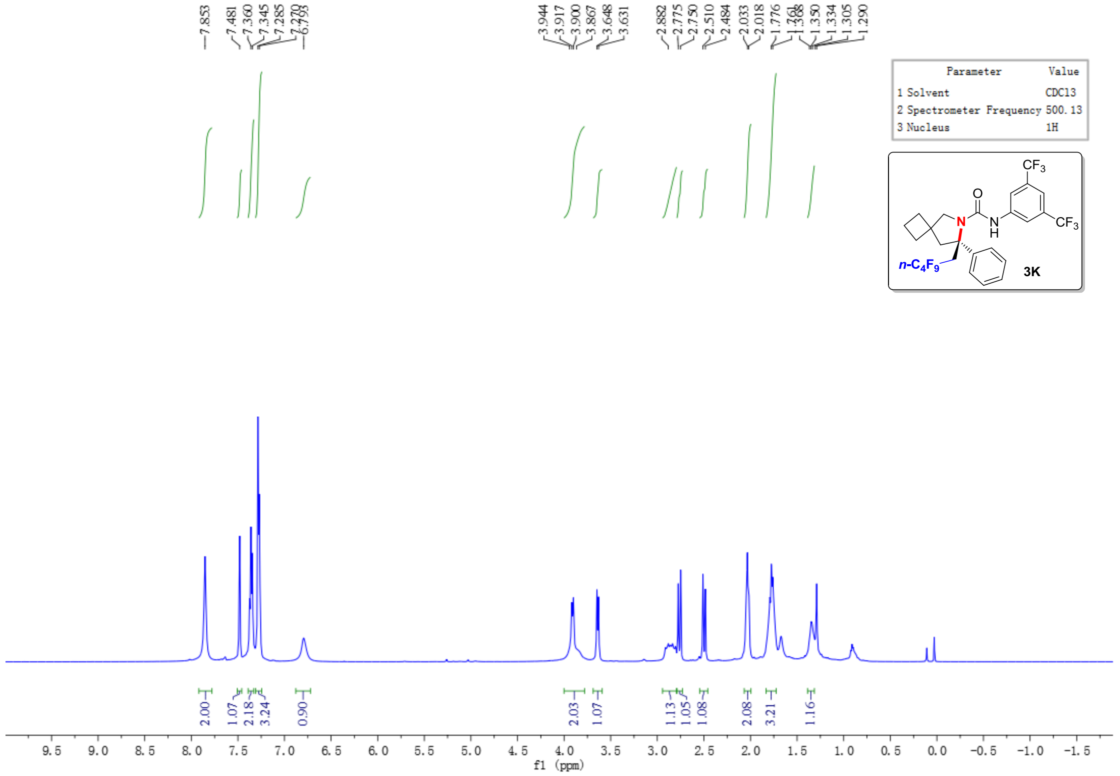

Supplementary Figure 36. ^{1}H NMR of **3K**

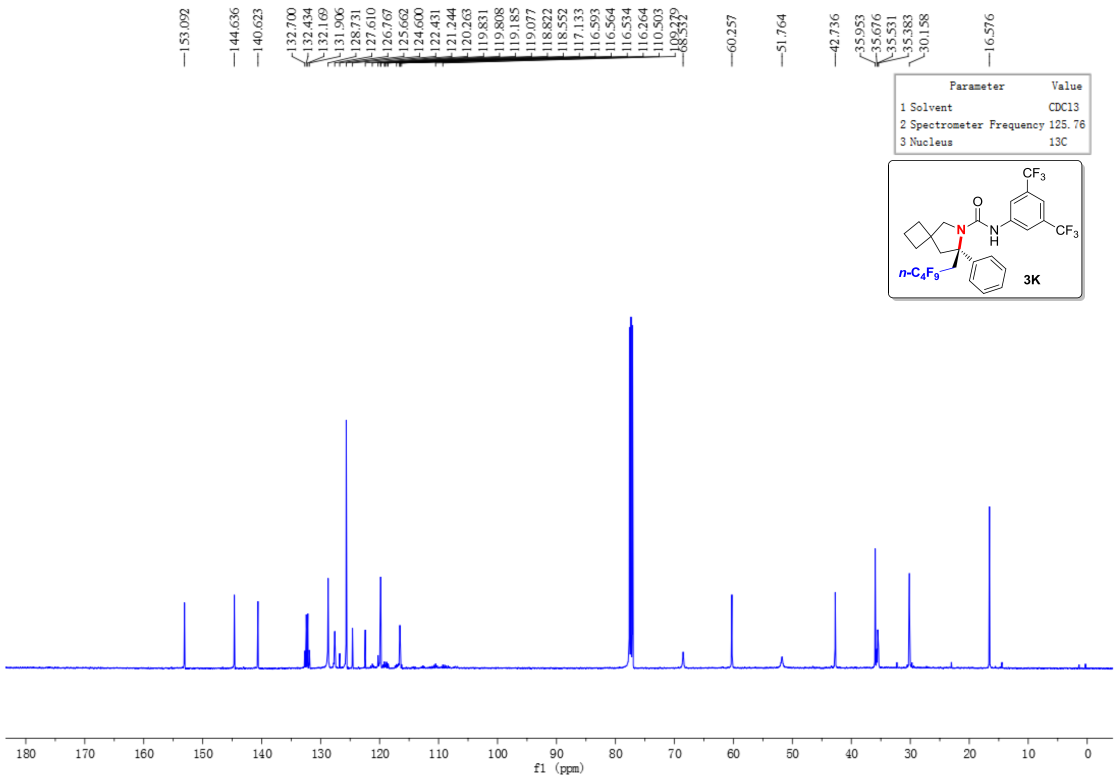

Supplementary Figure 37. ^{13}C NMR of **3K**

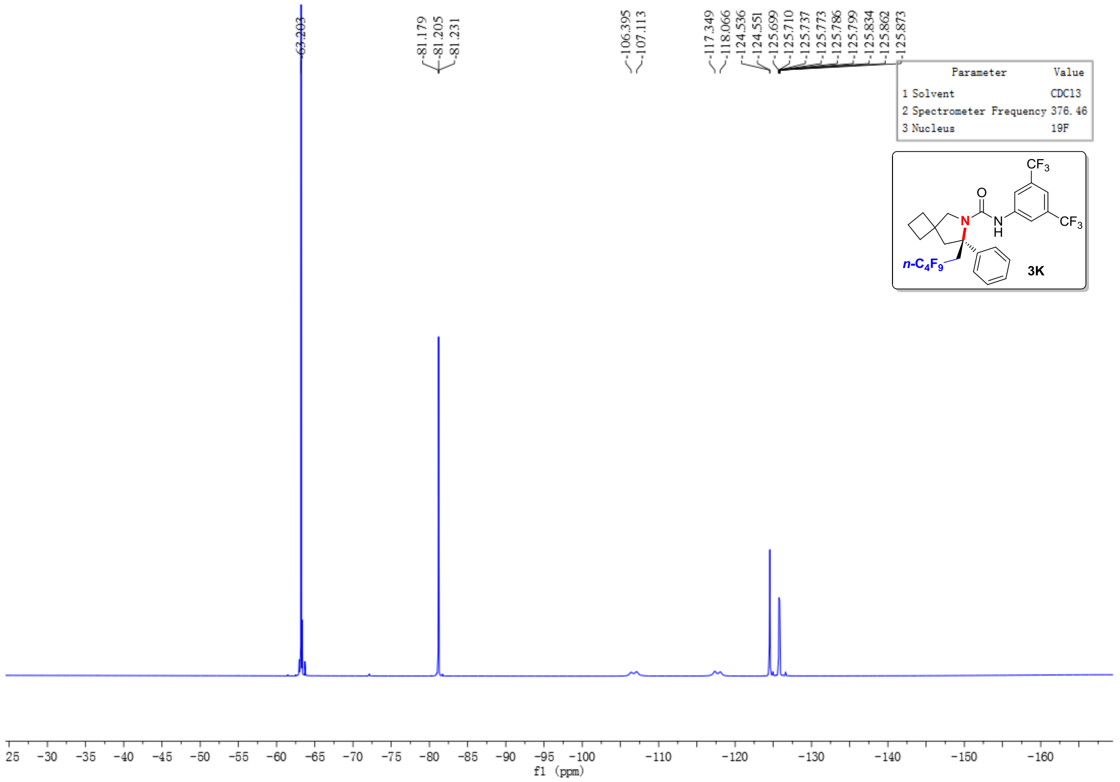

Supplementary Figure 38. ^{19}F NMR of **3K**

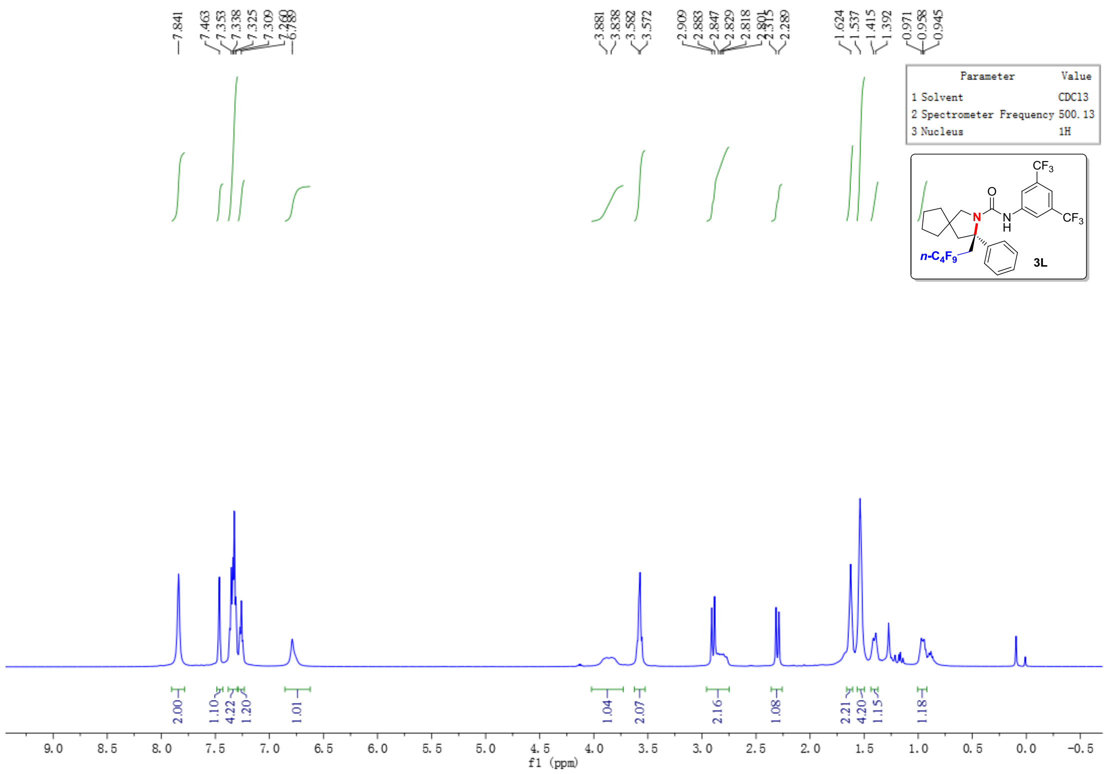

Supplementary Figure 39. ^{1}H NMR of **3L**

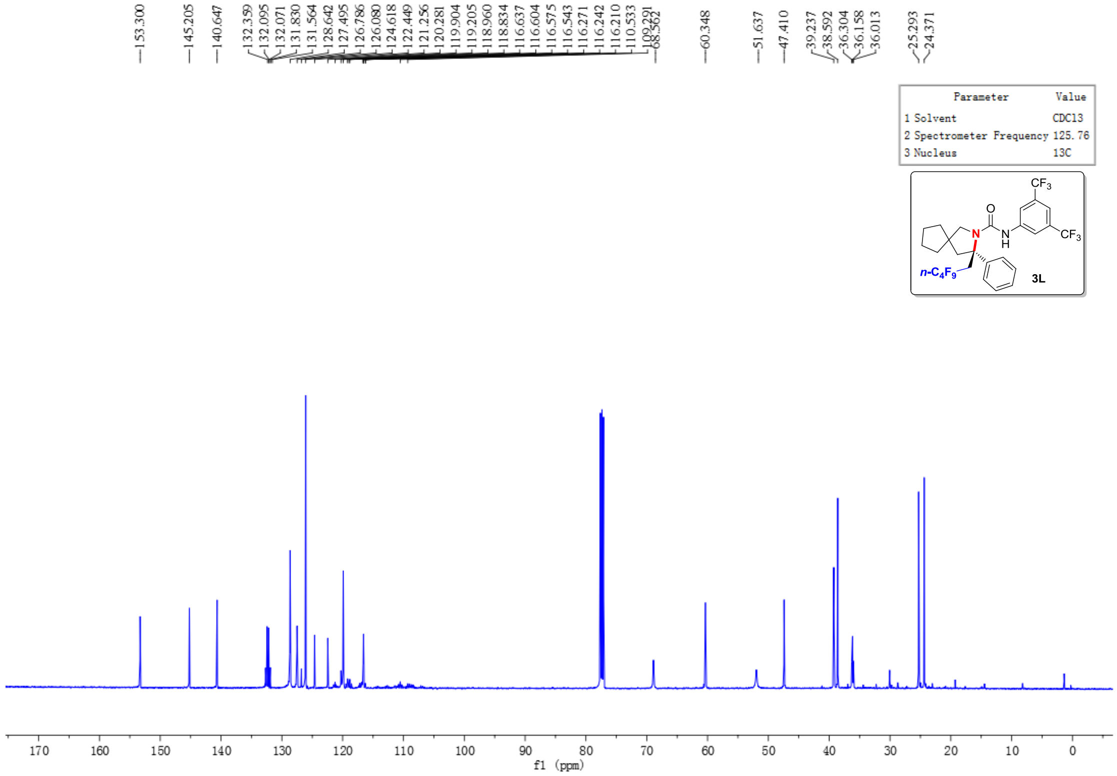

Supplementary Figure 40. ^{13}C NMR of **3L**

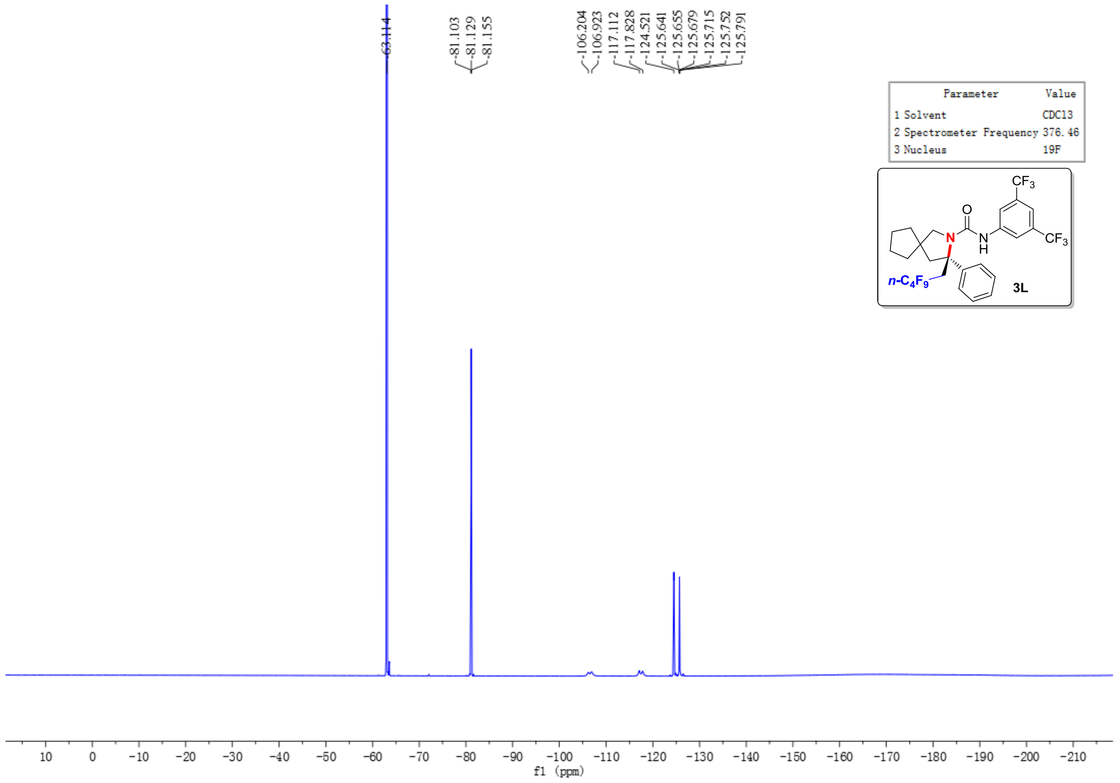

Supplementary Figure 41. ^{19}F NMR of **3L**

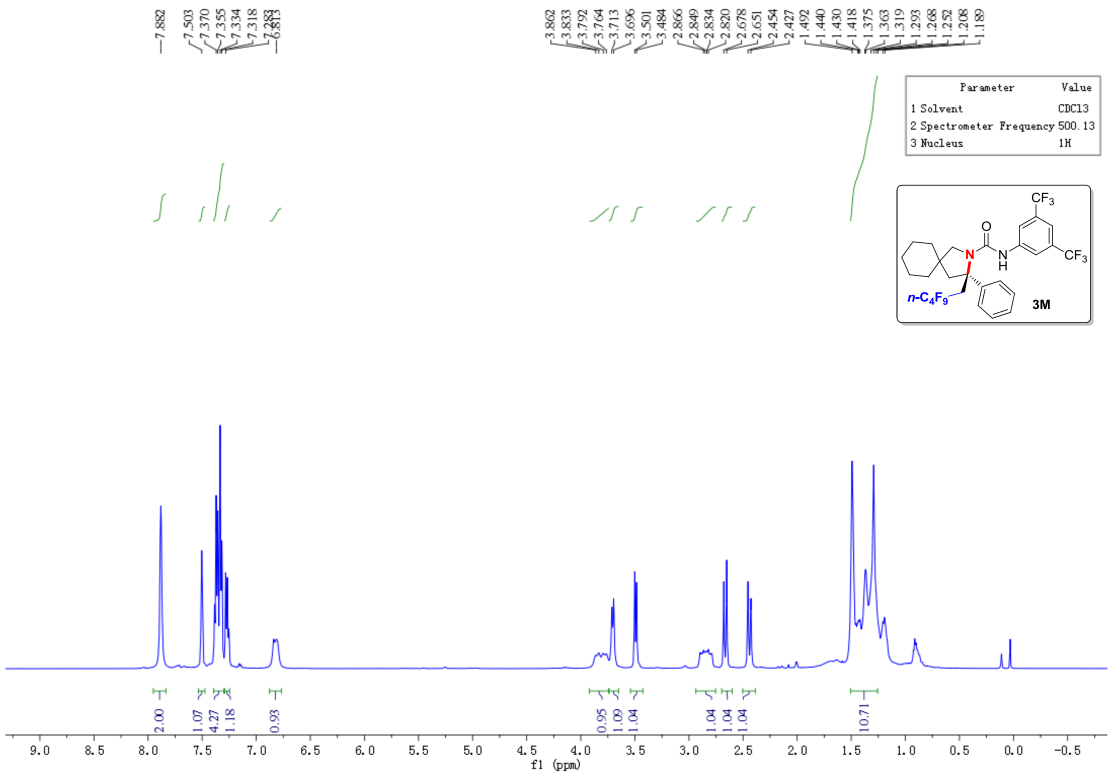

Supplementary Figure 42. ^{1}H NMR of **3M**

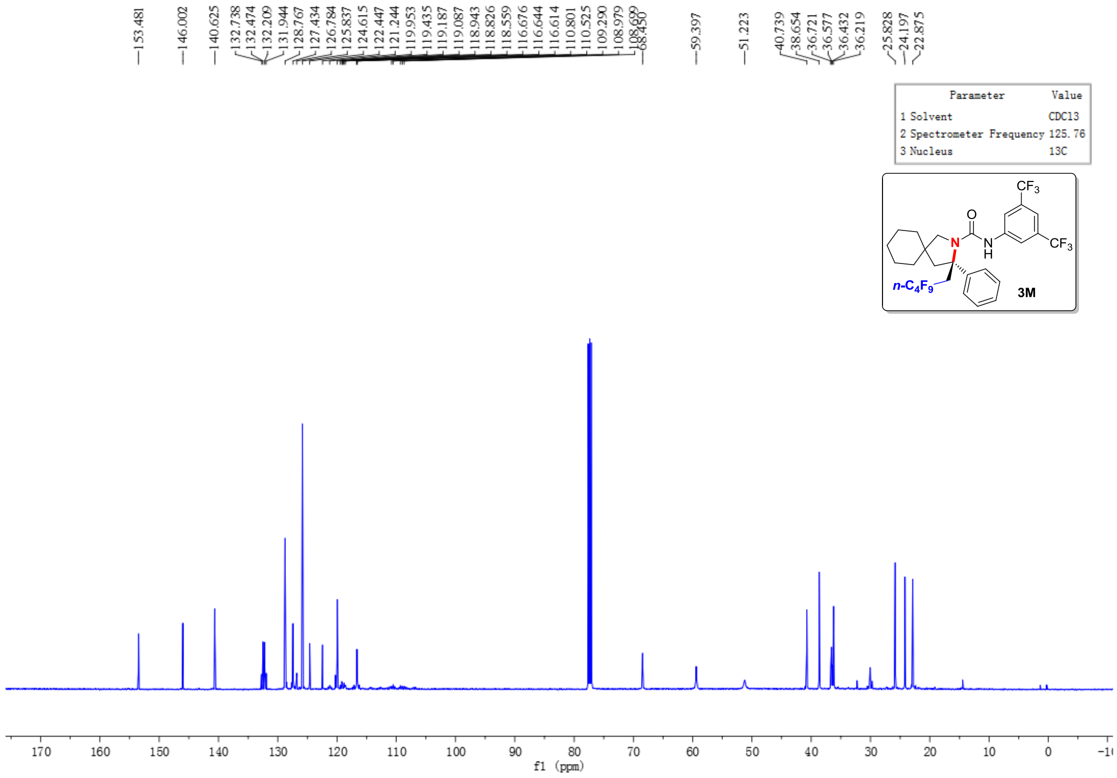

Supplementary Figure 43. ^{13}C NMR of **3M**

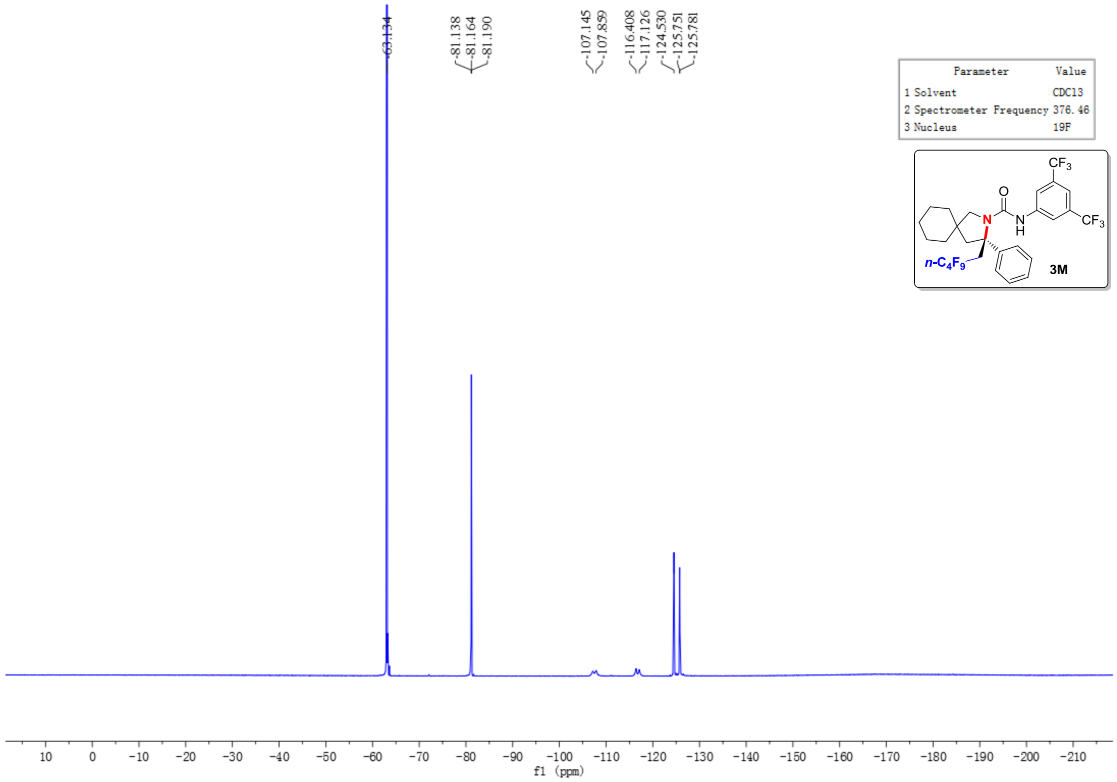

Supplementary Figure 44. ^{19}F NMR of **3M**

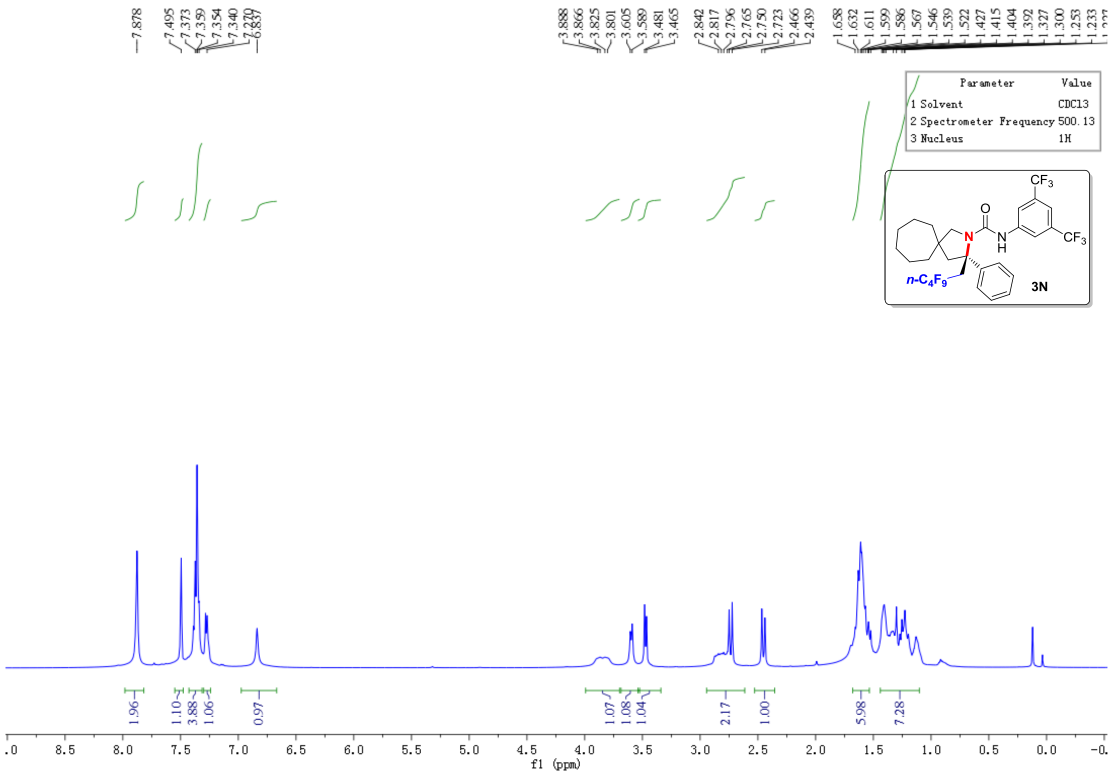

Supplementary Figure 45. ^{1}H NMR of **3N**

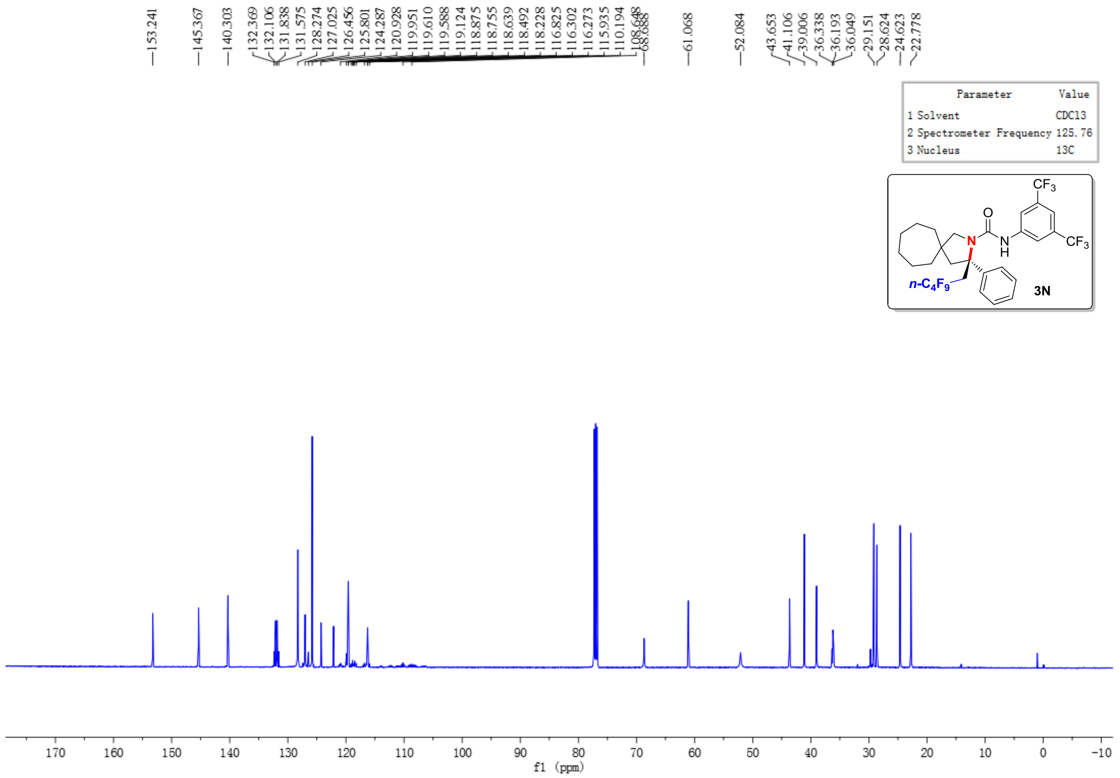

Supplementary Figure 46. ^{13}C NMR of **3N**

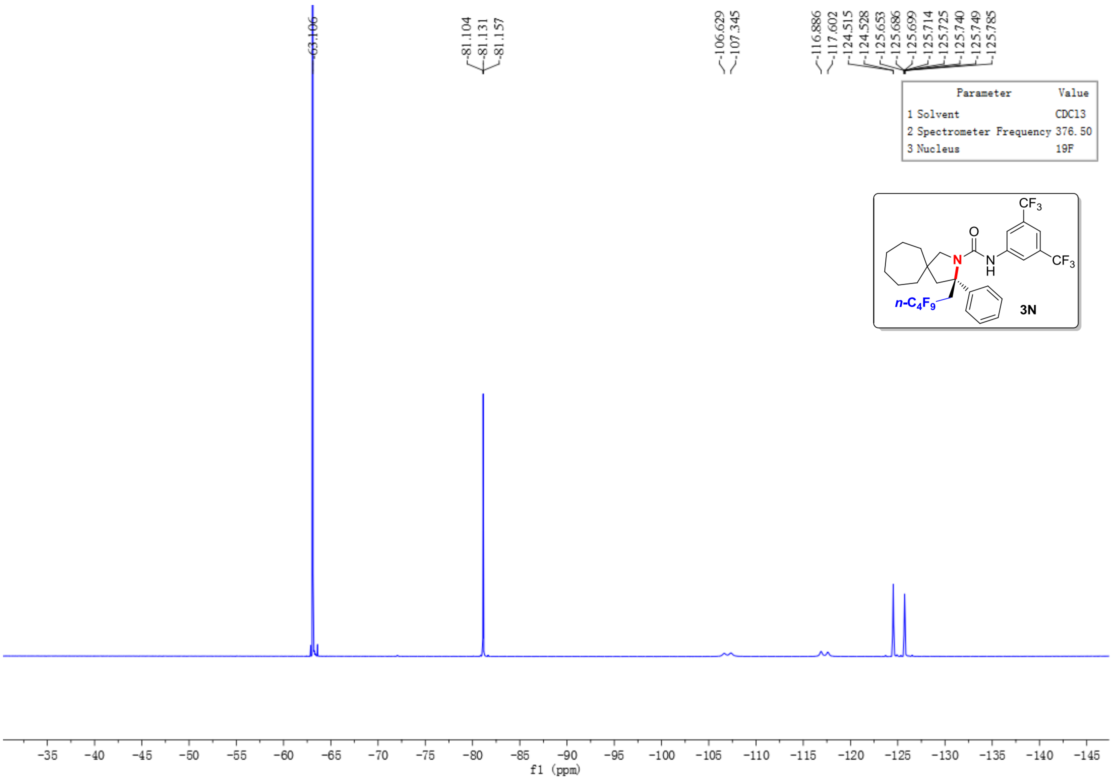

Supplementary Figure 47. ^{19}F NMR of **3N**

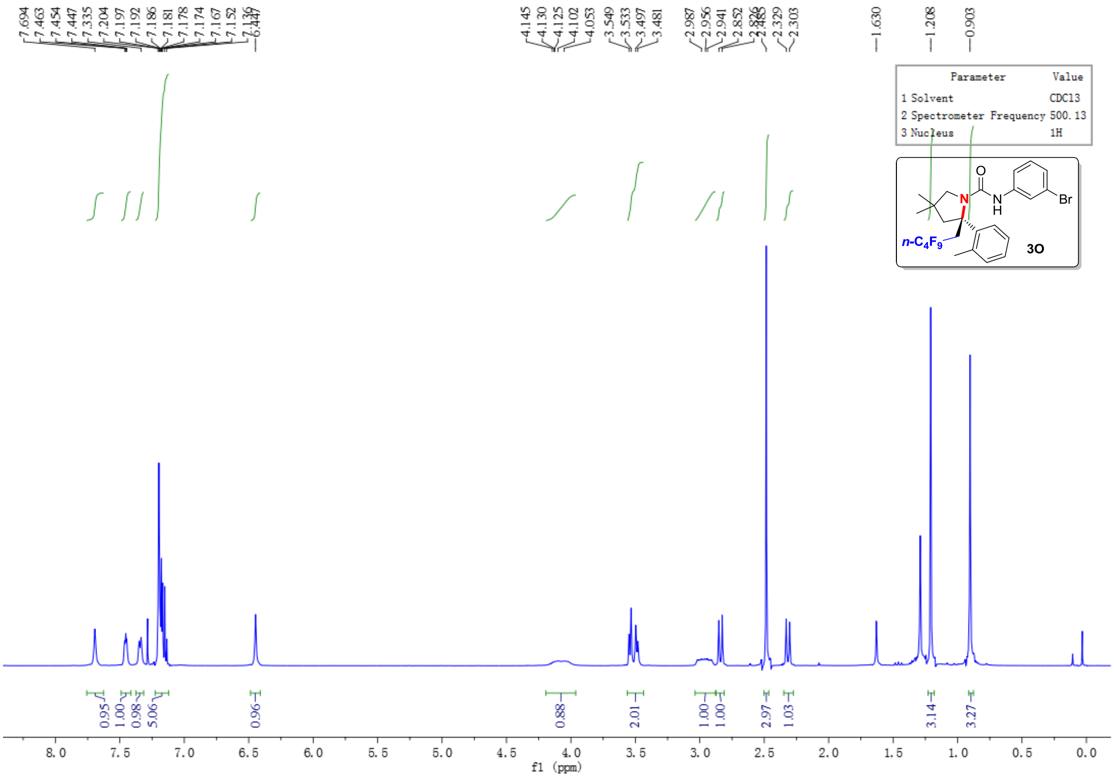

Supplementary Figure 48. ^{1}H NMR of **3O**

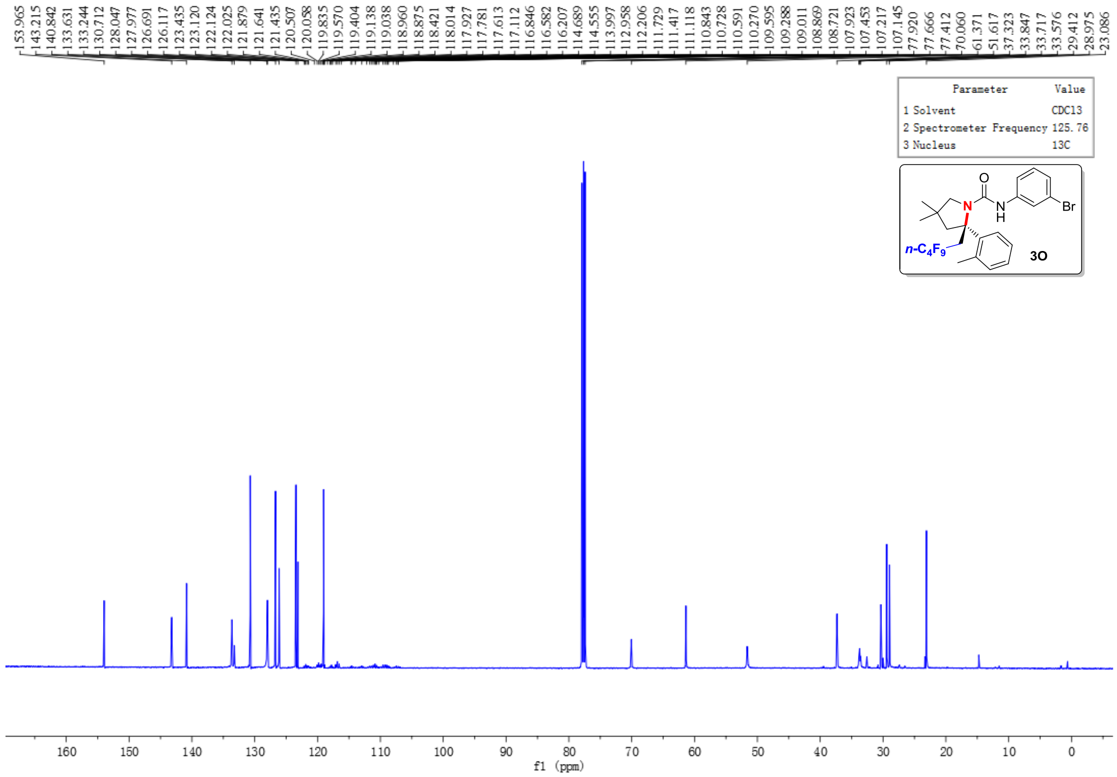

Supplementary Figure 49. ^{13}C NMR of **3O**

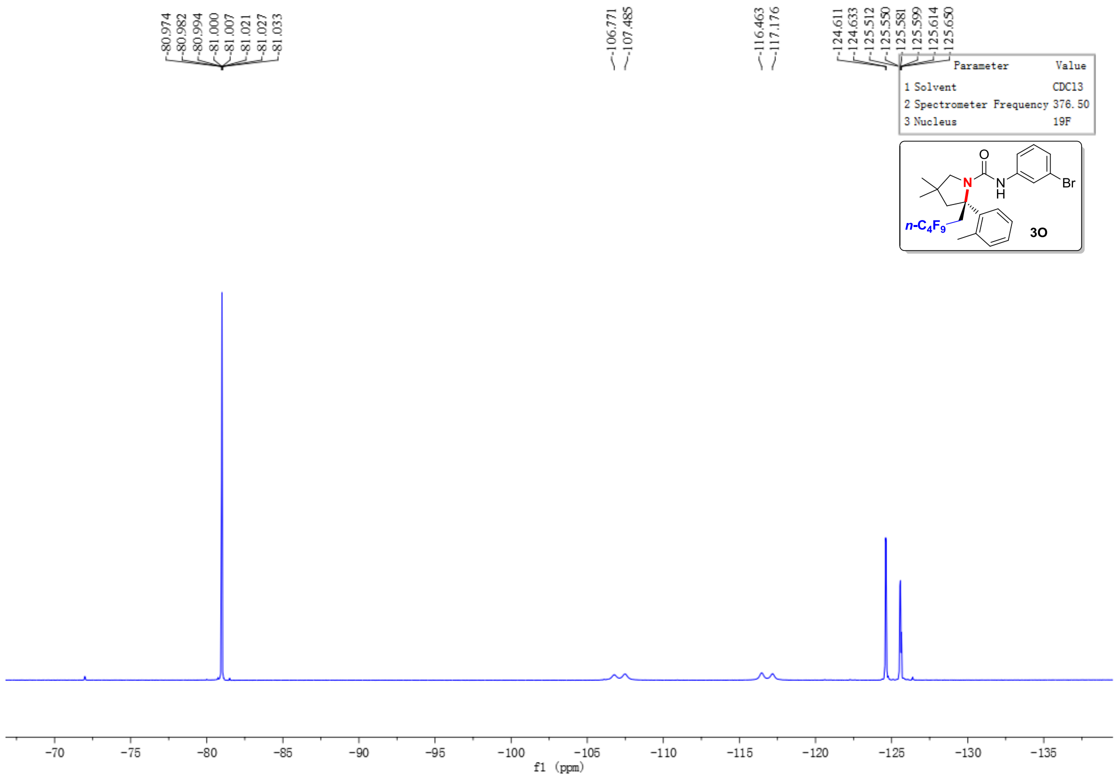

Supplementary Figure 50. ^{19}F NMR of **3O**

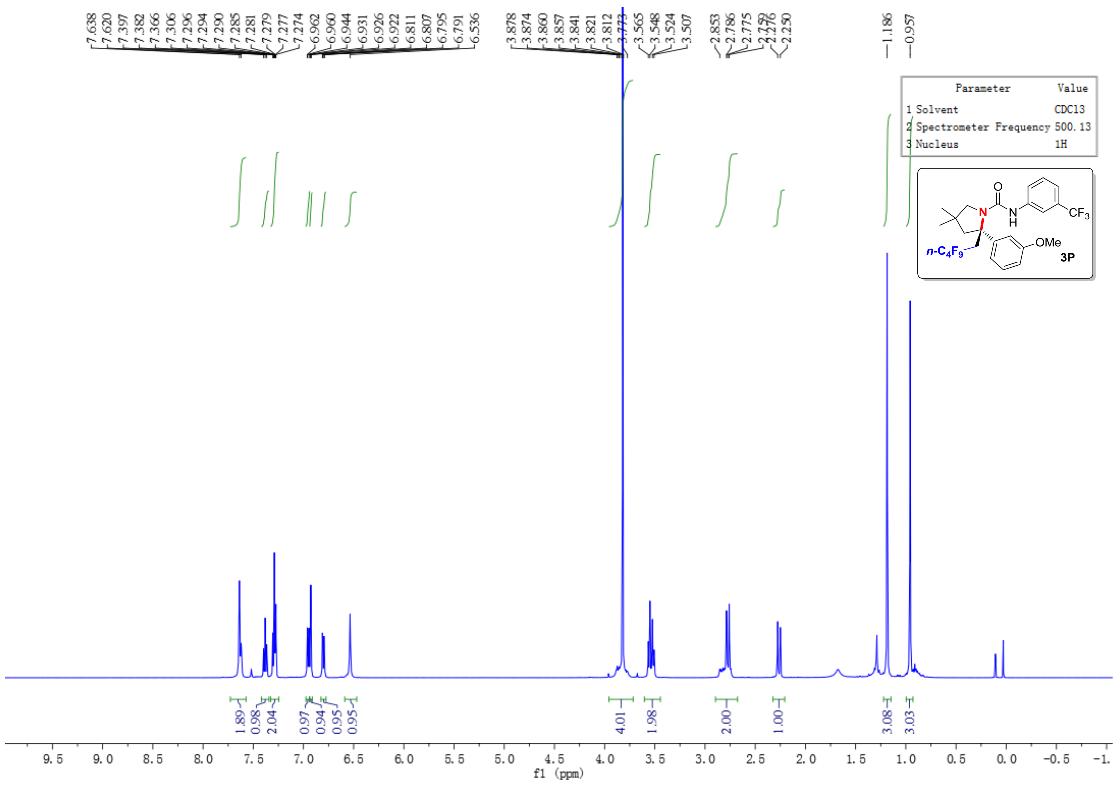

Supplementary Figure 51. ^{1}H NMR of **3P**

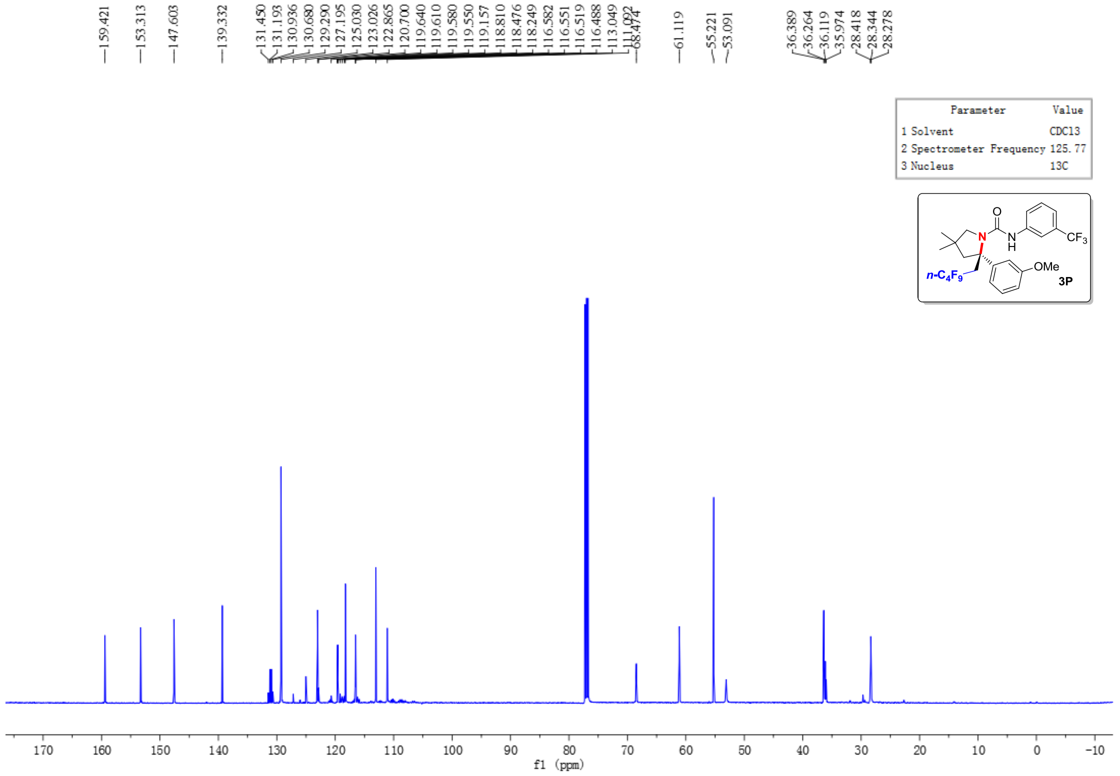

Supplementary Figure 52. ^{13}C NMR of **3P**

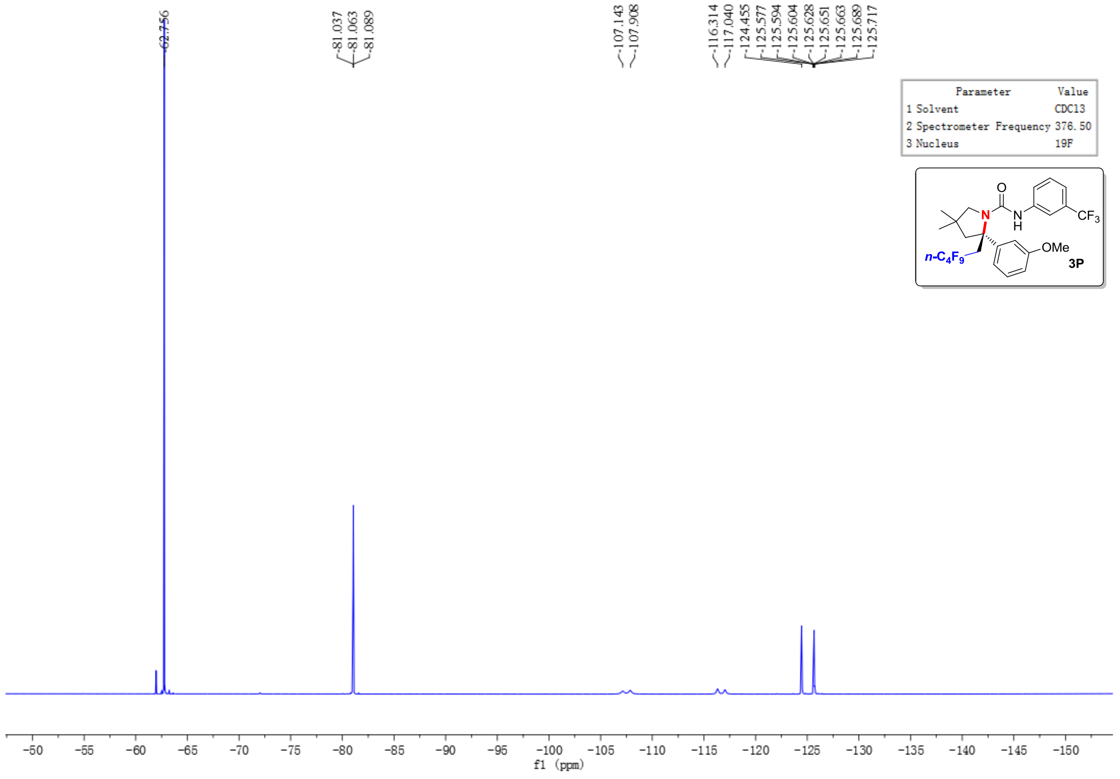

Supplementary Figure 53. ^{19}F NMR of **3P**

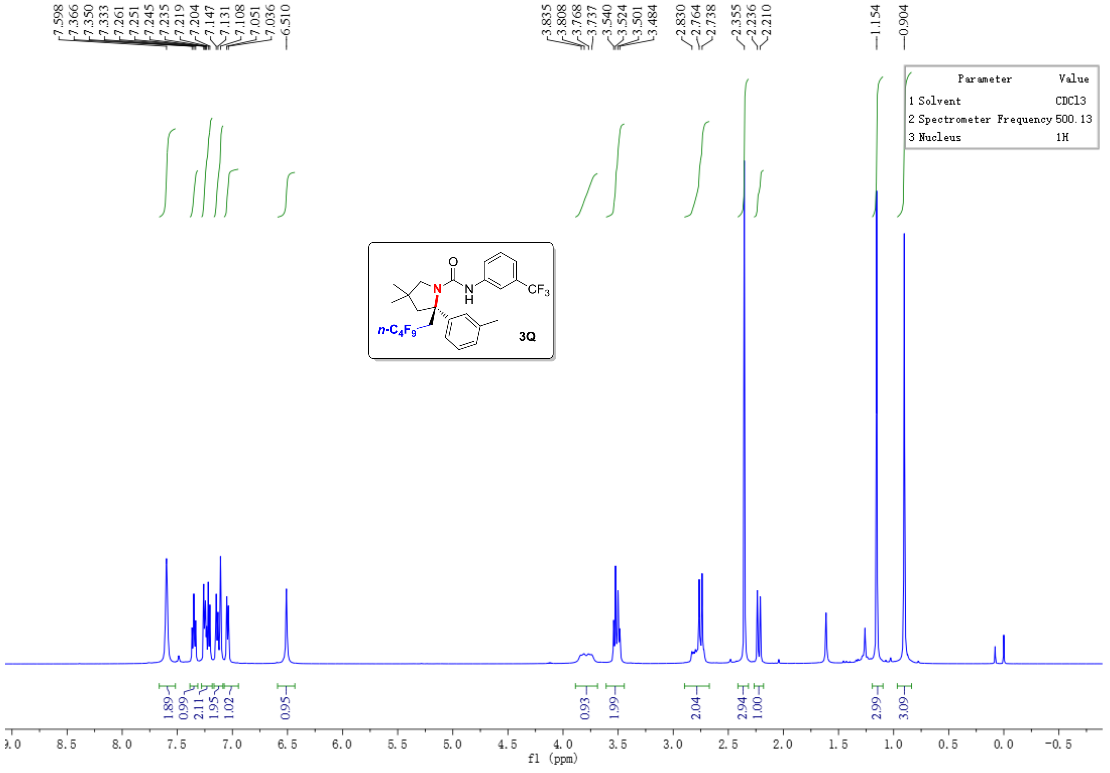

Supplementary Figure 54. ^{1}H NMR of **3Q**

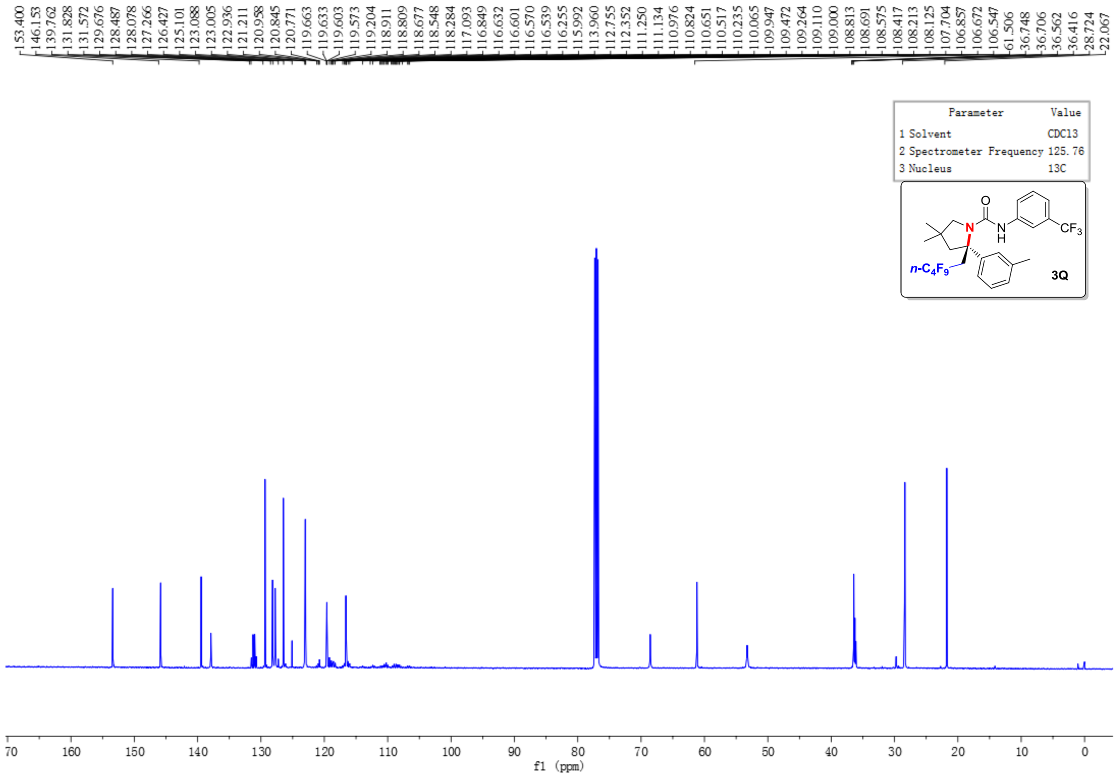

Supplementary Figure 55. ^{13}C NMR of **3Q**

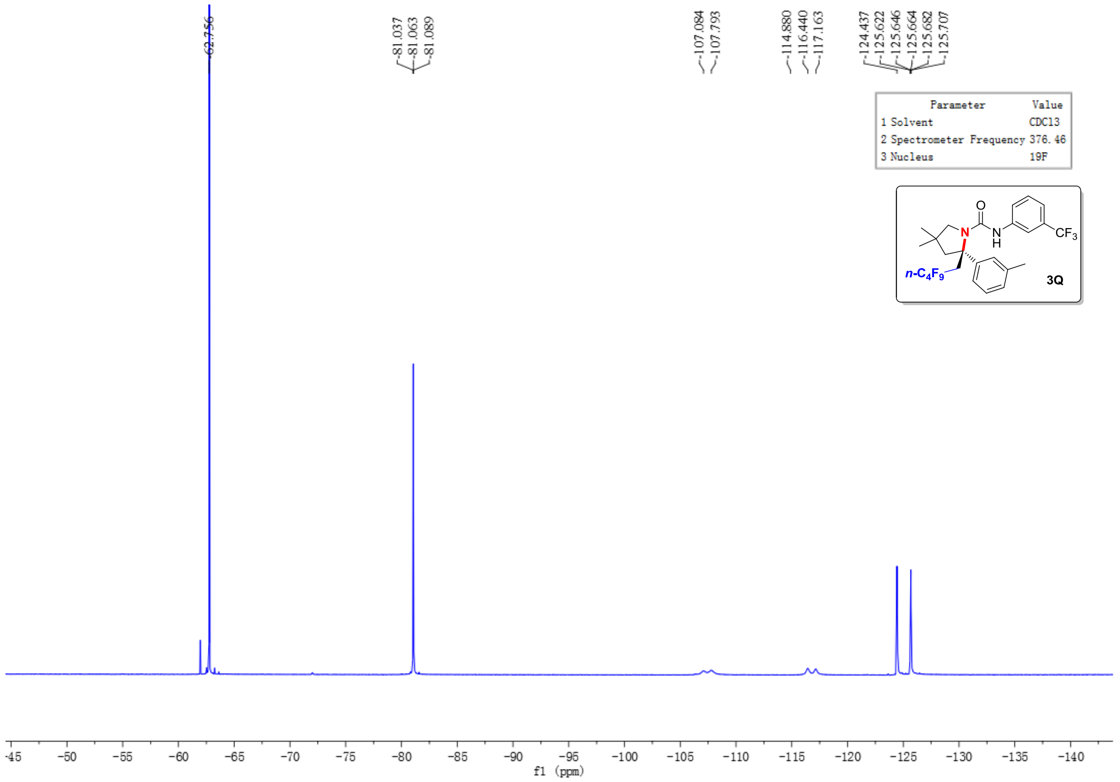

Supplementary Figure 56. ^{19}F NMR of **3Q**

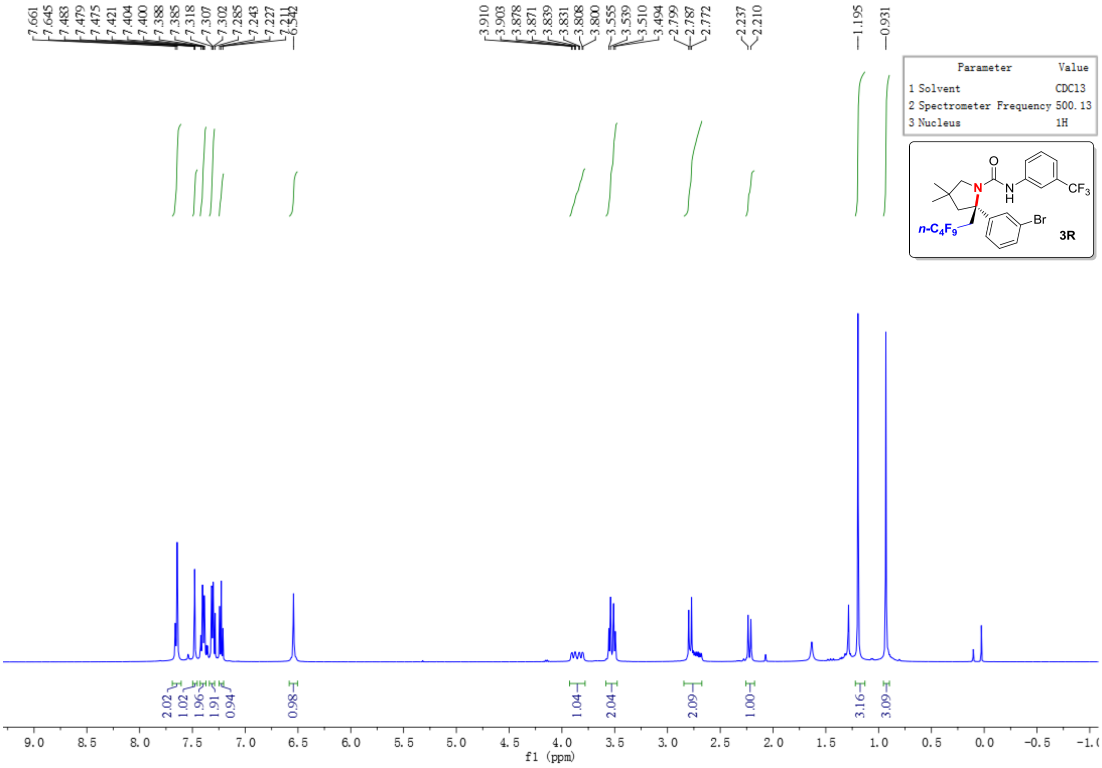

Supplementary Figure 57. ^{1}H NMR of **3R**

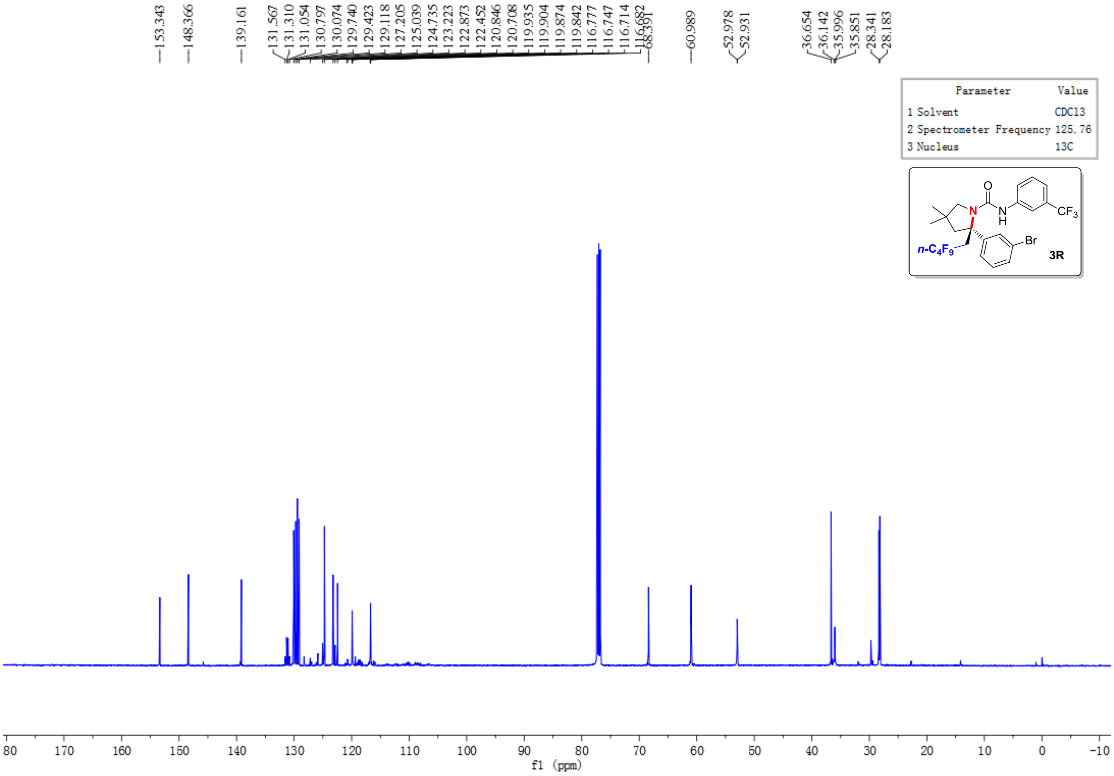

Supplementary Figure 58. ^{13}C NMR of **3R**

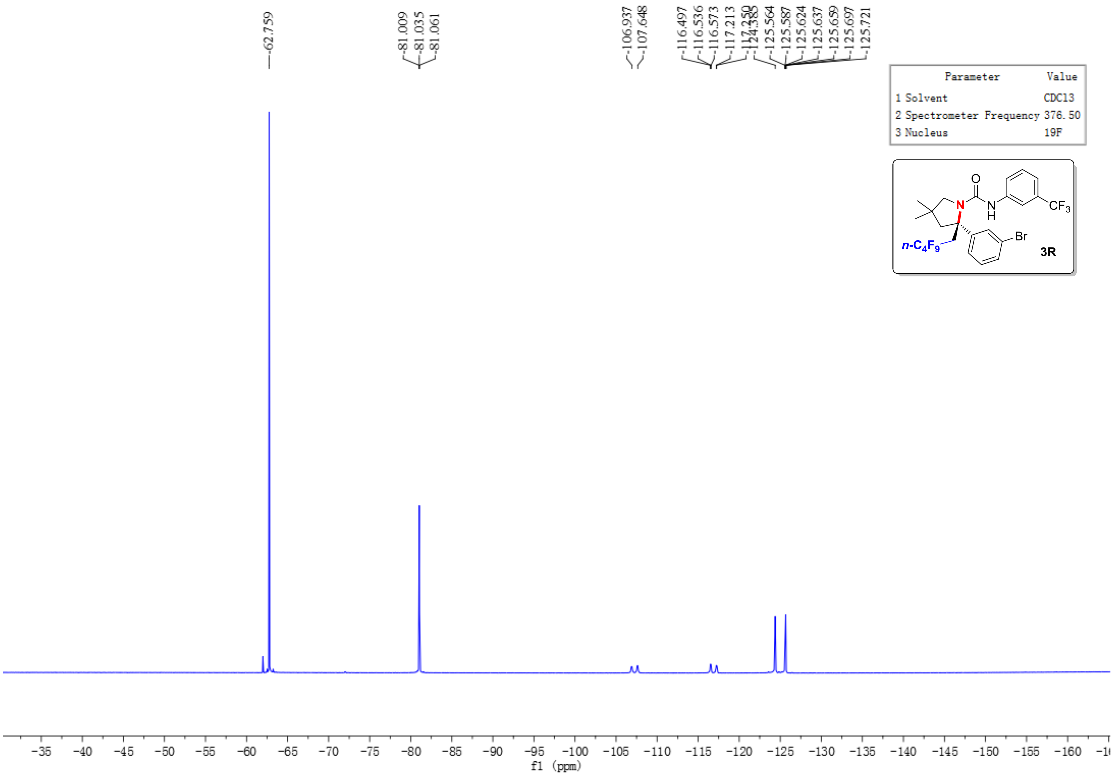

Supplementary Figure 59. ^{19}F NMR of **3R**

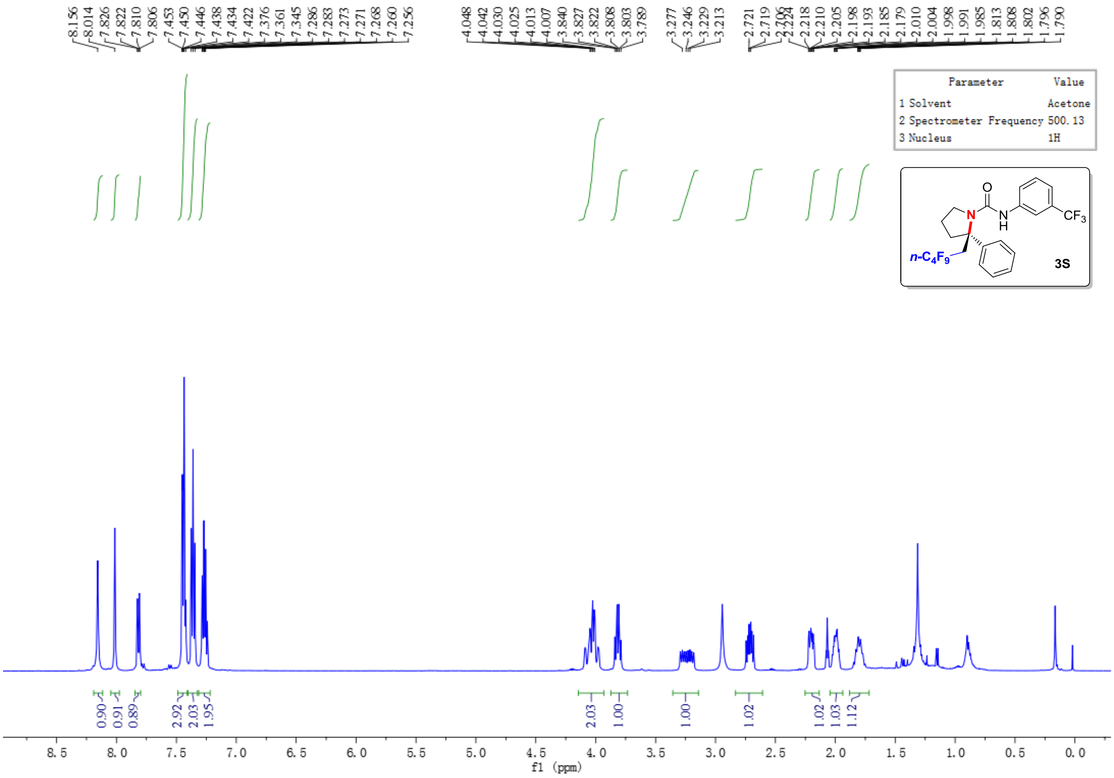

Supplementary Figure 60. ^{1}H NMR of **3S**

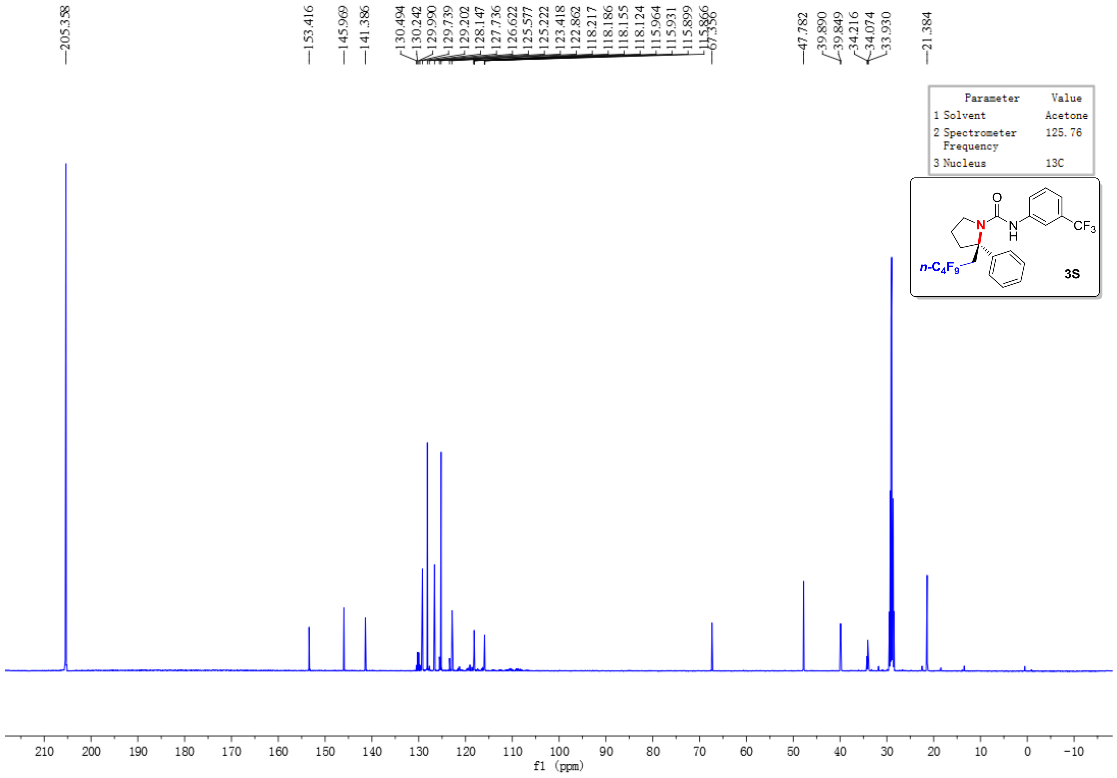

Supplementary Figure 61. ^{13}C NMR of **3S**

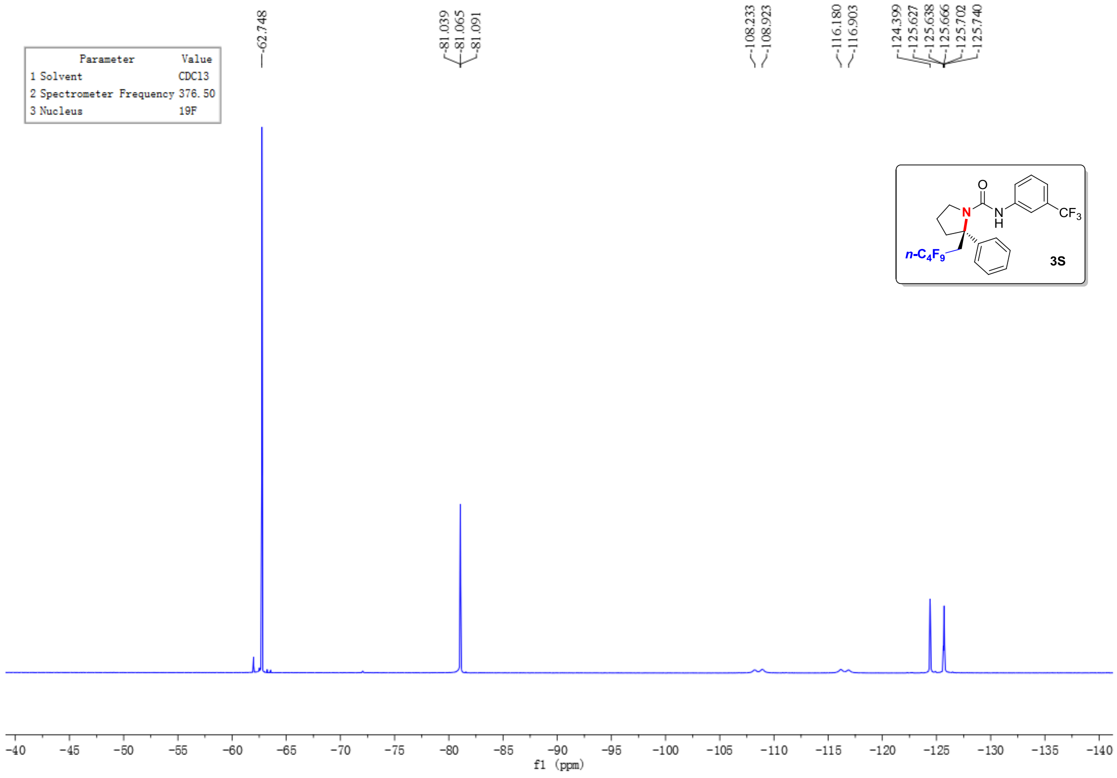

Supplementary Figure 62. ^{19}F NMR of **3S**

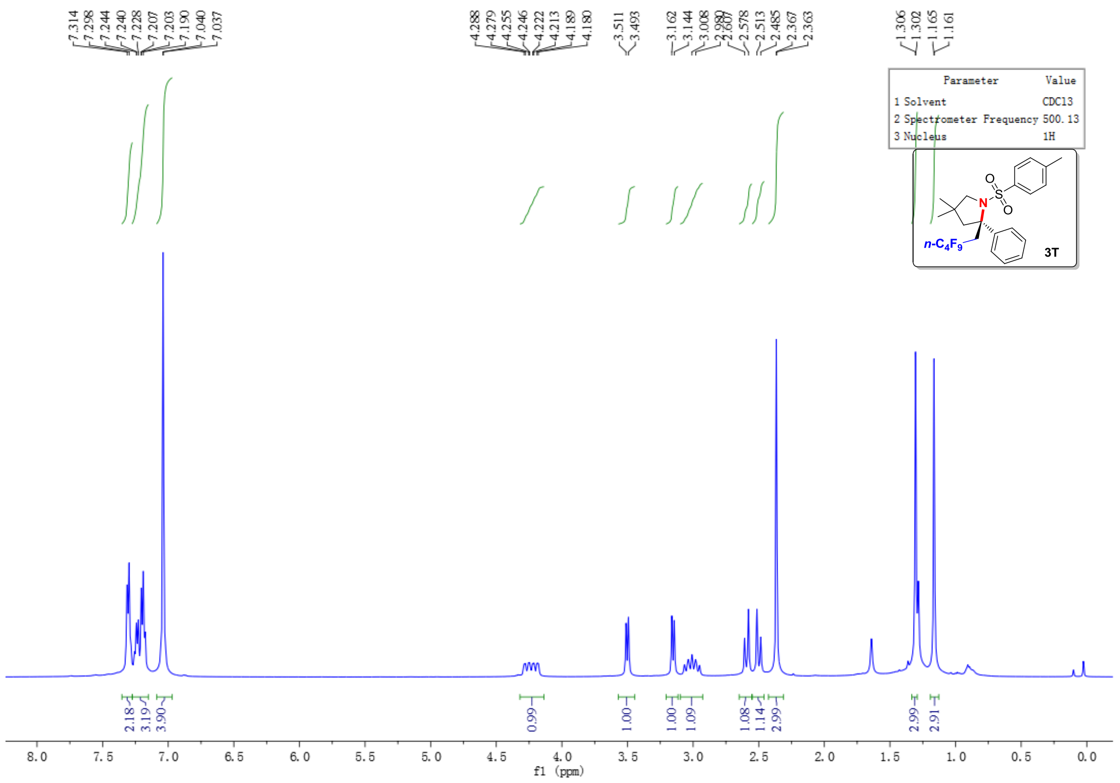

Supplementary Figure 63. ^{1}H NMR of **3T**

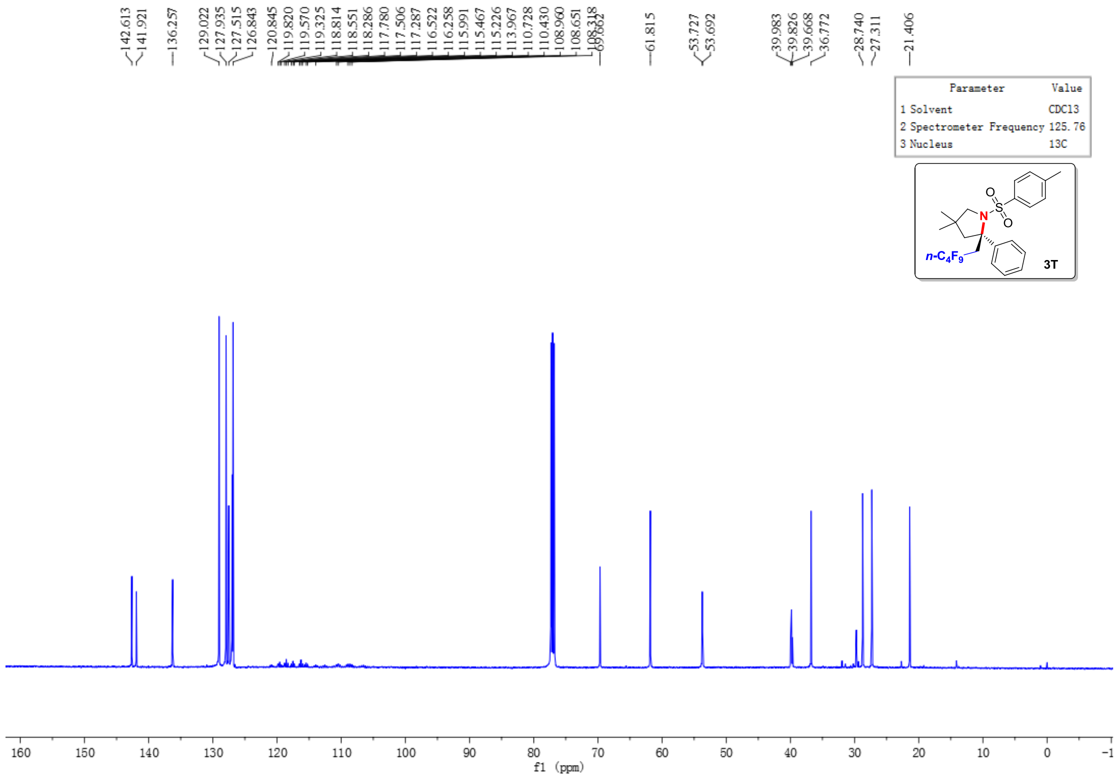

Supplementary Figure 64. ^{13}C NMR of **3T**

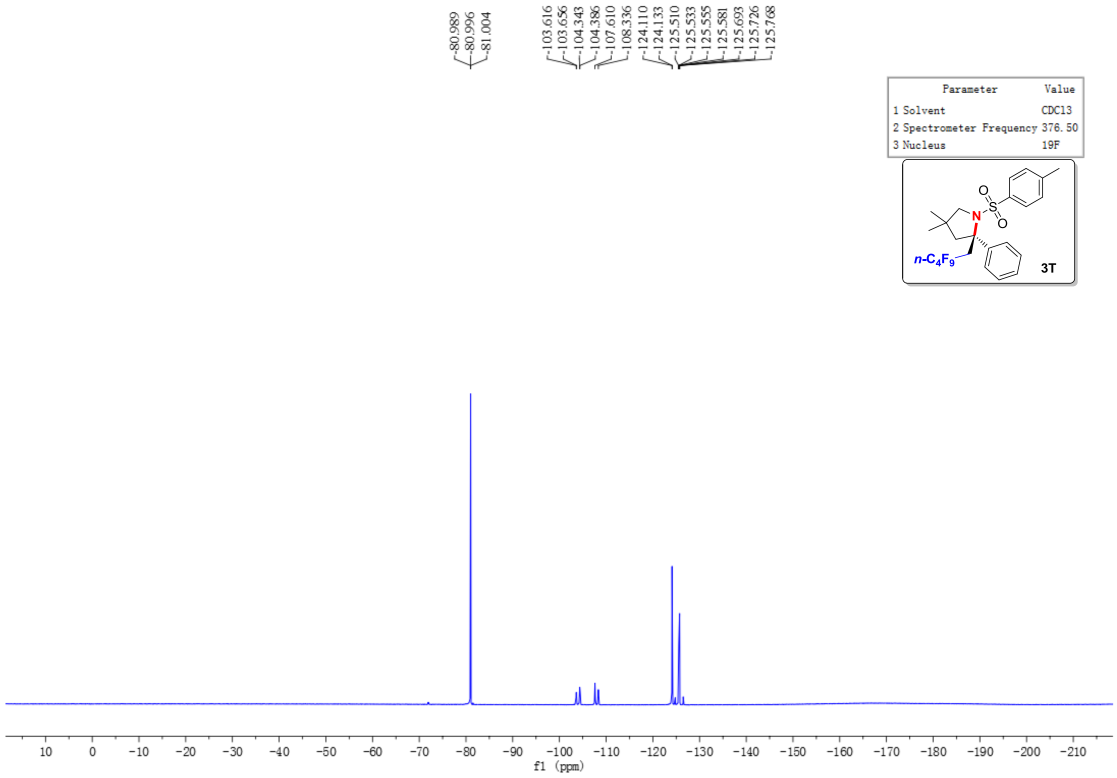

Supplementary Figure 65. ^{19}F NMR of **3T**

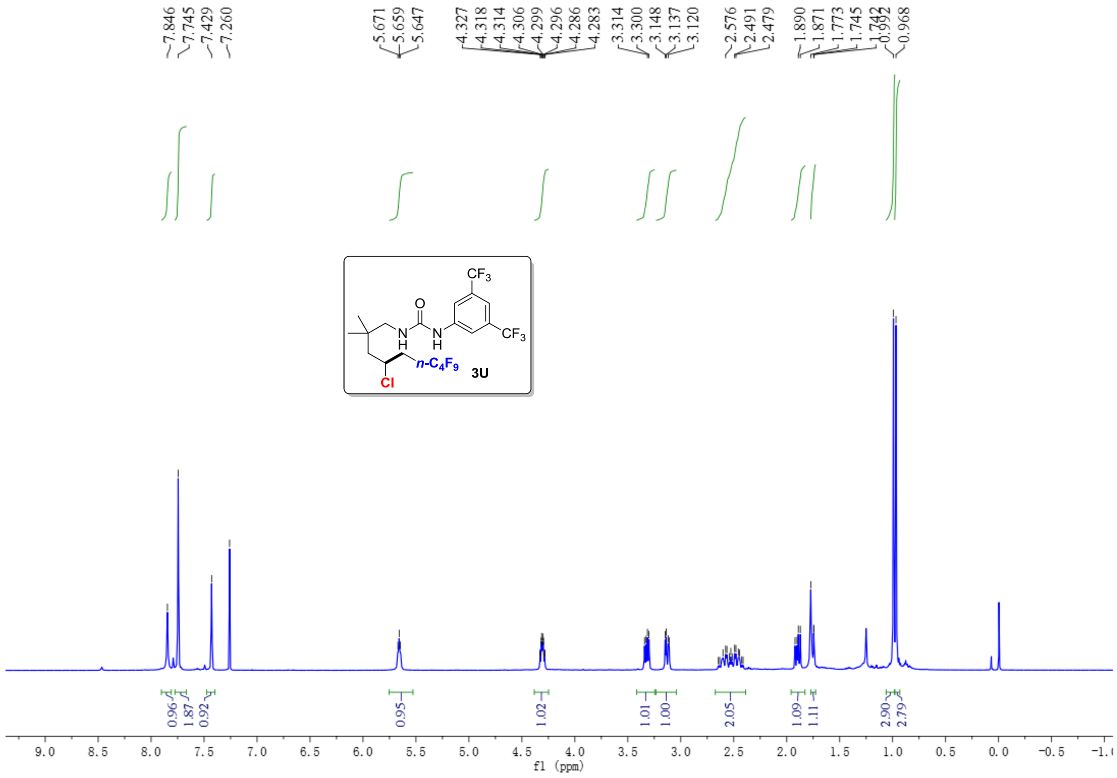

Supplementary Figure 66. ^{1}H NMR of **3U**

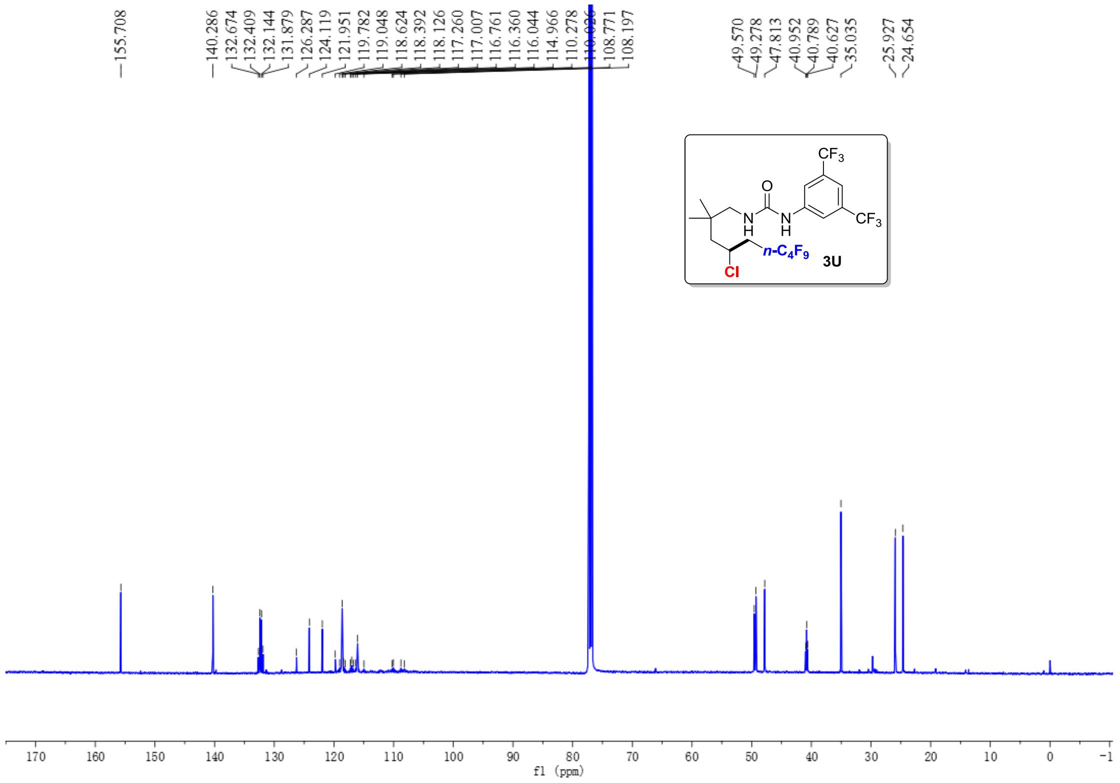

Supplementary Figure 67. ^{13}C NMR of **3U**

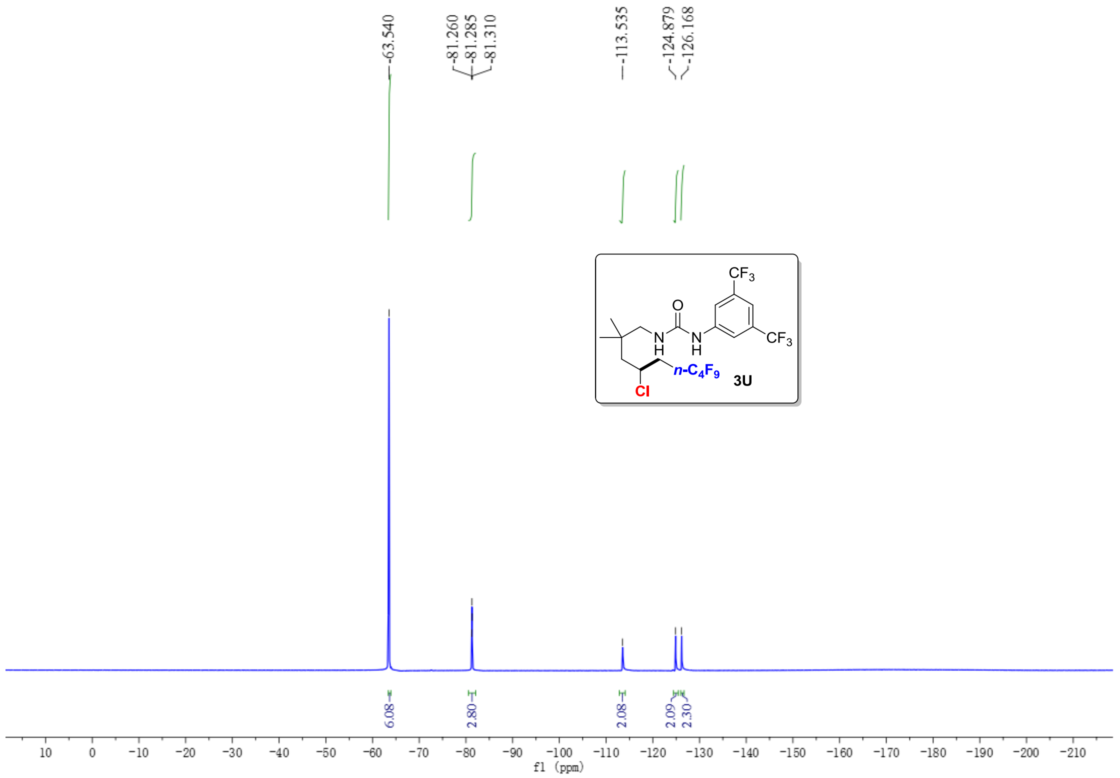

Supplementary Figure 68. ^{19}F NMR of **3U**

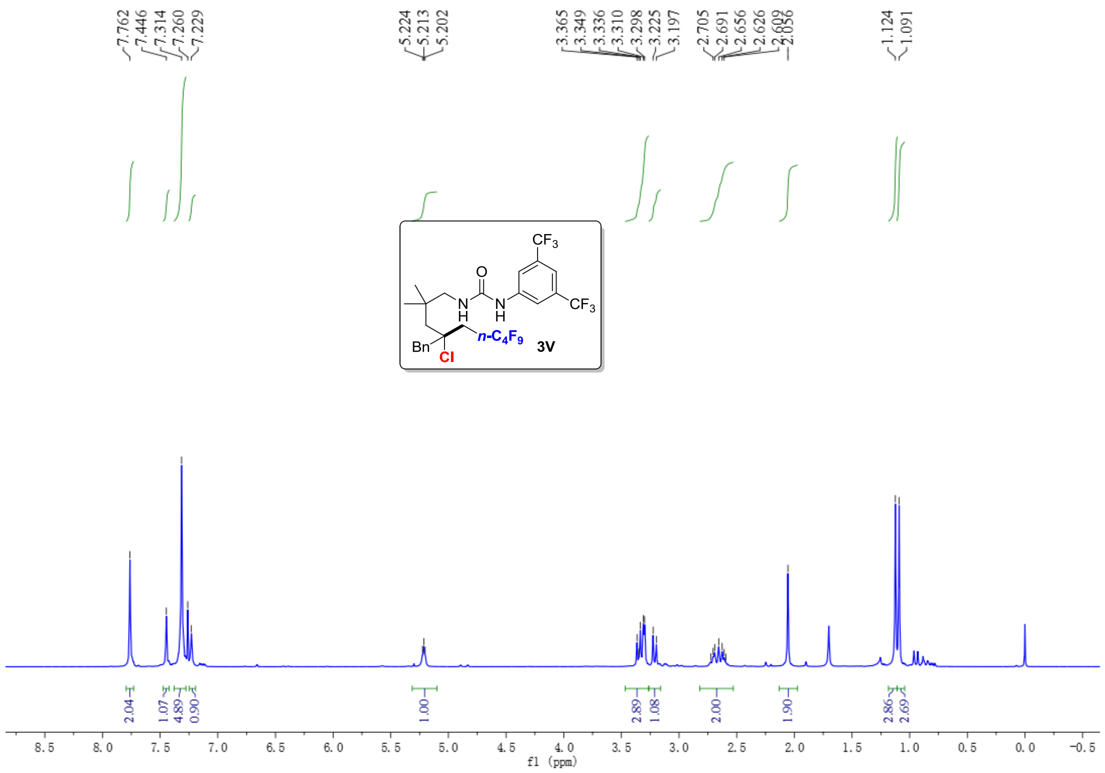

Supplementary Figure 69. ^{1}H NMR of **3V**

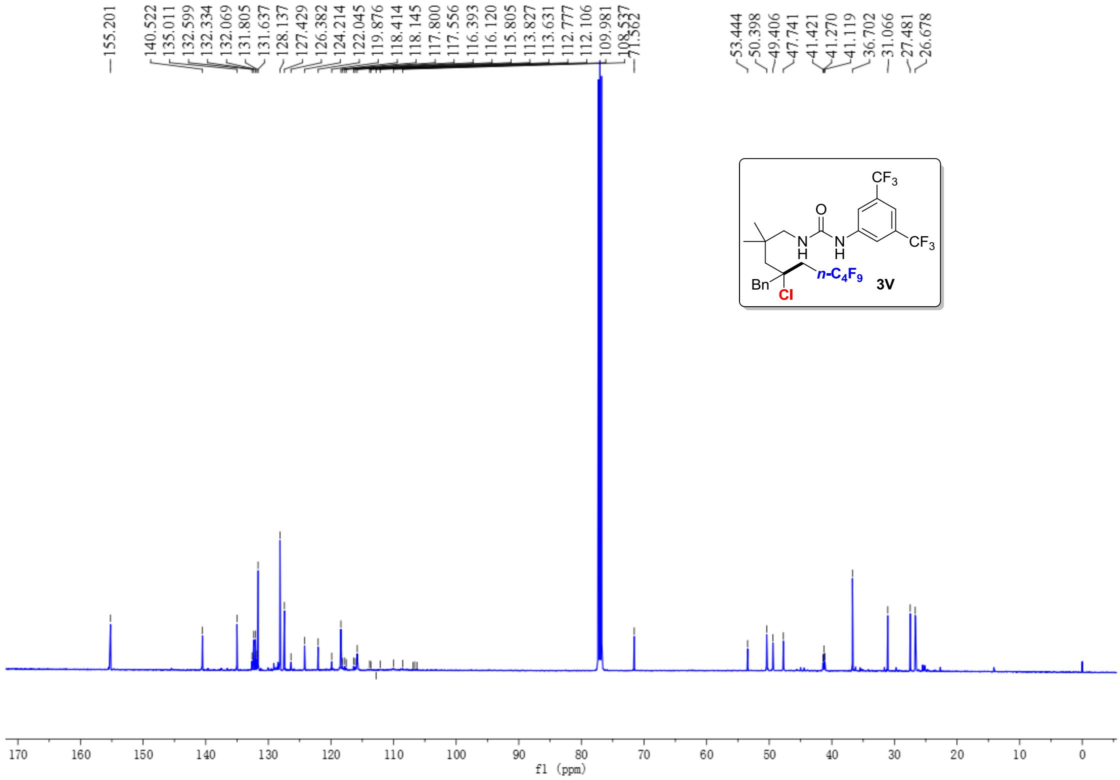

Supplementary Figure 70. ^{13}C NMR of **3V**

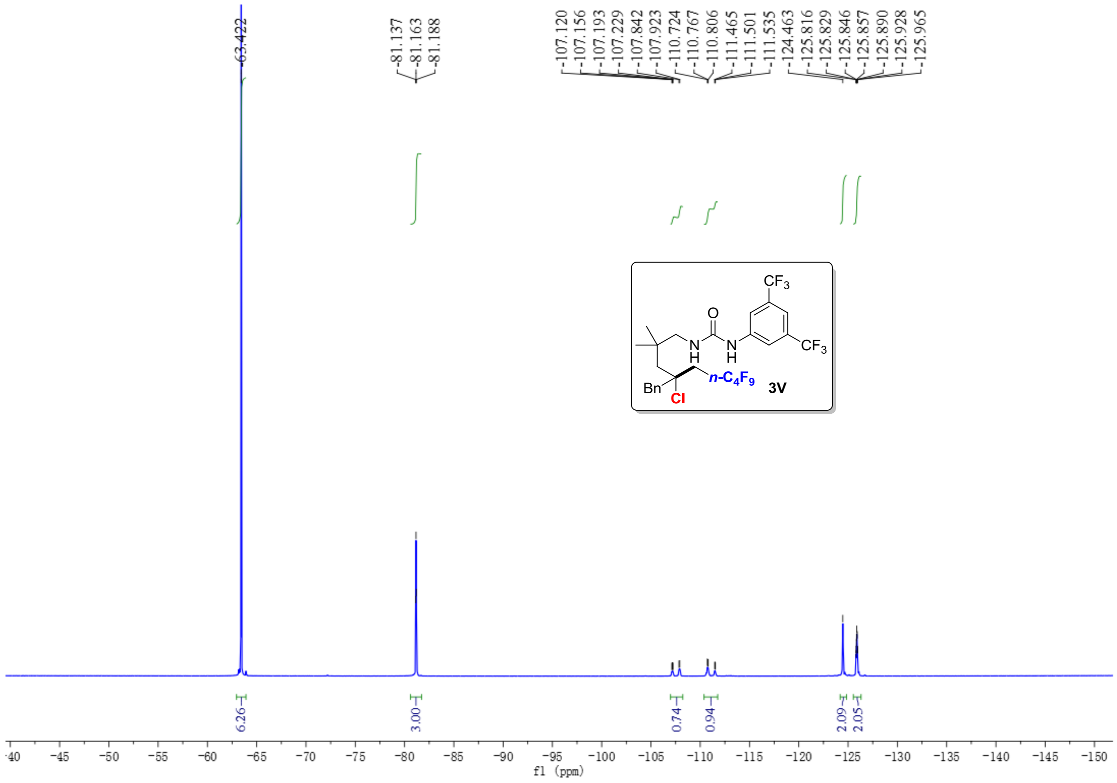

Supplementary Figure 71. ^{19}F NMR of **3V**

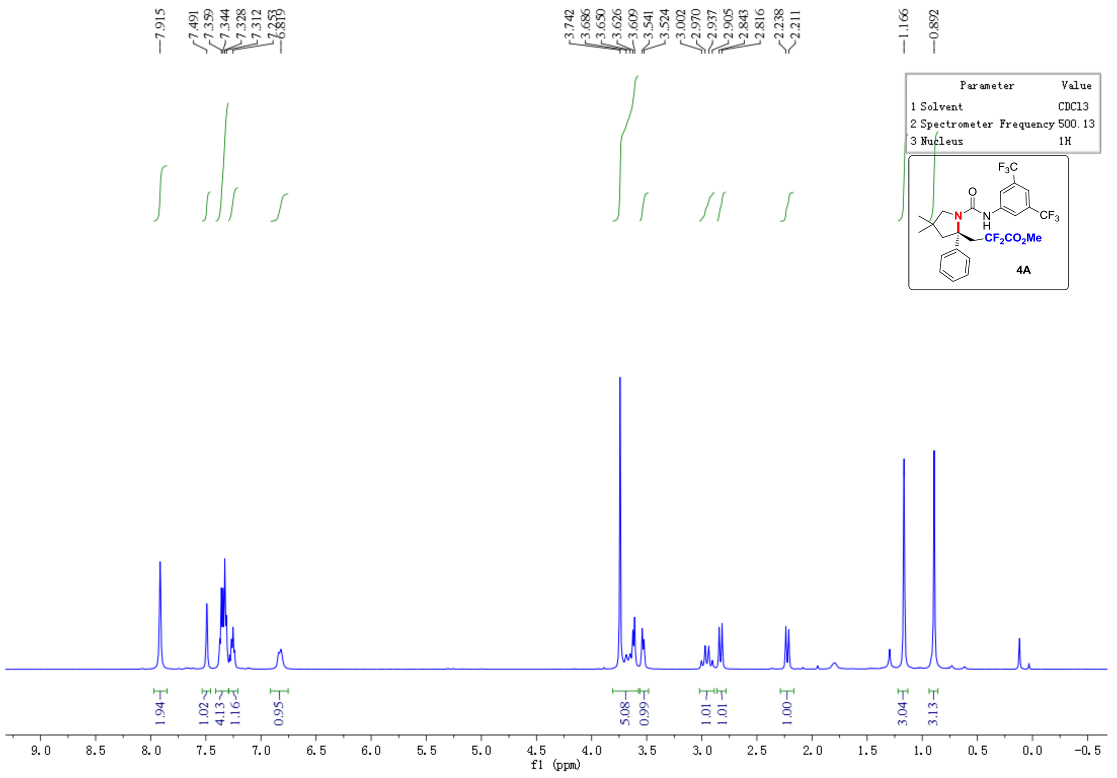

Supplementary Figure 72. ^{1}H NMR of **4A**

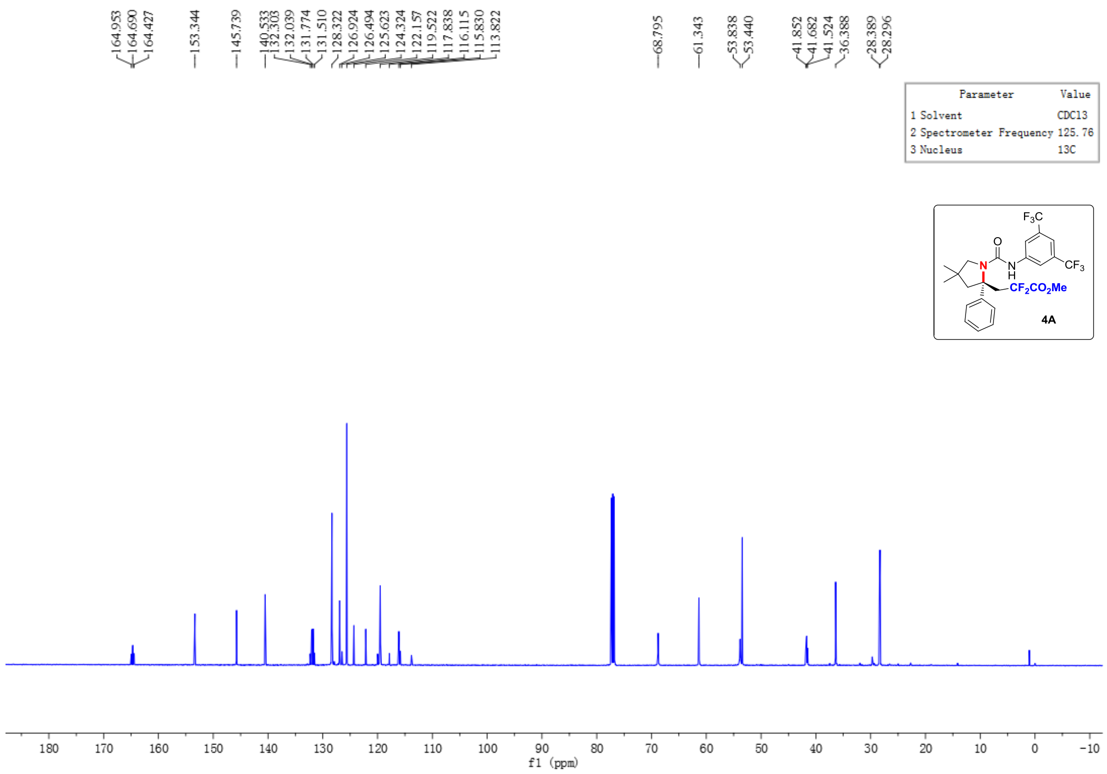

Supplementary Figure 73. ^{13}C NMR of **4A**

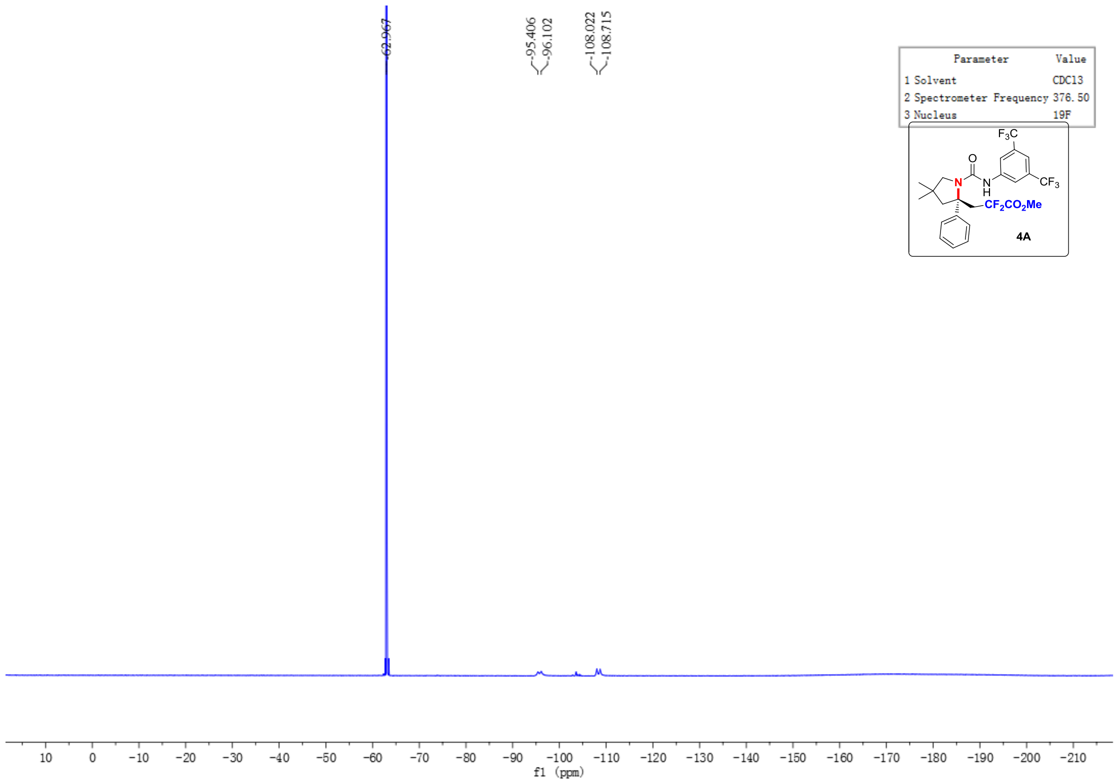

Supplementary Figure 74. ^{19}F NMR of **4A**

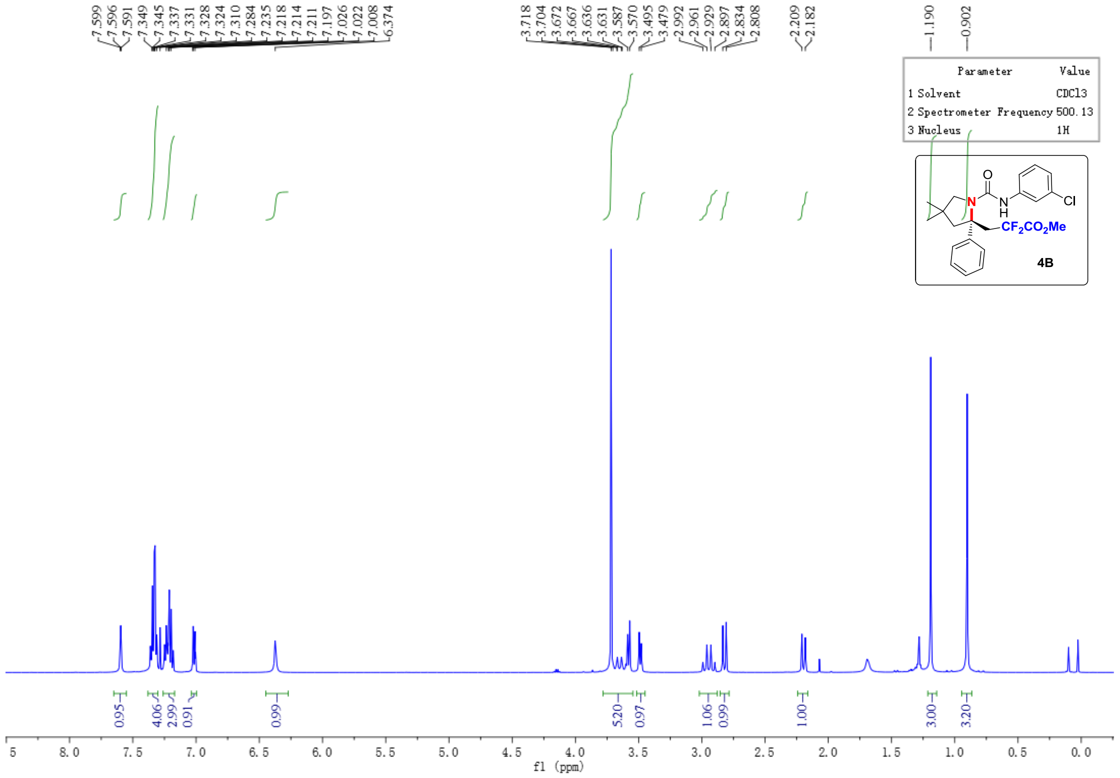

Supplementary Figure 75. ^{1}H NMR of **4B**

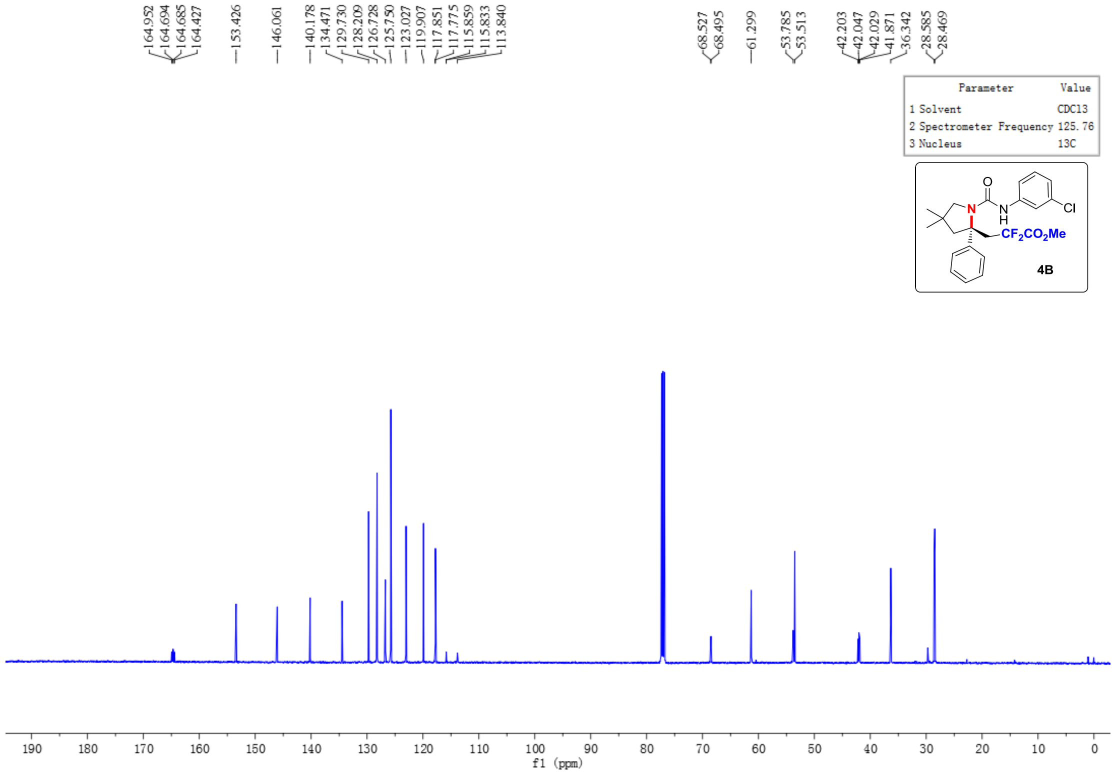

Supplementary Figure 76. ^{13}C NMR of **4B**

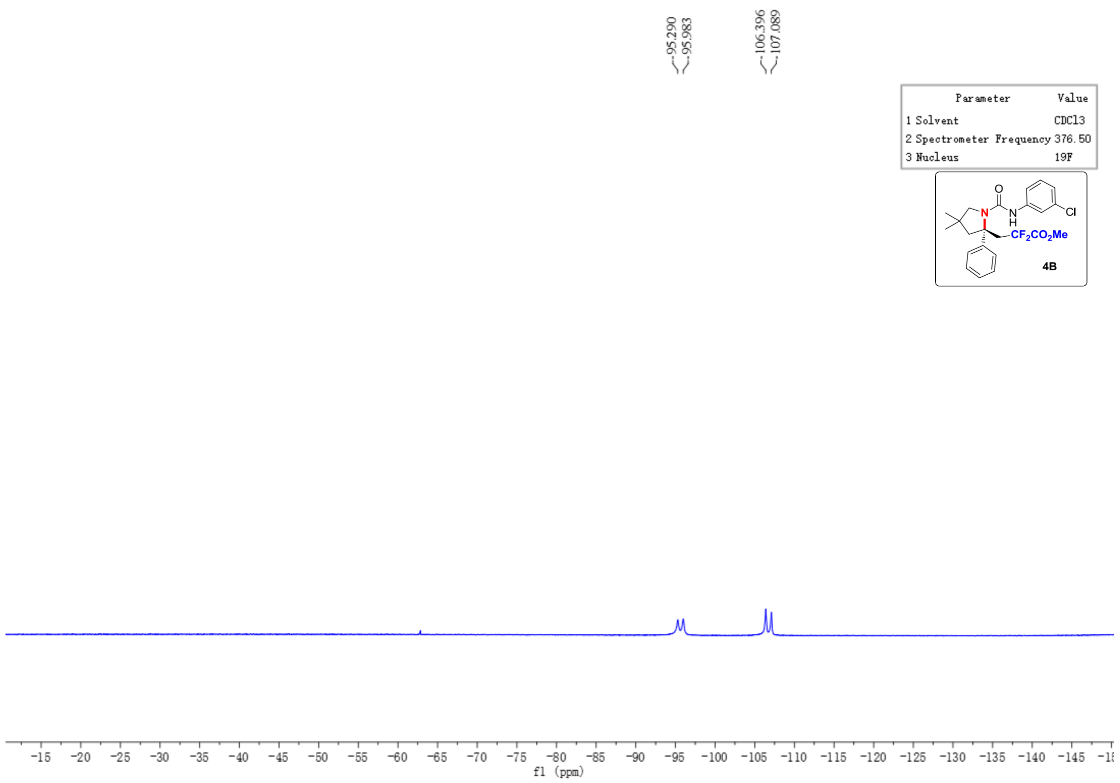

Supplementary Figure 77. ^{19}F NMR of **4B**

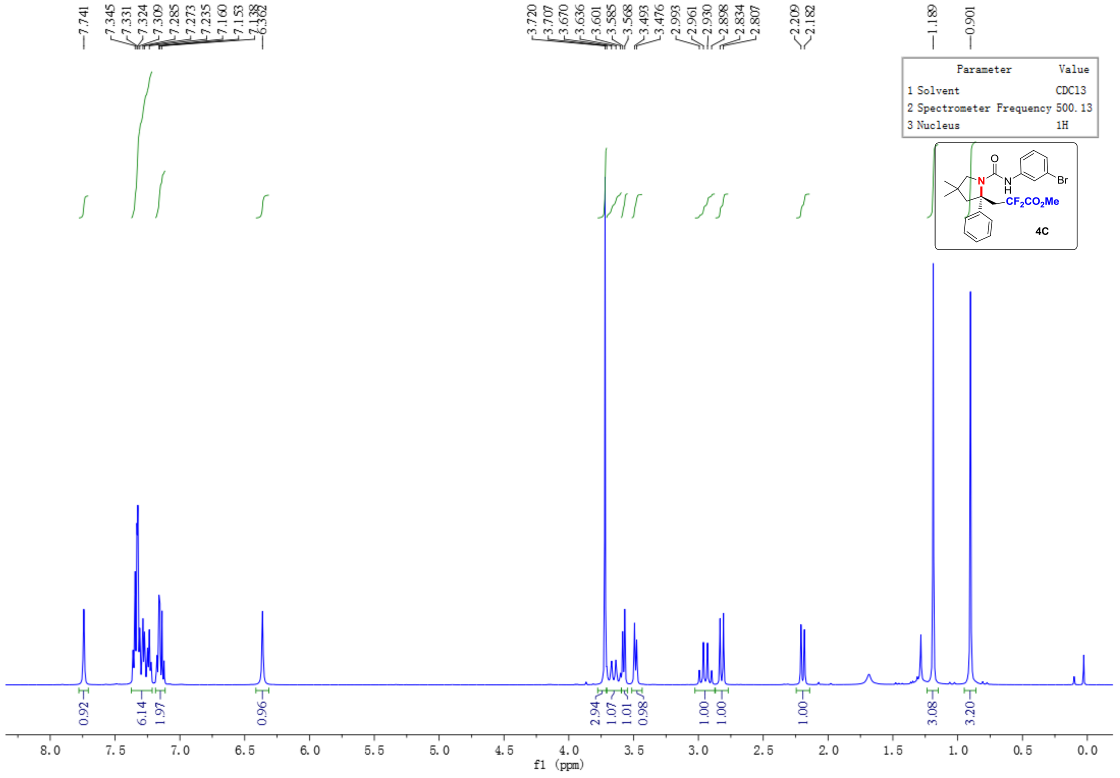

Supplementary Figure 78. ^{1}H NMR of **4C**

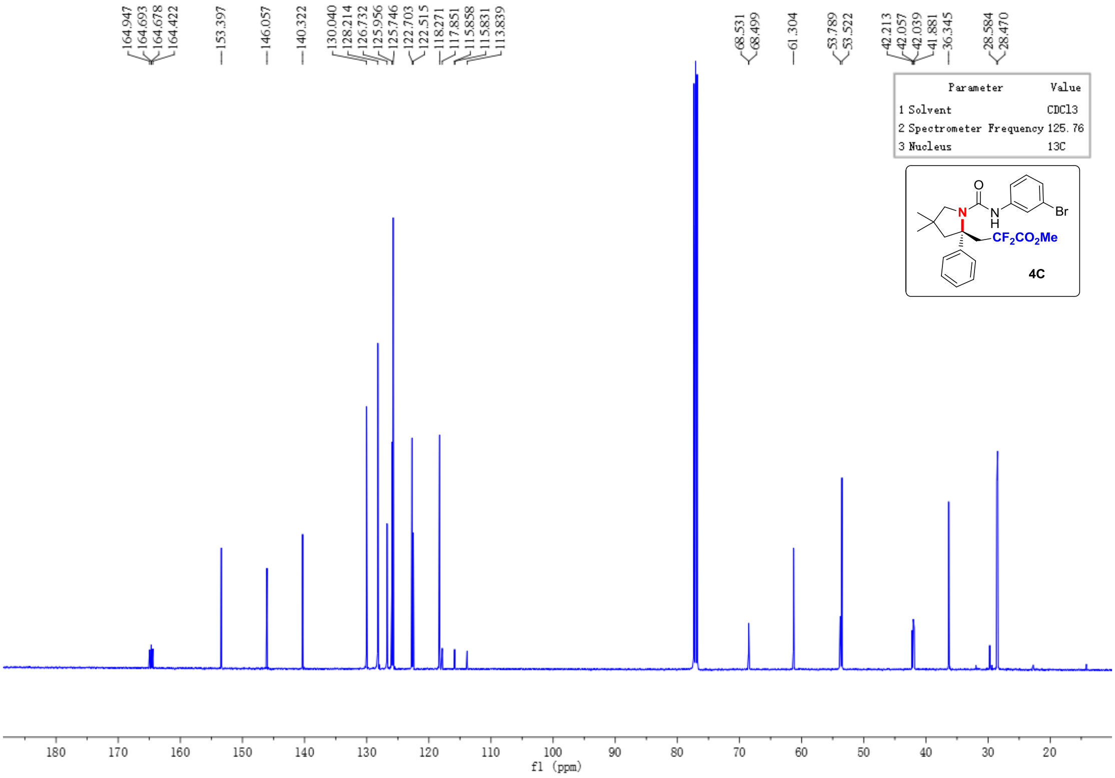

Supplementary Figure 79. ^{13}C NMR of **4C**

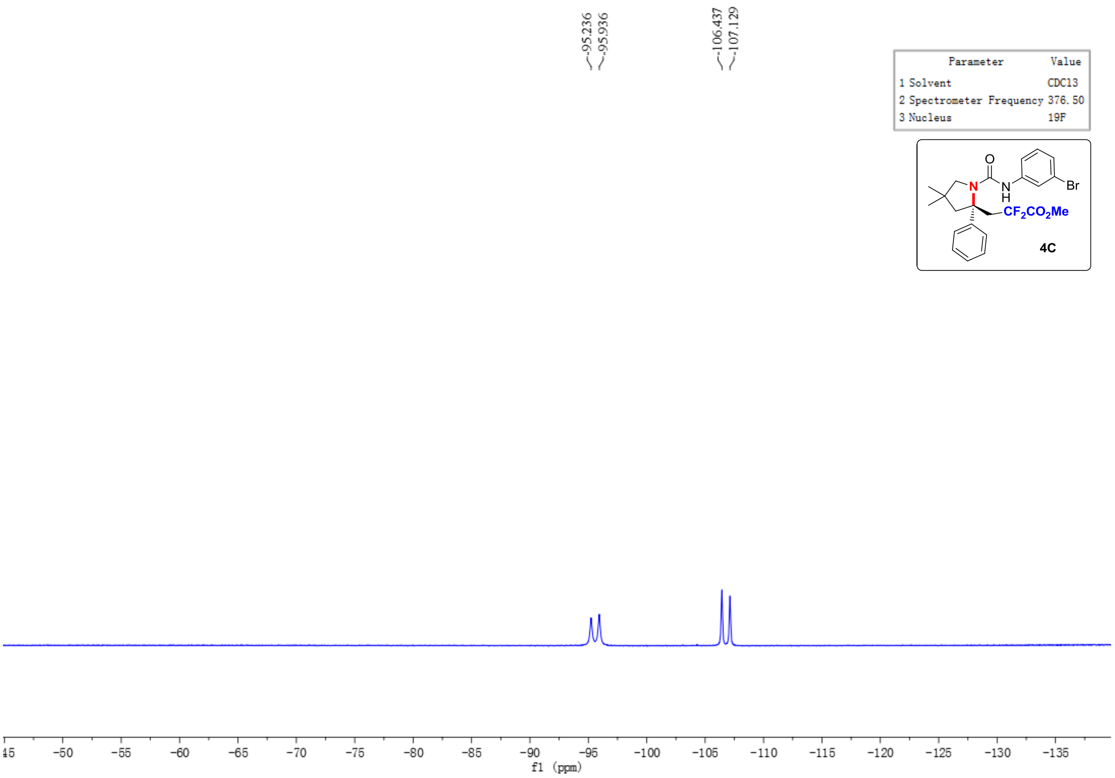

Supplementary Figure 80. ^{19}F NMR of **4C**

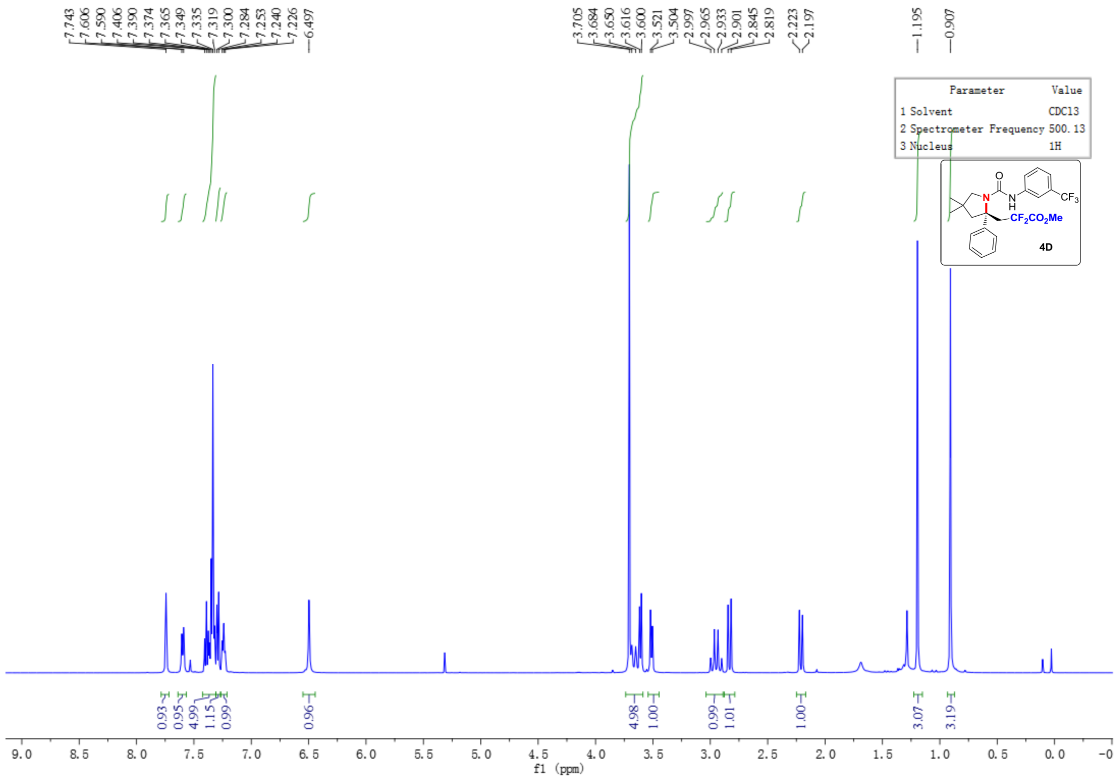

Supplementary Figure 81. ^{1}H NMR of **4D**

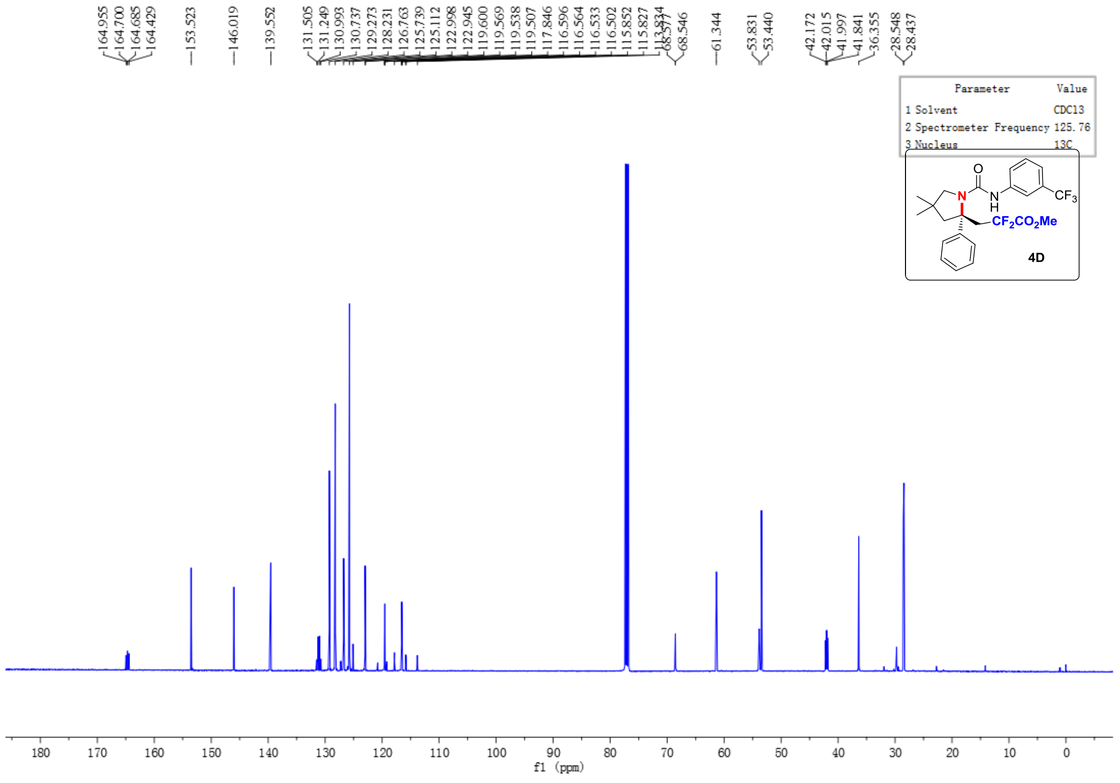

Supplementary Figure 82. ^{13}C NMR of **4D**

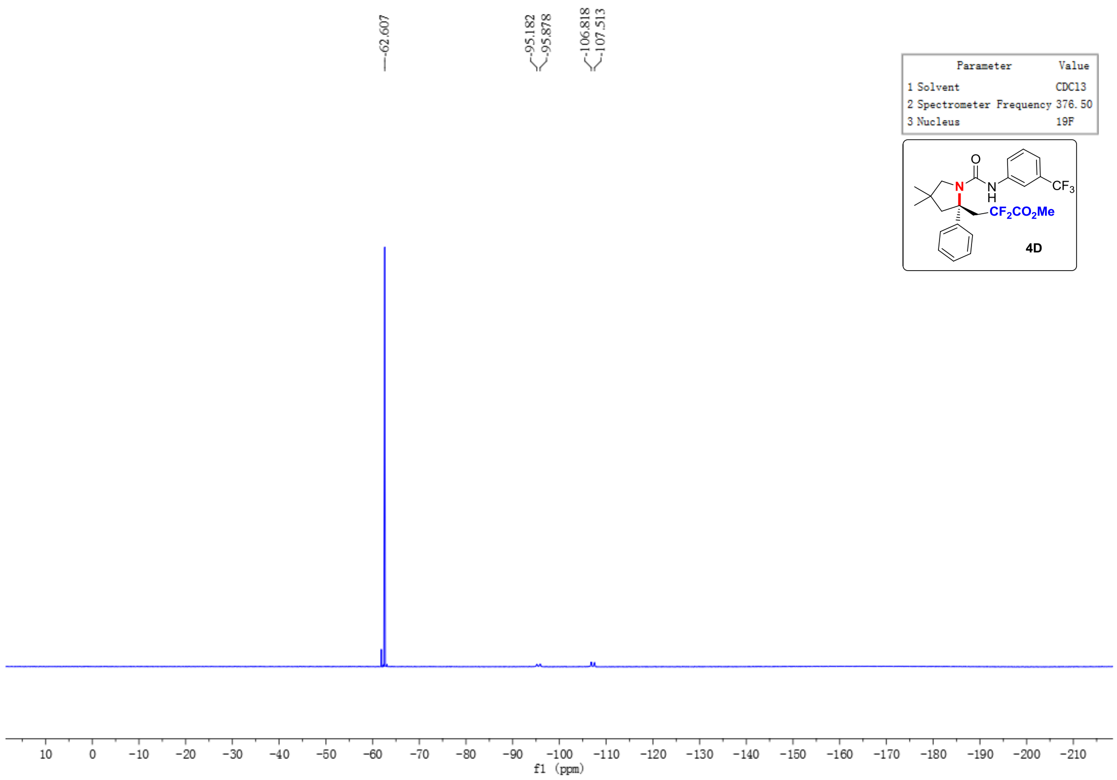

Supplementary Figure 83. ^{19}F NMR of **4D**

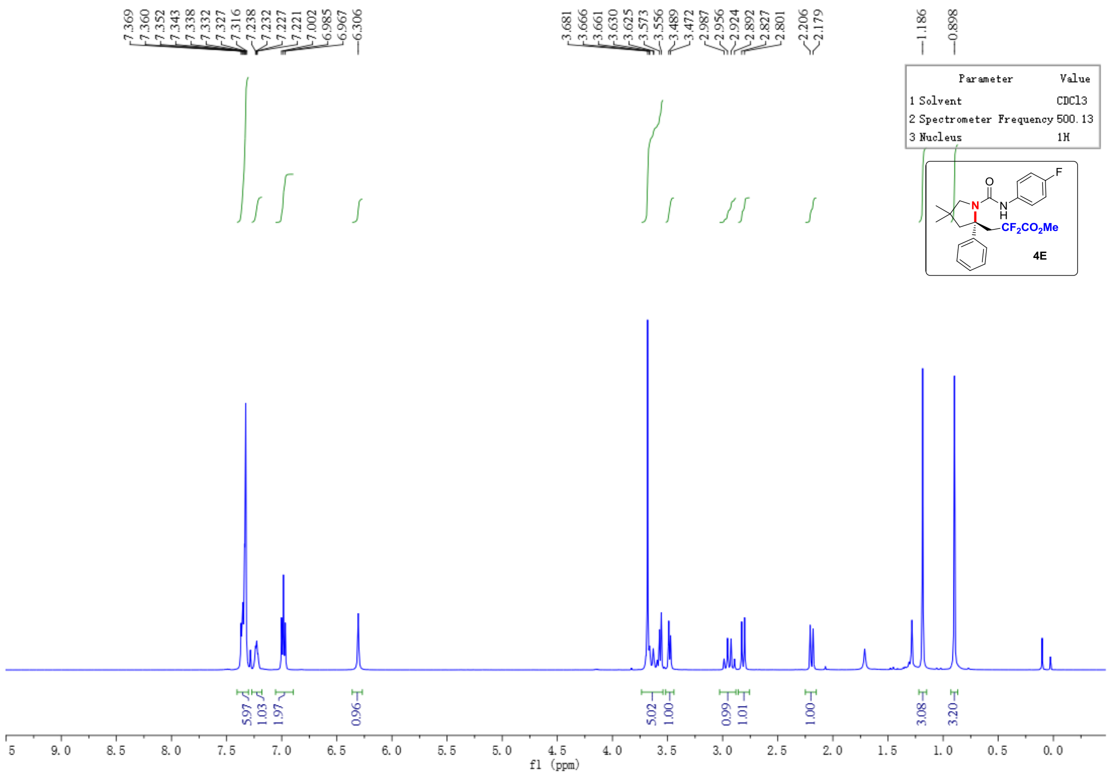

Supplementary Figure 84. ^{1}H NMR of **4E**

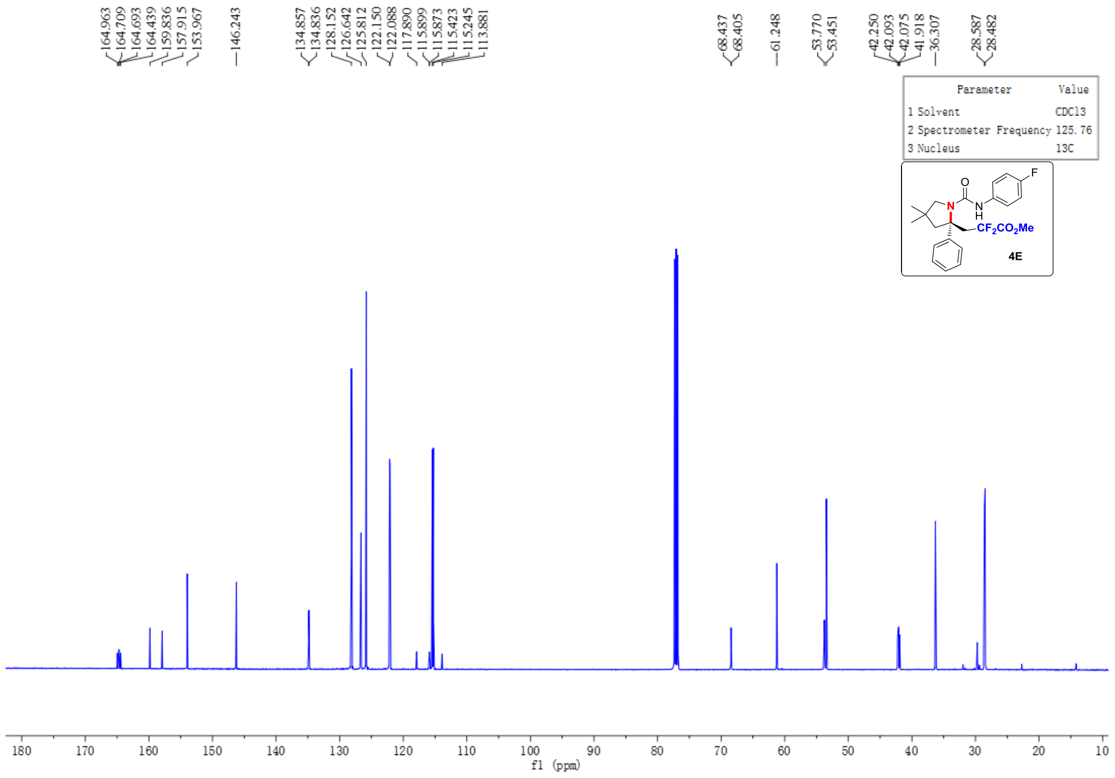

Supplementary Figure 85. ^{13}C NMR of **4E**

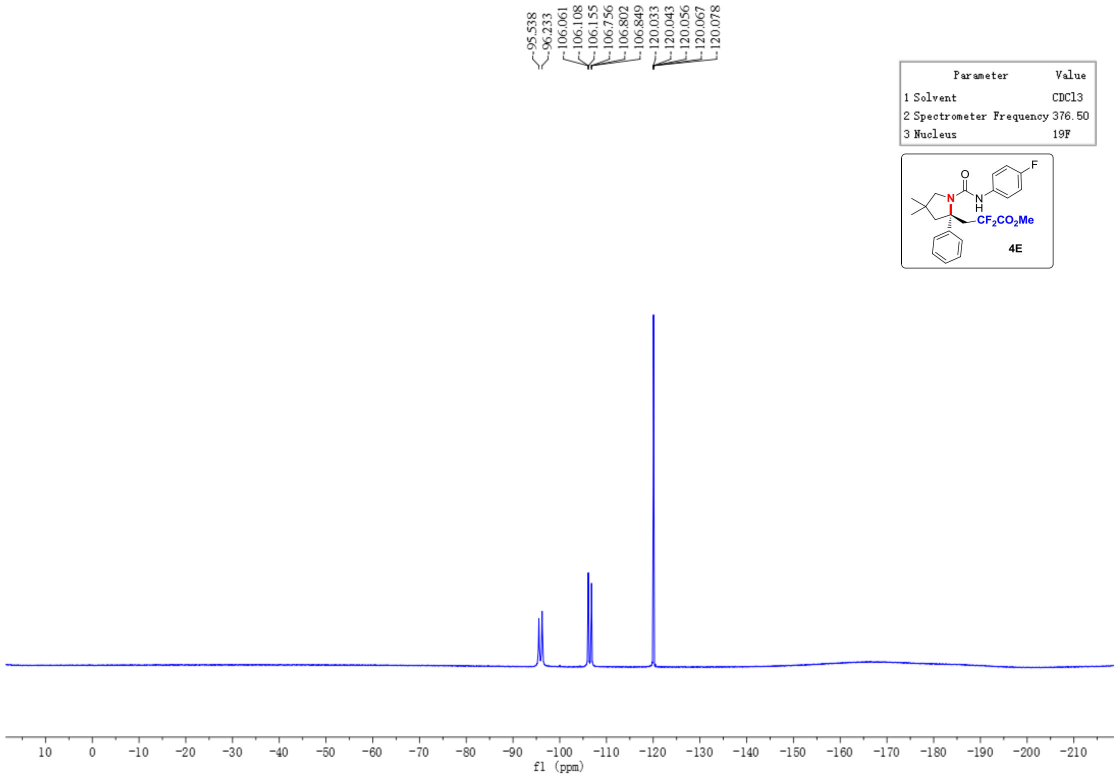

Supplementary Figure 86. ^{19}F NMR of **4E**

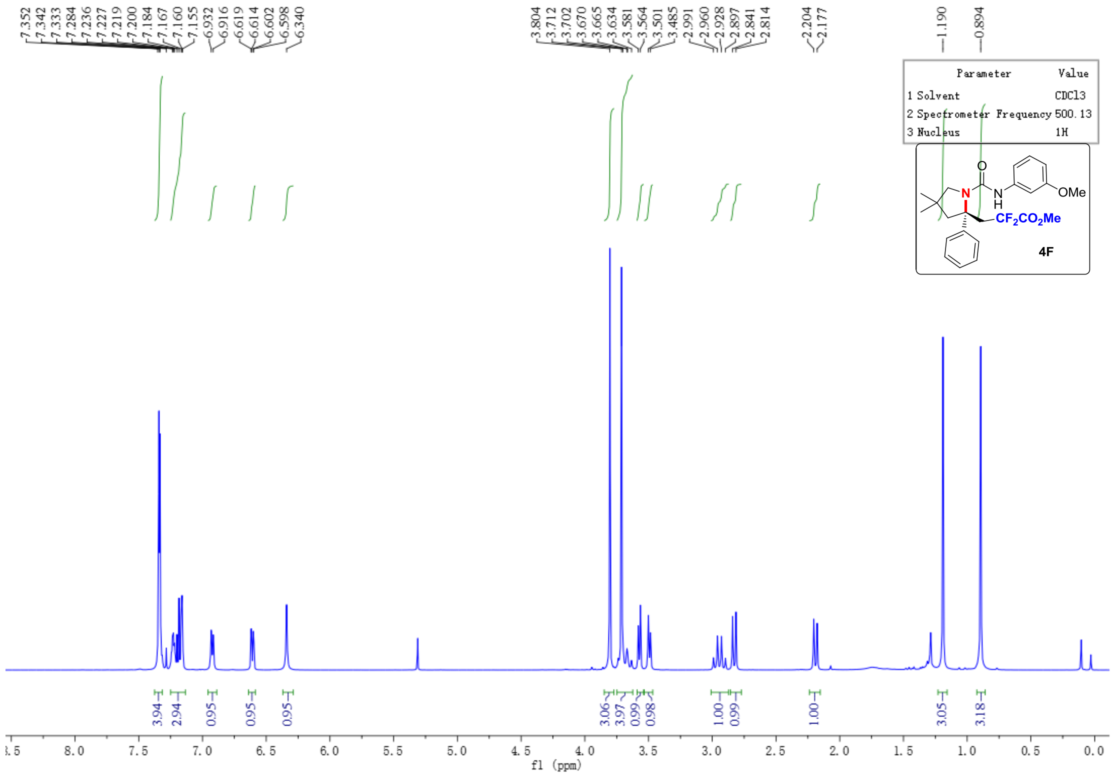

Supplementary Figure 87. ^{1}H NMR of **4F**

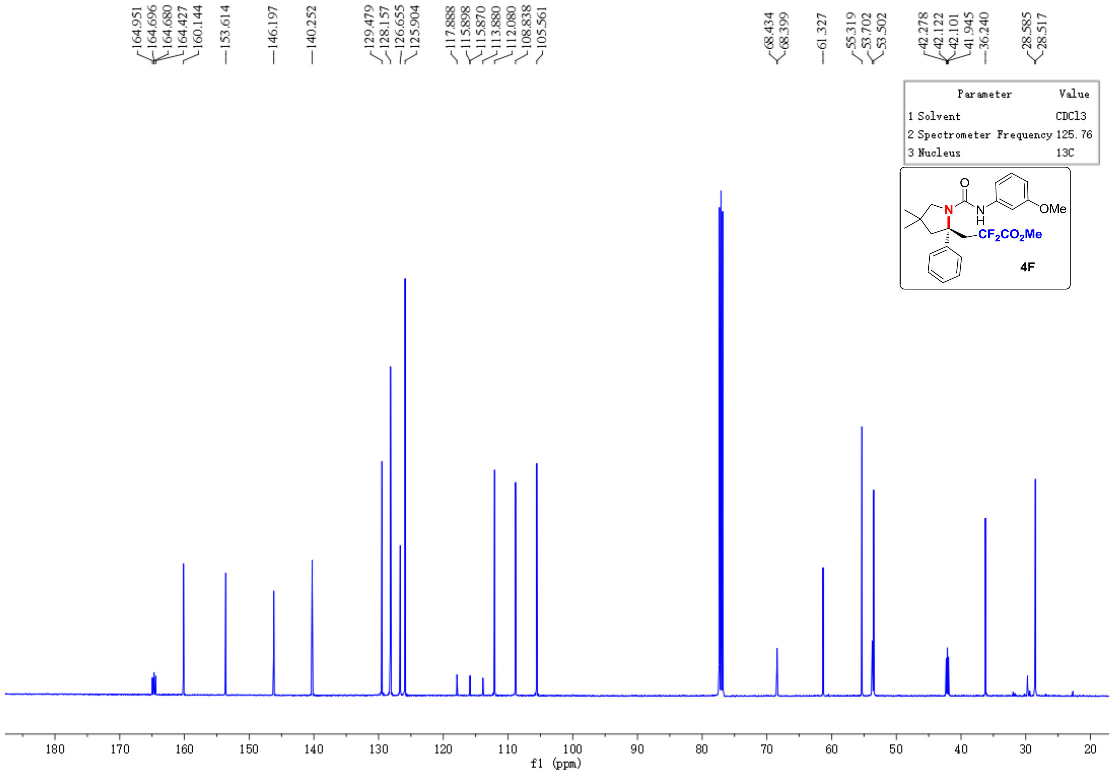

Supplementary Figure 88. ^{13}C NMR of **4F**

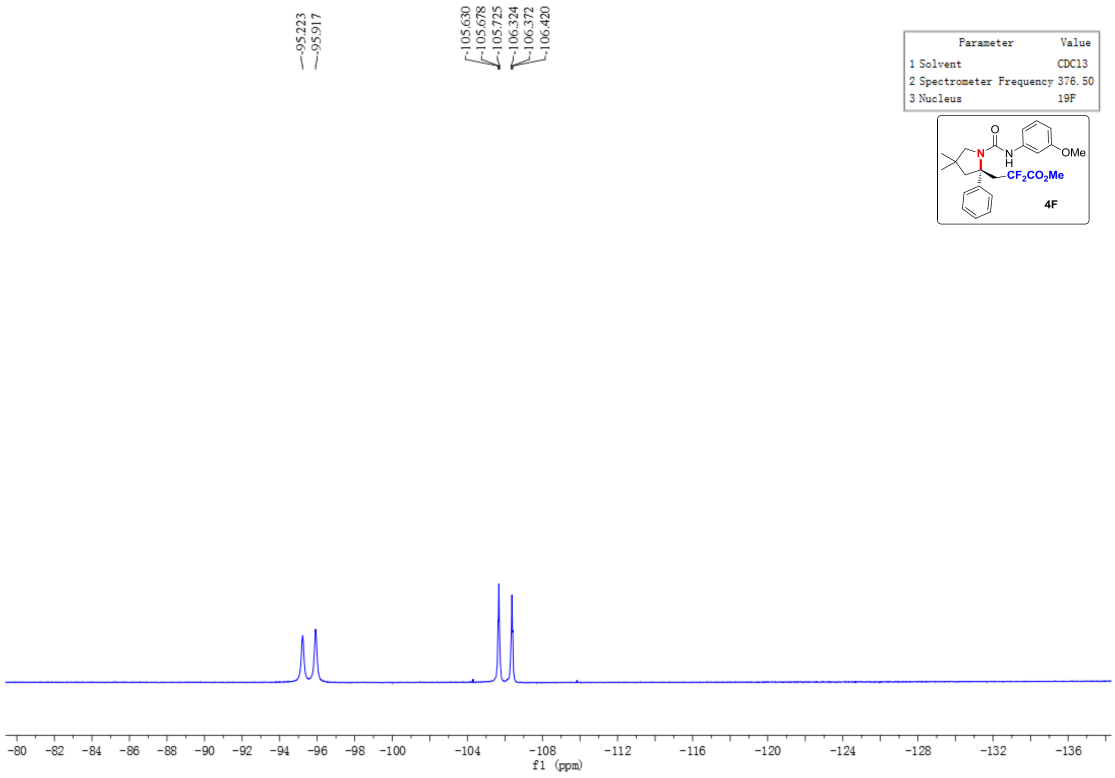

Supplementary Figure 89. ^{19}F NMR of **4F**

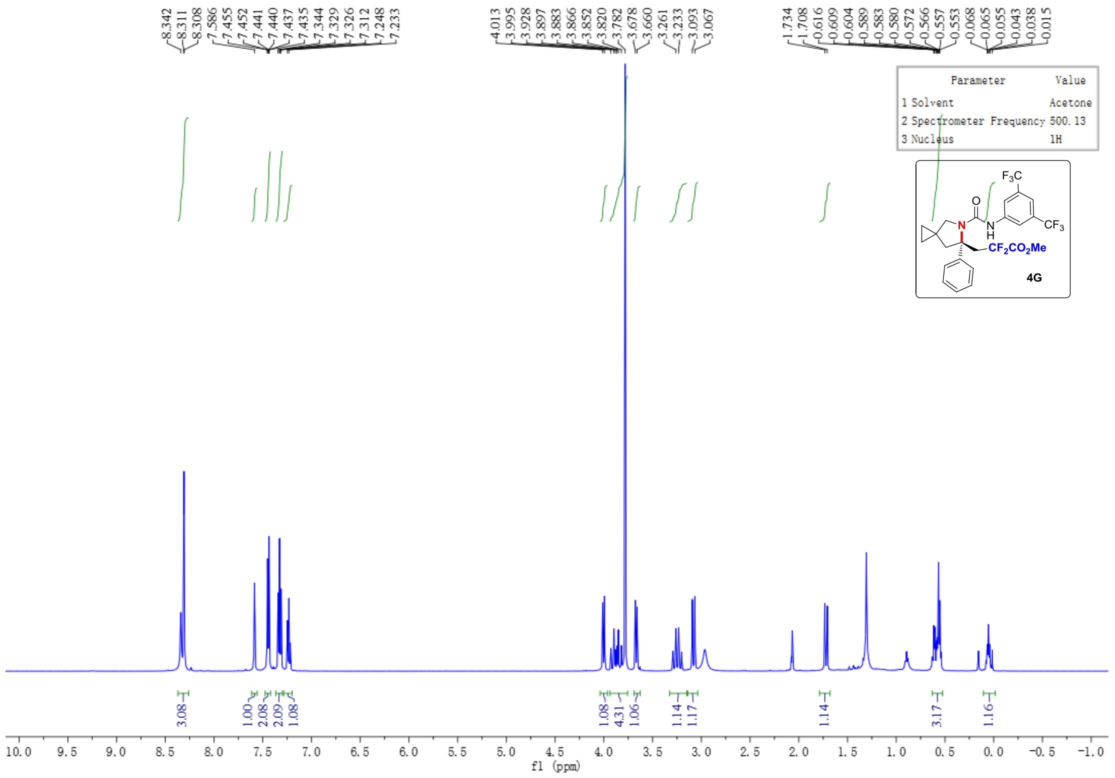

Supplementary Figure 90. ^{1}H NMR of **4G**

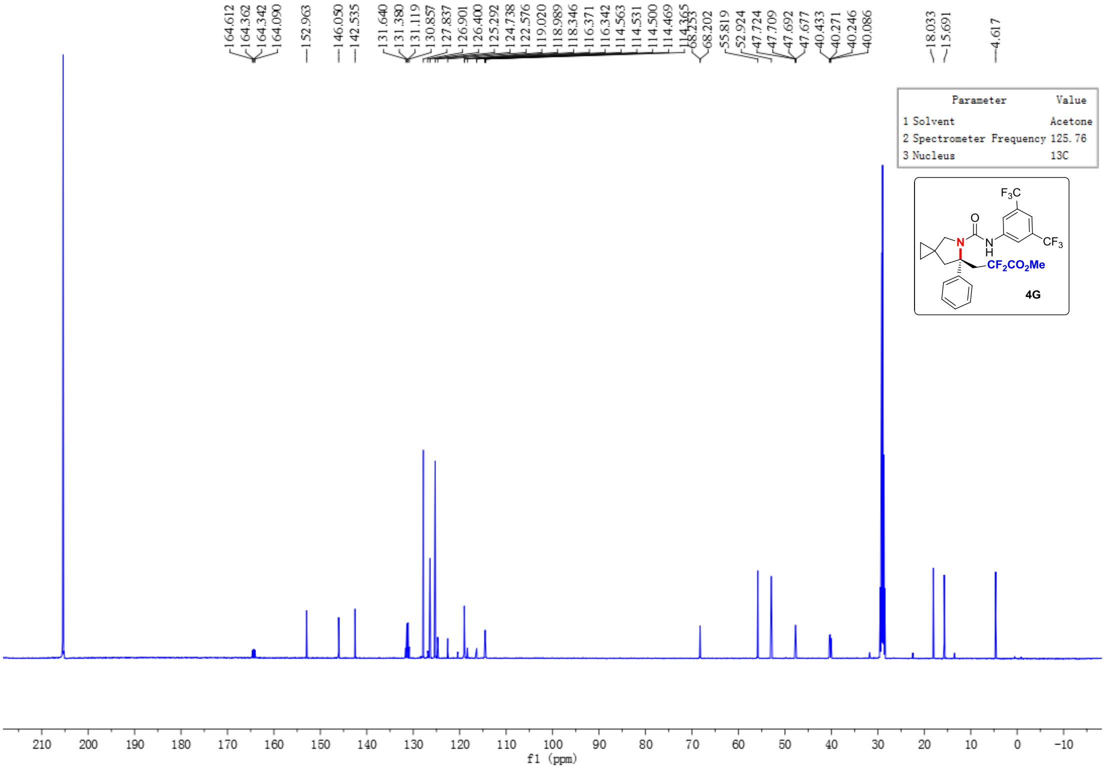

Supplementary Figure 91. ^{13}C NMR of **4G**

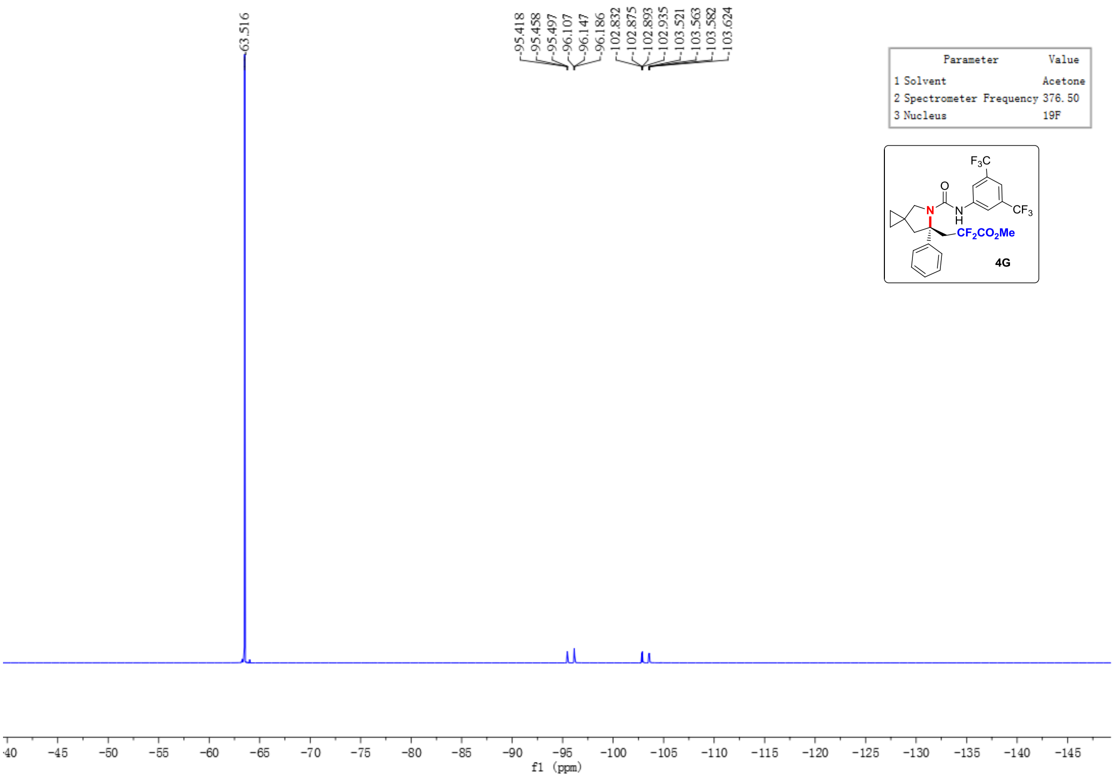

Supplementary Figure 92. ^{19}F NMR of **4G**

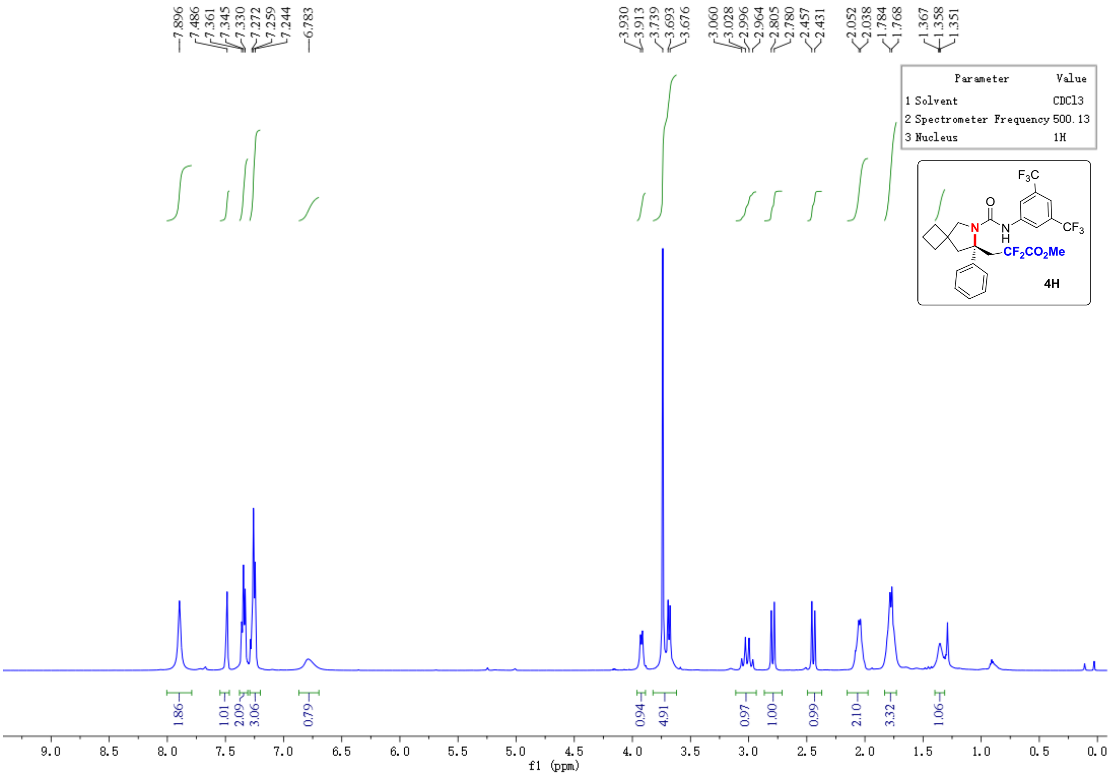

Supplementary Figure 93. ^{1}H NMR of **4H**

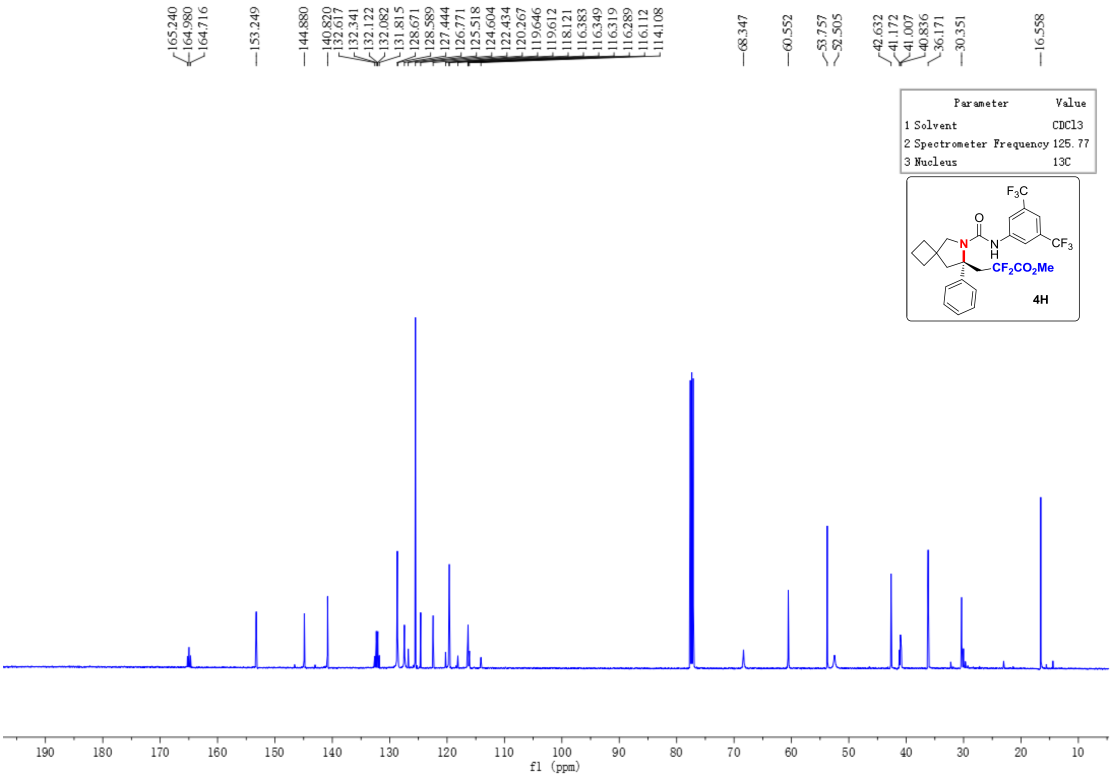

Supplementary Figure 94. ^{13}C NMR of **4H**

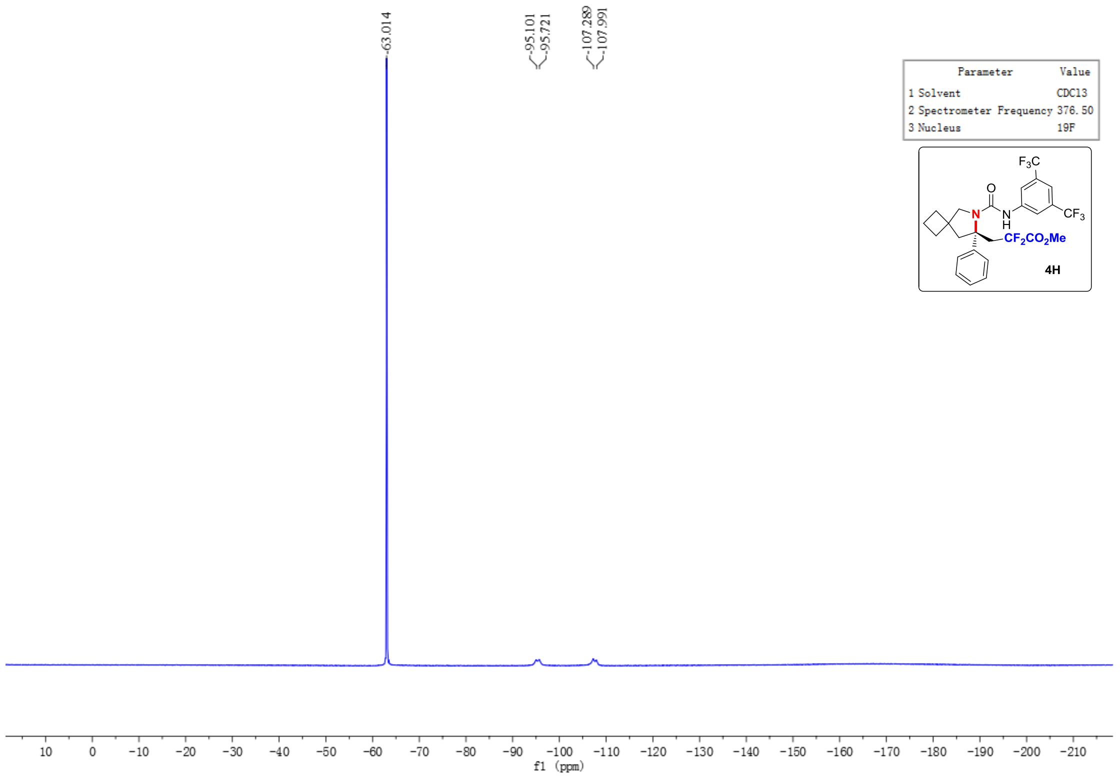

Supplementary Figure 95. ^{19}F NMR of **4H**

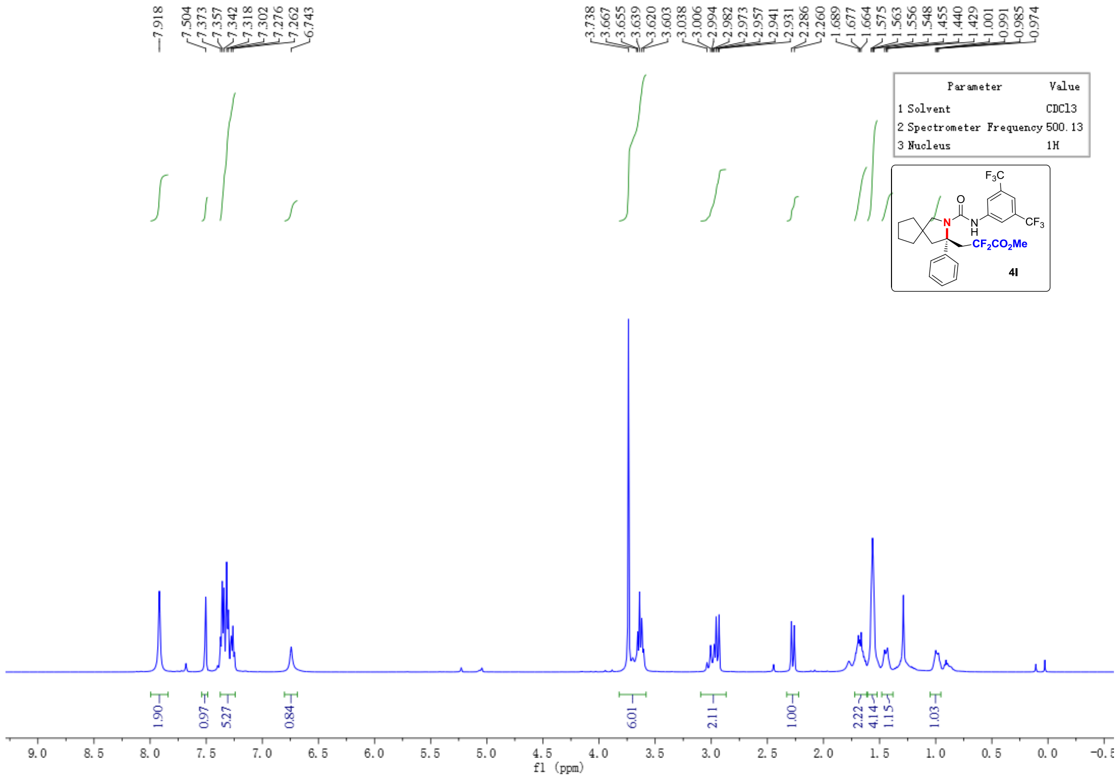

Supplementary Figure 96. ^{1}H NMR of **4I**

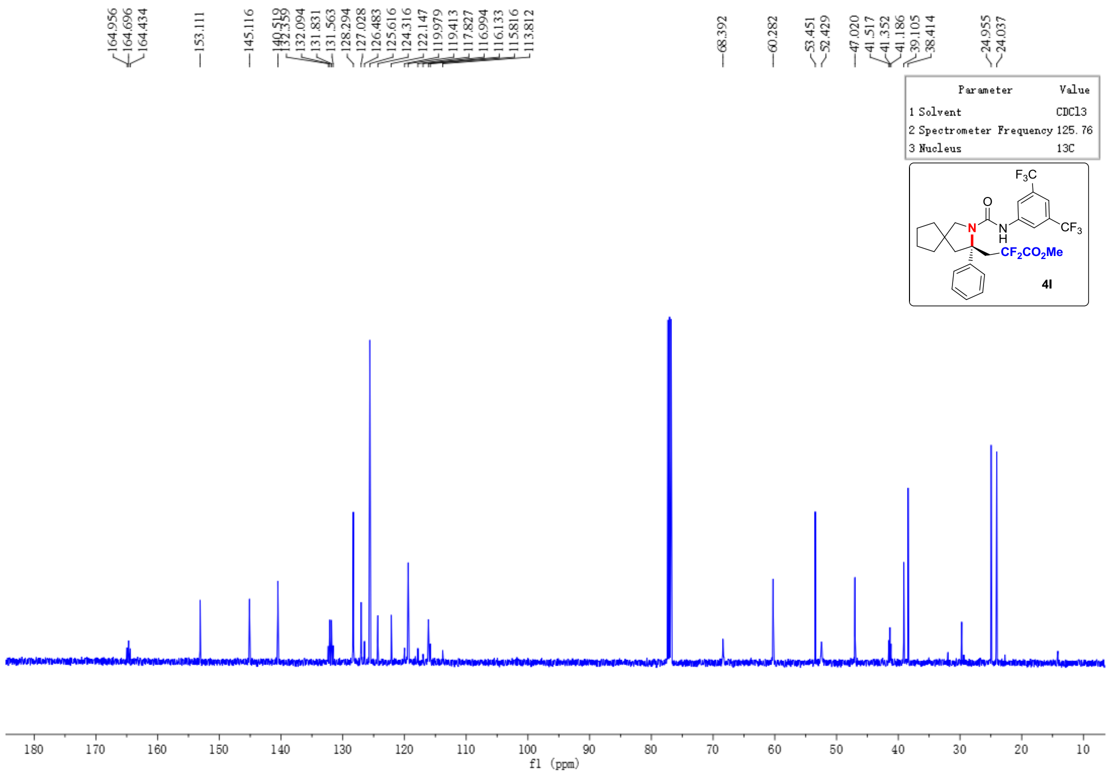

Supplementary Figure 97. ^{13}C NMR of **4l**

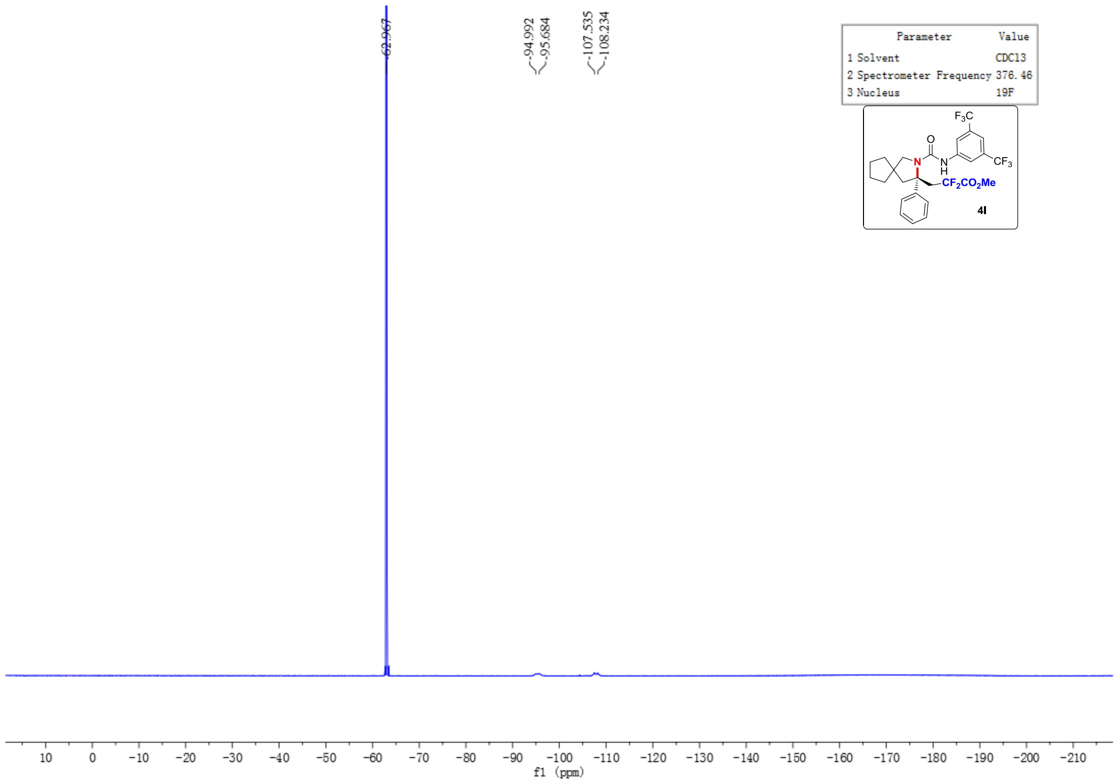

Supplementary Figure 98. ^{19}F NMR of **4l**

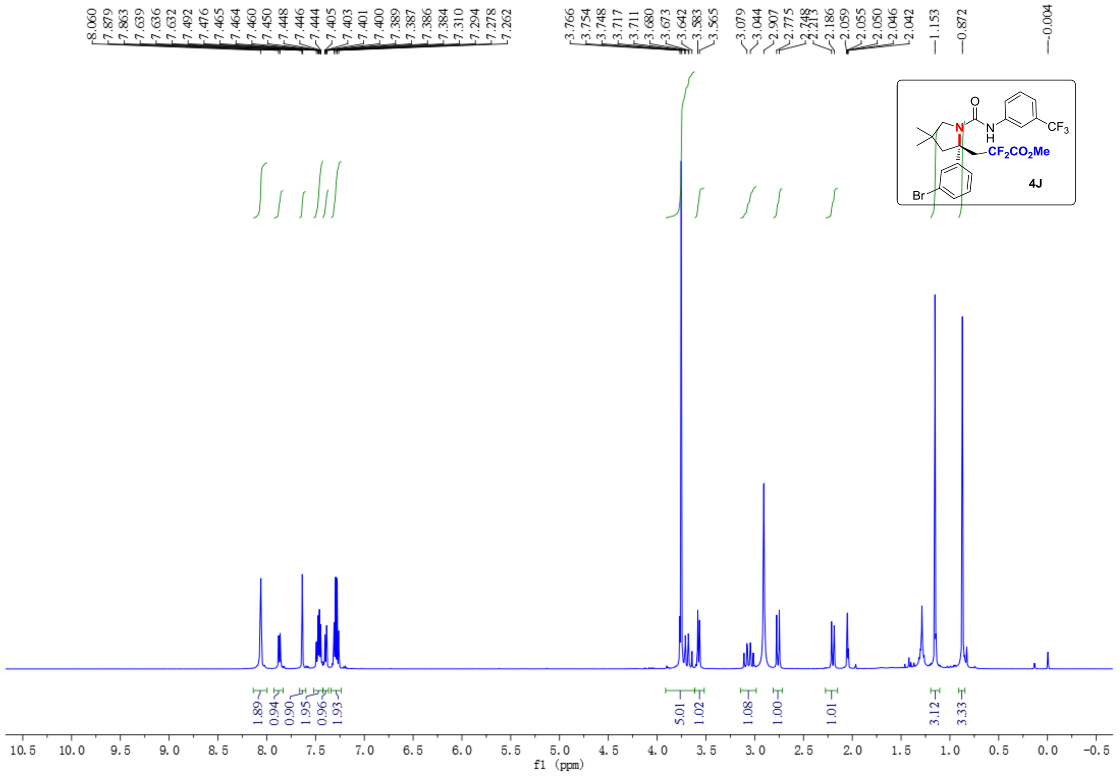

Supplementary Figure 99. ^{1}H NMR of **4J**

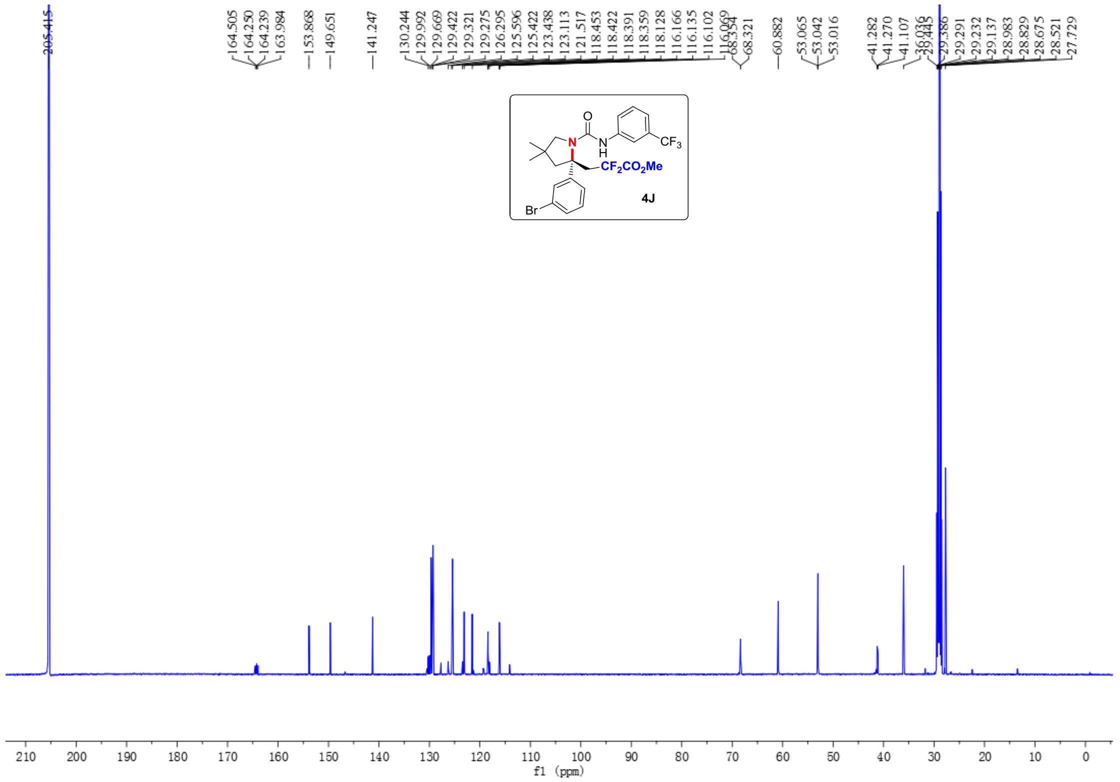

Supplementary Figure 100. ^{13}C NMR of **4J**

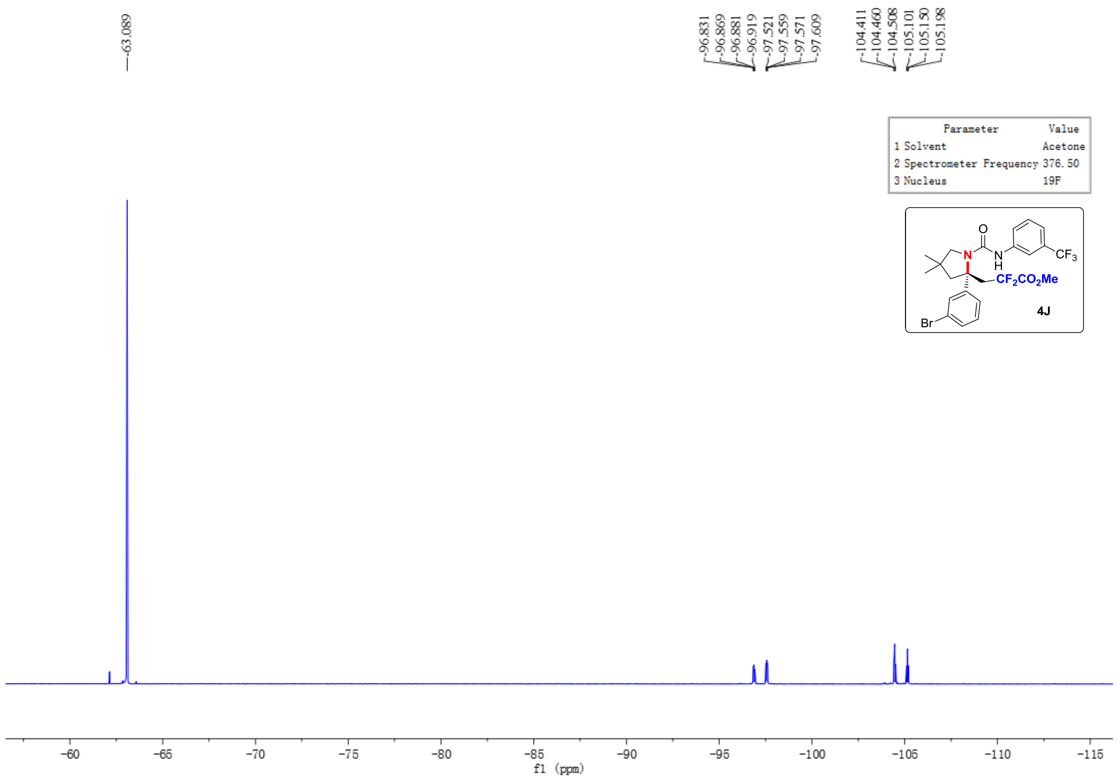

Supplementary Figure 101. ^{19}F NMR of **4J**

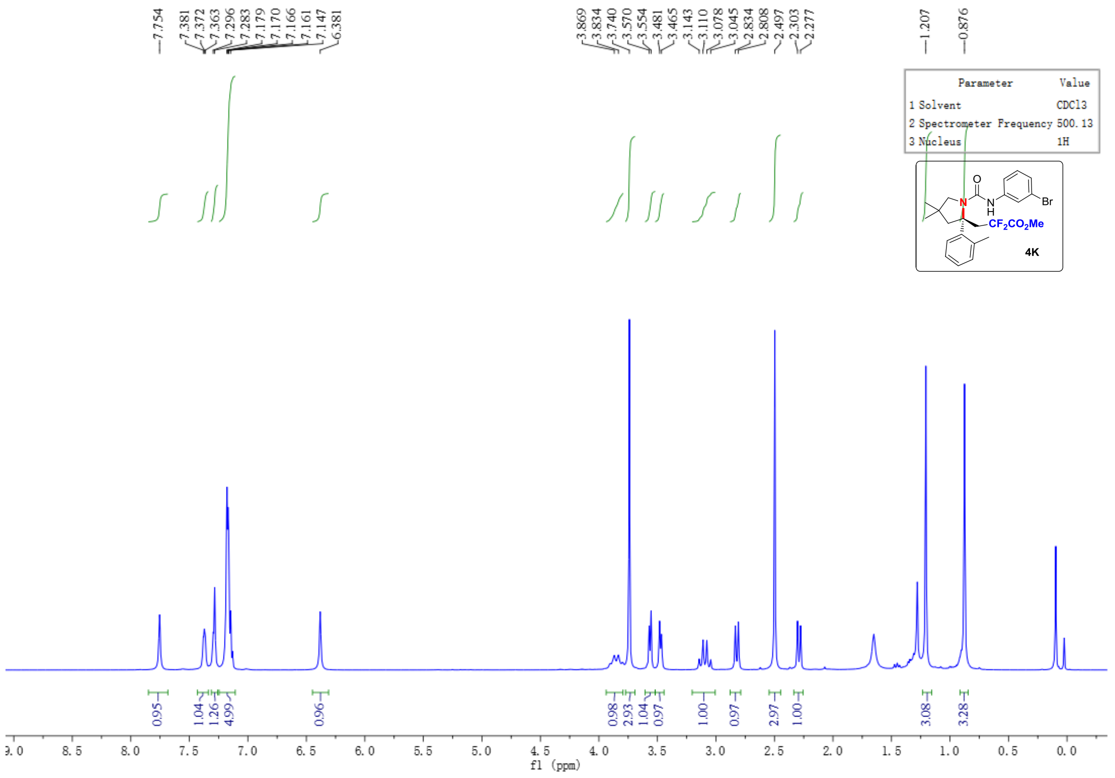

Supplementary Figure 102. ^{1}H NMR of **4K**

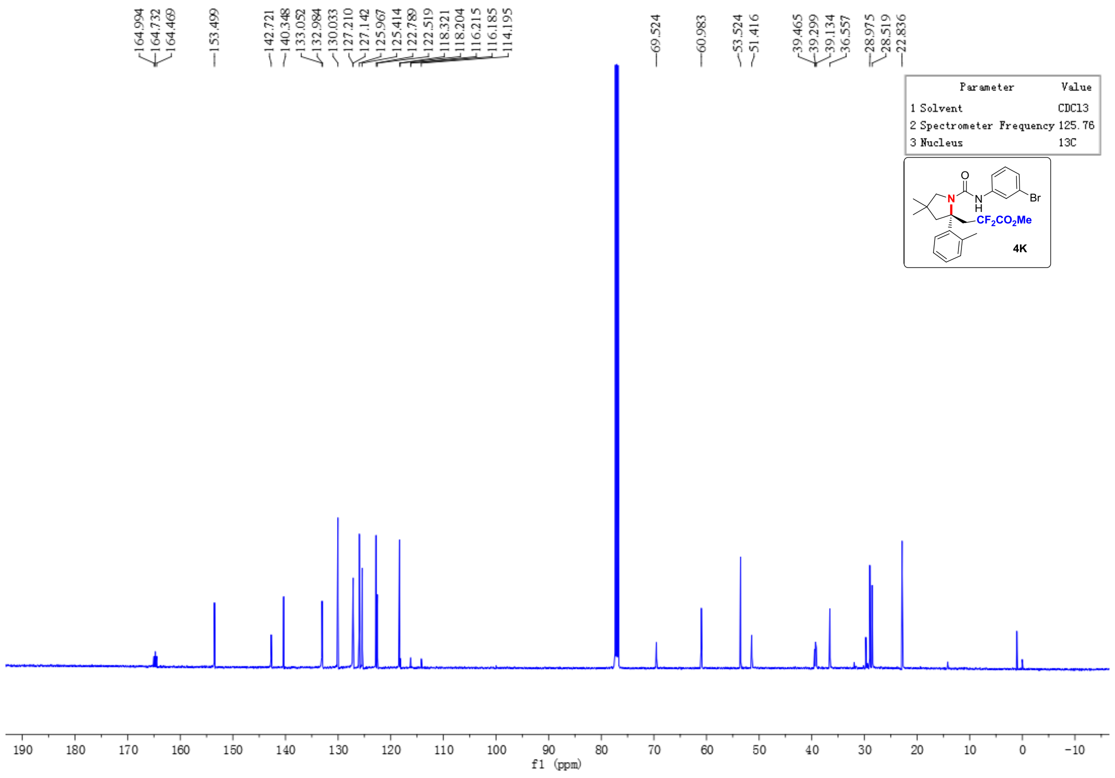

Supplementary Figure 103. ^{13}C NMR of **4K**

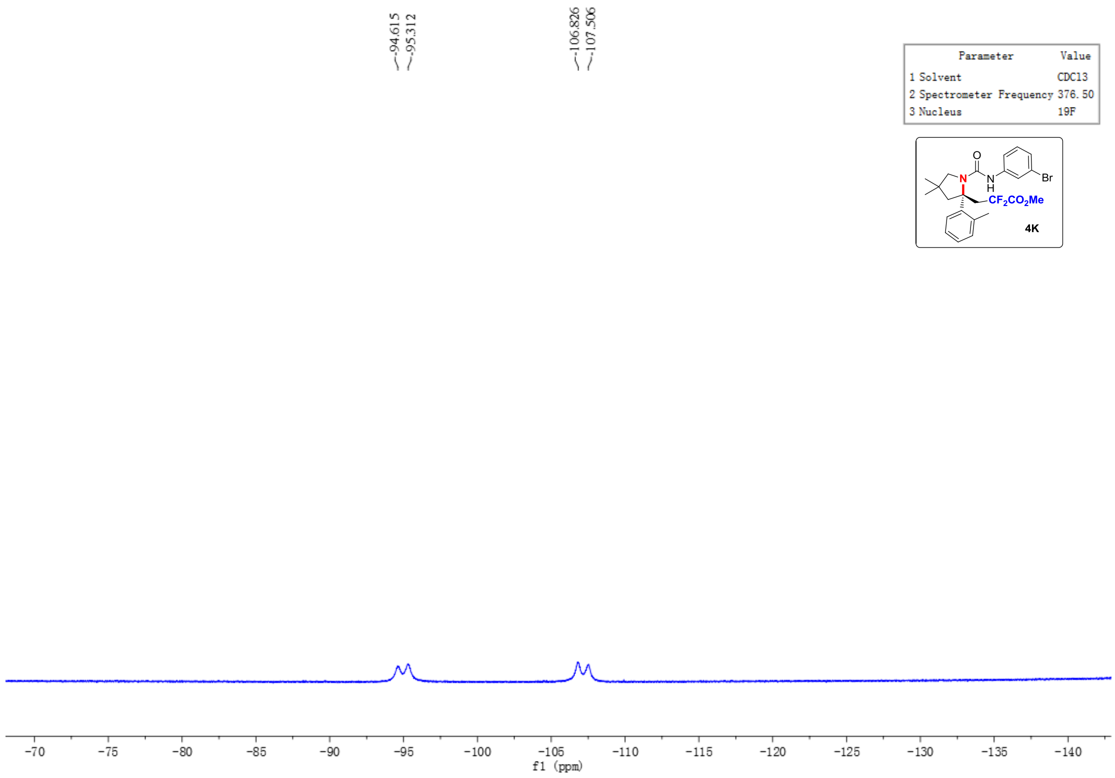

Supplementary Figure 104. ^{19}F NMR of **4K**

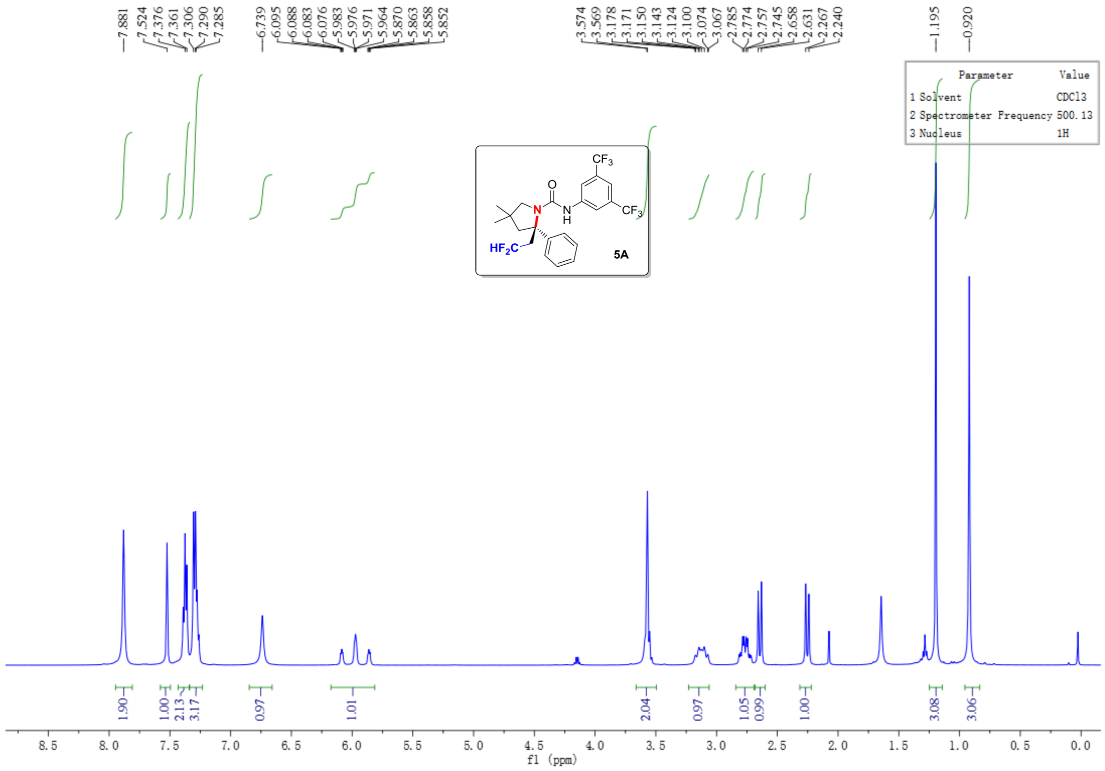

Supplementary Figure 105. ^{1}H NMR of **5A**

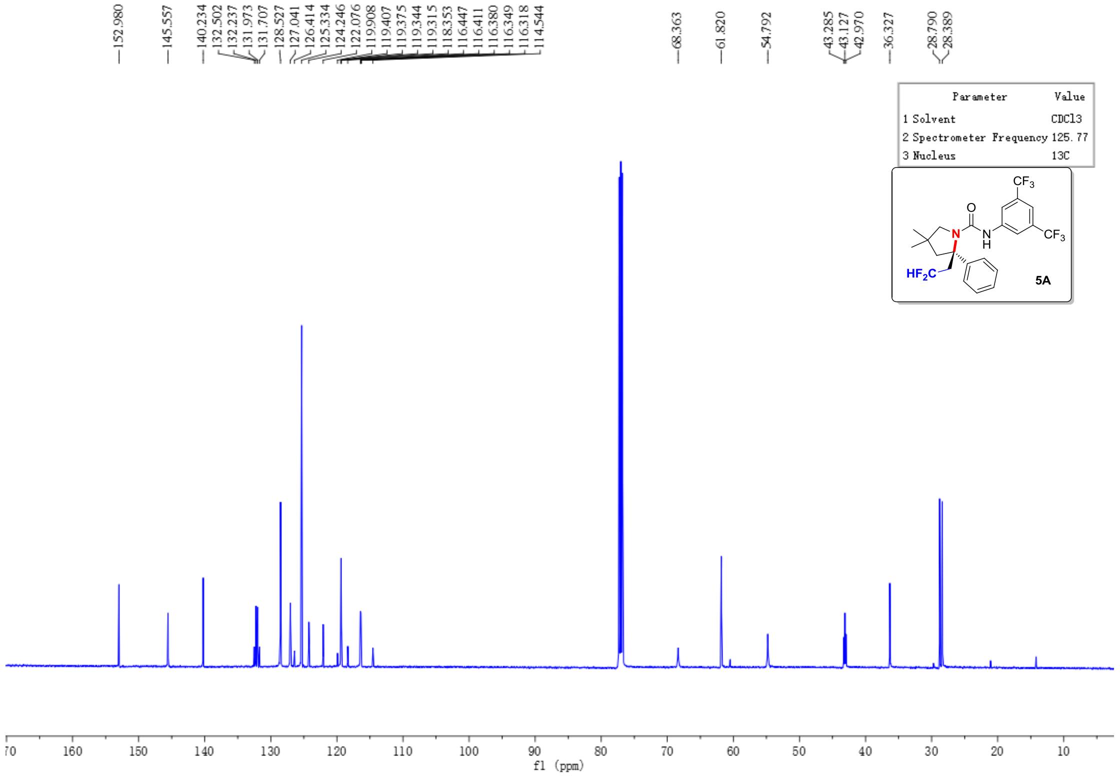

Supplementary Figure 106. ^{13}C NMR of **5A**

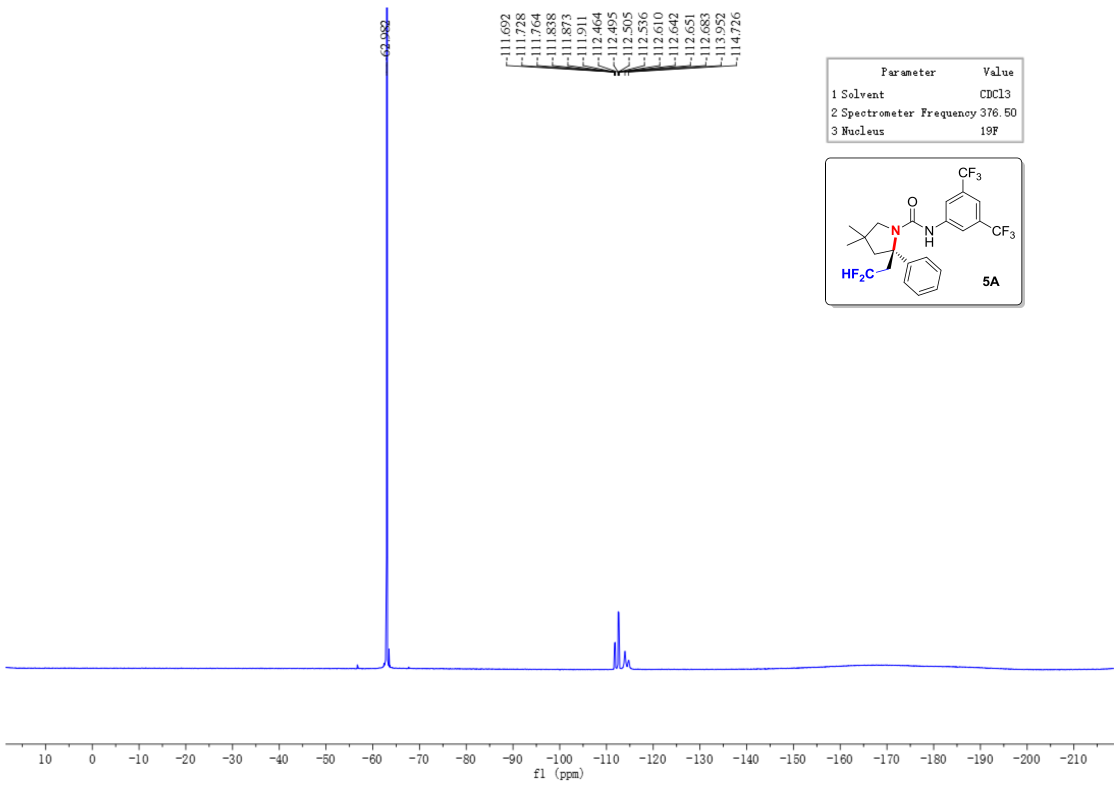

Supplementary Figure 107. ^{19}F NMR of **5A**

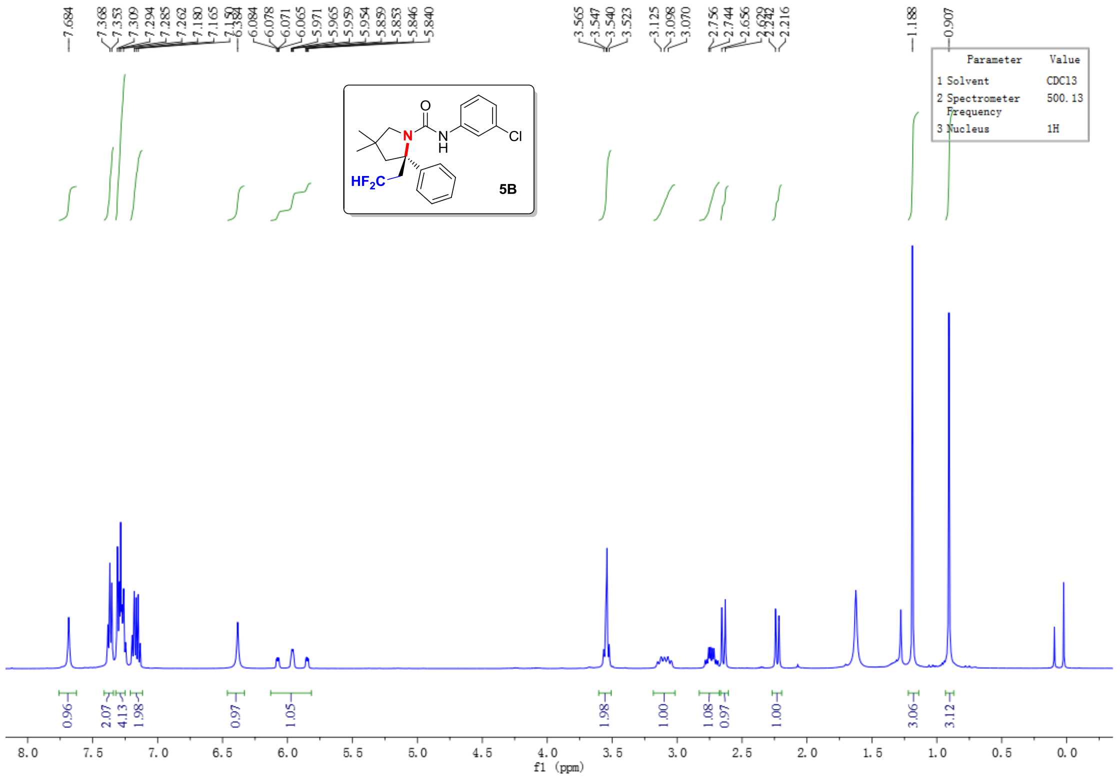

Supplementary Figure 108. ^{1}H NMR of **5B**

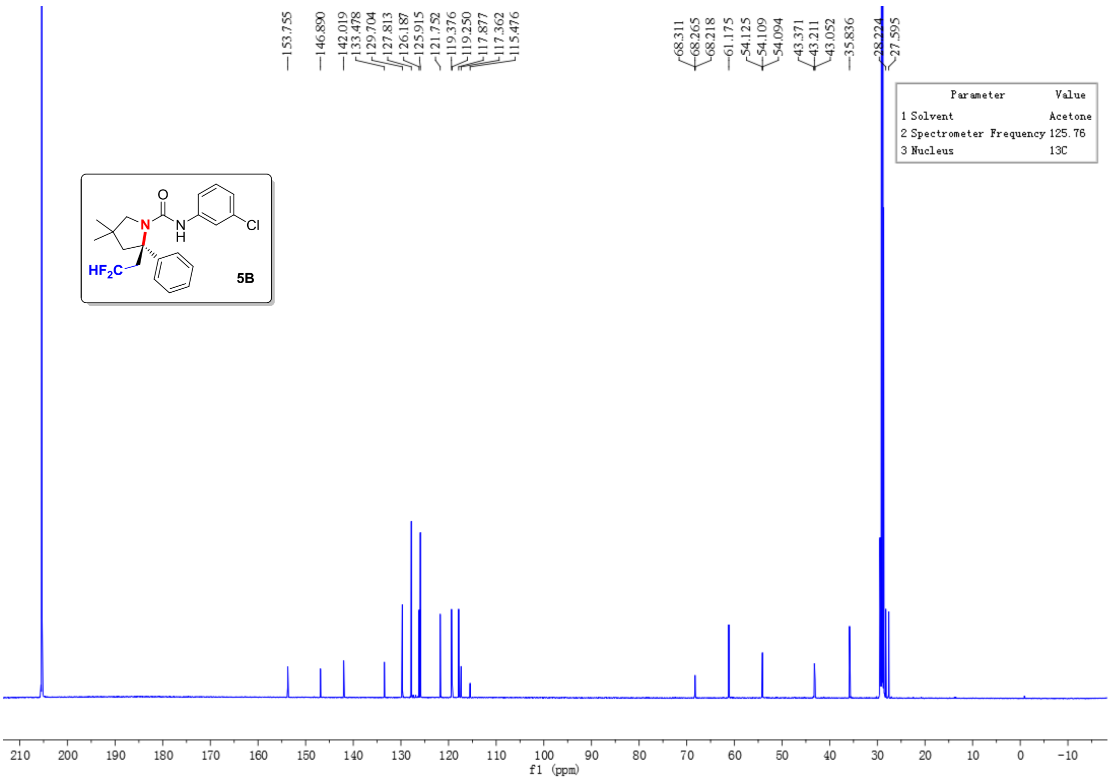

Supplementary Figure 109. ^{13}C NMR of **5B**

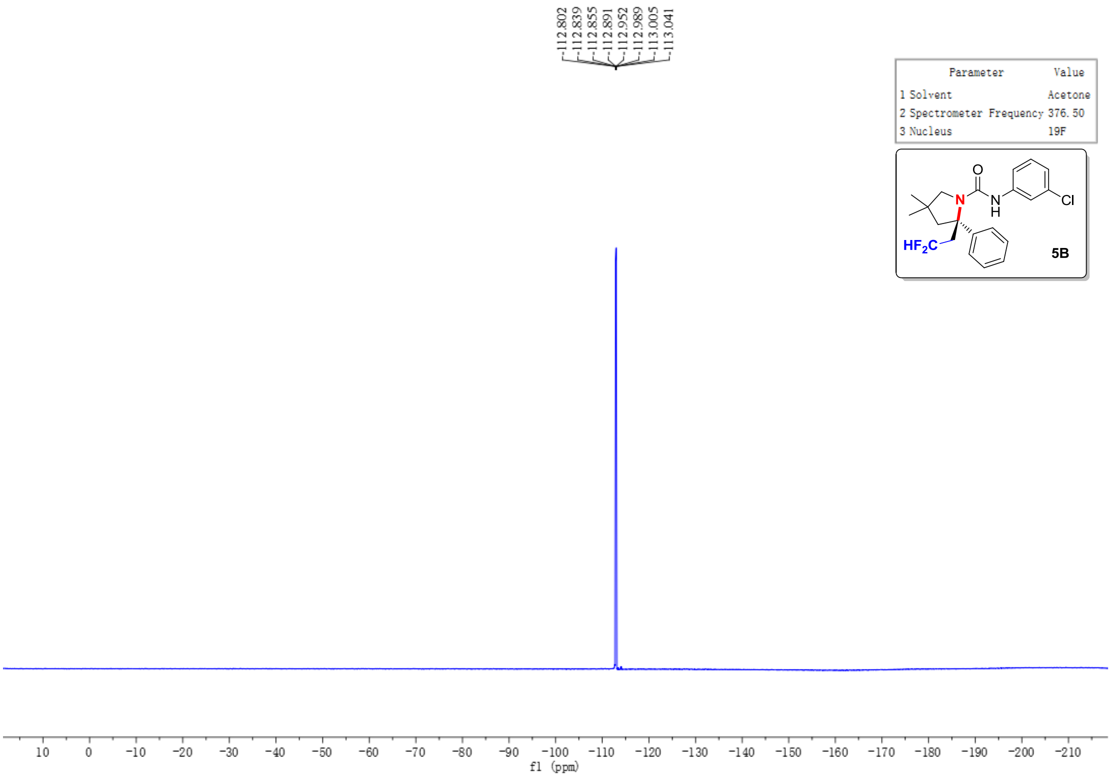

Supplementary Figure 110. ^{19}F NMR of **5B**

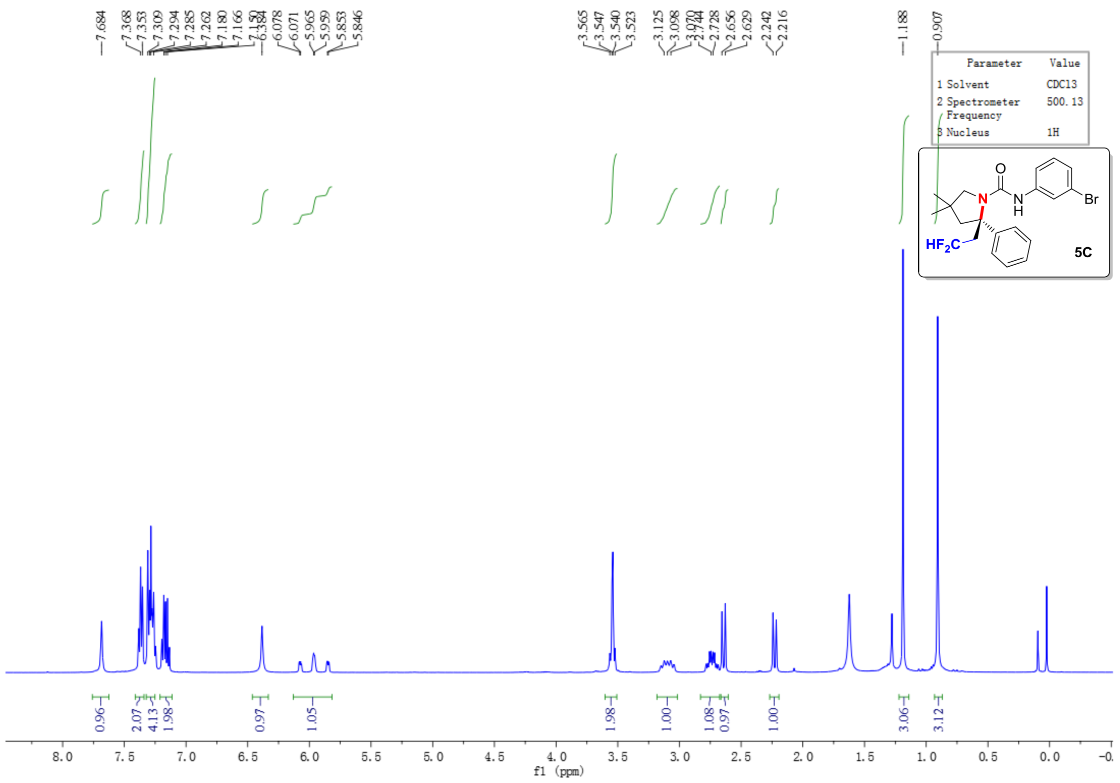

Supplementary Figure 111. ^{1}H NMR of **5C**

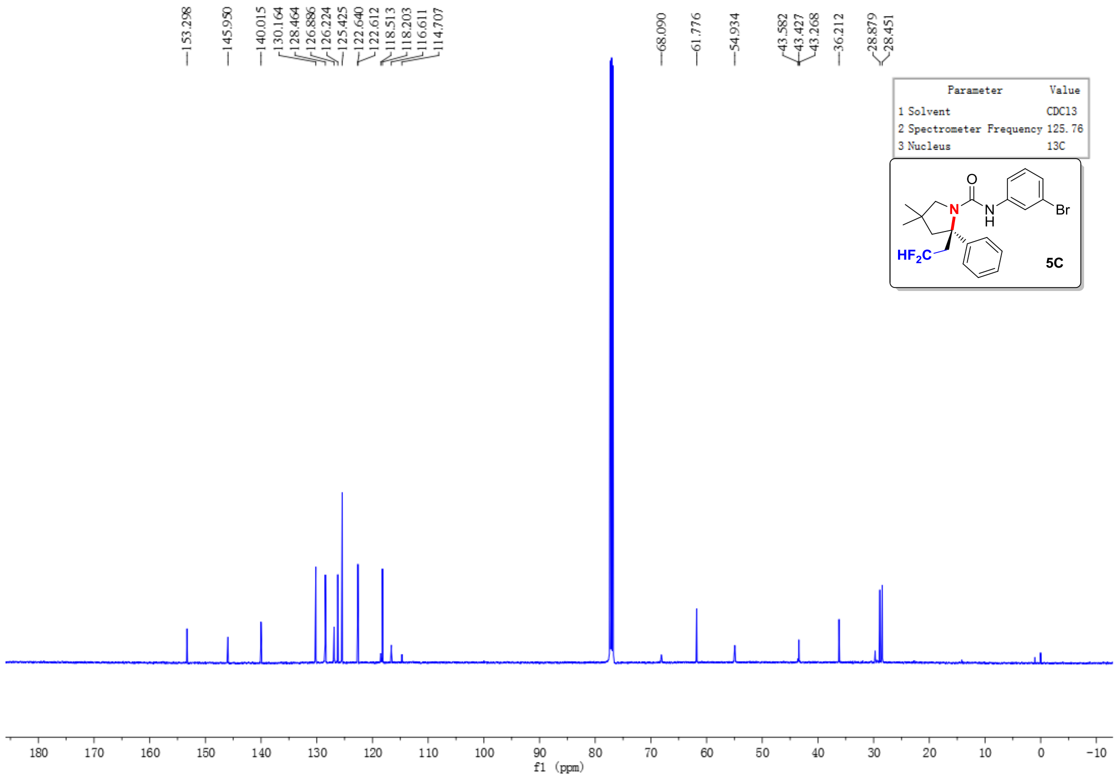

Supplementary Figure 112. ^{13}C NMR of **5C**

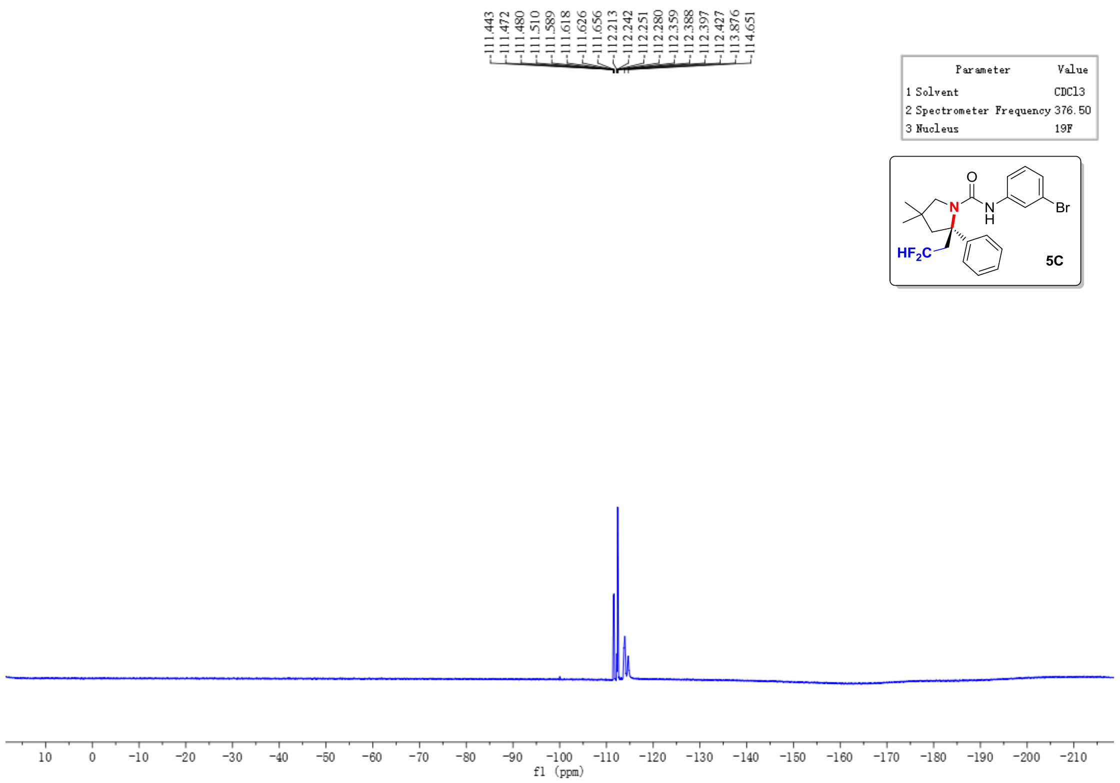

Supplementary Figure 113. ^{19}F NMR of **5C**

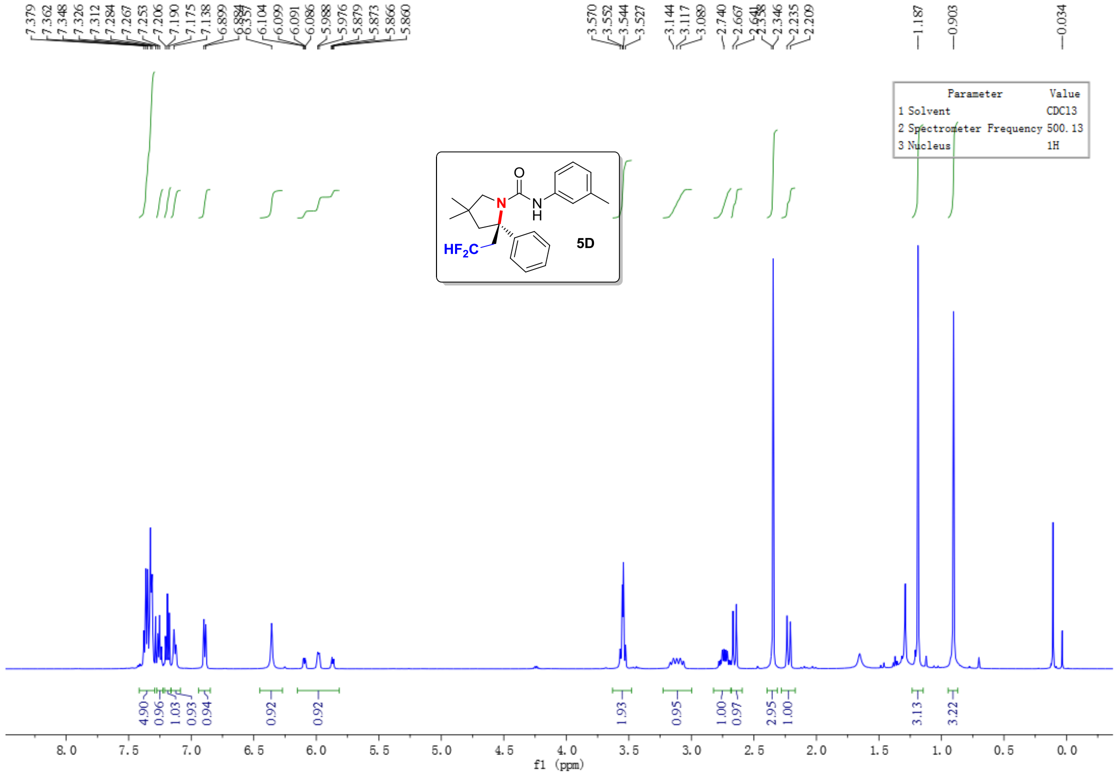

Supplementary Figure 114. ^{1}H NMR of **5D**

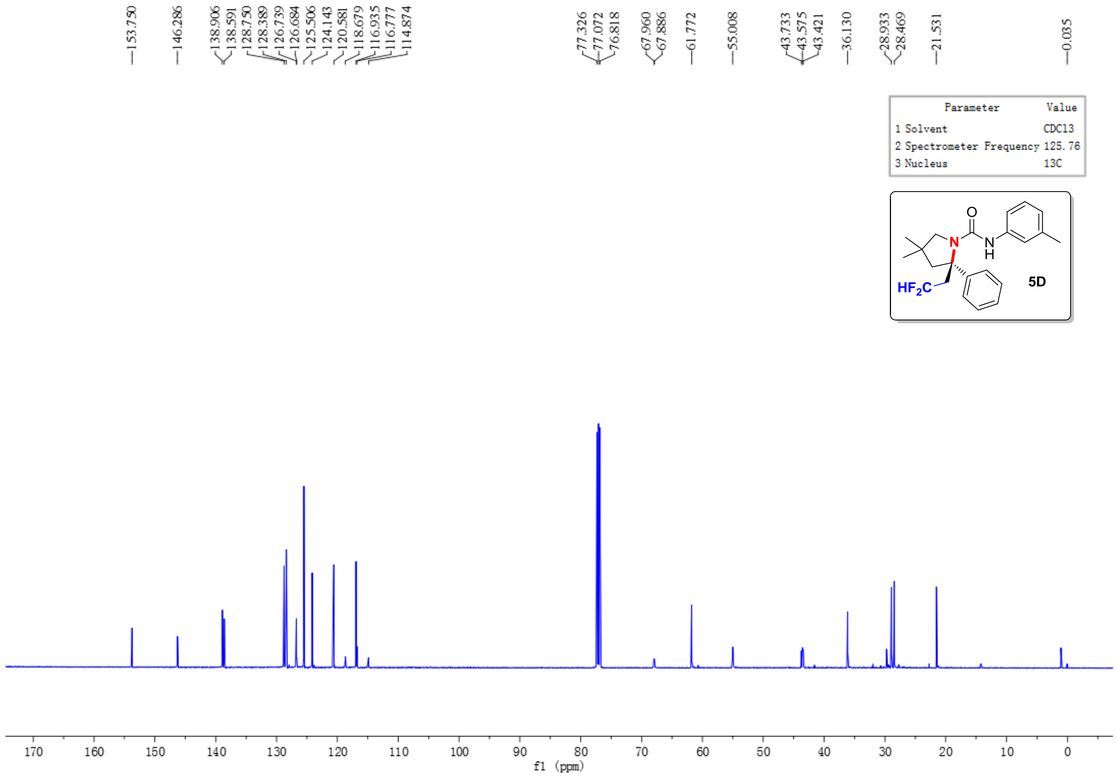

Supplementary Figure 115. ^{13}C NMR of **5D**

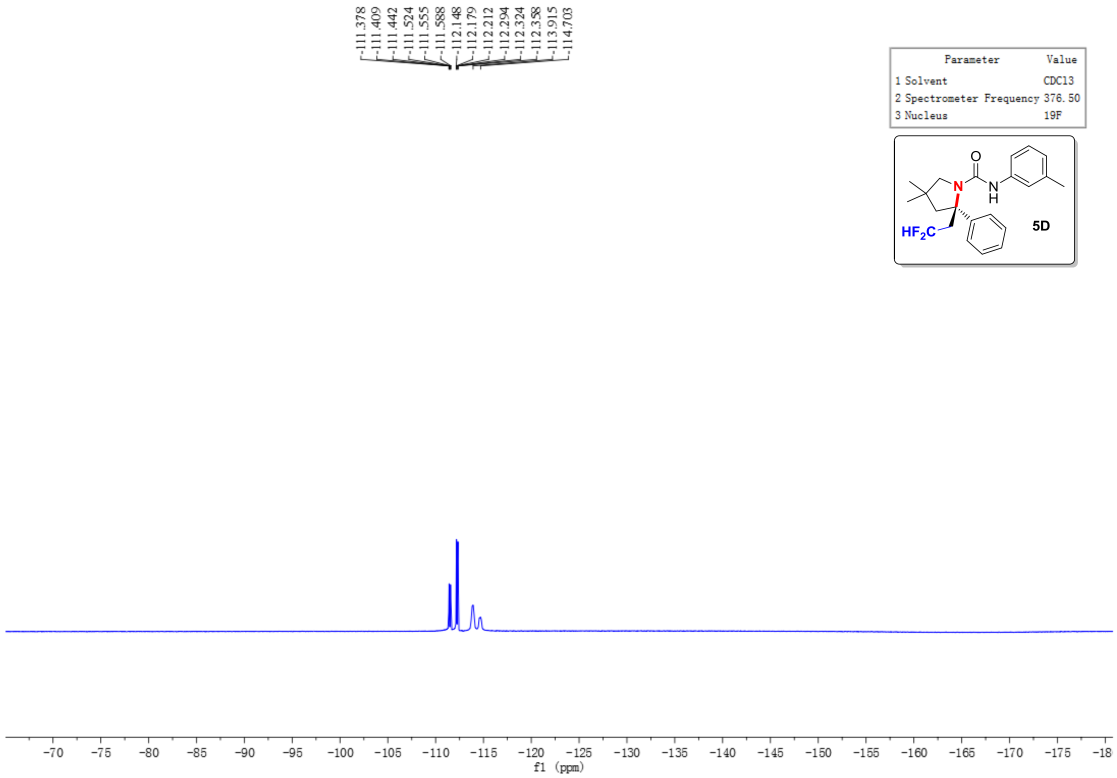

Supplementary Figure 116. ^{19}F NMR of **5D**

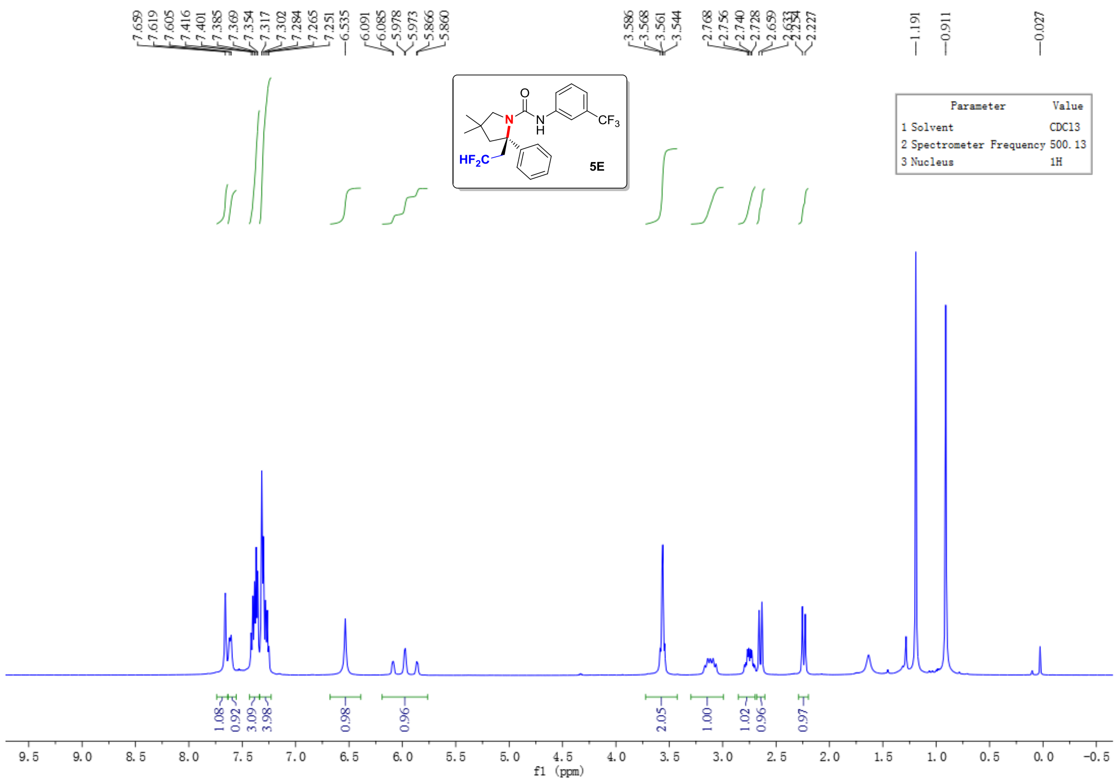

Supplementary Figure 117. ^{1}H NMR of **5E**

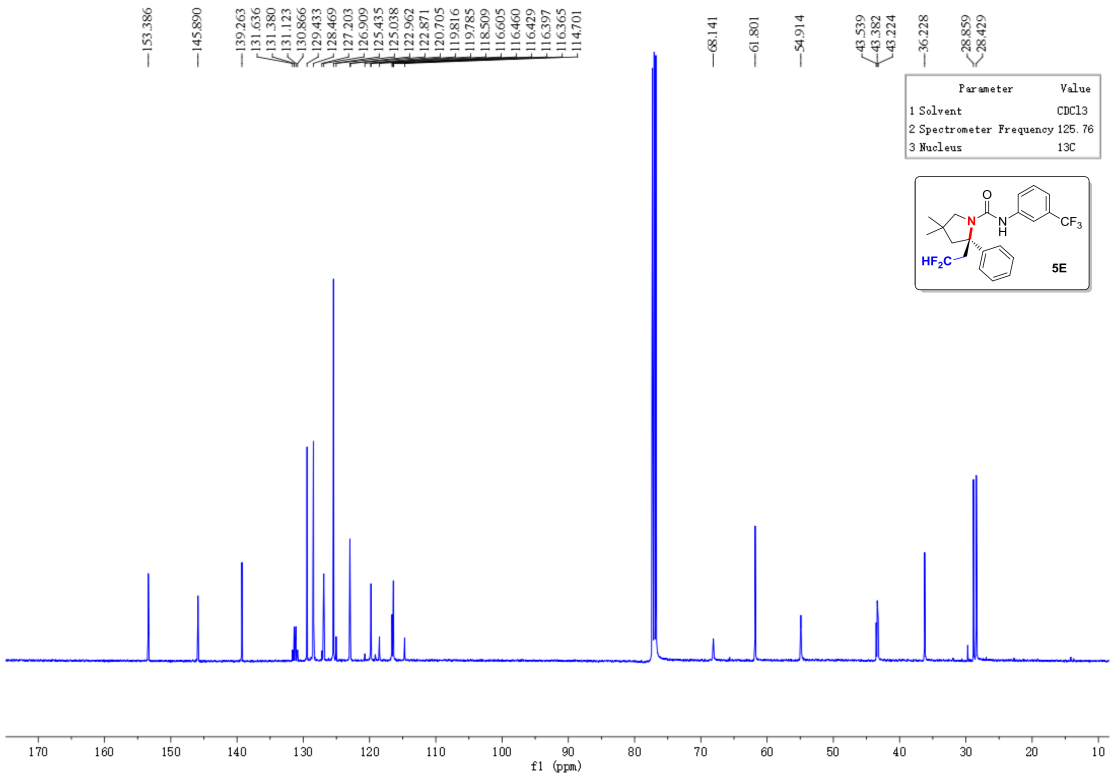

Supplementary Figure 118. ^{13}C NMR of **5E**

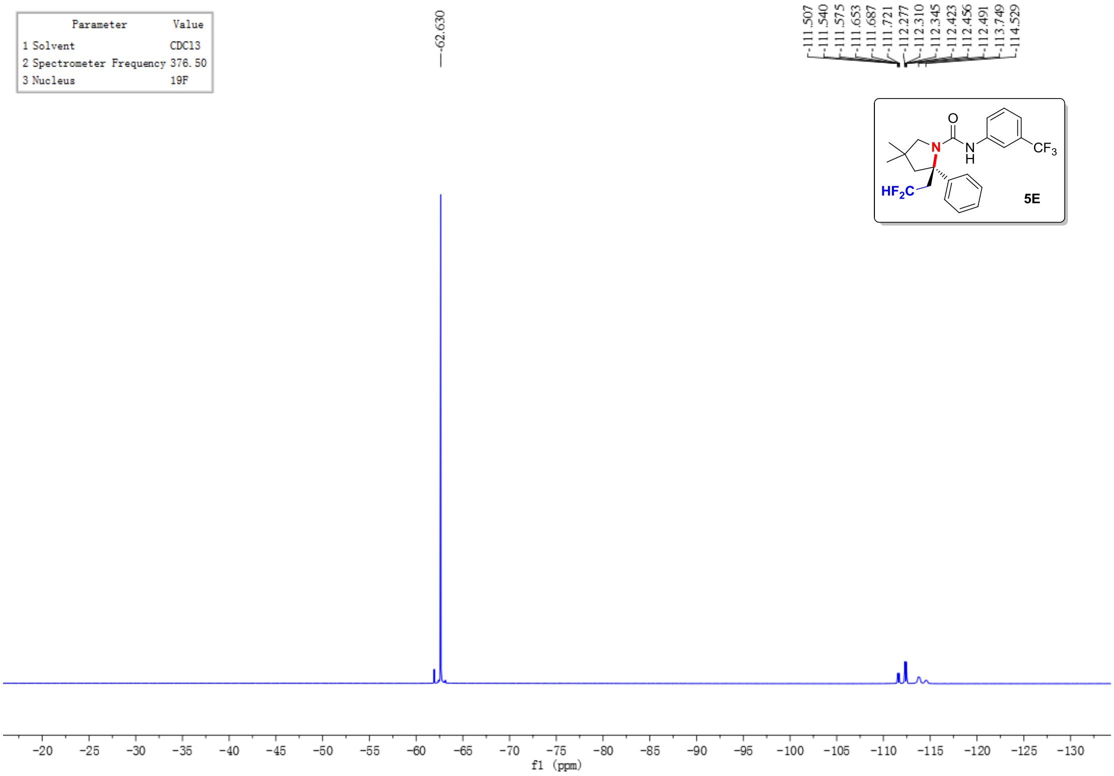

Supplementary Figure 119. ^{19}F NMR of **5E**

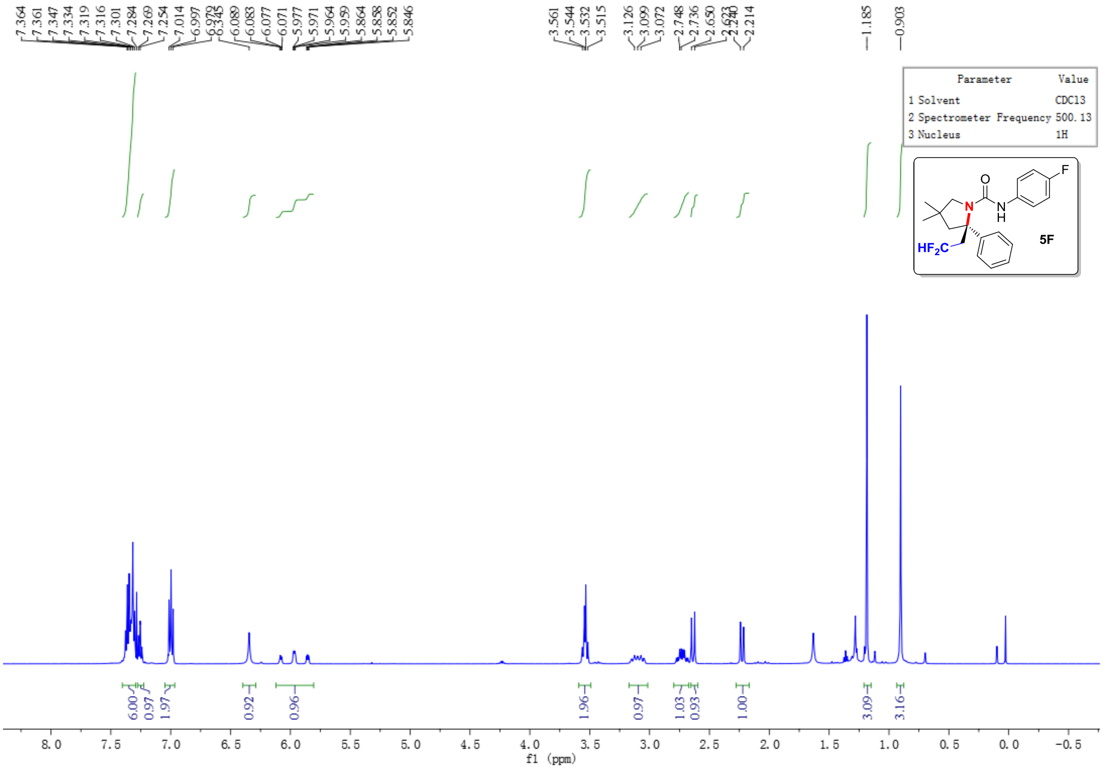

Supplementary Figure 120. ^{1}H NMR of **5F**

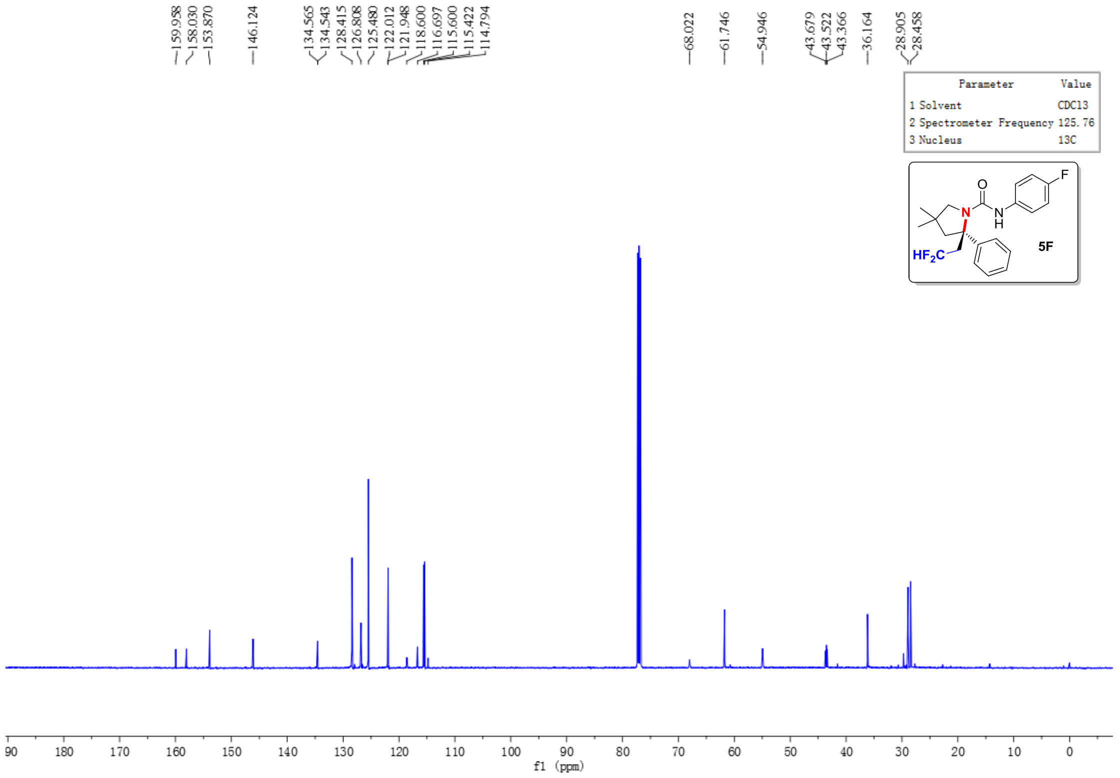

Supplementary Figure 121. ^{13}C NMR of **5F**

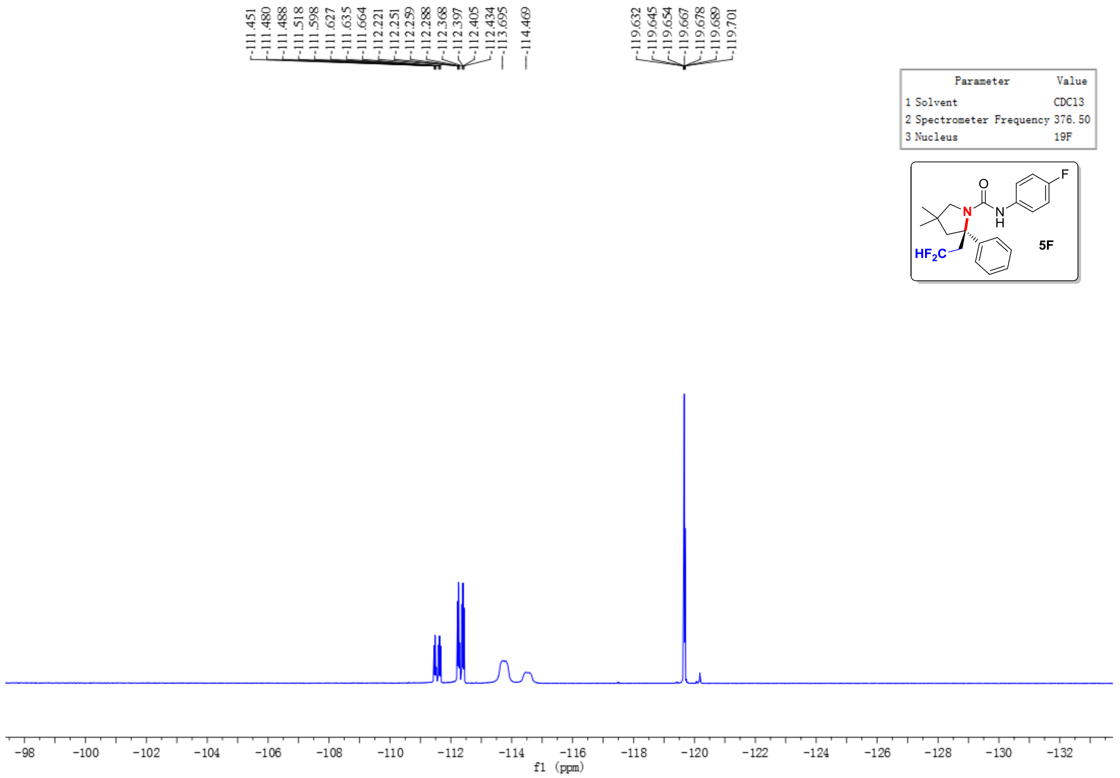

Supplementary Figure 122. ^{19}F NMR of **5F**

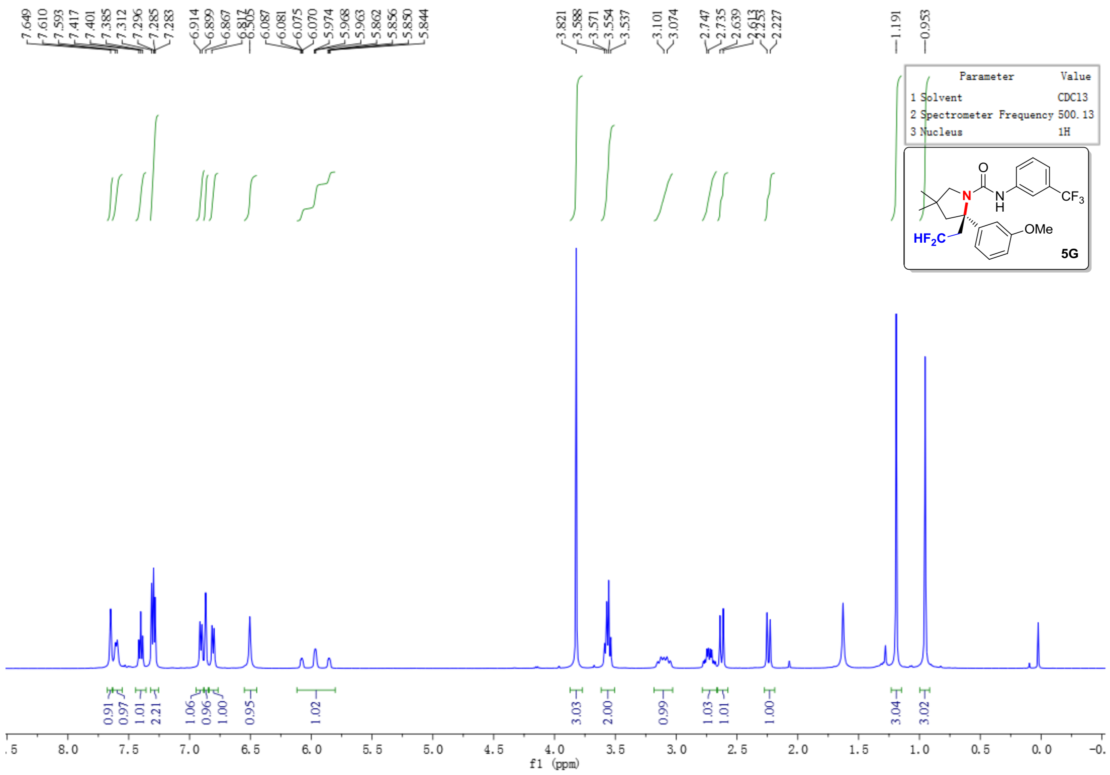

Supplementary Figure 123. ^{1}H NMR of **5G**

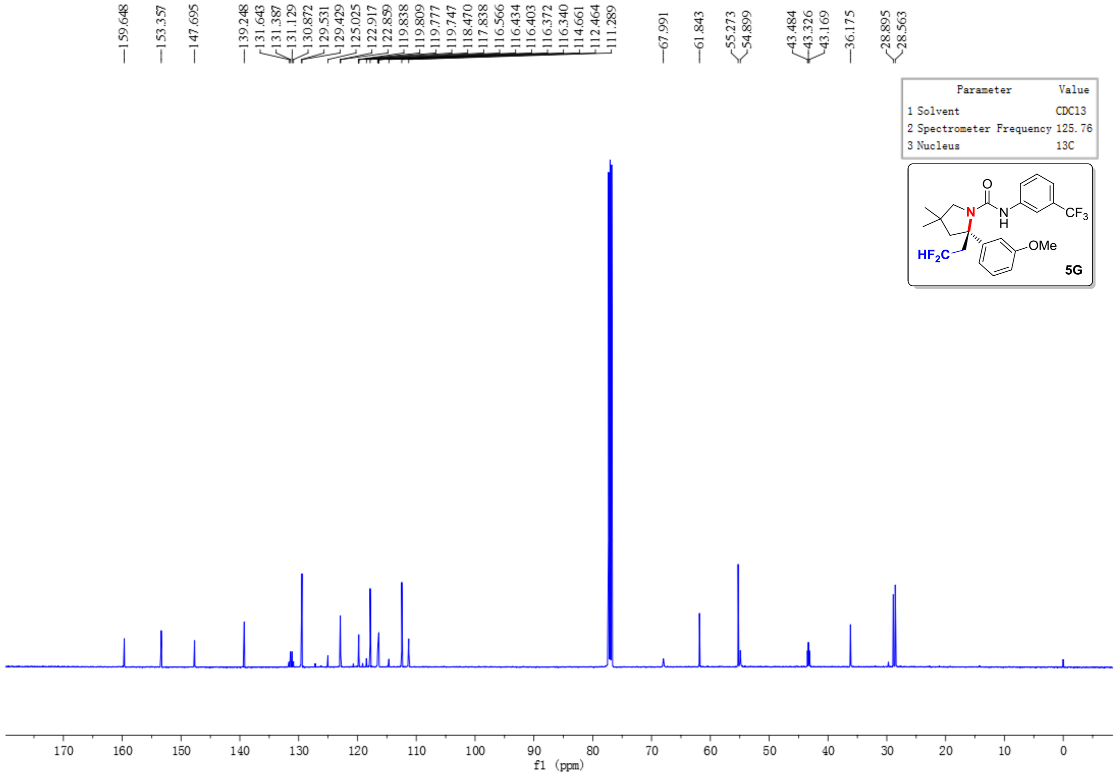

Supplementary Figure 124. ^{13}C NMR of **5G**

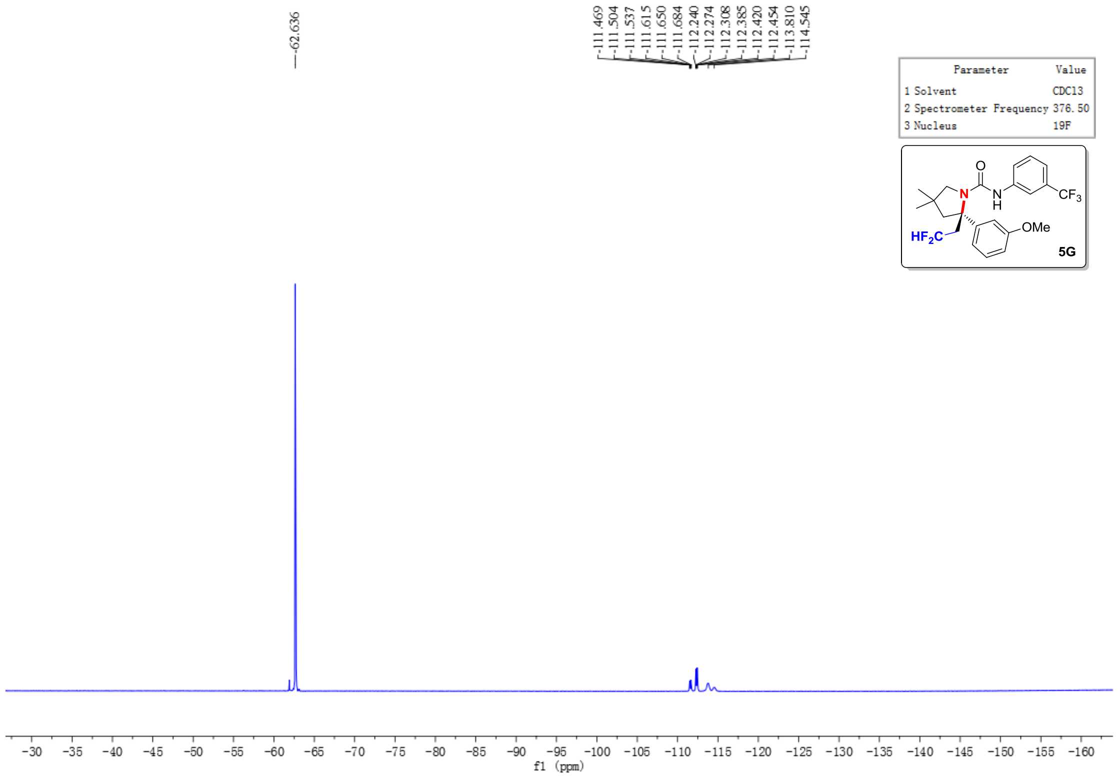

Supplementary Figure 125. ^{19}F NMR of **5G**

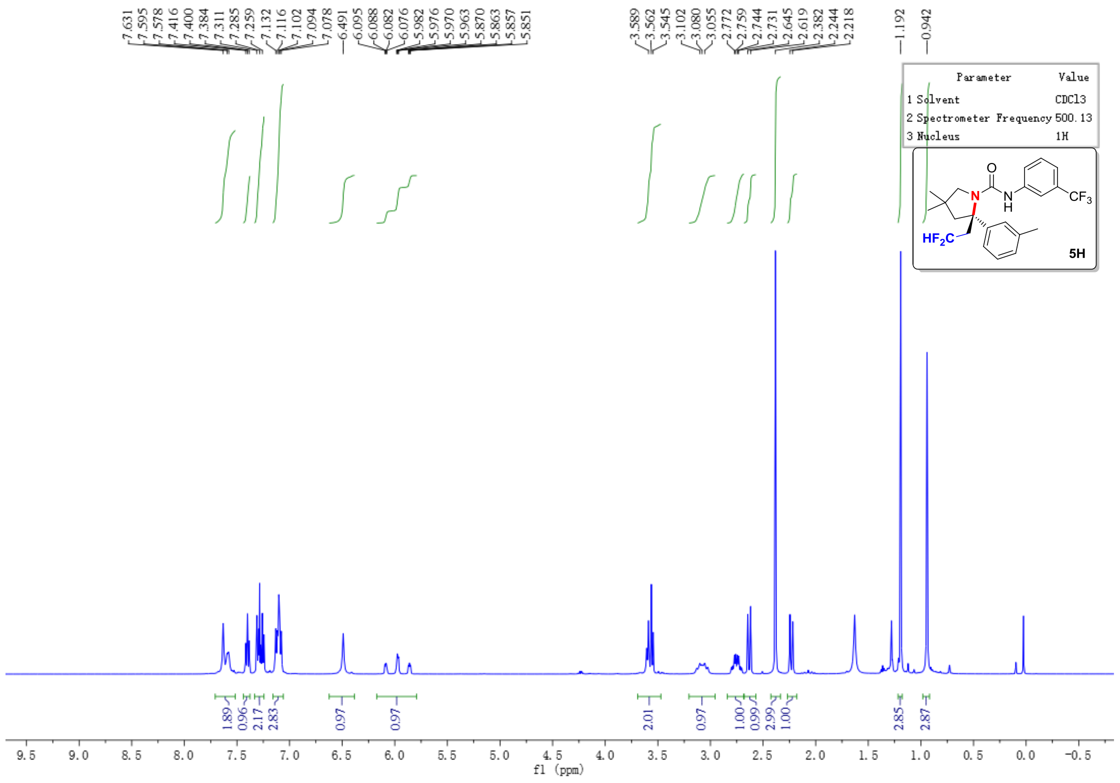

Supplementary Figure 126. ^{1}H NMR of **5H**

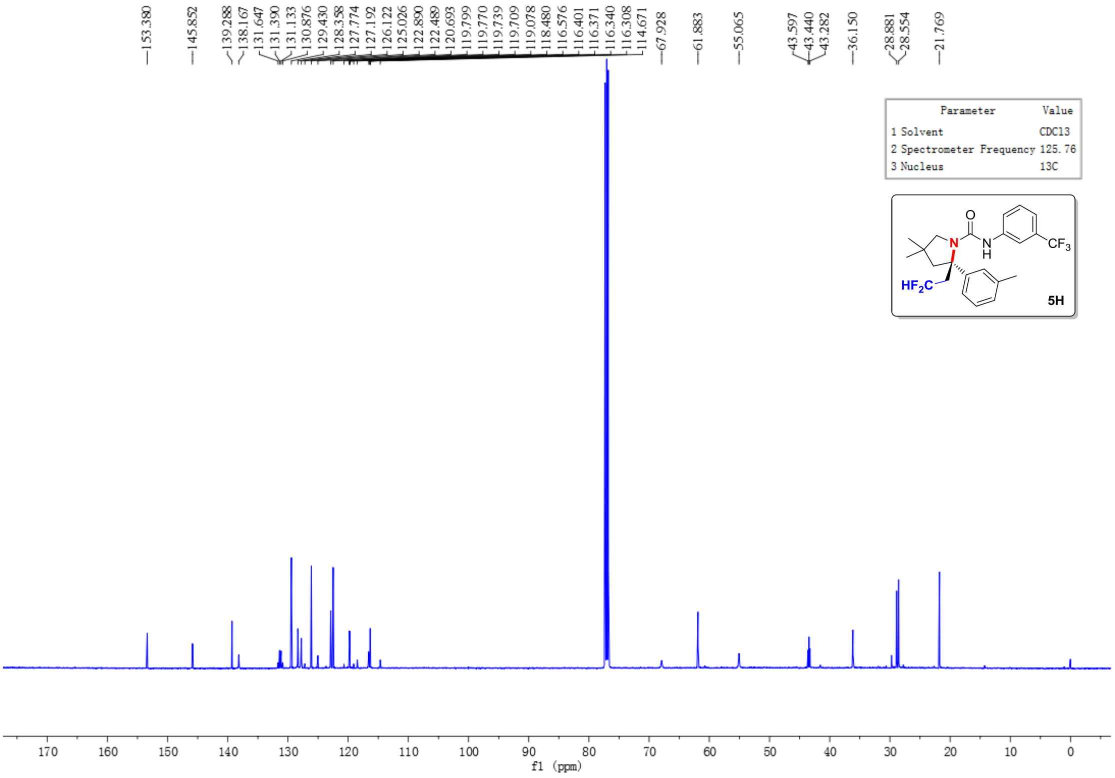

Supplementary Figure 127. ^{13}C NMR of **5H**

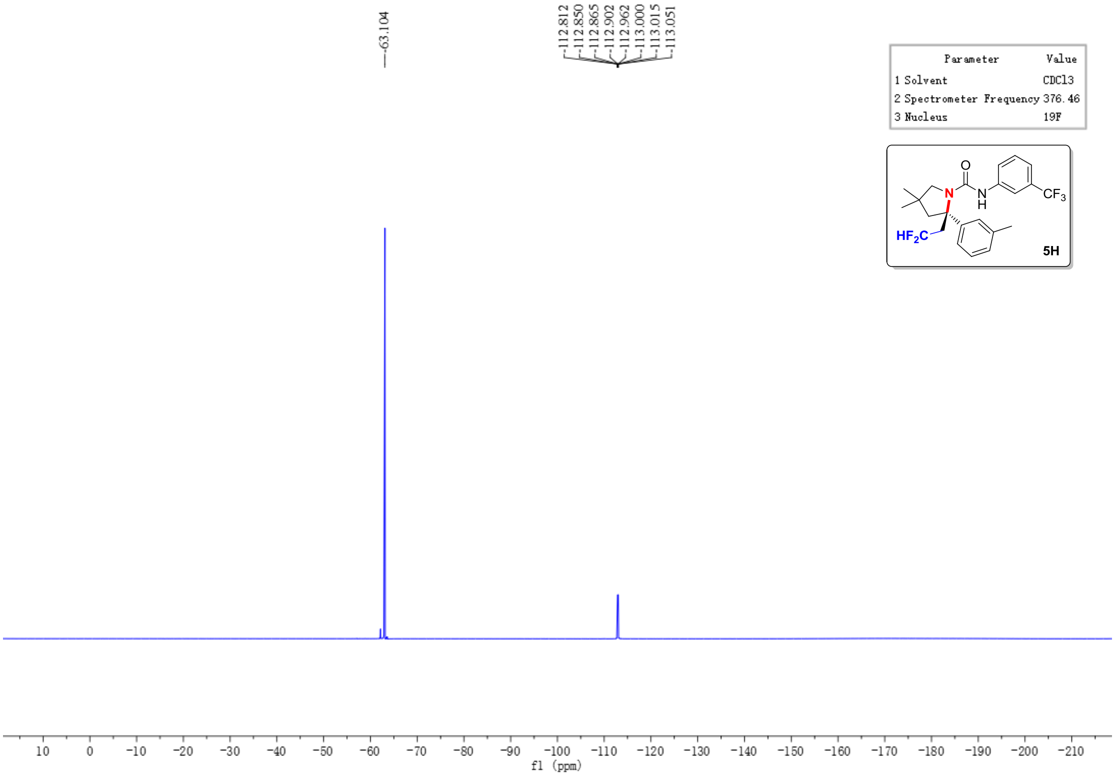

Supplementary Figure 128. ^{19}F NMR of **5H**

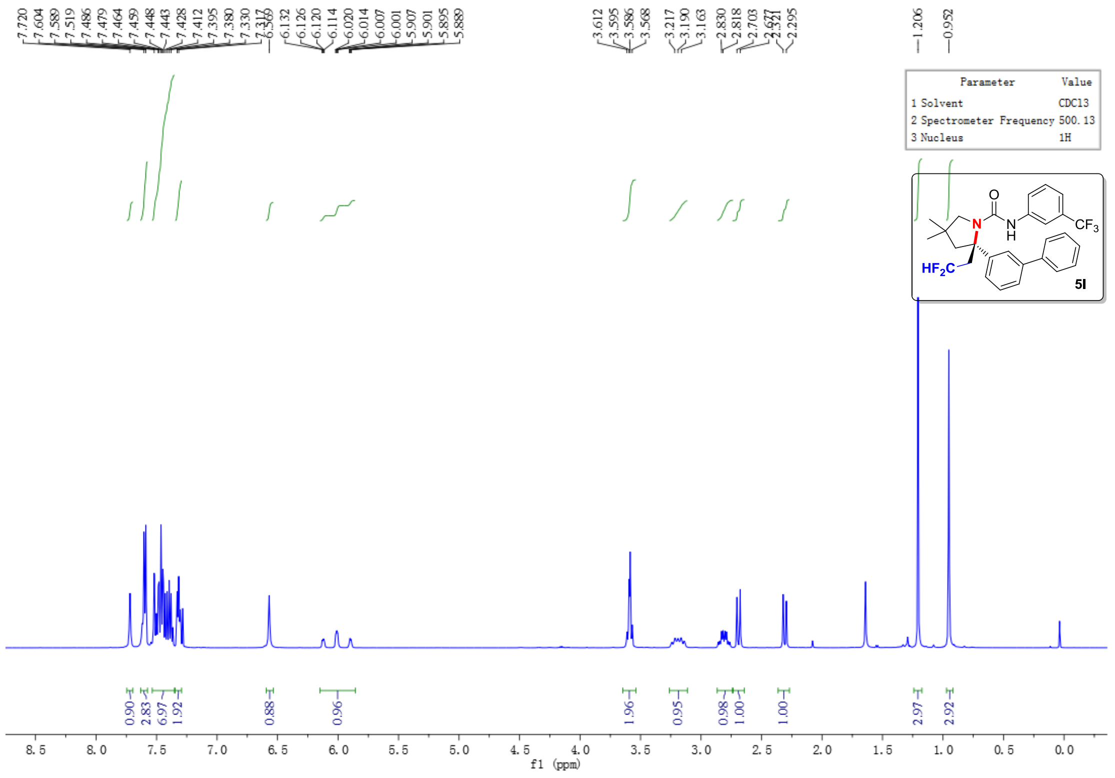

Supplementary Figure 129. ^{1}H NMR of **5l**

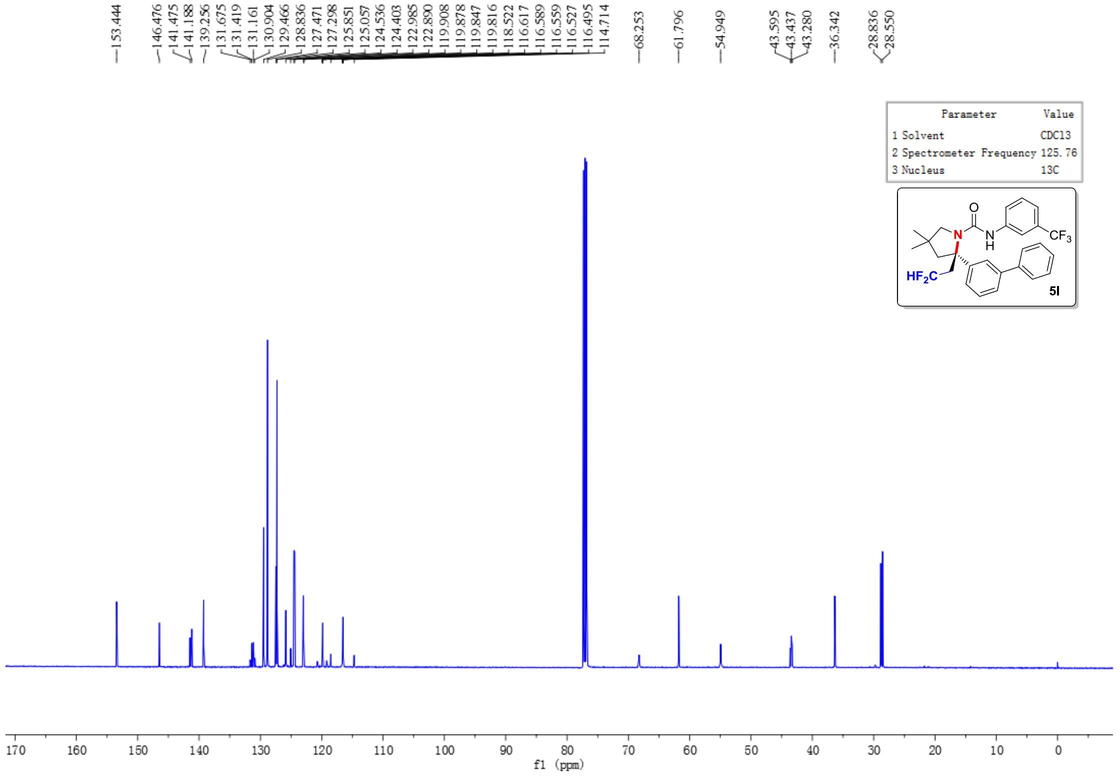

Supplementary Figure 130. ^{13}C NMR of **5l**

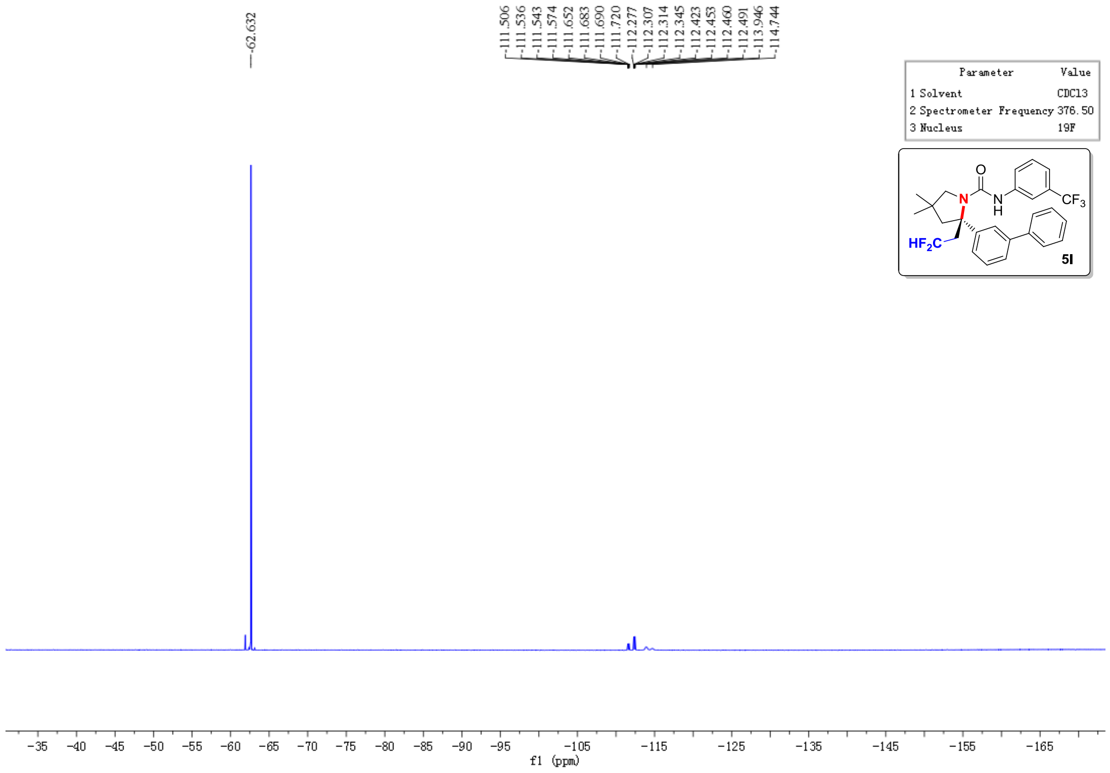

Supplementary Figure 131. ^{19}F NMR of **5I**

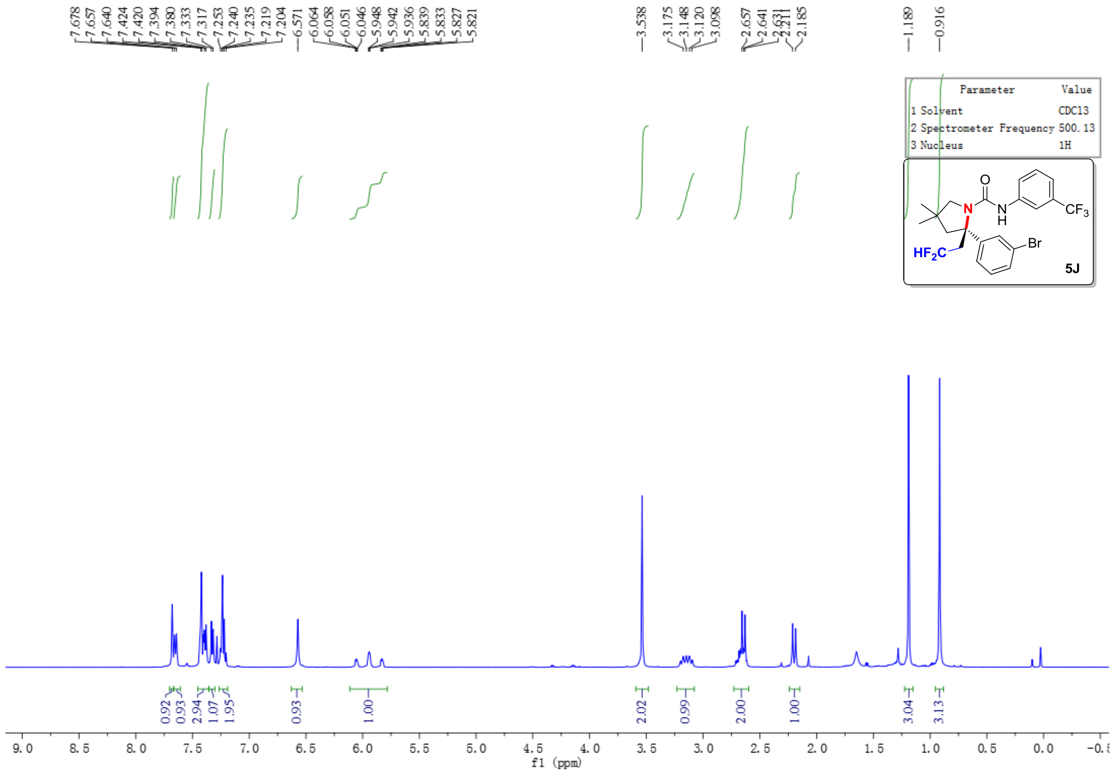

Supplementary Figure 132. ^{1}H NMR of **5J**

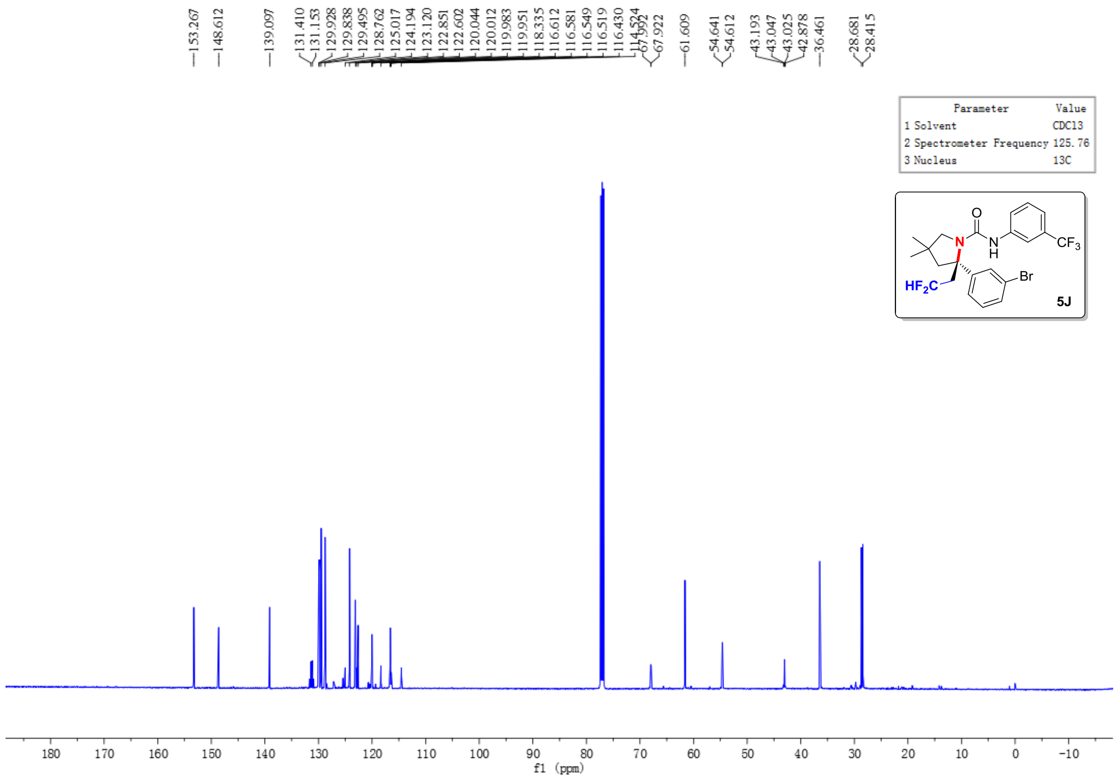

Supplementary Figure 133. ^{13}C NMR of **5J**

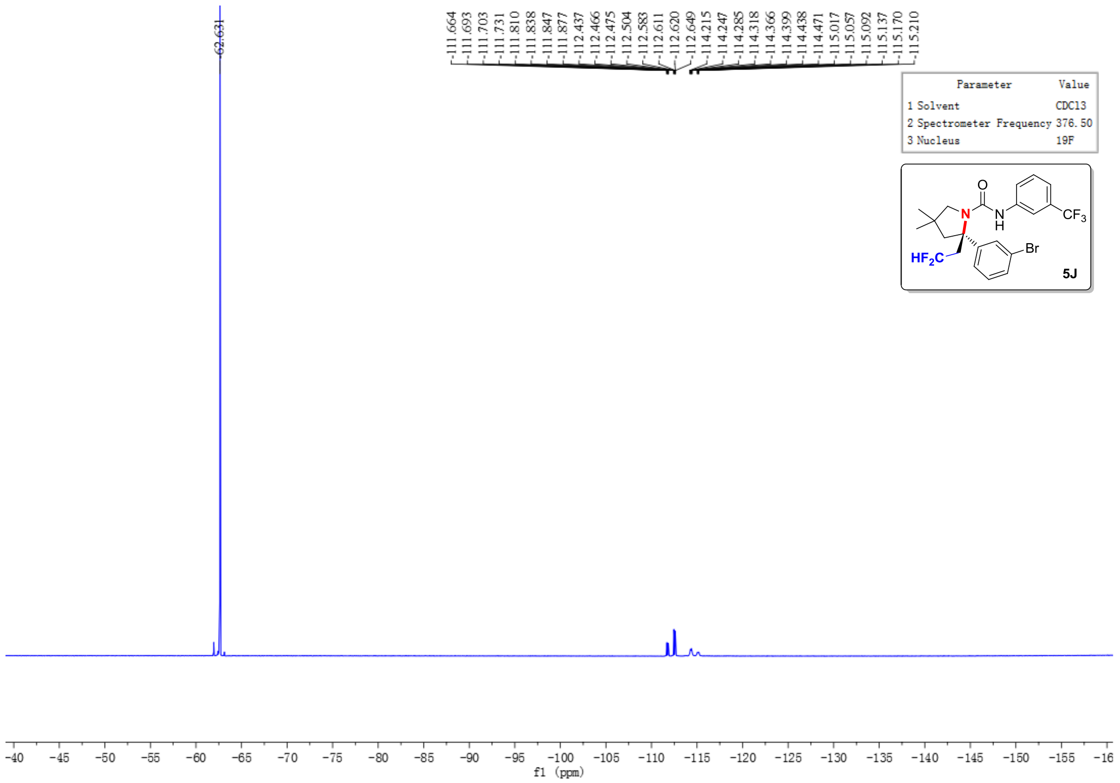

Supplementary Figure 134. ^{19}F NMR of **5J**

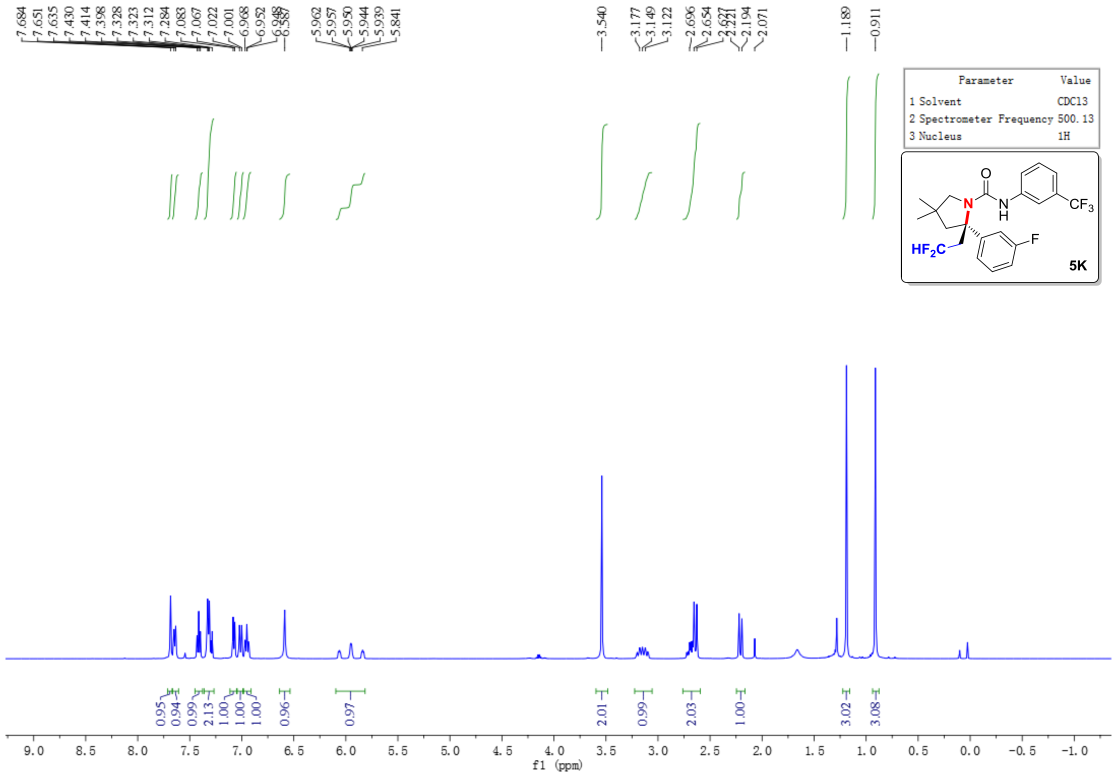

Supplementary Figure 135. ^{1}H NMR of **5K**

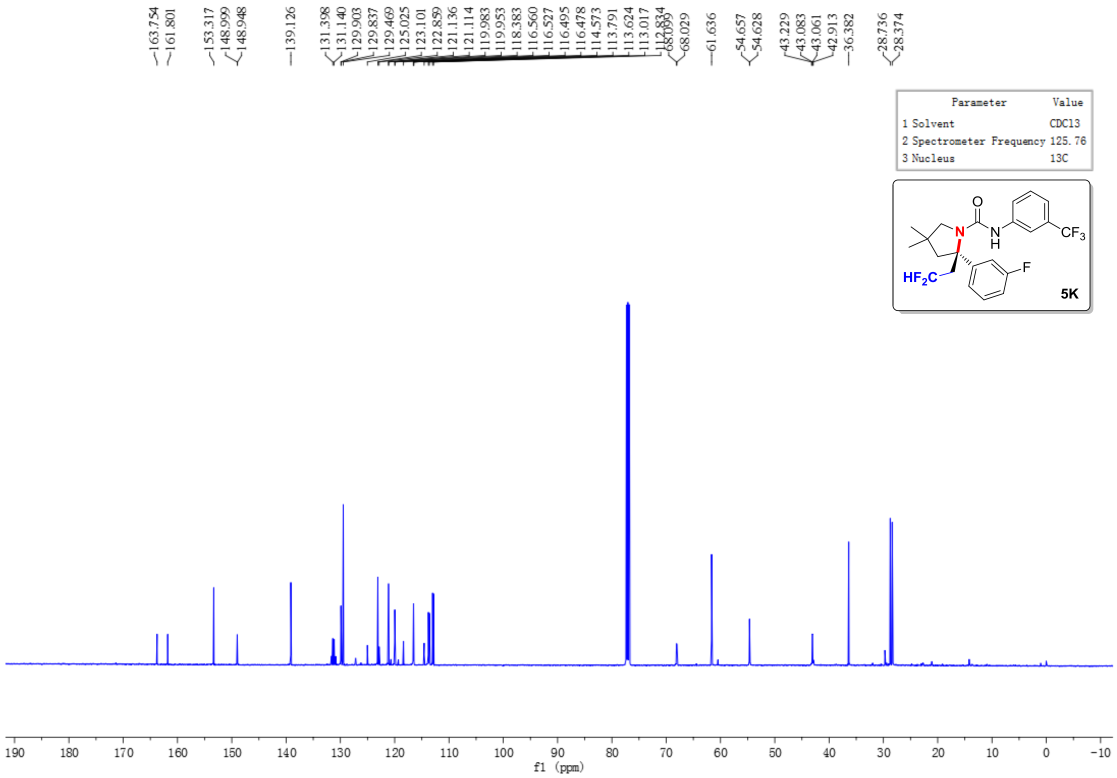

Supplementary Figure 136. ^{13}C NMR of **5K**

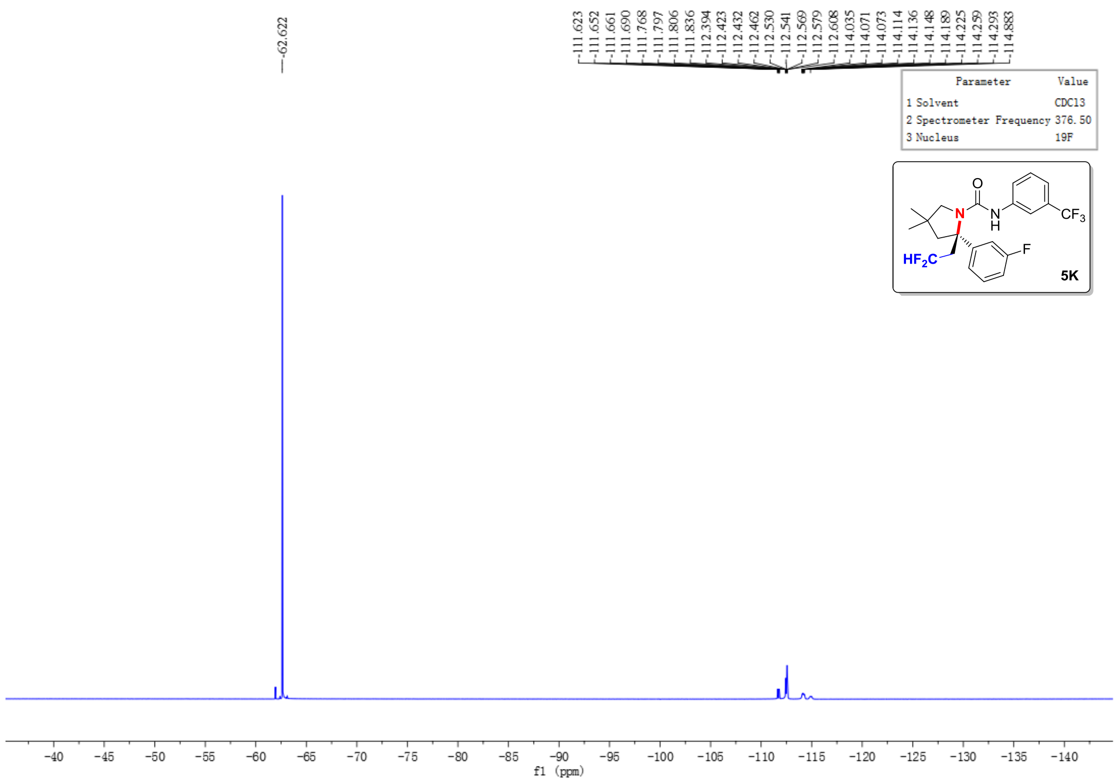

Supplementary Figure 137. ^{19}F NMR of **5K**

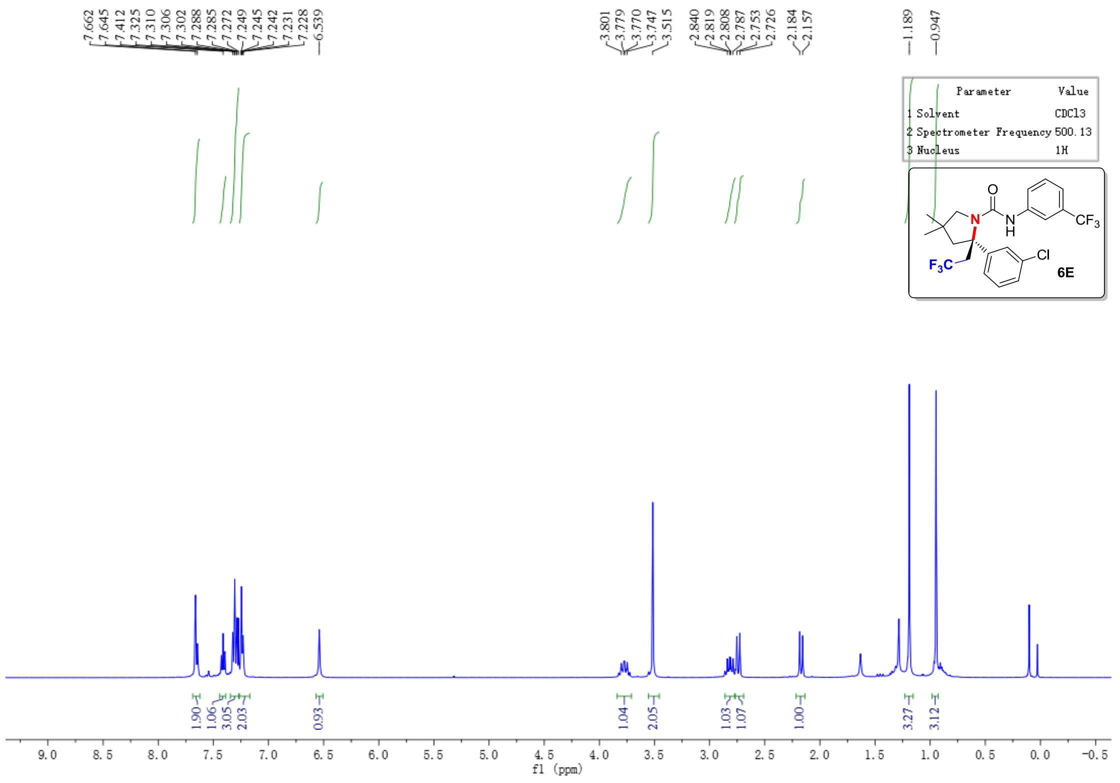

Supplementary Figure 138. ^{1}H NMR of **6E**

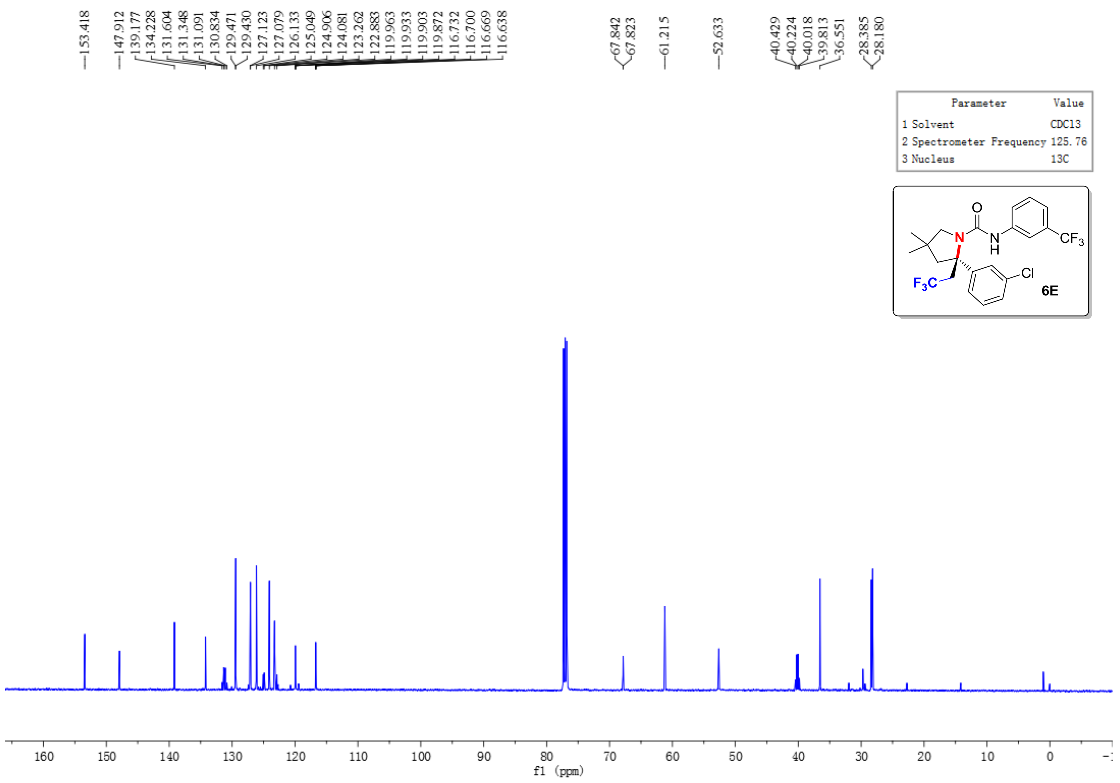

Supplementary Figure 139. ^{13}C NMR of **6E**

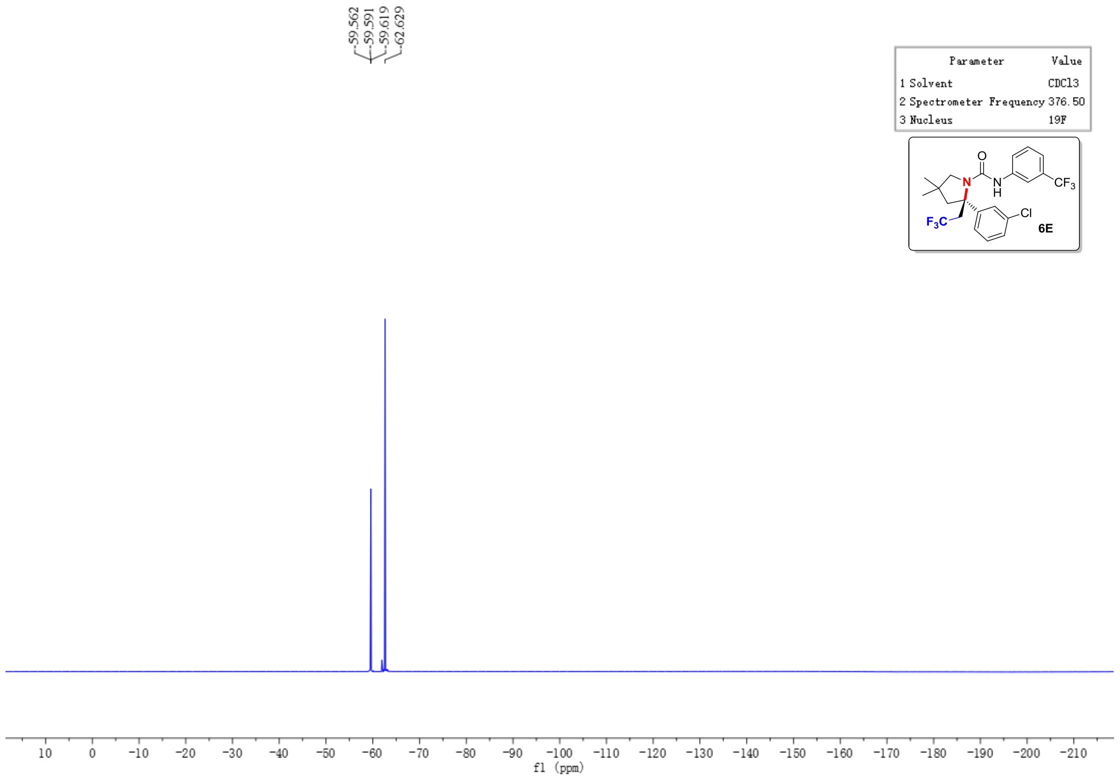

Supplementary Figure 140. ^{19}F NMR of **6E**

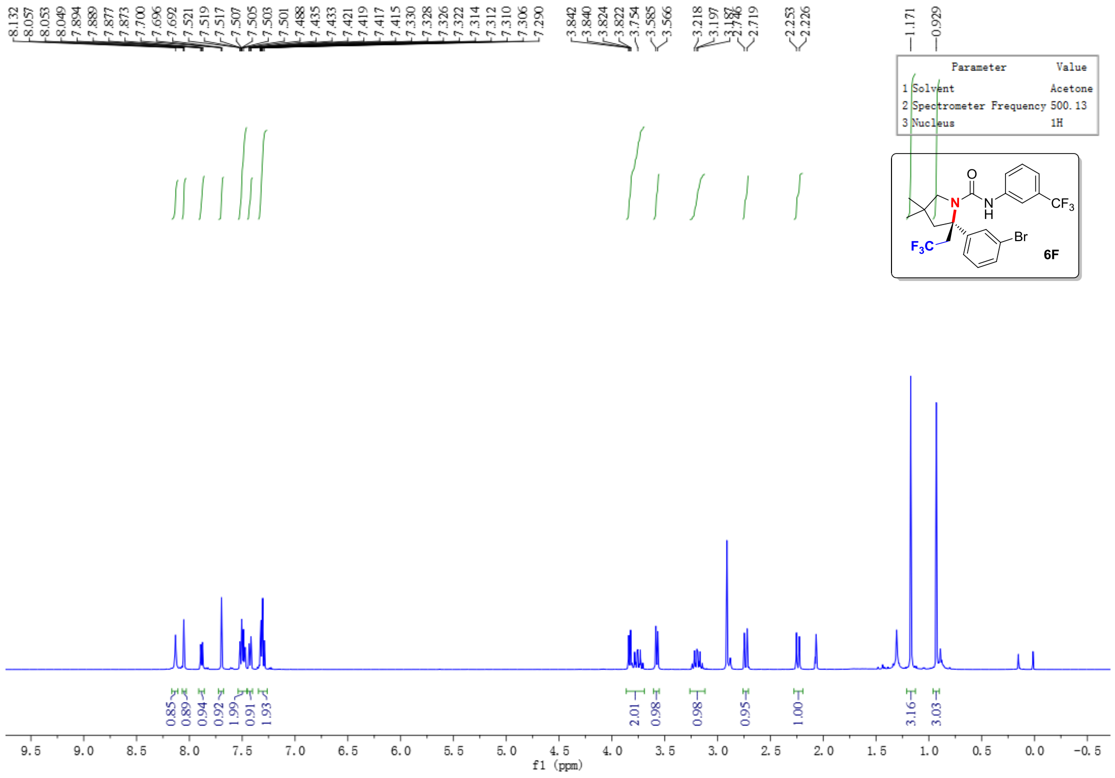

Supplementary Figure 141. ^{1}H NMR of **6F**

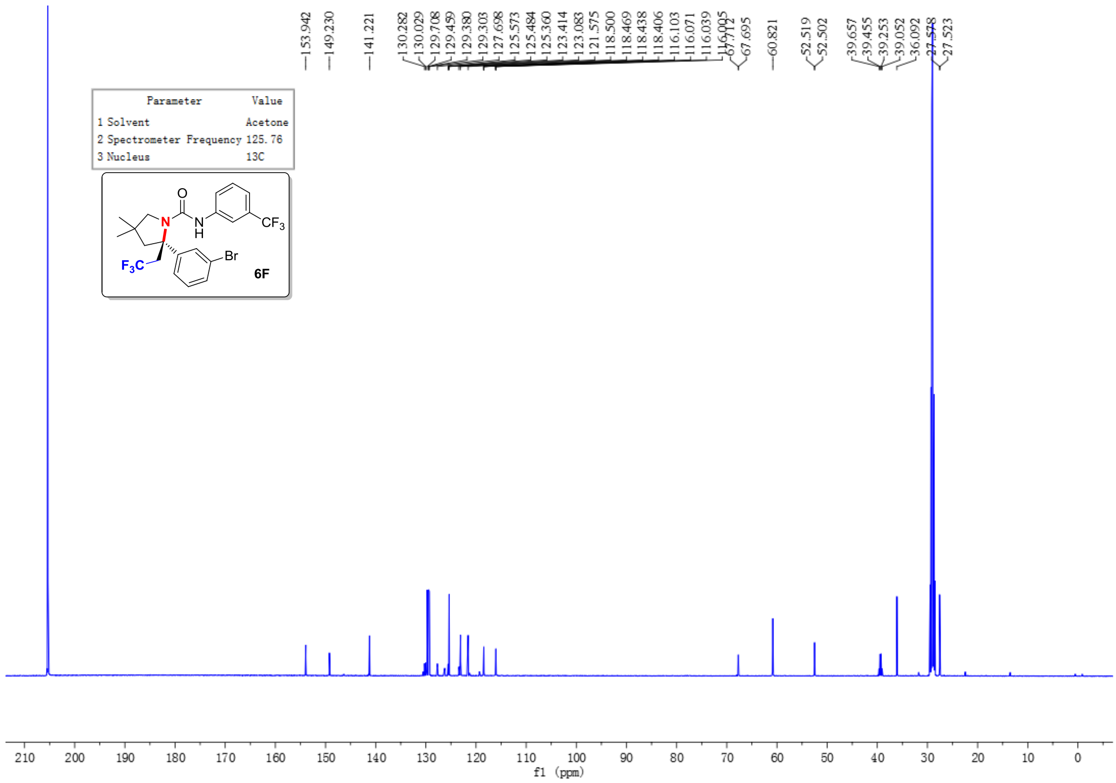

Supplementary Figure 142. ^{13}C NMR of **6F**

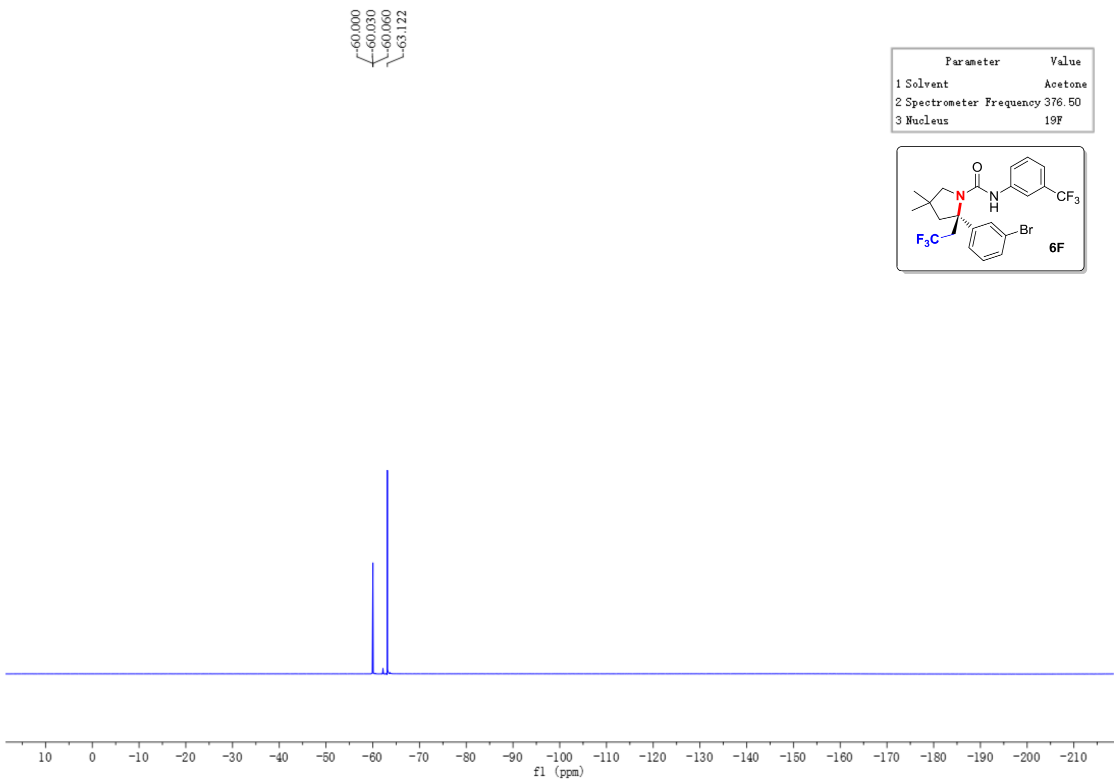

Supplementary Figure 143. ^{19}F NMR of **6F**

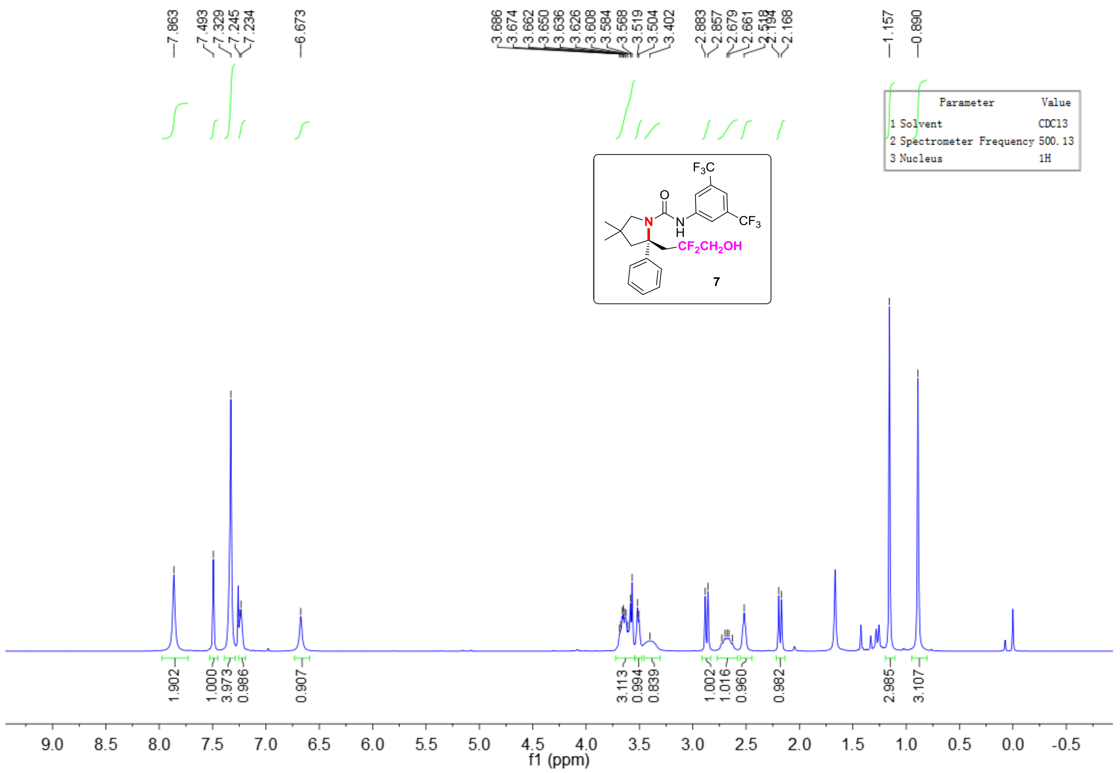

Supplementary Figure 144. ^{1}H NMR of **7**

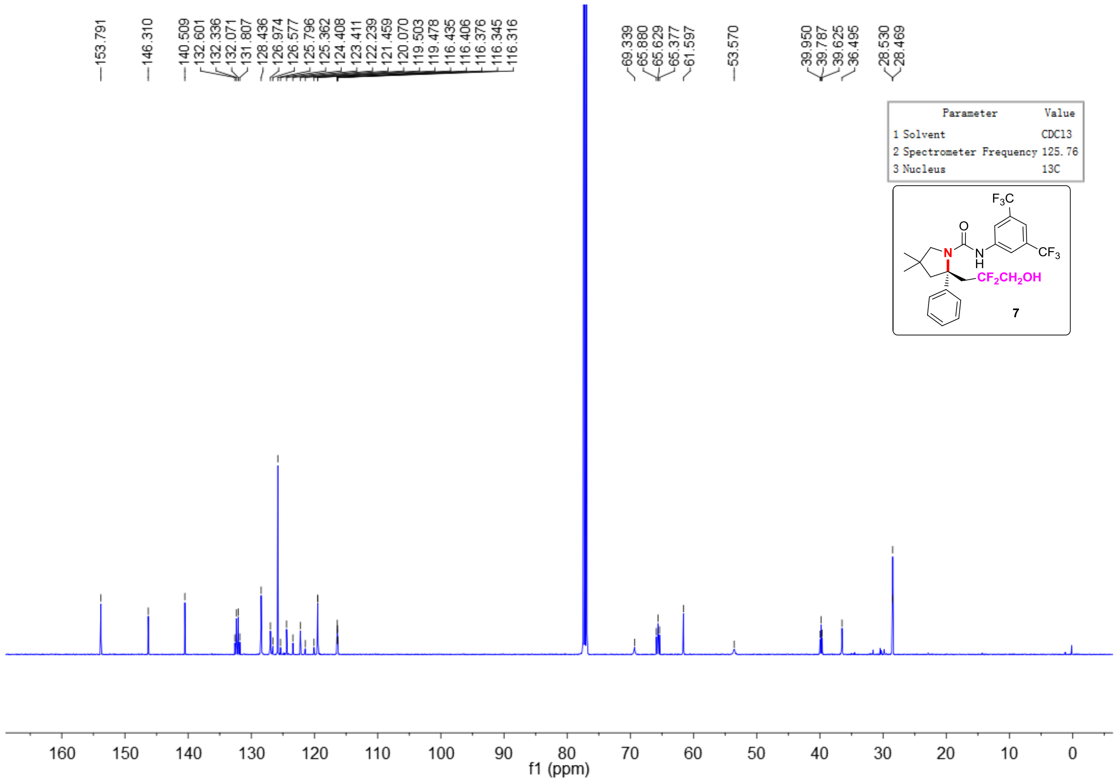

Supplementary Figure 145. ^{13}C NMR of **7**

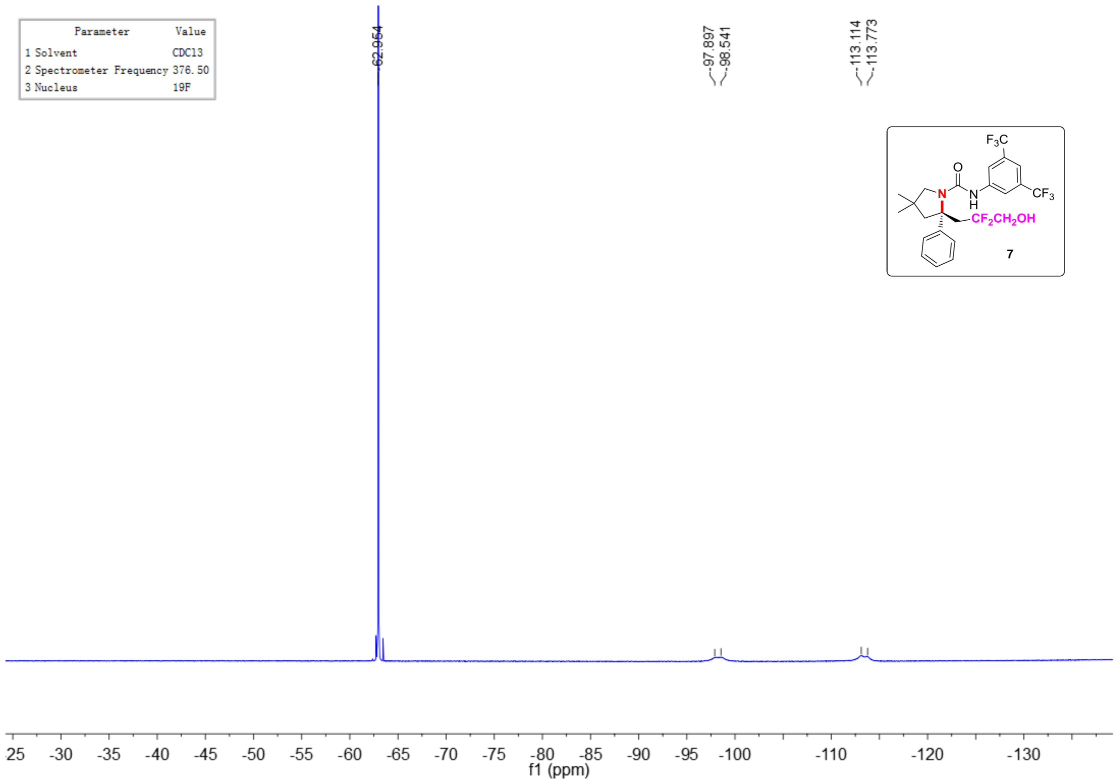

Supplementary Figure 146. ^{19}F NMR of **7**

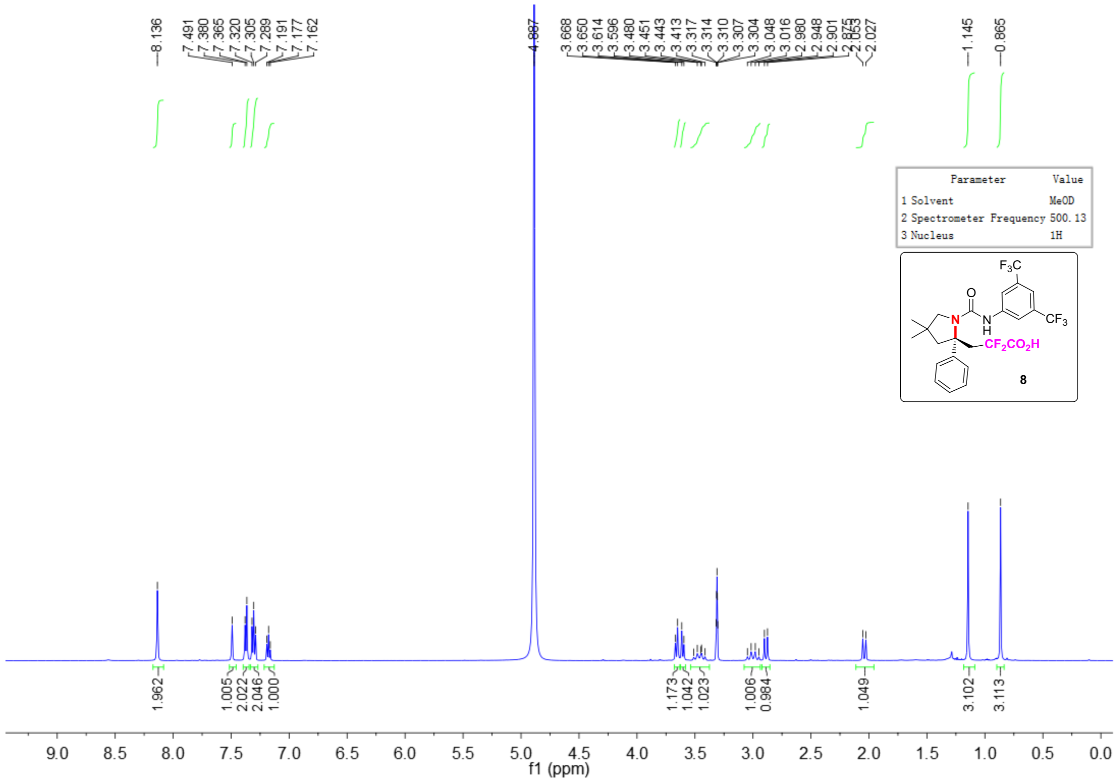

Supplementary Figure 147. ^{1}H NMR of **8**

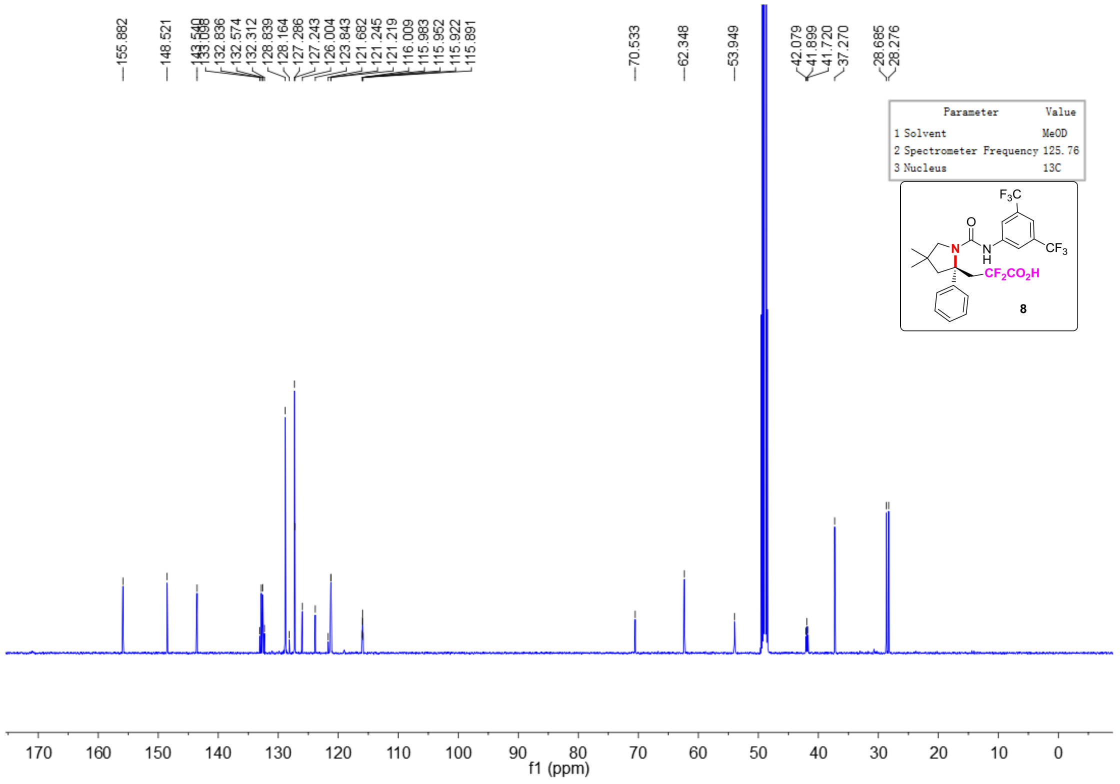

Supplementary Figure 148. ^{13}C NMR of **8**

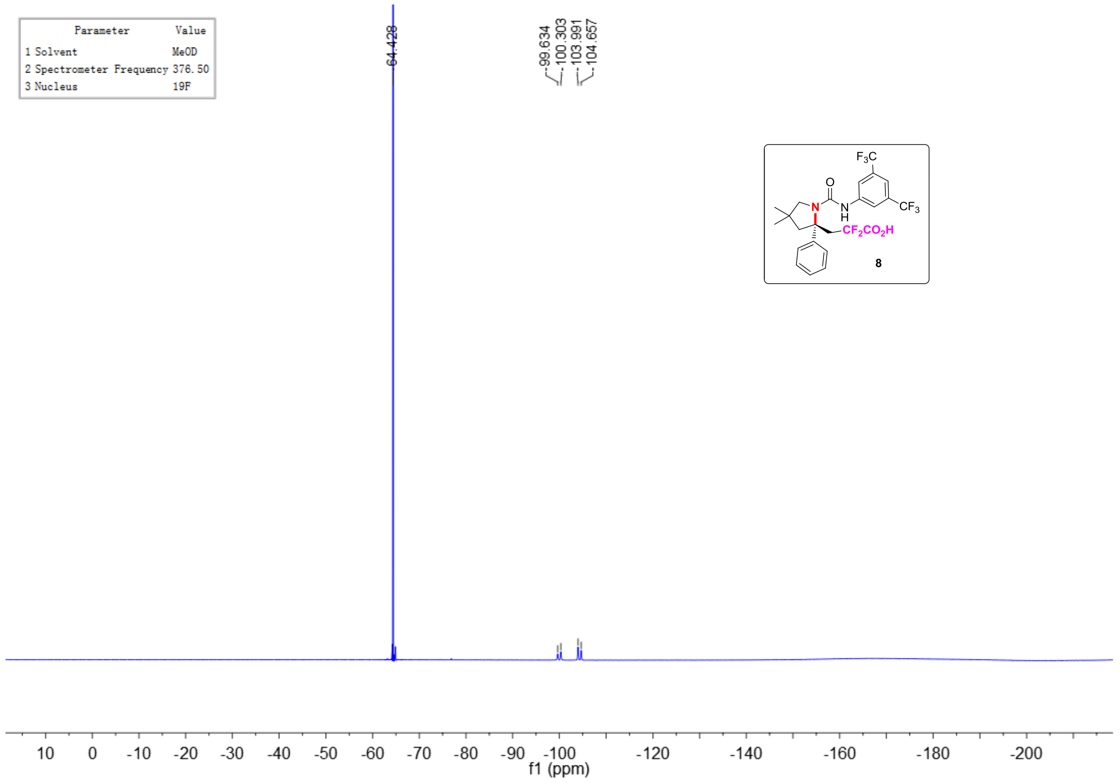

Supplementary Figure 149. ^{19}F NMR of **8**

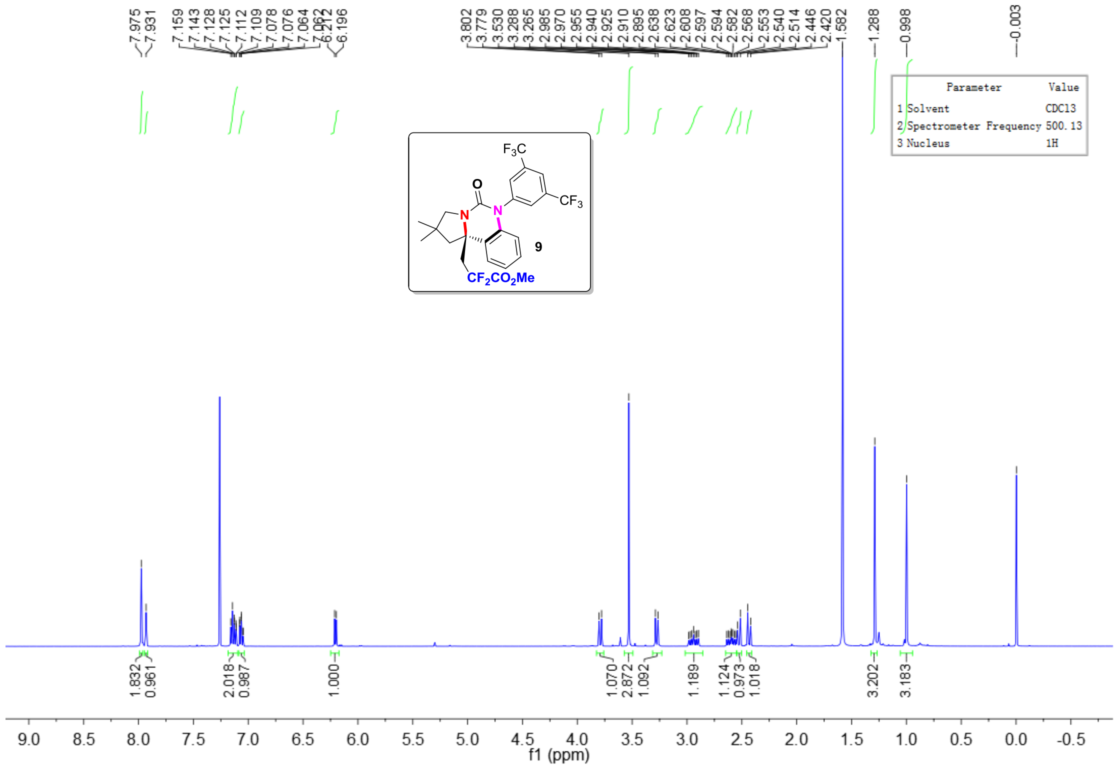

Supplementary Figure 150. ^{1}H NMR of **9**

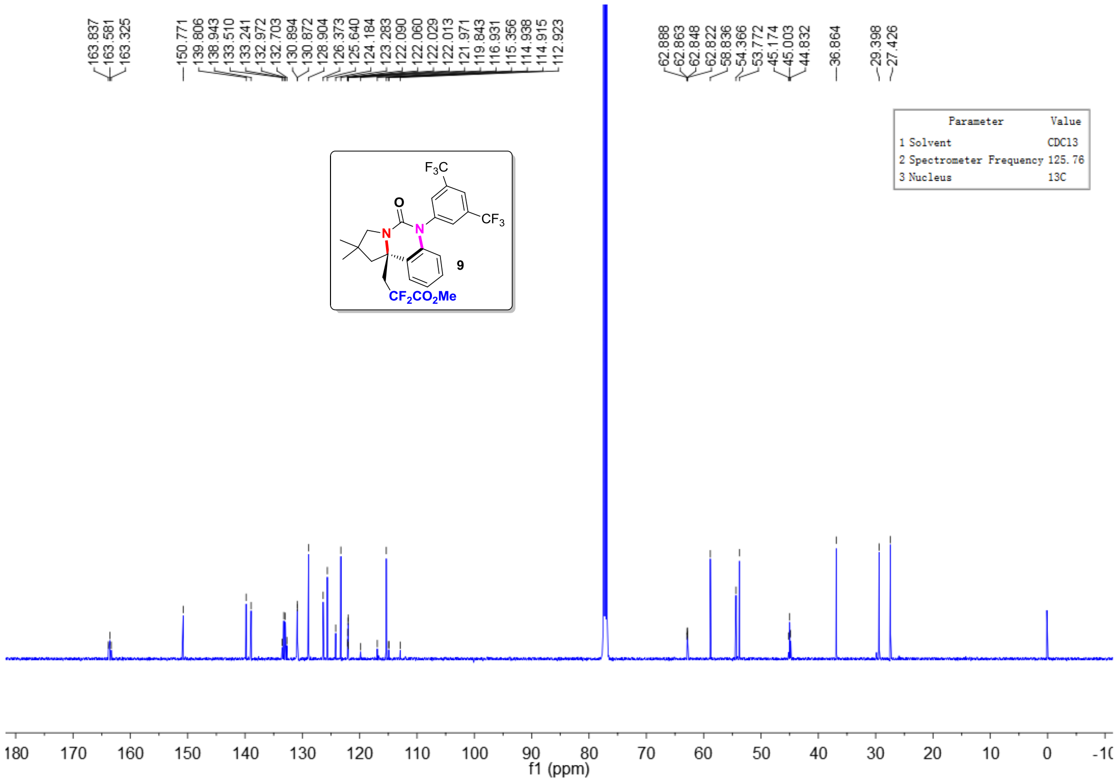

Supplementary Figure 151. ^{13}C NMR of **9**

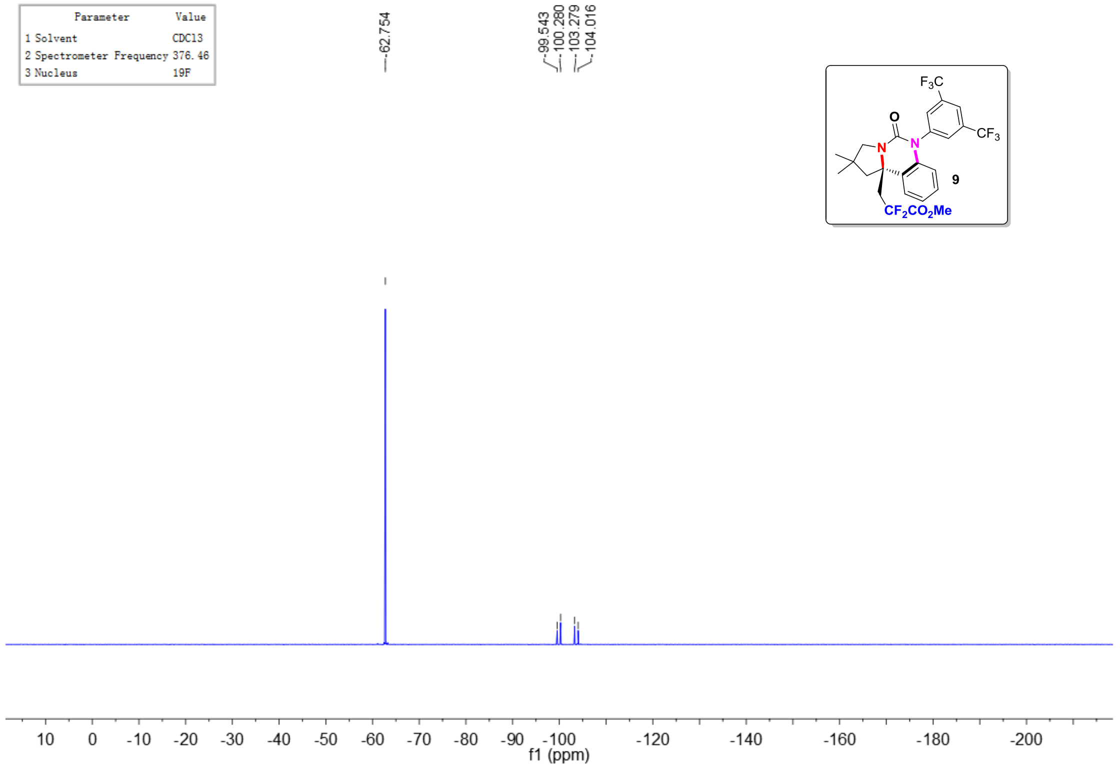

Supplementary Figure 152. ^{19}F NMR of **9**

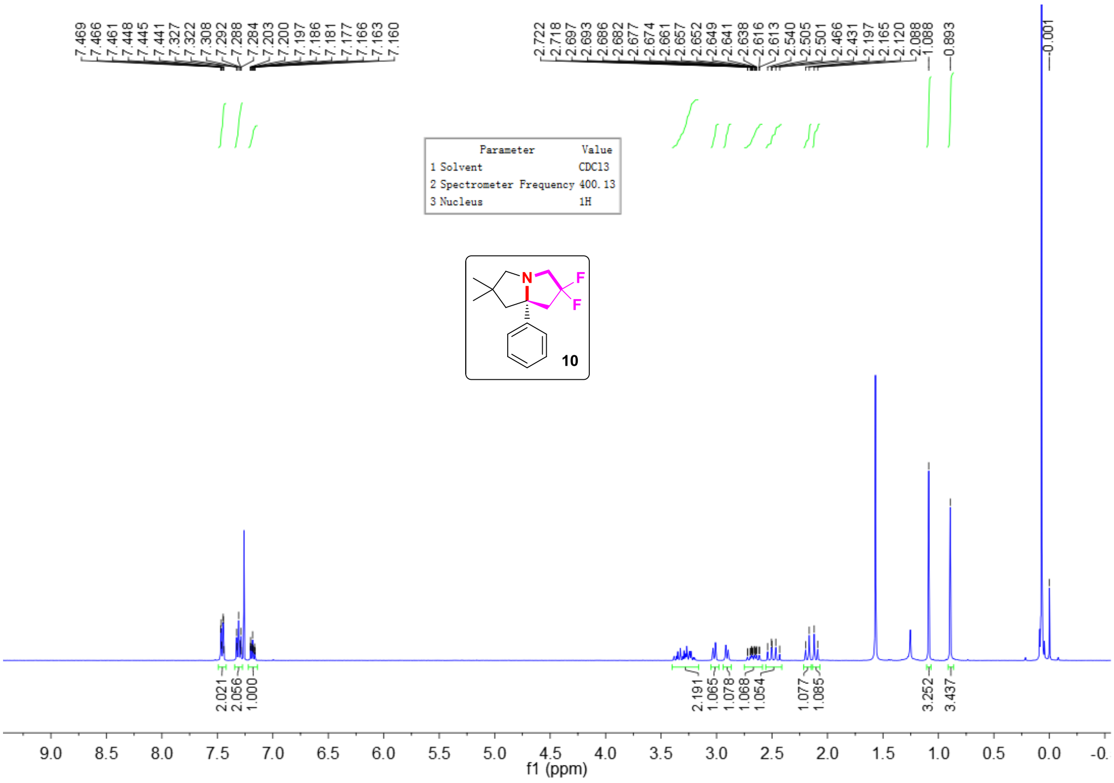

Supplementary Figure 153. ^{1}H NMR of **10**

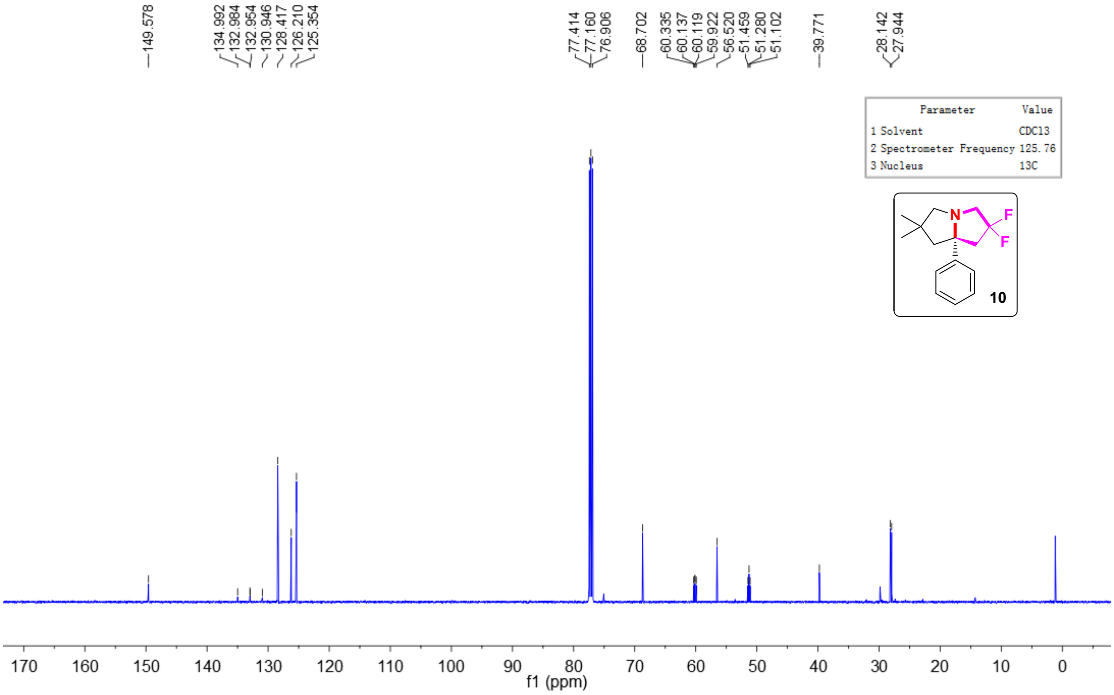

Supplementary Figure 154. ^{13}C NMR of **10**

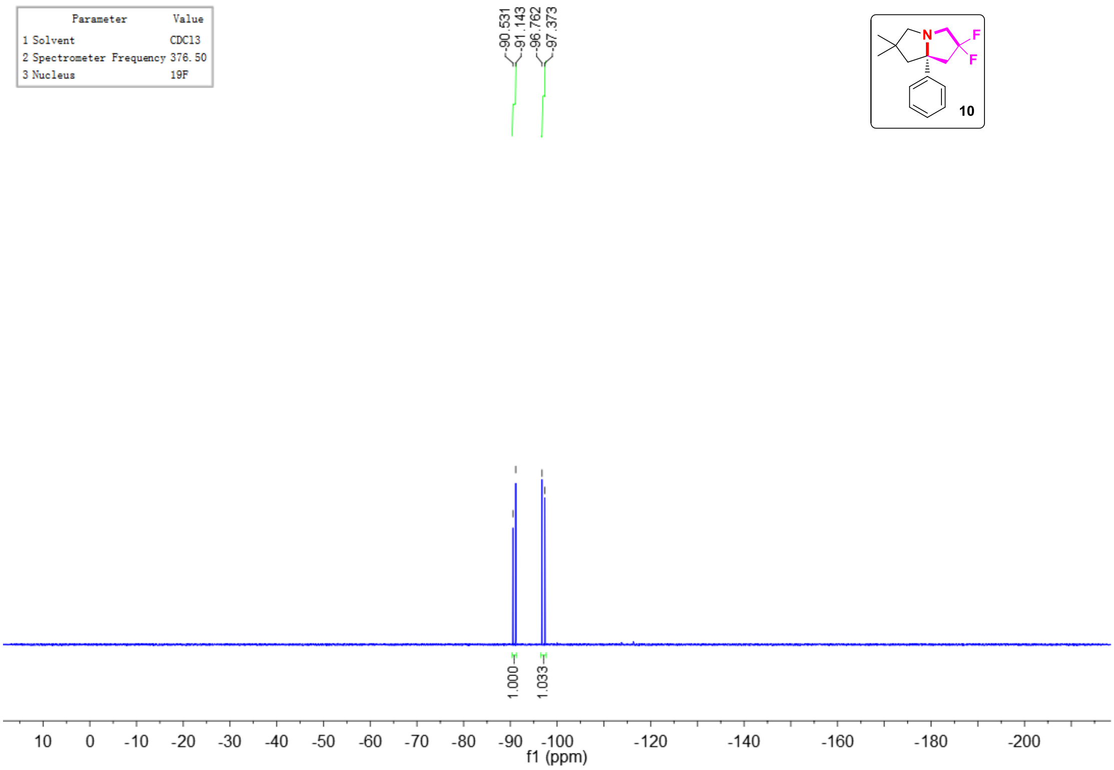

Supplementary Figure 155. ^{19}F NMR of **10**

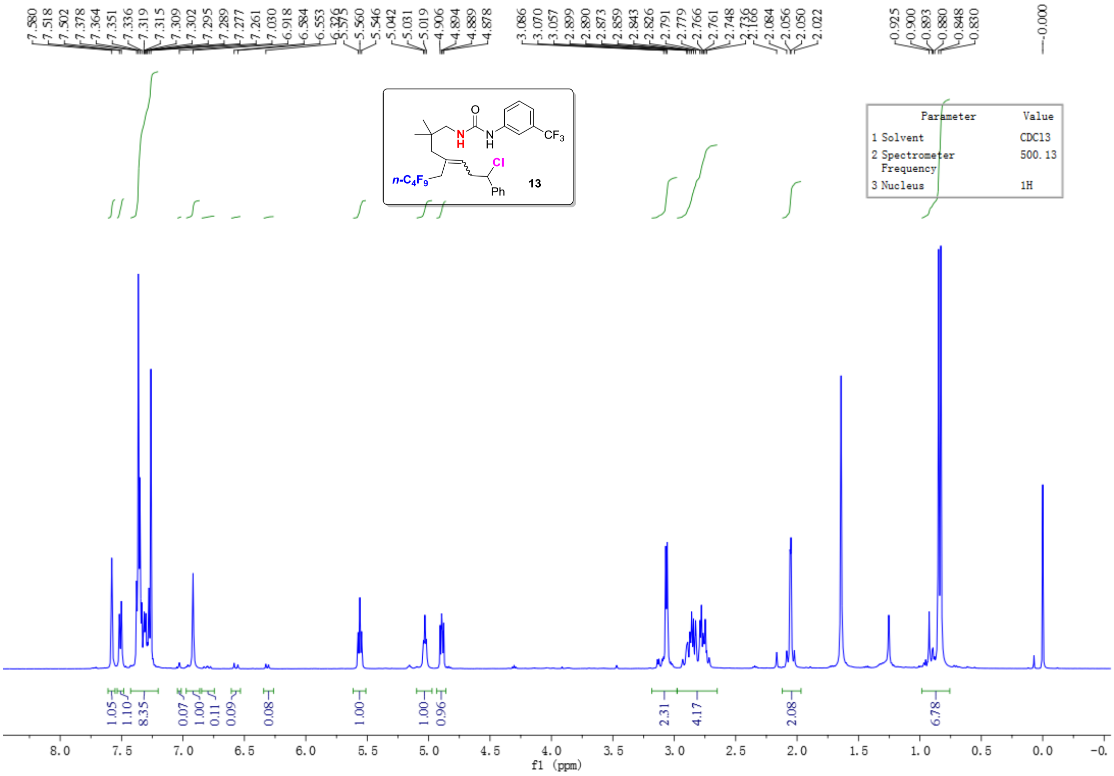

Supplementary Figure 156. ^{1}H NMR of **13**

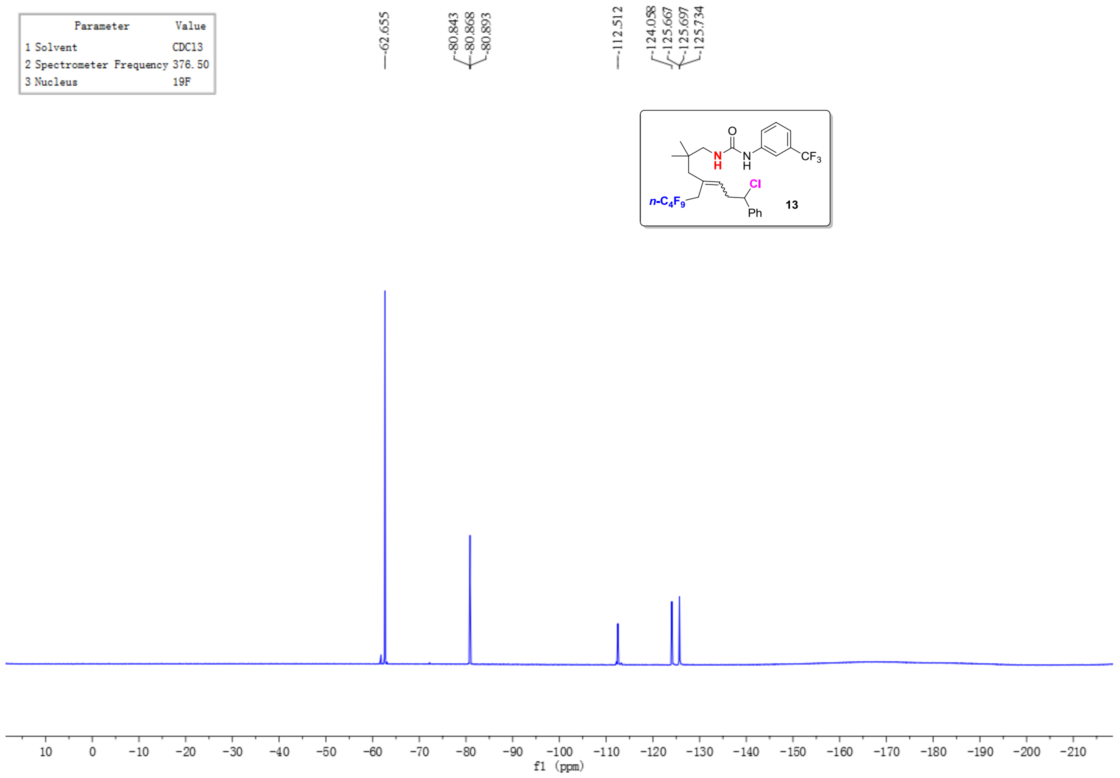

Supplementary Figure 157. ^{19}F NMR of **13**

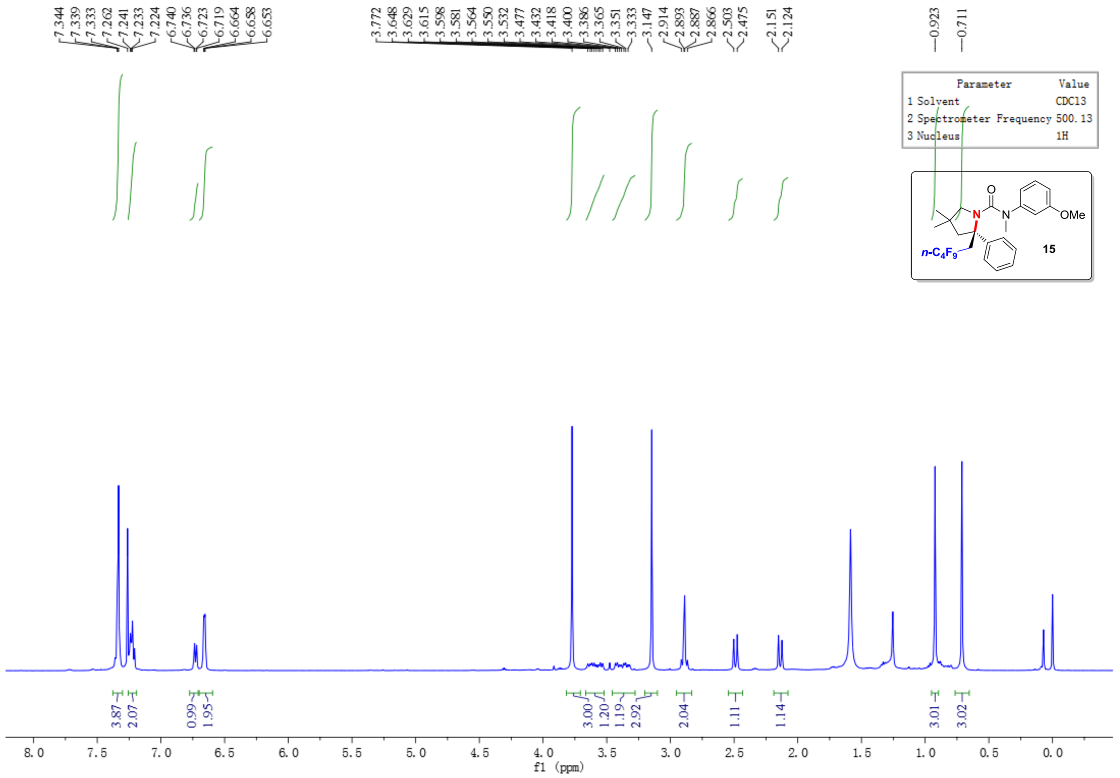

Supplementary Figure 158. ^{1}H NMR of **15**

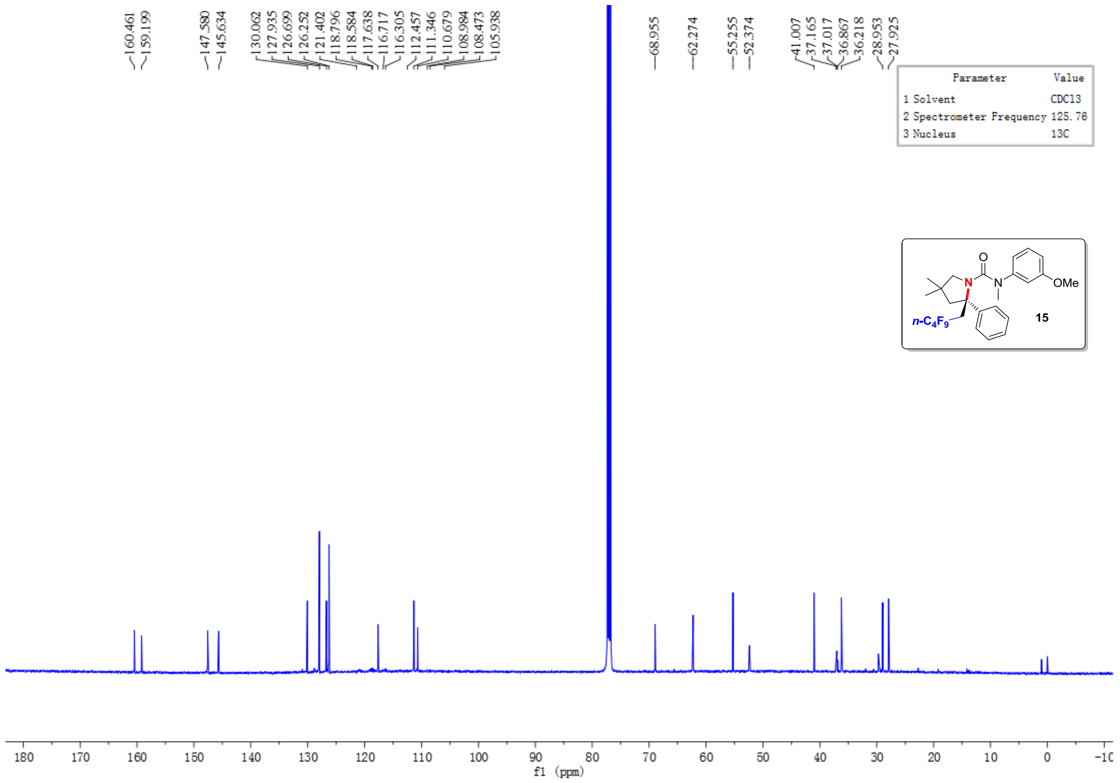

Supplementary Figure 159. ^{13}C NMR of **15**

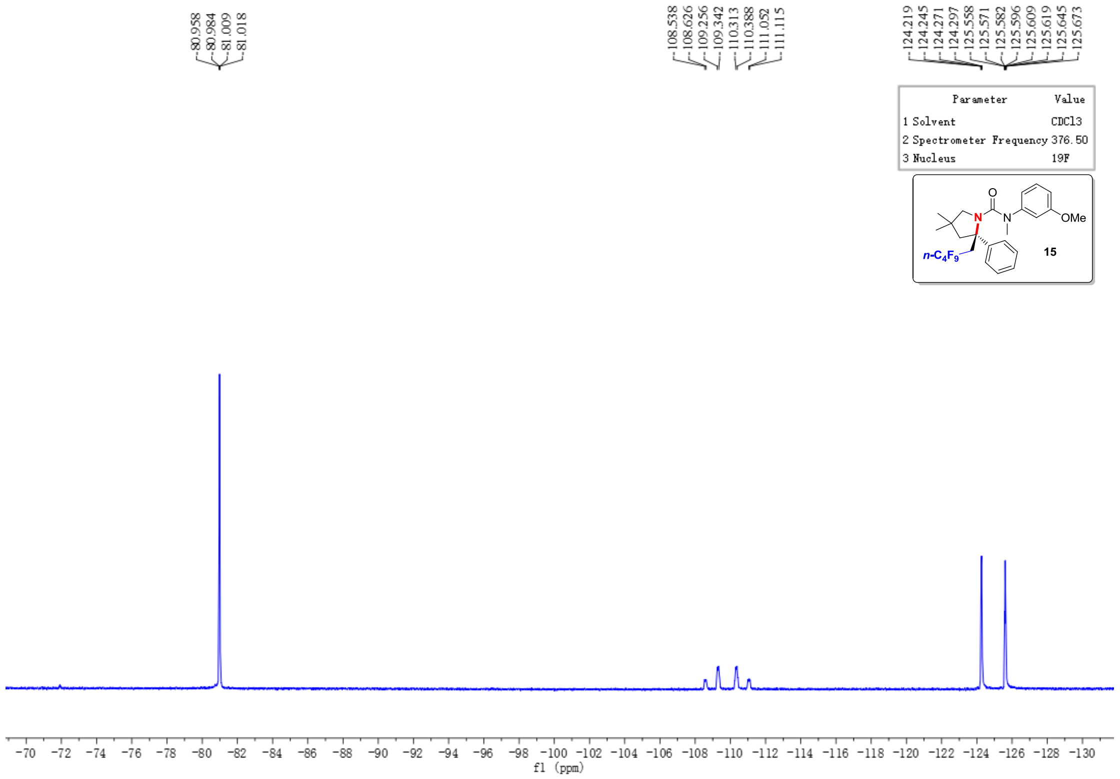

Supplementary Figure 160. ^{19}F NMR of **15**

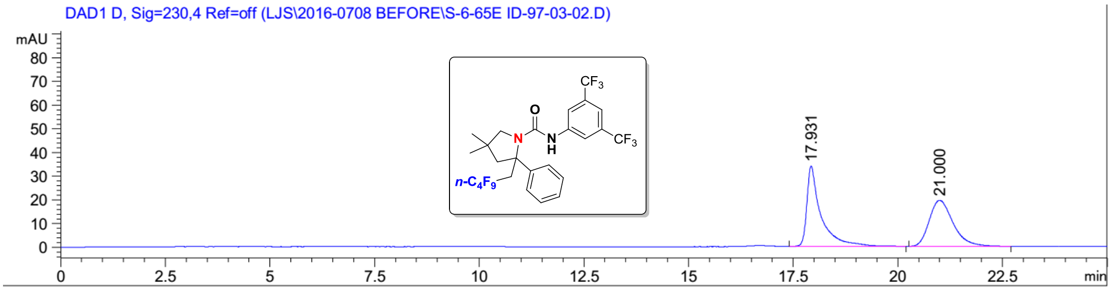

Signal 3: DAD1 D, Sig=230,4 Ref=off

Peak #	RetTime [min]	Type	Width [min]	Area [mAU*s]	Height [mAU]	Area %
1	17.931	BB	0.3253	800.77112	33.93017	50.8798
2	21.000	BB	0.5998	773.07703	19.44638	49.1202

Totals : 1573.84814 53.37654

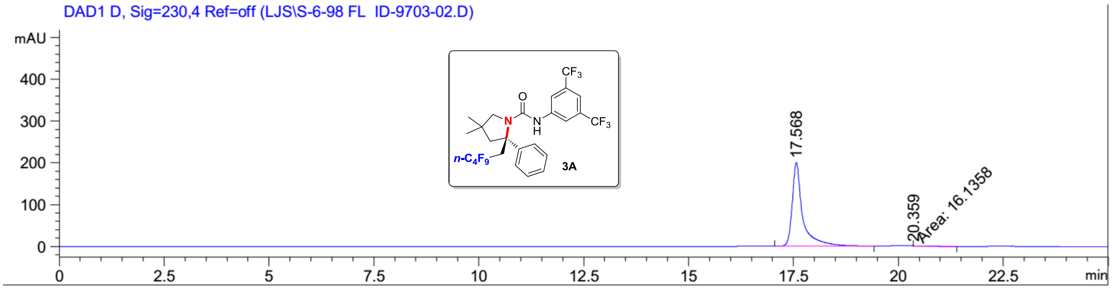

Signal 3: DAD1 D, Sig=230,4 Ref=off

Peak #	RetTime [min]	Type	Width [min]	Area [mAU*s]	Height [mAU]	Area %
1	17.568	BB	0.2478	3493.37964	200.00197	99.5402
2	20.359	MM	0.3637	16.13583	7.39386e-1	0.4598

Totals : 3509.51547 200.74135

Supplementary Figure 161. HPLC traces for racemic and chiral product **3A**

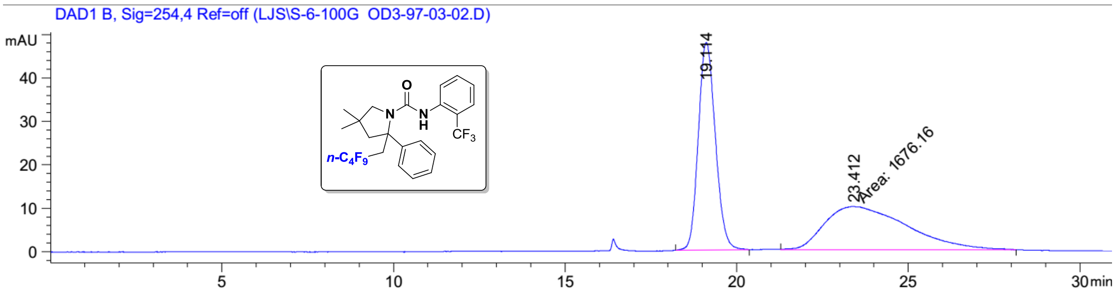

Signal 2: DAD1 B, Sig=254,4 Ref=off

Peak #	RetTime [min]	Type	Width [min]	Area [mAU*s]	Height [mAU]	Area %
1	19.114	BB	0.5306	1648.22839	47.75891	49.5799
2	23.412	MM	2.7968	1676.16284	9.98865	50.4201

Totals : 3324.39124 57.74756

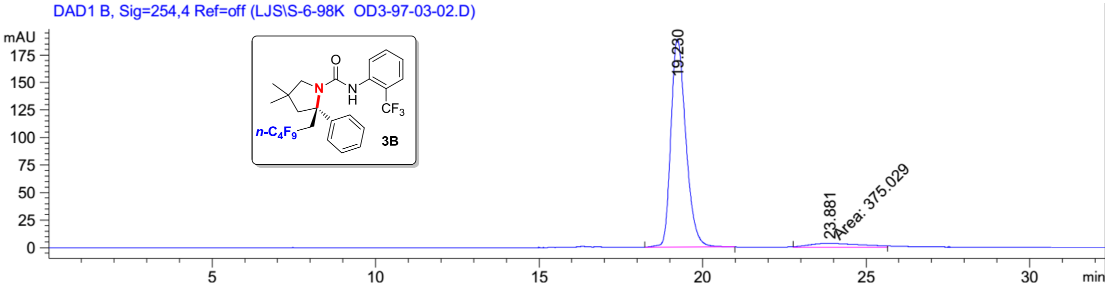

Signal 2: DAD1 B, Sig=254,4 Ref=off

Peak #	RetTime [min]	Type	Width [min]	Area [mAU*s]	Height [mAU]	Area %
1	19.230	BB	0.4894	6013.29492	188.90350	94.1295
2	23.881	MM	1.8333	375.02881	3.40935	5.8705

Totals : 6388.32373 192.31285

Supplementary Figure 162. HPLC traces for racemic and chiral product **3B**

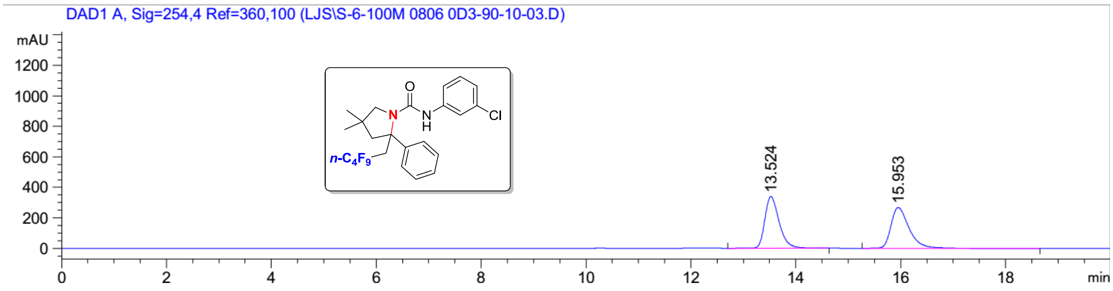

Signal 1: DAD1 A, Sig=254,4 Ref=360,100

Peak #	RetTime [min]	Type	Width [min]	Area [mAU*s]	Height [mAU]	Area %
1	13.524	BB	0.2900	6397.17969	339.18106	49.8668
2	15.953	BB	0.3665	6431.35938	267.52280	50.1332

Totals : 1.28285e4 606.70386

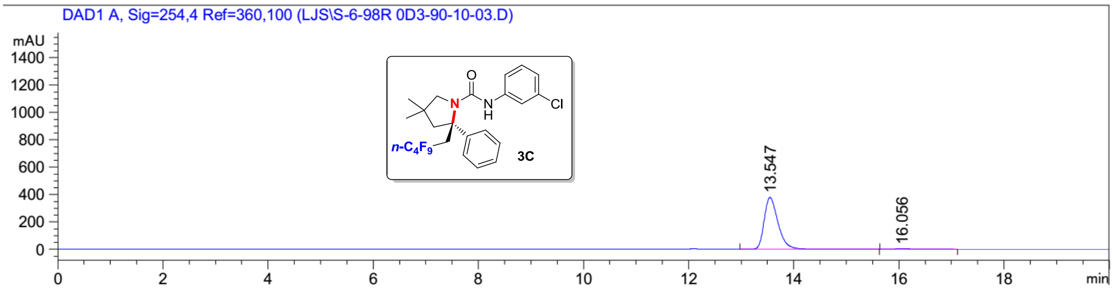

Signal 1: DAD1 A, Sig=254,4 Ref=360,100

Peak #	RetTime [min]	Type	Width [min]	Area [mAU*s]	Height [mAU]	Area %
1	13.547	BB	0.2829	6973.62842	378.52771	98.4699
2	16.056	BB	0.3663	108.36497	4.60833	1.5301

Totals : 7081.99339 383.13604

Supplementary Figure 163. HPLC traces for racemic and chiral product **3C**

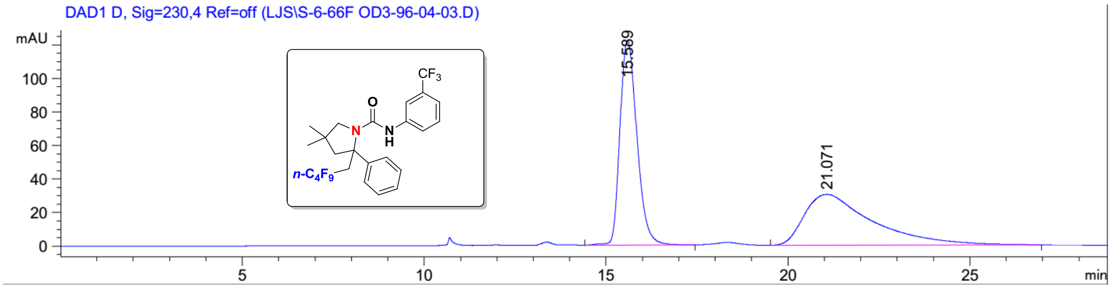

Signal 3: DAD1 D, Sig=230,4 Ref=off

Peak #	RetTime [min]	Type	Width [min]	Area [mAU*s]	Height [mAU]	Area %
1	15.589	BB	0.5109	3968.44995	122.19631	50.1053
2	21.071	BB	1.6767	3951.77026	30.31696	49.8947

Totals : 7920.22021 152.51327

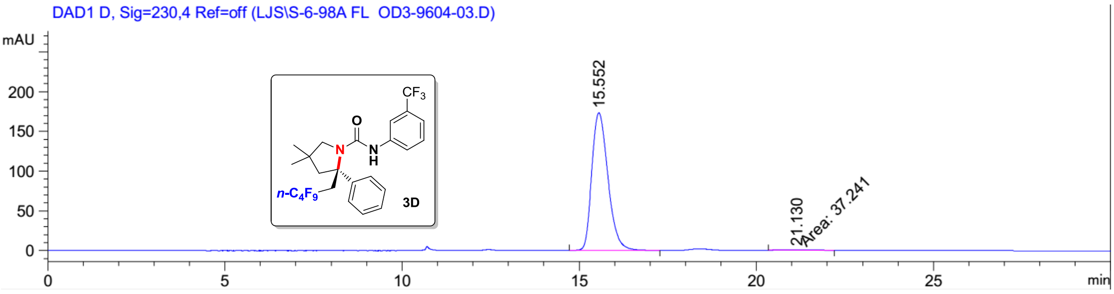

Signal 3: DAD1 D, Sig=230,4 Ref=off

Peak #	RetTime [min]	Type	Width [min]	Area [mAU*s]	Height [mAU]	Area %
1	15.552	BB	0.4950	5516.68066	173.45717	99.3295
2	21.130	MM	1.2500	37.24101	4.96531e-1	0.6705

Totals : 5553.92167 173.95370

Supplementary Figure 164. HPLC traces for racemic and chiral product **3D**

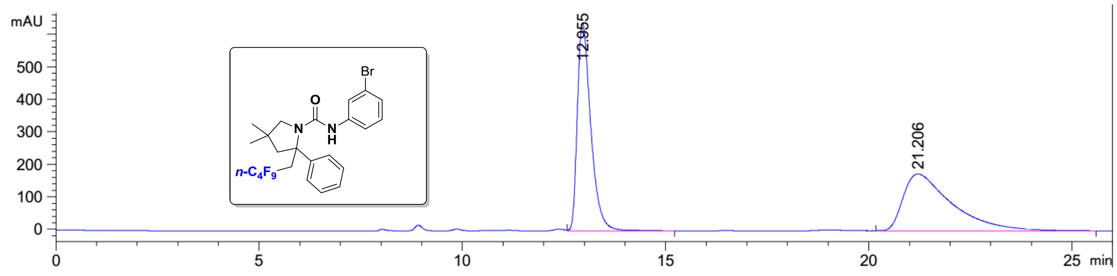

Signal 1: DAD1 A, Sig=214,4 Ref=off

Peak #	RetTime [min]	Type	Width [min]	Area [mAU*s]	Height [mAU]	Area %
1	12.955	VB	0.3501	1.47020e4	639.69098	50.2376
2	21.206	BB	1.1780	1.45630e4	174.79558	49.7624
Totals :				2.92650e4	814.48656	

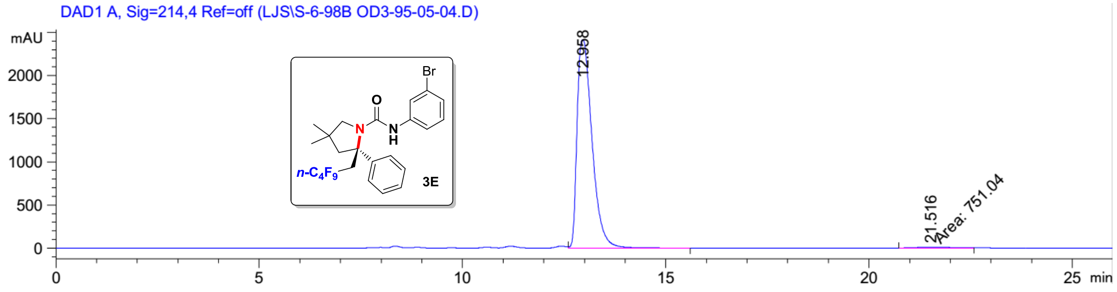

Signal 1: DAD1 A, Sig=214,4 Ref=off

Peak #	RetTime [min]	Type	Width [min]	Area [mAU*s]	Height [mAU]	Area %
1	12.958	VB	0.3669	6.14621e4	2414.93921	98.7928
2	21.516	FM	1.2016	751.03979	10.41683	1.2072
Totals :				6.22131e4	2425.35604	

Supplementary Figure 165. HPLC traces for racemic and chiral product **3E**

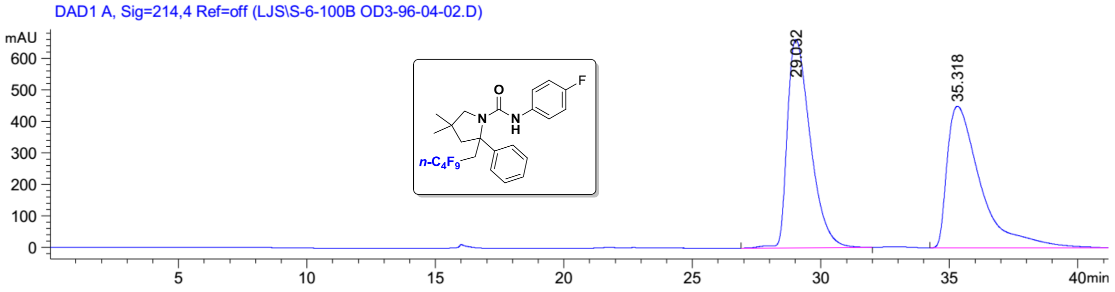

Signal 1: DAD1 A, Sig=214,4 Ref=off

Peak #	RetTime [min]	Type	Width [min]	Area [mAU*s]	Height [mAU]	Area %
1	29.032	BB	0.9417	4.10942e4	660.61847	50.2033
2	35.318	BBA	1.3147	4.07614e4	449.16583	49.7967

Totals : 8.18556e4 1109.78430

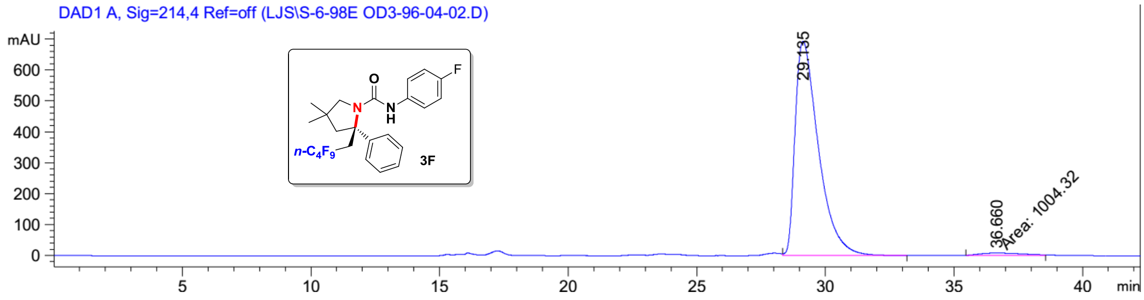

Signal 1: DAD1 A, Sig=214,4 Ref=off

Peak #	RetTime [min]	Type	Width [min]	Area [mAU*s]	Height [mAU]	Area %
1	29.135	VB	0.9352	4.37860e4	692.91858	97.7577
2	36.660	FM	2.0435	1004.32001	8.19115	2.2423

Totals : 4.47903e4 701.10973

Supplementary Figure 166. HPLC traces for racemic and chiral product **3F**

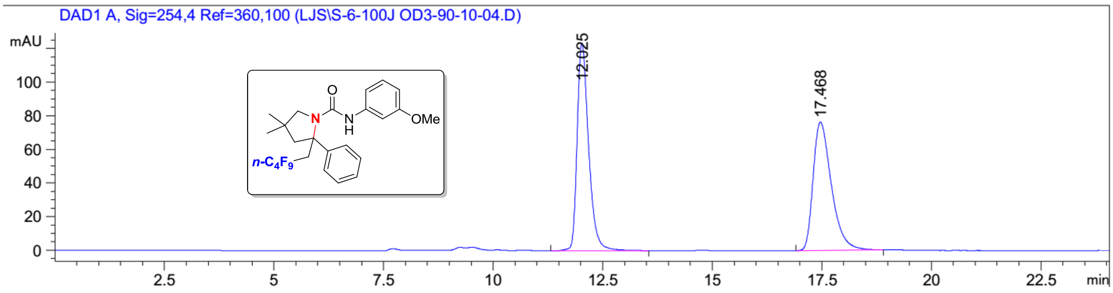

Signal 1: DAD1 A, Sig=254,4 Ref=360,100

Peak #	RetTime [min]	Type	Width [min]	Area [mAU*s]	Height [mAU]	Area %
1	12.025	BB	0.2781	2243.88403	123.44540	50.4227
2	17.468	BB	0.4412	2206.26514	76.32285	49.5773

Totals : 4450.14917 199.76826

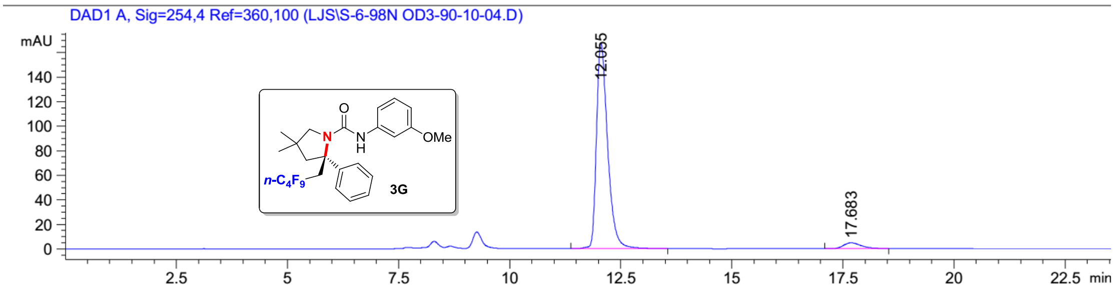

Signal 1: DAD1 A, Sig=254,4 Ref=360,100

Peak #	RetTime [min]	Type	Width [min]	Area [mAU*s]	Height [mAU]	Area %
1	12.055	BB	0.2656	2927.06689	167.72528	95.7389
2	17.683	BB	0.4225	130.27585	4.74249	4.2611

Totals : 3057.34274 172.46777

Supplementary Figure 167. HPLC traces for racemic and chiral product **3G**

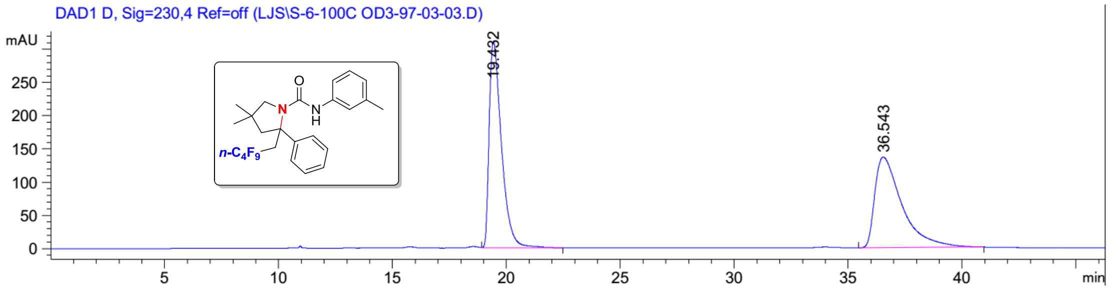

Signal 3: DAD1 D, Sig=230,4 Ref=off

Peak #	RetTime [min]	Type	Width [min]	Area [mAU*s]	Height [mAU]	Area %
1	19.432	VB	0.5648	1.16285e4	310.61533	50.6285
2	36.543	BB	1.1661	1.13398e4	136.37222	49.3715

Totals : 2.29684e4 446.98755

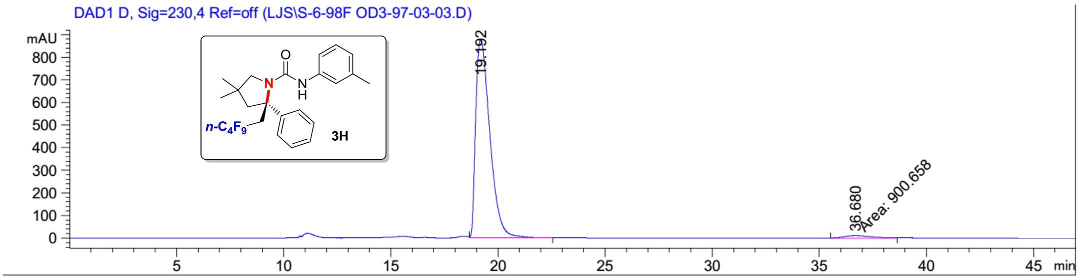

Signal 3: DAD1 D, Sig=230,4 Ref=off

Peak #	RetTime [min]	Type	Width [min]	Area [mAU*s]	Height [mAU]	Area %
1	19.192	VB	0.7127	4.02874e4	875.82117	97.8133
2	36.680	MF	1.3874	900.65814	10.81913	2.1867

Totals : 4.11881e4 886.64030

Supplementary Figure 168. HPLC traces for racemic and chiral product **3H**

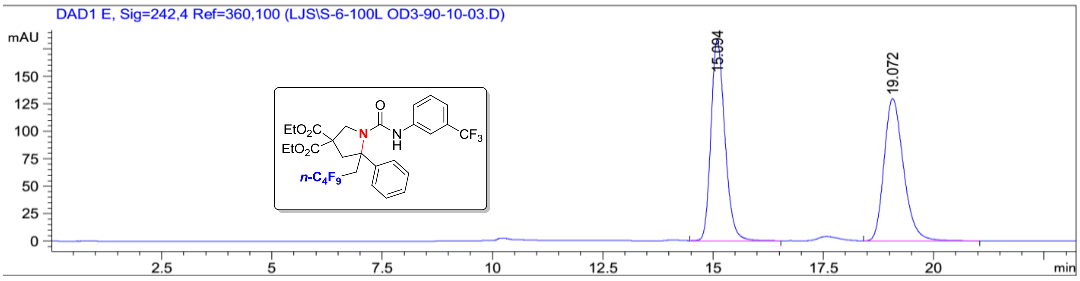

Signal 5: DAD1 E, Sig=242,4 Ref=360,100

Peak #	RetTime [min]	Type	Width [min]	Area [mAU*s]	Height [mAU]	Area %
1	15.094	BB	0.3379	4011.11133	182.87270	50.2567
2	19.072	BB	0.4693	3970.13013	129.64368	49.7433
Totals :				7981.24146	312.51637	

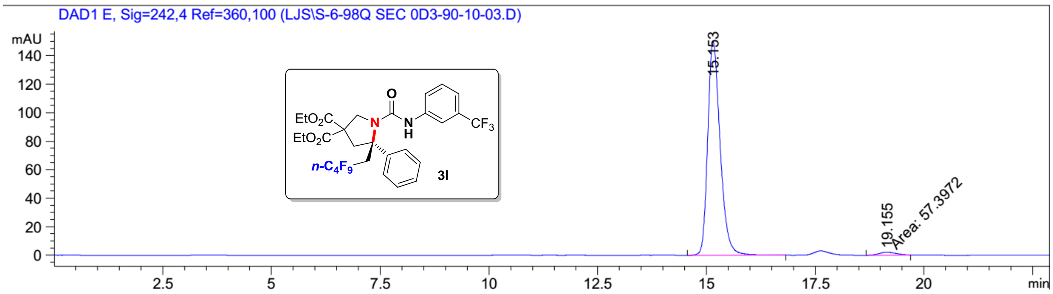

Signal 5: DAD1 E, Sig=242,4 Ref=360,100

Peak #	RetTime [min]	Type	Width [min]	Area [mAU*s]	Height [mAU]	Area %
1	15.153	BB	0.3041	2980.29883	149.79857	98.1105
2	19.155	MF	0.4505	57.39724	2.12345	1.8895
Totals :				3037.69607	151.92202	

Supplementary Figure 169. HPLC traces for racemic and chiral product **3l**

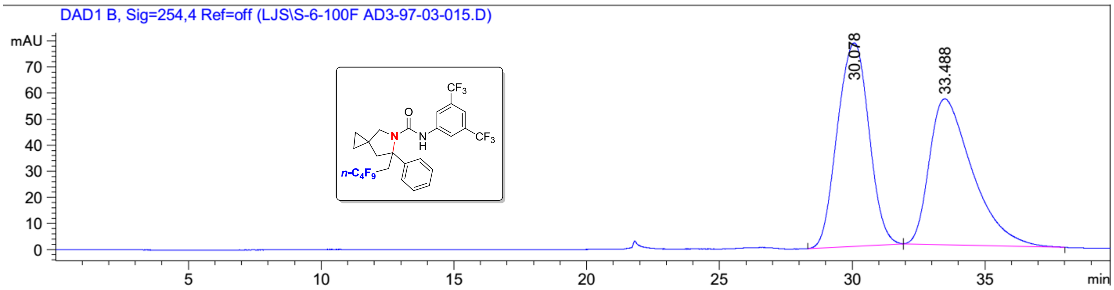

Signal 2: DAD1 B, Sig=254,4 Ref=off

Peak #	RetTime [min]	Type	Width [min]	Area [mAU*s]	Height [mAU]	Area %
1	30.078	BB	1.1360	6429.24170	77.96957	50.5137
2	33.488	BB	1.3203	6298.47168	55.90906	49.4863
Totals :				1.27277e4	133.87863	

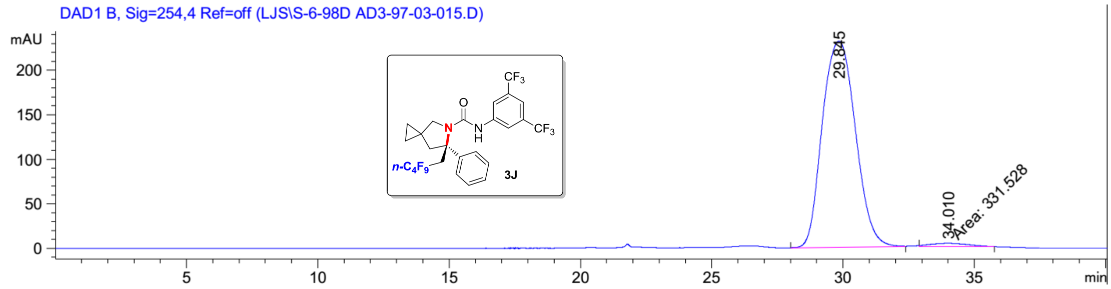

Signal 2: DAD1 B, Sig=254,4 Ref=off

Peak #	RetTime [min]	Type	Width [min]	Area [mAU*s]	Height [mAU]	Area %
1	29.845	BB	1.3882	2.02498e4	231.11113	98.3892
2	34.010	MM	1.6213	331.52789	3.40802	1.6108
Totals :				2.05813e4	234.51915	

Supplementary Figure 170. HPLC traces for racemic and chiral product **3J**

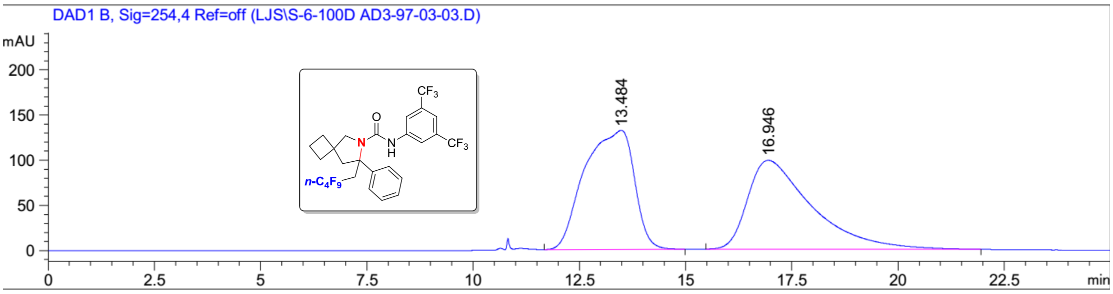

Signal 2: DAD1 B, Sig=254,4 Ref=off

Peak #	RetTime [min]	Type	Width [min]	Area [mAU*s]	Height [mAU]	Area %
1	13.484	BB	1.0718	1.07051e4	131.77003	50.8776
2	16.946	BB	1.3849	1.03358e4	98.28535	49.1224

Totals : 2.10409e4 230.05539

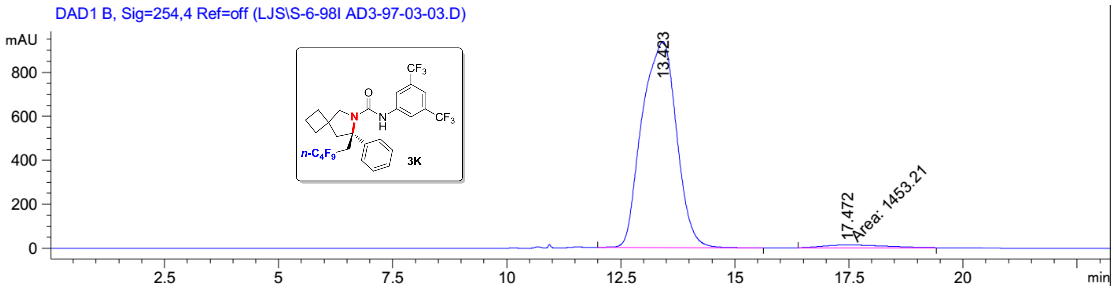

Signal 2: DAD1 B, Sig=254,4 Ref=off

Peak #	RetTime [min]	Type	Width [min]	Area [mAU*s]	Height [mAU]	Area %
1	13.423	BB	0.7243	4.91491e4	936.55481	97.1282
2	17.472	FM	1.7456	1453.21008	13.87508	2.8718

Totals : 5.06023e4 950.42989

Supplementary Figure 171. HPLC traces for racemic and chiral product **3K**

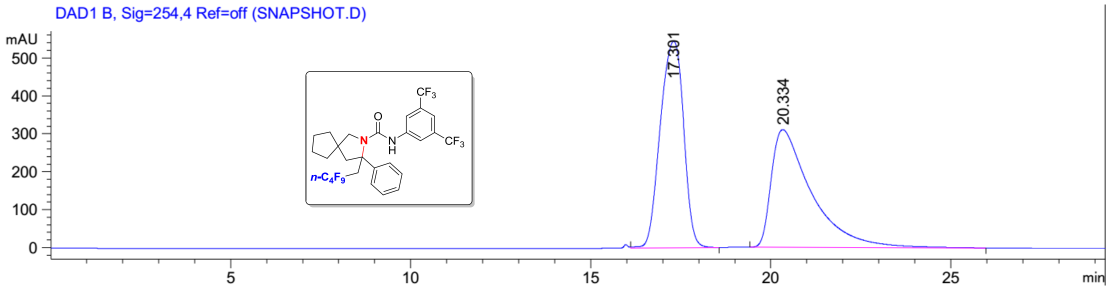

Signal 2: DAD1 B, Sig=254,4 Ref=off

Peak #	RetTime [min]	Type	Width [min]	Area [mAU*s]	Height [mAU]	Area %
1	17.301	VB	0.7481	2.46526e4	544.59674	50.5234
2	20.334	BB	1.1118	2.41417e4	310.24789	49.4766

Totals : 4.87943e4 854.84464

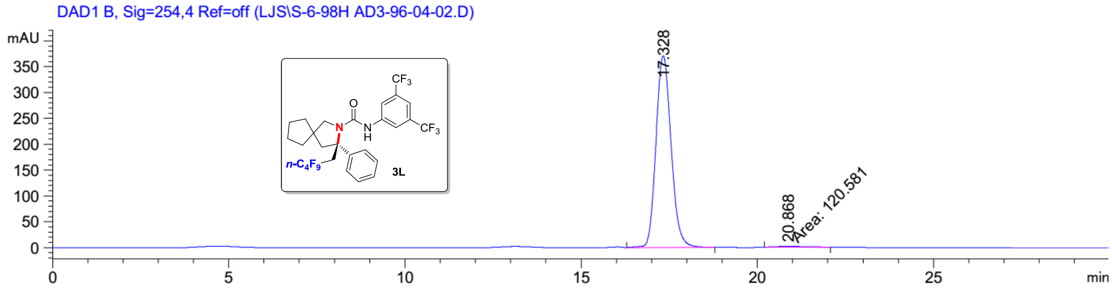

Signal 2: DAD1 B, Sig=254,4 Ref=off

Peak #	RetTime [min]	Type	Width [min]	Area [mAU*s]	Height [mAU]	Area %
1	17.328	BB	0.4663	1.10681e4	370.76822	98.9223
2	20.868	MM	1.1442	120.58081	1.75636	1.0777

Totals : 1.11886e4 372.52458

Supplementary Figure 172. HPLC traces for racemic and chiral product **3L**

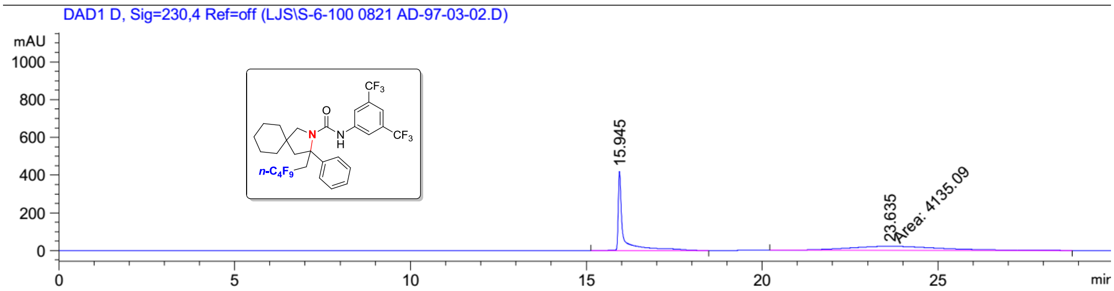

Signal 3: DAD1 D, Sig=230,4 Ref=off

Peak #	RetTime [min]	Type	Width [min]	Area [mAU*s]	Height [mAU]	Area %
1	15.945	BB	0.1351	4153.62207	420.85046	50.1118
2	23.635	MM	3.1419	4135.09473	21.93503	49.8882
Totals :				8288.71680	442.78549	

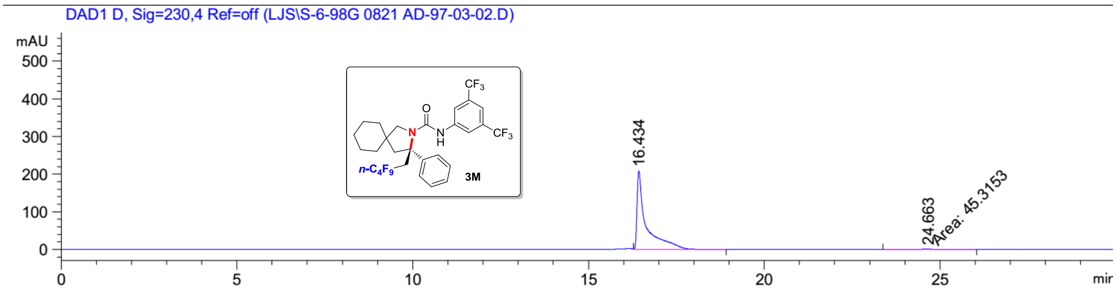

Signal 3: DAD1 D, Sig=230,4 Ref=off

Peak #	RetTime [min]	Type	Width [min]	Area [mAU*s]	Height [mAU]	Area %
1	16.434	VB	0.2405	3749.71362	207.77669	98.8059
2	24.663	MM	1.5185	45.31535	4.97354e-1	1.1941
Totals :				3795.02897	208.27404	

Supplementary Figure 173. HPLC traces for racemic and chiral product **3M**

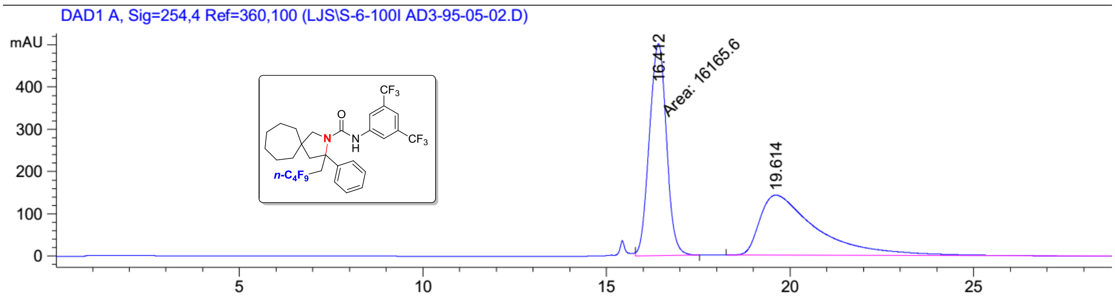

Signal 1: DAD1 A, Sig=254,4 Ref=360,100

Peak #	RetTime [min]	Type	Width [min]	Area [mAU*s]	Height [mAU]	Area %
1	16.412	FM	0.5379	1.61656e4	500.92740	51.0494
2	19.614	BB	1.5690	1.55010e4	142.50835	48.9506

Totals : 3.16667e4 643.43575

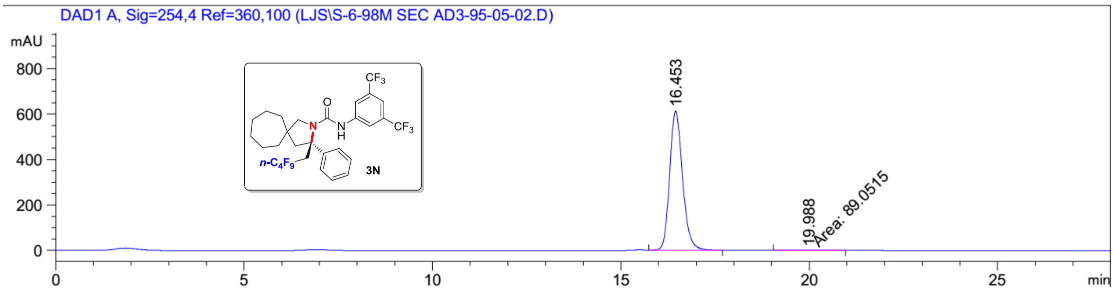

Signal 1: DAD1 A, Sig=254,4 Ref=360,100

Peak #	RetTime [min]	Type	Width [min]	Area [mAU*s]	Height [mAU]	Area %
1	16.453	BB	0.3680	1.45863e4	612.20386	99.3932
2	19.988	MM	0.7750	89.05150	1.40214	0.6068

Totals : 1.46753e4 613.60600

Supplementary Figure 174. HPLC traces for racemic and chiral product **3N**

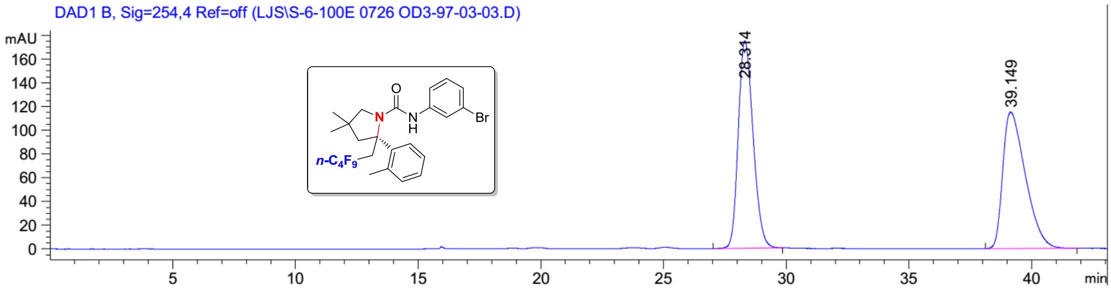

Signal 2: DAD1 B, Sig=254,4 Ref=off

Peak #	RetTime [min]	Type	Width [min]	Area [mAU*s]	Height [mAU]	Area %
1	28.314	BB	0.6504	7350.66162	174.85565	49.9251
2	39.149	BB	0.9459	7372.70605	115.01134	50.0749

Totals : 1.47234e4 289.86699

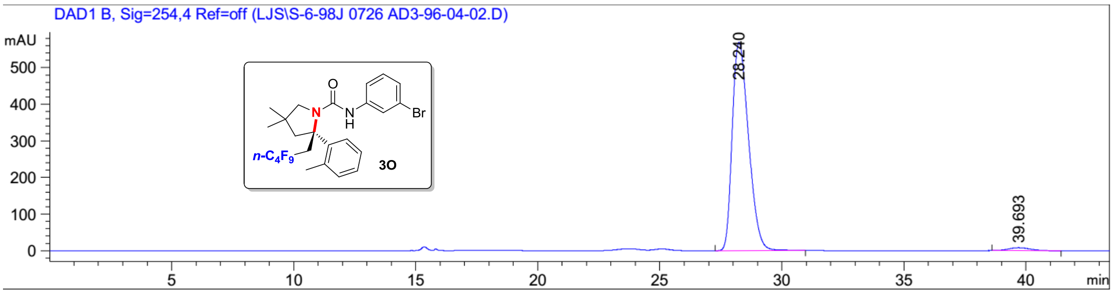

Signal 2: DAD1 B, Sig=254,4 Ref=off

Peak #	RetTime [min]	Type	Width [min]	Area [mAU*s]	Height [mAU]	Area %
1	28.240	BB	0.7203	2.63548e4	569.05585	98.1651
2	39.693	BB	0.7218	492.60959	8.02860	1.8349

Totals : 2.68474e4 577.08445

Supplementary Figure 175. HPLC traces for racemic and chiral product **3O**

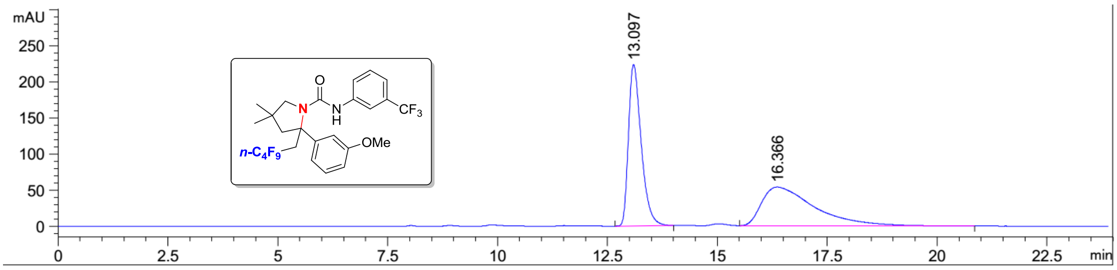


```
Signal 3: DAD1 D, Sig=230,4 Ref=off

Peak RetTime Type  Width     Area       Height      Area
  #   [min]        [min]   [mAU*s]      [mAU]        %
----|-------|----|-------|----------|----------|--------|
   1  13.097 BB    0.3067 4493.03467  223.39069  50.0887
   2  16.366 BB    1.1455 4477.12891   53.54652  49.9113

Totals :                  8970.16357  276.93721
```

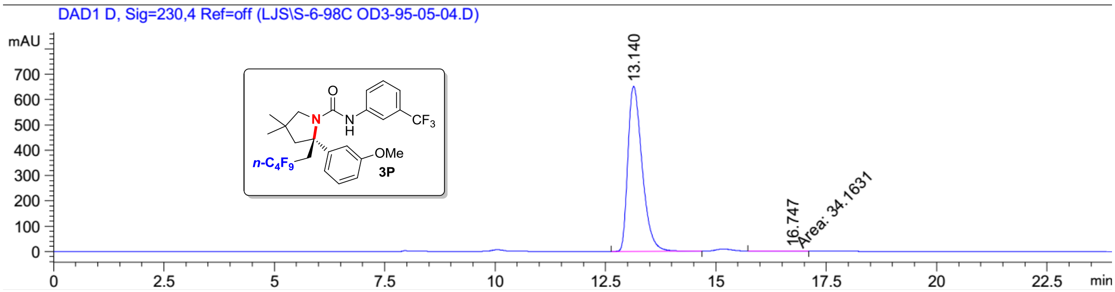


```
Signal 3: DAD1 D, Sig=230,4 Ref=off

Peak RetTime Type  Width     Area      Height      Area
  #   [min]        [min]   [mAU*s]     [mAU]        %
----|-------|----|-------|----------|----------|--------|
   1  13.140 BB    0.3480 1.46455e4  652.15692  99.7673
   2  16.747 MM    0.6239   34.16309 9.12557e-1   0.2327

Totals :                  1.46796e4  653.06948
```

Supplementary Figure 176. HPLC traces for racemic and chiral product **3P**

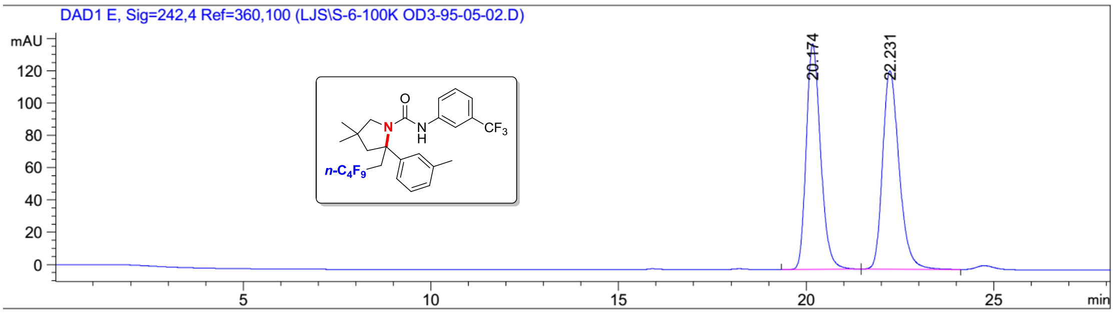

Signal 5: DAD1 E, Sig=242,4 Ref=360,100

Peak #	RetTime [min]	Type	Width [min]	Area [mAU*s]	Height [mAU]	Area %
1	20.174	BB	0.4113	3701.23022	139.62553	49.6375
2	22.231	BB	0.4689	3755.28735	122.75401	50.3625

Totals : 7456.51758 262.37954

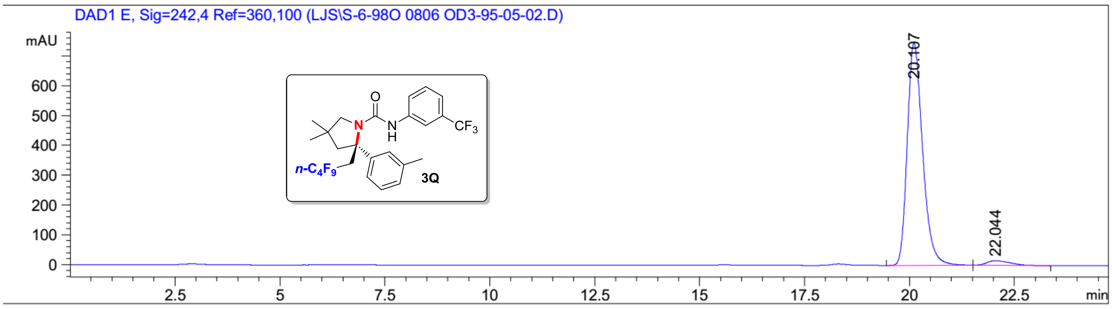

Signal 5: DAD1 E, Sig=242,4 Ref=360,100

Peak #	RetTime [min]	Type	Width [min]	Area [mAU*s]	Height [mAU]	Area %
1	20.107	BB	0.3905	1.89446e4	745.51324	97.1027
2	22.044	BB	0.6115	565.25977	15.14158	2.8973

Totals : 1.95098e4 760.65483

Supplementary Figure 177. HPLC traces for racemic and chiral product **3Q**

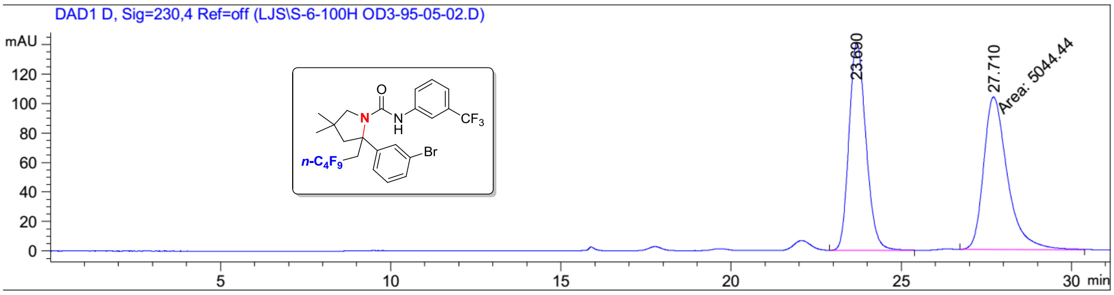

Signal 3: DAD1 D, Sig=230,4 Ref=off

Peak #	RetTime [min]	Type	Width [min]	Area [mAU*s]	Height [mAU]	Area %
1	23.690	BB	0.5366	4855.87451	140.72638	49.0477
2	27.710	MF	0.8129	5044.44141	103.42953	50.9523
Totals :				9900.31592	244.15591	

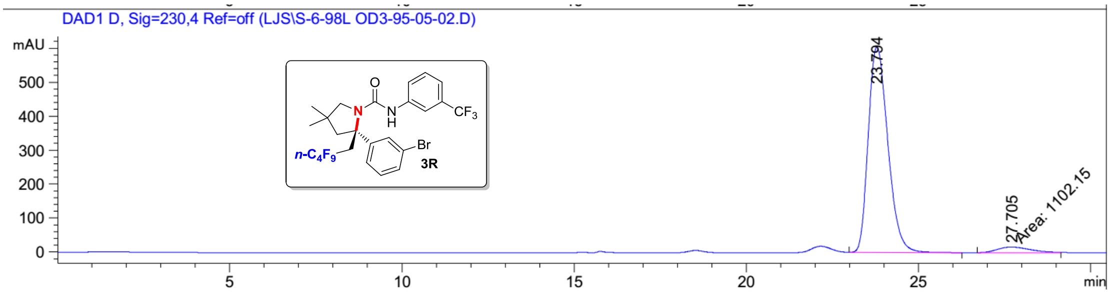

Signal 3: DAD1 D, Sig=230,4 Ref=off

Peak #	RetTime [min]	Type	Width [min]	Area [mAU*s]	Height [mAU]	Area %
1	23.794	BB	0.6220	2.38969e4	606.10822	95.5912
2	27.705	MF	1.1064	1102.15369	16.60305	4.4088
Totals :				2.49990e4	622.71127	

Supplementary Figure 178. HPLC traces for racemic and chiral product **3R**

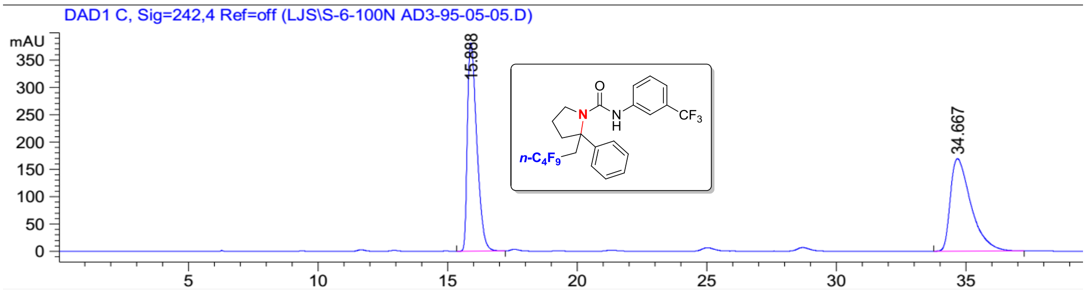

Signal 3: DAD1 C, Sig=242,4 Ref=off

Peak #	RetTime [min]	Type	Width [min]	Area [mAU*s]	Height [mAU]	Area %
1	15.888	BB	0.3917	9584.79395	380.68045	50.6591
2	34.667	BB	0.8430	9335.37891	168.85371	49.3409

Totals : 1.89202e4 549.53416

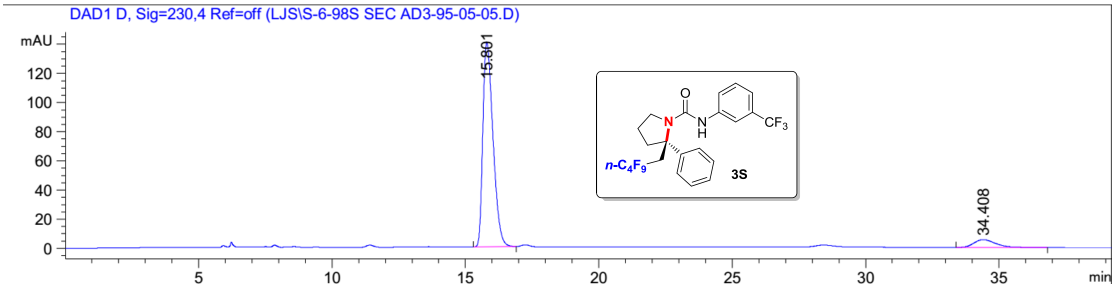

Signal 3: DAD1 D, Sig=230,4 Ref=off

Peak #	RetTime [min]	Type	Width [min]	Area [mAU*s]	Height [mAU]	Area %
1	15.801	BB	0.4099	3706.19043	140.43170	92.5880
2	34.408	BB	0.7429	296.69266	5.31766	7.4120

Totals : 4002.88309 145.74936

Supplementary Figure 179. HPLC traces for racemic and chiral product **3S**

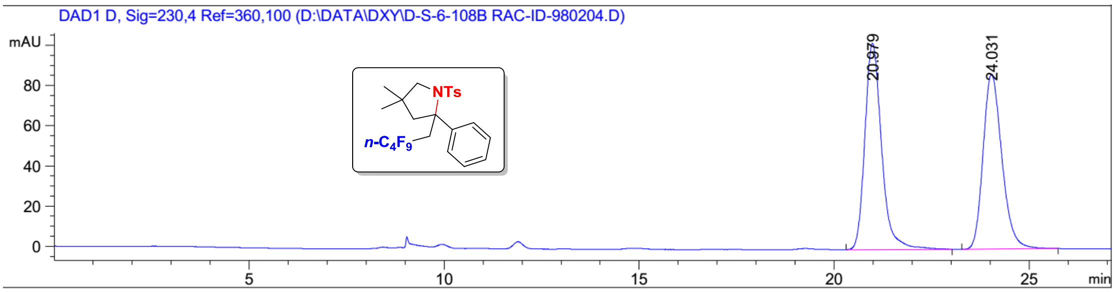

Signal 4: DAD1 D, Sig=230,4 Ref=360,100

Peak #	RetTime [min]	Type	Width [min]	Area [mAU*s]	Height [mAU]	Area %
1	20.979	BB	0.4578	3059.14771	102.61470	51.1492
2	24.031	BB	0.5173	2921.68066	87.11403	48.8508

Totals : 5980.82837 189.72873

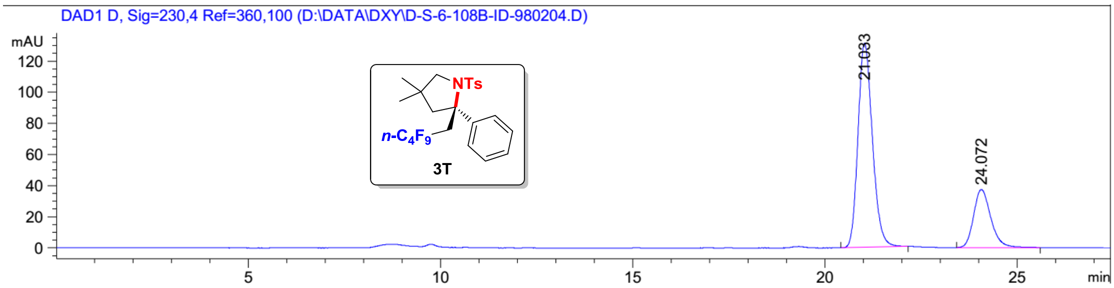

Signal 4: DAD1 D, Sig=230,4 Ref=360,100

Peak #	RetTime [min]	Type	Width [min]	Area [mAU*s]	Height [mAU]	Area %
1	21.033	BB	0.4016	3415.71509	131.27971	74.6704
2	24.072	BB	0.4739	1158.67334	37.35483	25.3296

Totals : 4574.38843 168.63454

Supplementary Figure 180. HPLC traces for racemic and chiral product **3T**

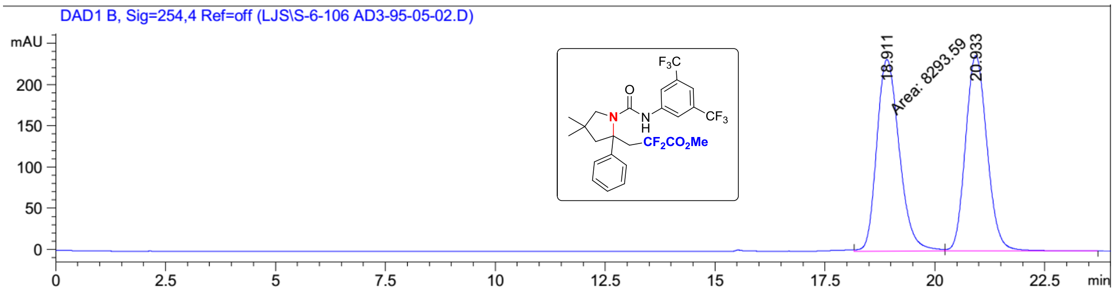

Signal 2: DAD1 B, Sig=254,4 Ref=off

Peak #	RetTime [min]	Type	Width [min]	Area [mAU*s]	Height [mAU]	Area %
1	18.911	FM	0.5952	8293.58789	232.24873	50.6721
2	20.933	VB	0.5315	8073.58789	236.93889	49.3279
Totals :				1.63672e4	469.18762	

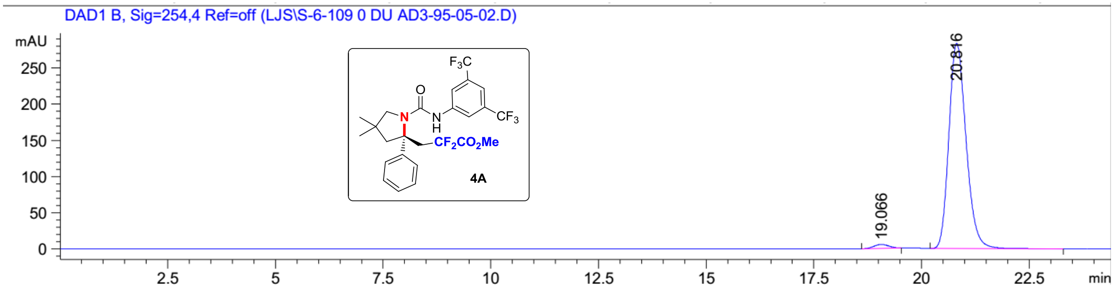

Signal 2: DAD1 B, Sig=254,4 Ref=off

Peak #	RetTime [min]	Type	Width [min]	Area [mAU*s]	Height [mAU]	Area %
1	19.066	BB	0.3825	130.83678	5.44195	1.6322
2	20.816	BB	0.4302	7885.35889	283.77231	98.3678
Totals :				8016.19566	289.21426	

Supplementary Figure 181. HPLC traces for racemic and chiral product **4A**

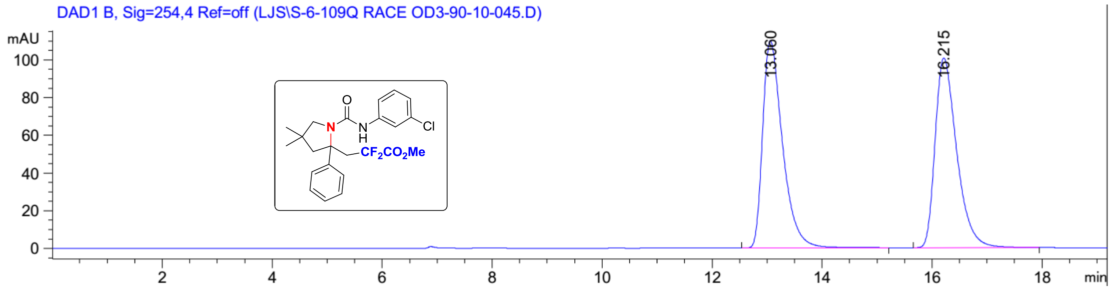

Signal 2: DAD1 B, Sig=254,4 Ref=off

Peak #	RetTime [min]	Type	Width [min]	Area [mAU*s]	Height [mAU]	Area %
1	13.060	BB	0.3829	2760.13477	109.95606	50.1234
2	16.215	BB	0.4165	2746.53882	100.59978	49.8766
Totals :				5506.67358	210.55584	

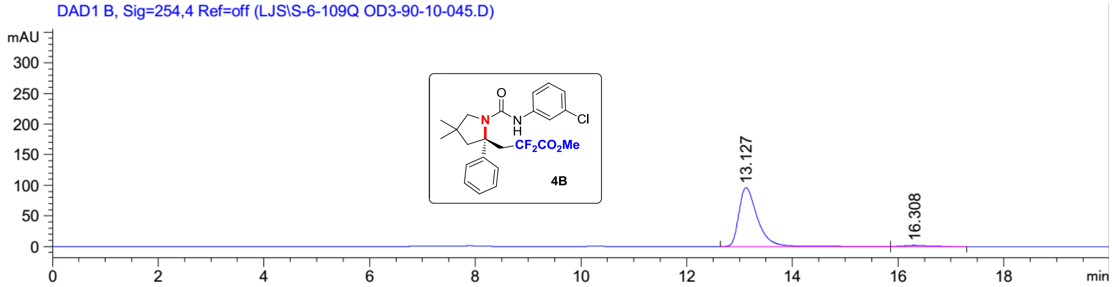

Signal 2: DAD1 B, Sig=254,4 Ref=off

Peak #	RetTime [min]	Type	Width [min]	Area [mAU*s]	Height [mAU]	Area %
1	13.127	BB	0.3651	2316.44653	96.13580	97.9963
2	16.308	BB	0.3959	47.36271	1.73811	2.0037
Totals :				2363.80924	97.87391	

Supplementary Figure 182. HPLC traces for racemic and chiral product **4B**

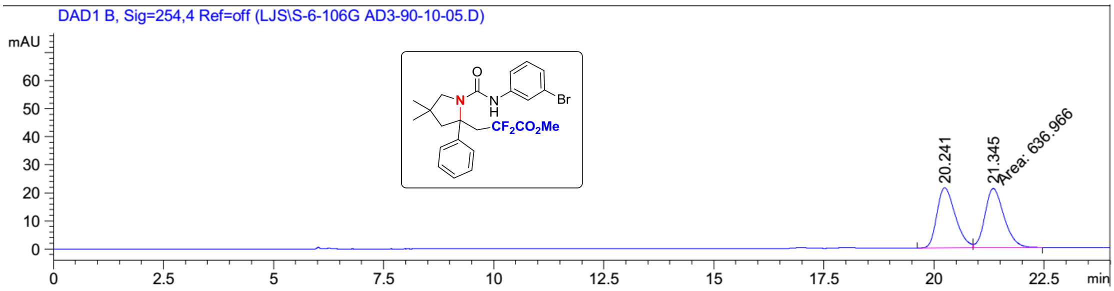

Signal 2: DAD1 B, Sig=254,4 Ref=off

Peak #	RetTime [min]	Type	Width [min]	Area [mAU*s]	Height [mAU]	Area %
1	20.241	BV	0.4505	622.35138	21.44972	49.4197
2	21.345	MF	0.5023	636.96649	21.13545	50.5803

Totals : 1259.31787 42.58517

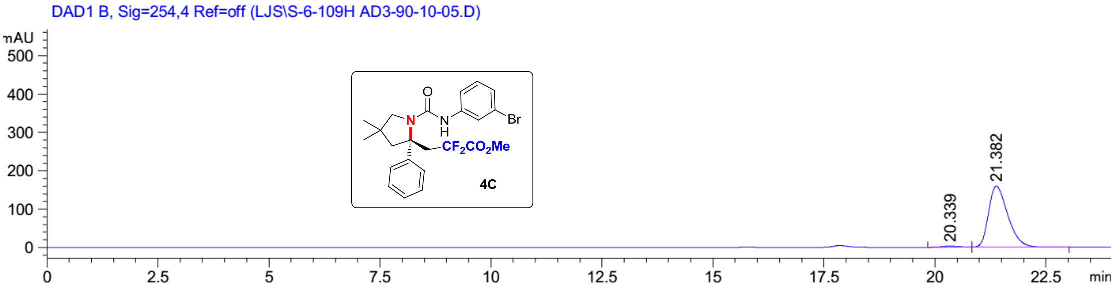

Signal 2: DAD1 B, Sig=254,4 Ref=off

Peak #	RetTime [min]	Type	Width [min]	Area [mAU*s]	Height [mAU]	Area %
1	20.339	BB	0.3902	70.69450	2.76572	1.4640
2	21.382	BB	0.4592	4758.01465	158.95325	98.5360

Totals : 4828.70914 161.71896

Supplementary Figure 183. HPLC traces for racemic and chiral product **4C**

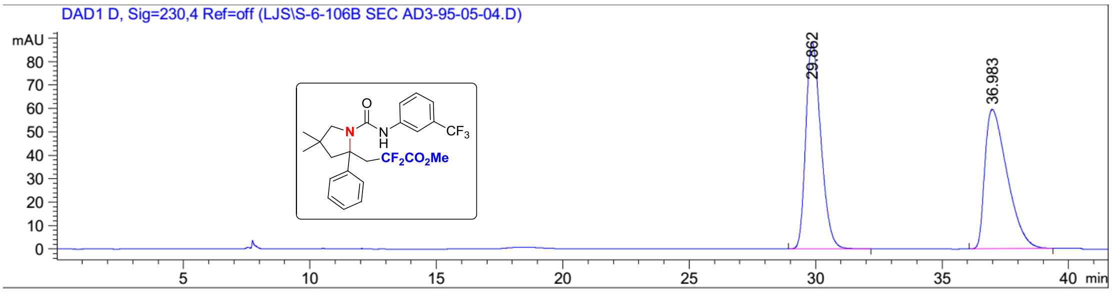

Signal 3: DAD1 D, Sig=230,4 Ref=off

Peak #	RetTime [min]	Type	Width [min]	Area [mAU*s]	Height [mAU]	Area %
1	29.862	BB	0.6430	3654.70972	88.27077	50.0938
2	36.983	BB	0.9280	3641.02905	59.49714	49.9062

Totals : 7295.73877 147.76791

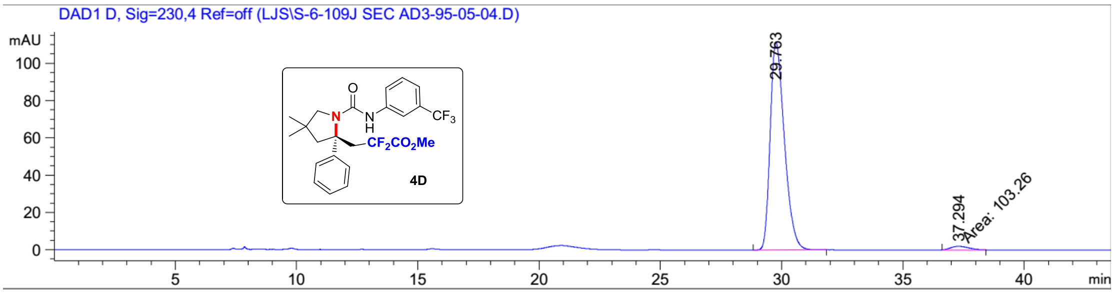

Signal 3: DAD1 D, Sig=230,4 Ref=off

Peak #	RetTime [min]	Type	Width [min]	Area [mAU*s]	Height [mAU]	Area %
1	29.763	BB	0.6266	4550.85645	111.40556	97.7813
2	37.294	MM	0.8737	103.26048	1.96976	2.2187

Totals : 4654.11693 113.37533

Supplementary Figure 184. HPLC traces for racemic and chiral product **4D**

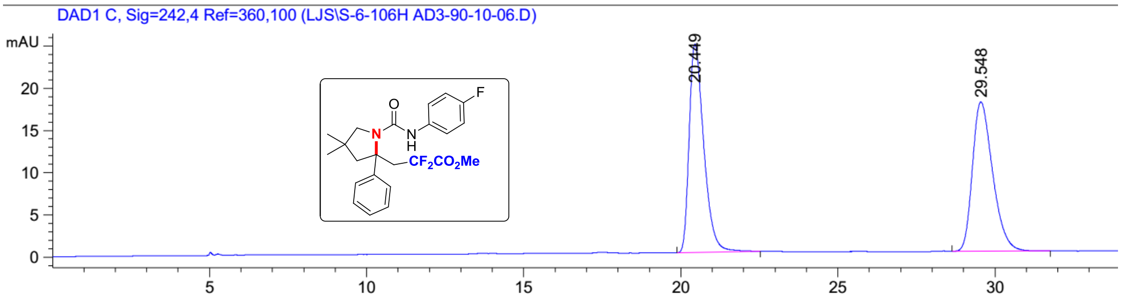

Signal 3: DAD1 C, Sig=242,4 Ref=360,100

Peak #	RetTime [min]	Type	Width [min]	Area [mAU*s]	Height [mAU]	Area %
1	20.449	BB	0.4952	798.98615	24.71168	50.2485
2	29.548	BB	0.6697	791.08398	17.68947	49.7515
Totals :				1590.07013	42.40115	

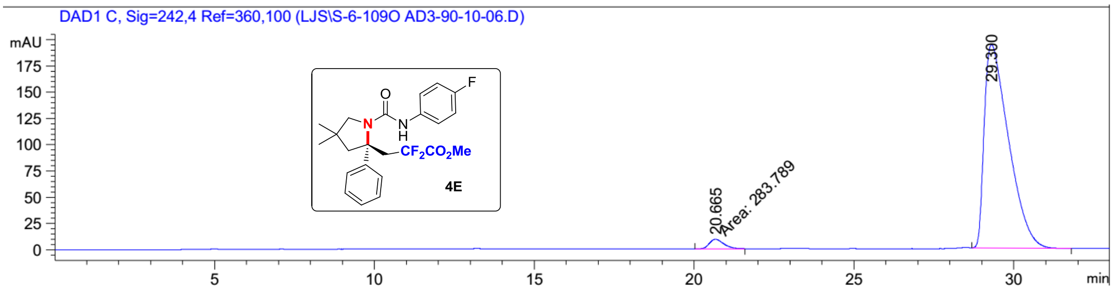

Signal 3: DAD1 C, Sig=242,4 Ref=360,100

Peak #	RetTime [min]	Type	Width [min]	Area [mAU*s]	Height [mAU]	Area %
1	20.665	MF	0.5220	283.78854	9.06128	2.7010
2	29.300	BB	0.8016	1.02230e4	193.74638	97.2990
Totals :				1.05068e4	202.80766	

Supplementary Figure 185. HPLC traces for racemic and chiral product **4E**

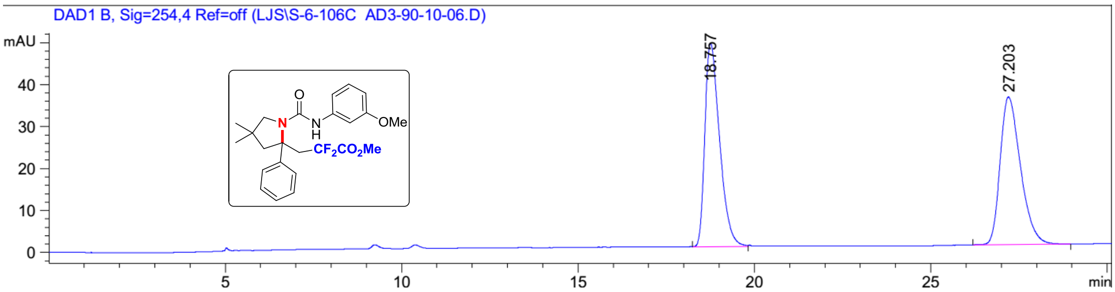

Signal 2: DAD1 B, Sig=254,4 Ref=off

Peak #	RetTime [min]	Type	Width [min]	Area [mAU*s]	Height [mAU]	Area %
1	18.757	BV	0.4459	1402.28833	48.41142	49.8962
2	27.203	BB	0.6115	1408.12195	35.14347	50.1038

Totals : 2810.41028 83.55489

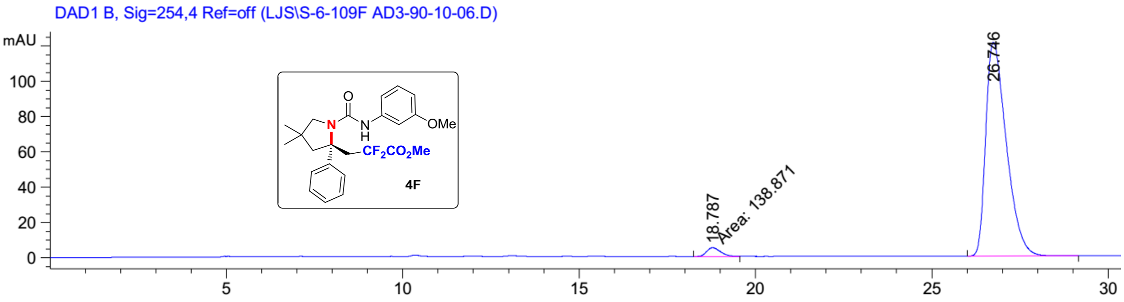

Signal 2: DAD1 B, Sig=254,4 Ref=off

Peak #	RetTime [min]	Type	Width [min]	Area [mAU*s]	Height [mAU]	Area %
1	18.787	MF	0.4638	138.87114	4.98993	2.7483
2	26.746	BB	0.6212	4914.18896	121.14713	97.2517

Totals : 5053.06010 126.13706

Supplementary Figure 186. HPLC traces for racemic and chiral product **4F**

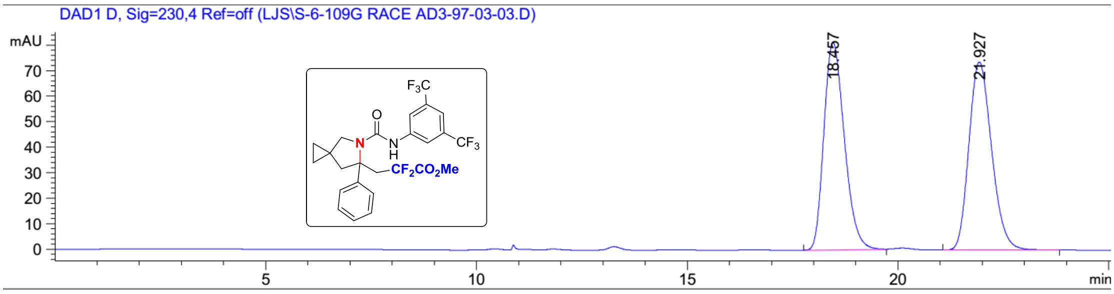

Signal 3: DAD1 D, Sig=230,4 Ref=off

Peak #	RetTime [min]	Type	Width [min]	Area [mAU*s]	Height [mAU]	Area %
1	18.457	BB	0.5276	2741.54102	81.27146	49.8816
2	21.927	BB	0.5843	2754.55054	73.65489	50.1184
Totals :				5496.09155	154.92635	

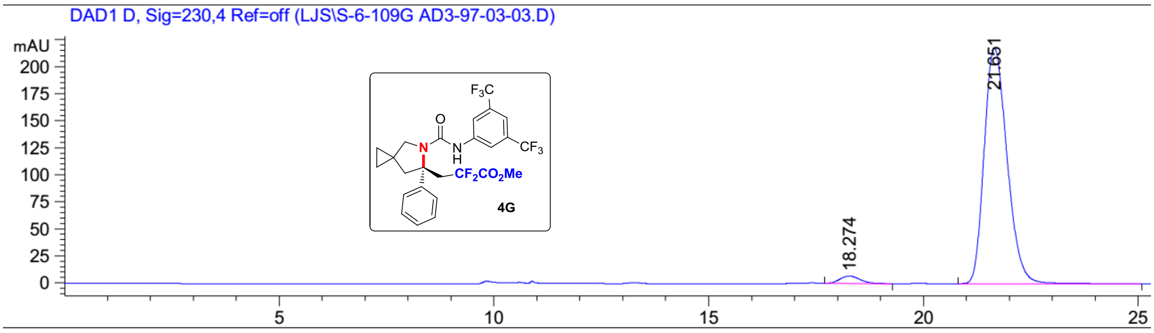

Signal 3: DAD1 D, Sig=230,4 Ref=off

Peak #	RetTime [min]	Type	Width [min]	Area [mAU*s]	Height [mAU]	Area %
1	18.274	BB	0.5062	218.56804	6.81420	2.6333
2	21.651	BB	0.5798	8081.47705	218.35092	97.3667
Totals :				8300.04509	225.16512	

Supplementary Figure 187. HPLC traces for racemic and chiral product **4G**

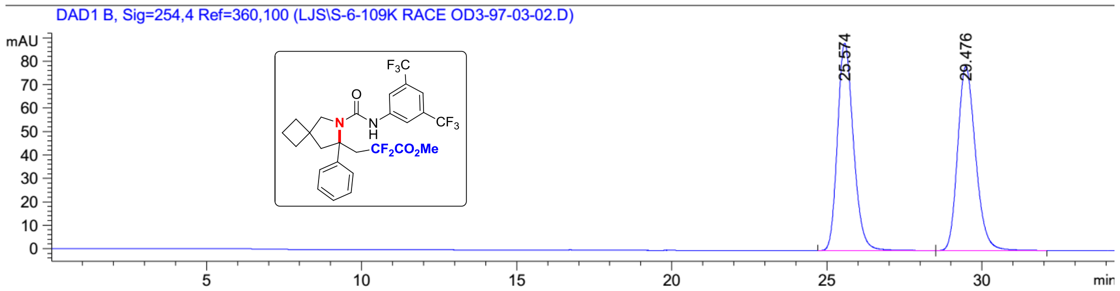

Signal 2: DAD1 B, Sig=254,4 Ref=360,100

Peak #	RetTime [min]	Type	Width [min]	Area [mAU*s]	Height [mAU]	Area %
1	25.574	BB	0.5547	3158.97607	88.44090	50.0967
2	29.476	BB	0.6199	3146.77954	78.45965	49.9033
Totals :				6305.75562	166.90054	

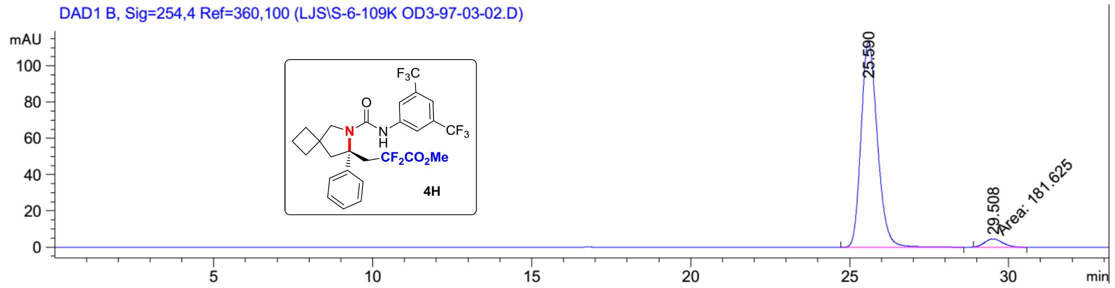

Signal 2: DAD1 B, Sig=254,4 Ref=360,100

Peak #	RetTime [min]	Type	Width [min]	Area [mAU*s]	Height [mAU]	Area %
1	25.590	BB	0.5516	4052.02686	113.75243	95.7100
2	29.508	FM	0.6596	181.62512	4.58960	4.2900
Totals :				4233.65198	118.34203	

Supplementary Figure 188. HPLC traces for racemic and chiral product **4H**

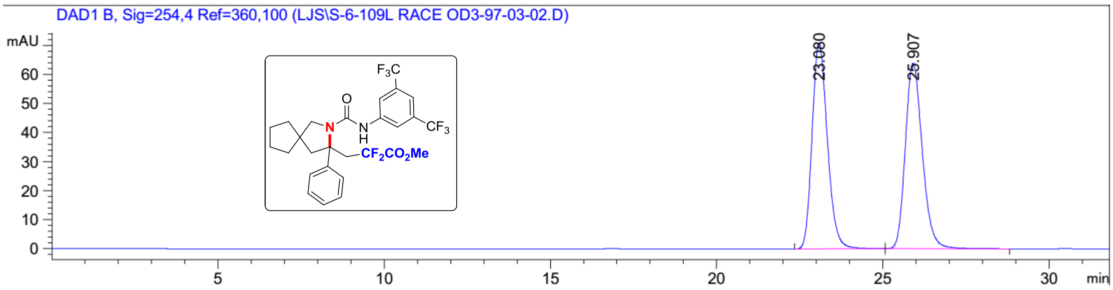

Signal 2: DAD1 B, Sig=254,4 Ref=360,100

Peak #	RetTime [min]	Type	Width [min]	Area [mAU*s]	Height [mAU]	Area %
1	23.080	BB	0.4976	2271.62842	70.92879	49.9807
2	25.907	BB	0.5530	2273.38086	63.90498	50.0193

Totals : 4545.00928 134.83378

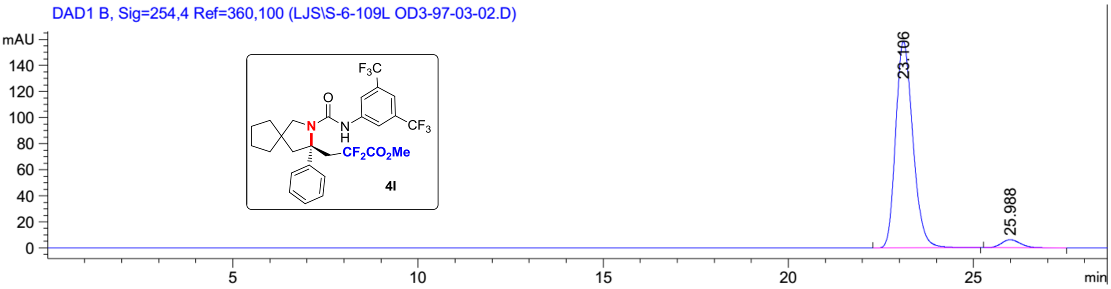

Signal 2: DAD1 B, Sig=254,4 Ref=360,100

Peak #	RetTime [min]	Type	Width [min]	Area [mAU*s]	Height [mAU]	Area %
1	23.106	BB	0.5002	5106.04883	158.36592	95.9077
2	25.988	BB	0.5385	217.87109	6.16192	4.0923

Totals : 5323.91992 164.52784

Supplementary Figure 189. HPLC traces for racemic and chiral product **4l**

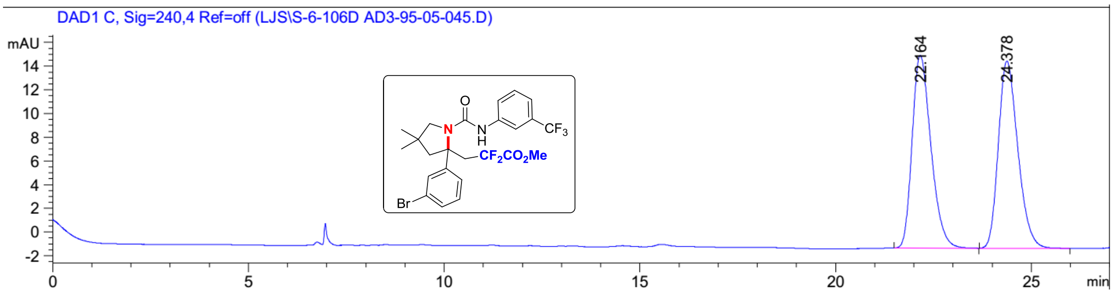

Signal 3: DAD1 C, Sig=240,4 Ref=off

Peak #	RetTime [min]	Type	Width [min]	Area [mAU*s]	Height [mAU]	Area %
1	22.164	BB	0.5033	542.76874	16.26550	49.8301
2	24.378	BB	0.5277	546.46912	15.79296	50.1699
Totals :				1089.23785	32.05846	

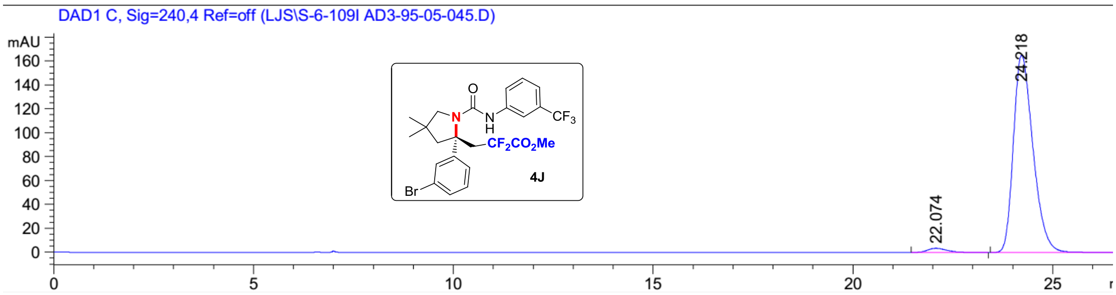

Signal 3: DAD1 C, Sig=240,4 Ref=off

Peak #	RetTime [min]	Type	Width [min]	Area [mAU*s]	Height [mAU]	Area %
1	22.074	BB	0.4836	113.29708	3.44599	1.9762
2	24.218	BB	0.5251	5619.78711	165.12523	98.0238
Totals :				5733.08419	168.57121	

Supplementary Figure 190. HPLC traces for racemic and chiral product **4J**

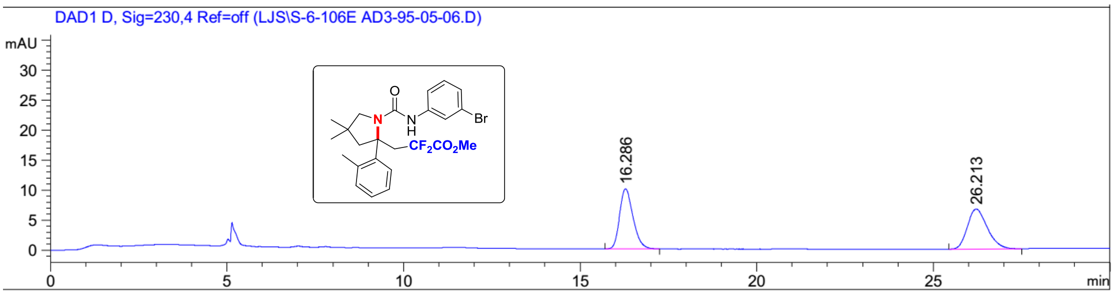

Signal 4: DAD1 D, Sig=230,4 Ref=off

Peak #	RetTime [min]	Type	Width [min]	Area [mAU*s]	Height [mAU]	Area %
1	16.286	BB	0.4051	262.91132	9.98912	50.1903
2	26.213	BB	0.5799	260.91800	6.64845	49.8097

Totals : 523.82932 16.63757

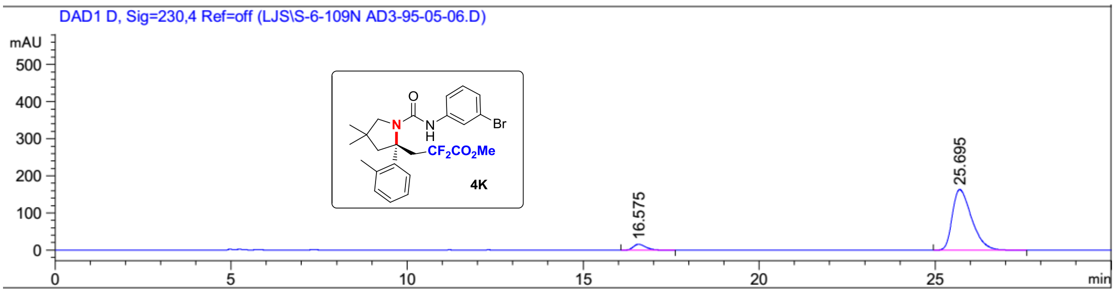

Signal 4: DAD1 D, Sig=230,4 Ref=off

Peak #	RetTime [min]	Type	Width [min]	Area [mAU*s]	Height [mAU]	Area %
1	16.575	BB	0.3735	388.19284	15.86223	5.8823
2	25.695	BB	0.5854	6211.13281	163.42307	94.1177

Totals : 6599.32565 179.28530

Supplementary Figure 191. HPLC traces for racemic and chiral product **4K**

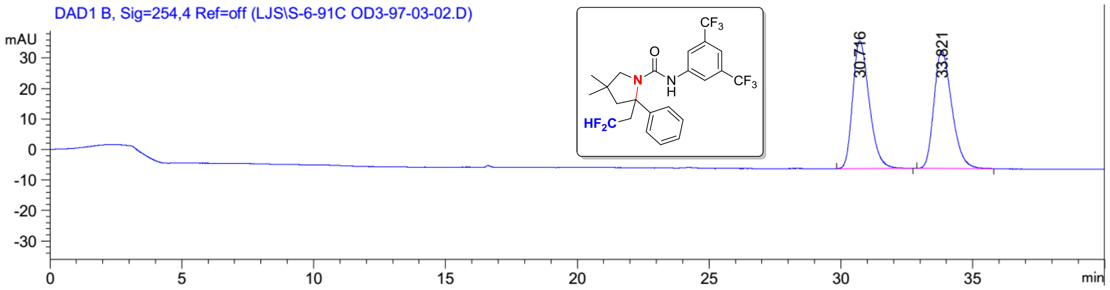

Signal 2: DAD1 B, Sig=254,4 Ref=off

Peak #	RetTime [min]	Type	Width [min]	Area [mAU*s]	Height [mAU]	Area %
1	30.716	BB	0.6670	1826.91833	41.87351	50.0669
2	33.821	BB	0.7082	1822.03638	38.63860	49.9331

Totals : 3648.95471 80.51211

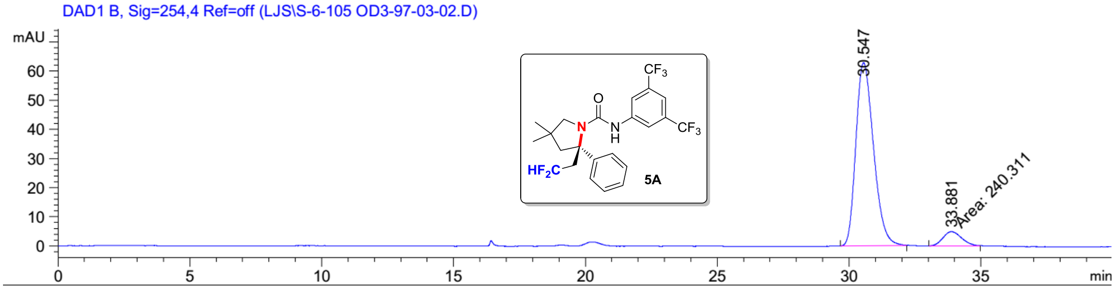

Signal 2: DAD1 B, Sig=254,4 Ref=off

Peak #	RetTime [min]	Type	Width [min]	Area [mAU*s]	Height [mAU]	Area %
1	30.547	BB	0.7163	2922.48291	62.87646	92.4020
2	33.881	MF	0.8204	240.31059	4.88187	7.5980

Totals : 3162.79350 67.75834

Supplementary Figure 192. HPLC traces for racemic and chiral product **5A**

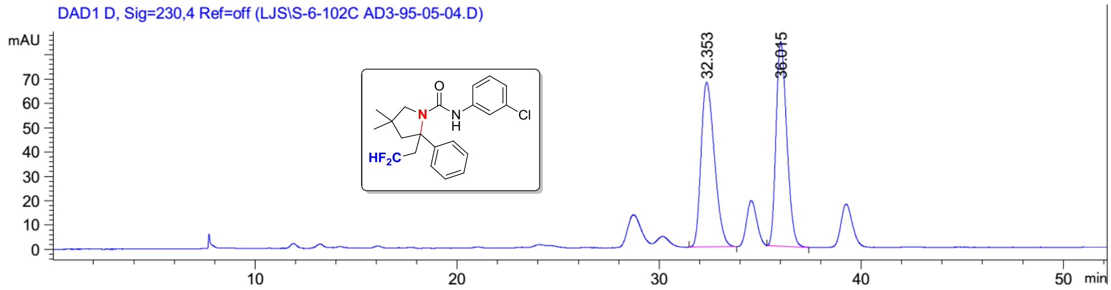

Signal 3: DAD1 D, Sig=230,4 Ref=off

Peak #	RetTime [min]	Type	Width [min]	Area [mAU*s]	Height [mAU]	Area %
1	32.353	BB	0.6955	3116.70142	67.65095	50.1120
2	36.015	BB	0.5710	3102.76538	83.99167	49.8880

Totals : 6219.46680 151.64262

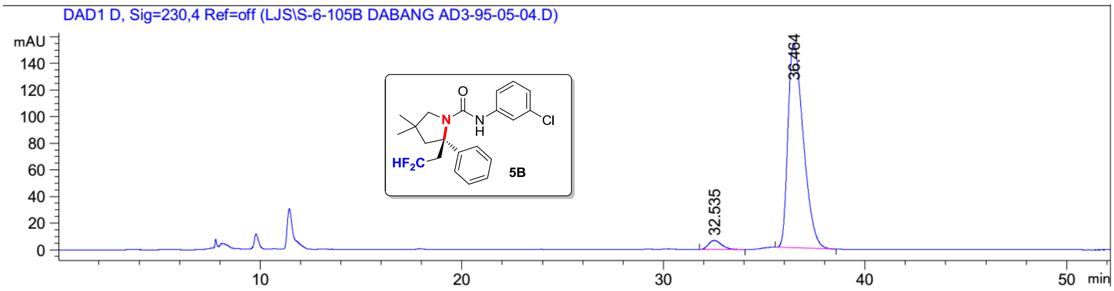

Signal 3: DAD1 D, Sig=230,4 Ref=off

Peak #	RetTime [min]	Type	Width [min]	Area [mAU*s]	Height [mAU]	Area %
1	32.535	BB	0.5922	315.44385	6.83765	3.7296
2	36.464	BB	0.8076	8142.33008	153.81068	96.2704

Totals : 8457.77393 160.64834

Supplementary Figure 193. HPLC traces for racemic and chiral product **5B**

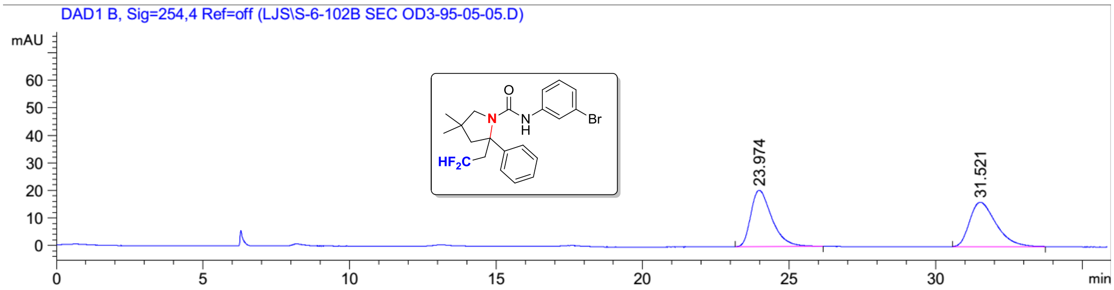

Signal 2: DAD1 B, Sig=254,4 Ref=off

Peak #	RetTime [min]	Type	Width [min]	Area [mAU*s]	Height [mAU]	Area %
1	23.974	BB	0.6997	1021.82849	20.49335	50.2755
2	31.521	BB	0.7449	1010.62885	16.13207	49.7245
Totals :				2032.45734	36.62542	

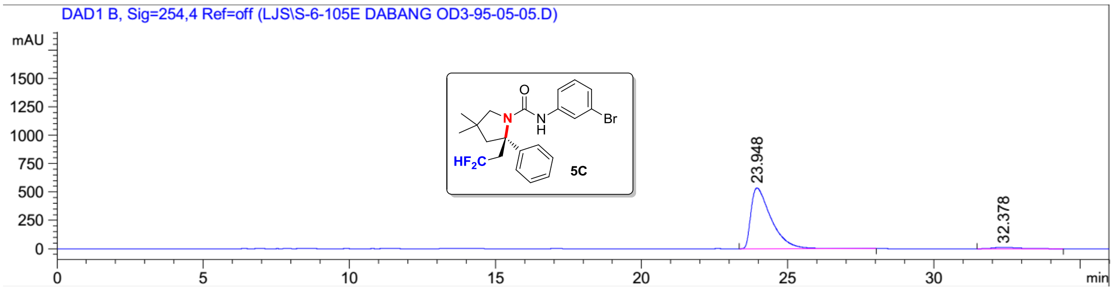

Signal 2: DAD1 B, Sig=254,4 Ref=off

Peak #	RetTime [min]	Type	Width [min]	Area [mAU*s]	Height [mAU]	Area %
1	23.948	BB	0.7137	2.57170e4	534.11377	97.3548
2	32.378	BB	0.7091	698.74554	11.96944	2.6452
Totals :				2.64157e4	546.08321	

Supplementary Figure 194. HPLC traces for racemic and chiral product **5C**

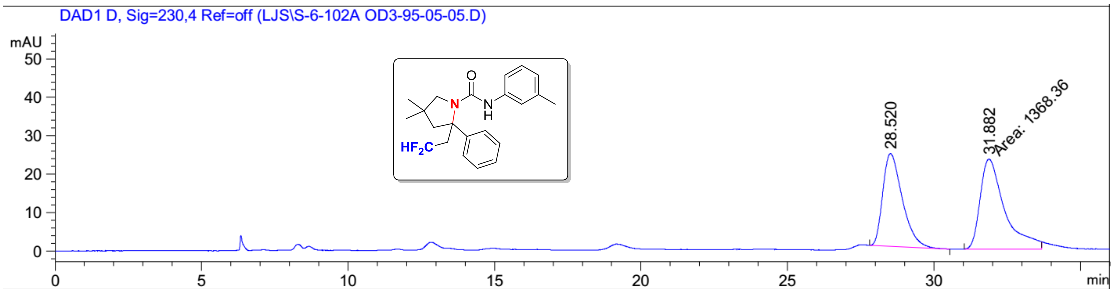

Signal 3: DAD1 D, Sig=230,4 Ref=off

Peak #	RetTime [min]	Type	Width [min]	Area [mAU*s]	Height [mAU]	Area %
1	28.520	BB	0.6530	1126.02942	24.19506	45.1425
2	31.882	MF	0.9721	1368.36169	23.45948	54.8575

Totals : 2494.39111 47.65454

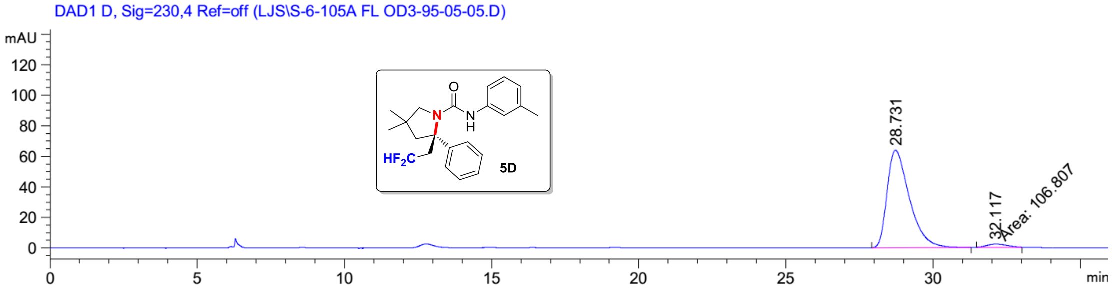

Signal 3: DAD1 D, Sig=230,4 Ref=off

Peak #	RetTime [min]	Type	Width [min]	Area [mAU*s]	Height [mAU]	Area %
1	28.731	BB	0.7663	3372.67847	63.84912	96.9304
2	32.117	MM	0.8196	106.80743	2.17200	3.0696

Totals : 3479.48589 66.02112

Supplementary Figure 195. HPLC traces for racemic and chiral product **5D**

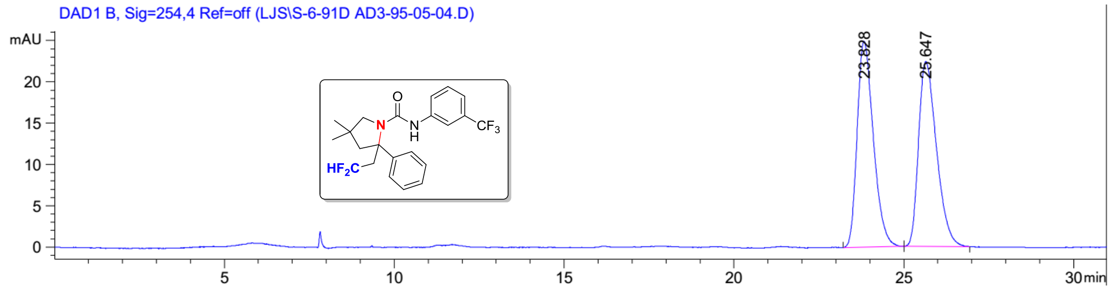

Signal 2: DAD1 B, Sig=254,4 Ref=off

Peak #	RetTime [min]	Type	Width [min]	Area [mAU*s]	Height [mAU]	Area %
1	23.828	BB	0.4906	825.62091	24.91761	50.1276
2	25.647	BB	0.5443	821.41687	22.37748	49.8724

Totals : 1647.03778 47.29509

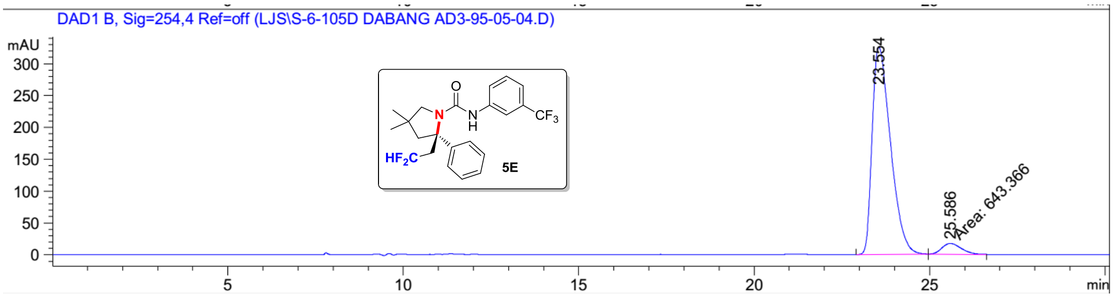

Signal 2: DAD1 B, Sig=254,4 Ref=off

Peak #	RetTime [min]	Type	Width [min]	Area [mAU*s]	Height [mAU]	Area %
1	23.554	BB	0.5595	1.18147e4	325.49966	94.8357
2	25.586	MF	0.6369	643.36609	16.83549	5.1643

Totals : 1.24580e4 342.33515

Supplementary Figure 196. HPLC traces for racemic and chiral product **5E**

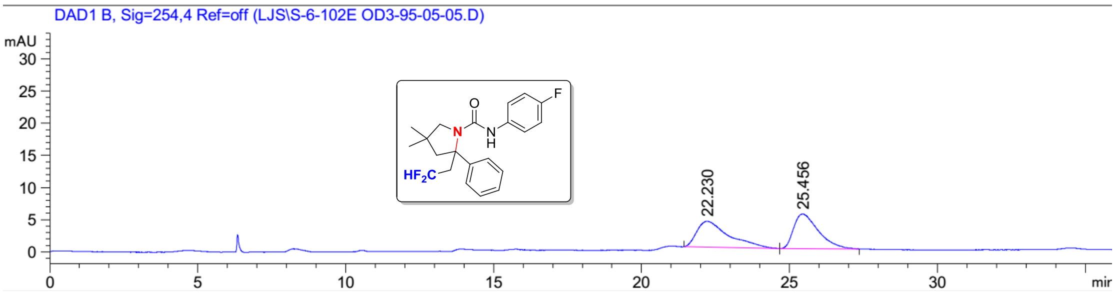

Signal 2: DAD1 B, Sig=254,4 Ref=off

Peak #	RetTime [min]	Type	Width [min]	Area [mAU*s]	Height [mAU]	Area %
1	22.230	BB	0.8903	304.10242	4.00317	49.1315
2	25.456	BB	0.6946	314.85309	5.39678	50.8685
Totals :				618.95551	9.39995	

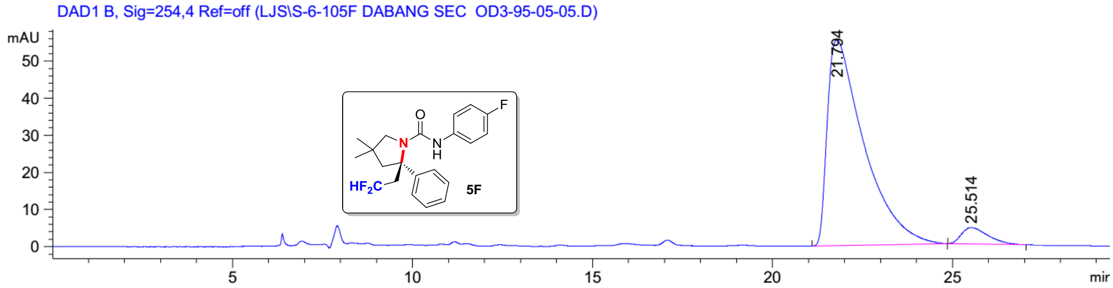

Signal 2: DAD1 B, Sig=254,4 Ref=off

Peak #	RetTime [min]	Type	Width [min]	Area [mAU*s]	Height [mAU]	Area %
1	21.794	BB	0.9598	3927.75415	55.49100	94.5663
2	25.514	BB	0.5990	225.68350	4.42540	5.4337
Totals :				4153.43765	59.91641	

Supplementary Figure 197. HPLC traces for racemic and chiral product **5F**

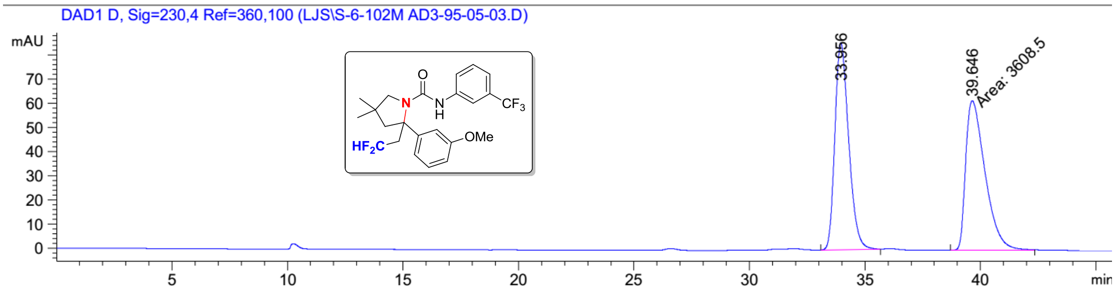

Signal 4: DAD1 D, Sig=230,4 Ref=360,100

Peak #	RetTime [min]	Type	Width [min]	Area [mAU*s]	Height [mAU]	Area %
1	33.956	BB	0.6428	3555.83887	85.57086	49.6325
2	39.646	MF	0.9729	3608.50195	61.81738	50.3675
Totals :				7164.34082	147.38824	

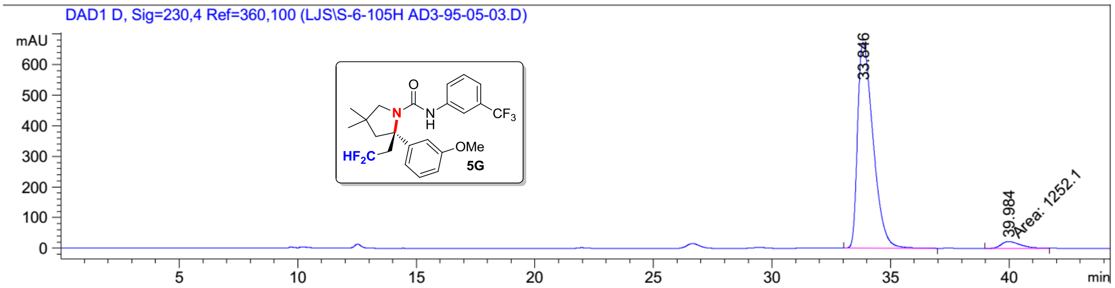

Signal 4: DAD1 D, Sig=230,4 Ref=360,100

Peak #	RetTime [min]	Type	Width [min]	Area [mAU*s]	Height [mAU]	Area %
1	33.846	BB	0.6969	3.04732e4	672.22363	96.0533
2	39.984	MF	0.9508	1252.10388	21.94824	3.9467
Totals :				3.17253e4	694.17187	

Supplementary Figure 198. HPLC traces for racemic and chiral product **5G**

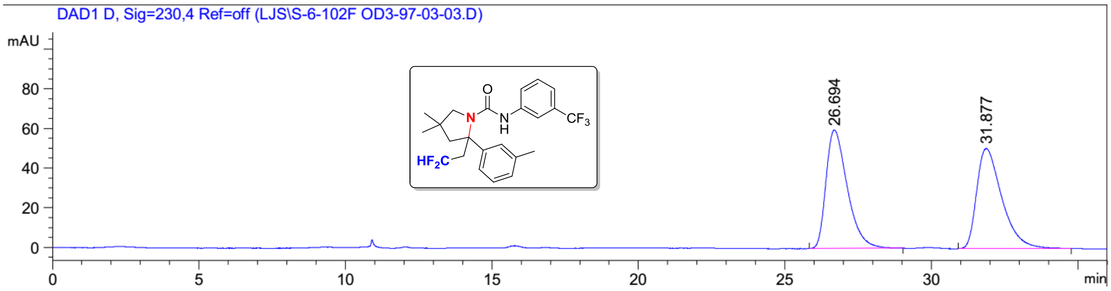

Signal 3: DAD1 D, Sig=230,4 Ref=off

Peak #	RetTime [min]	Type	Width [min]	Area [mAU*s]	Height [mAU]	Area %
1	26.694	BB	0.7237	3018.39380	59.72080	50.0360
2	31.877	BB	0.8542	3014.05322	50.50678	49.9640

Totals : 6032.44702 110.22758

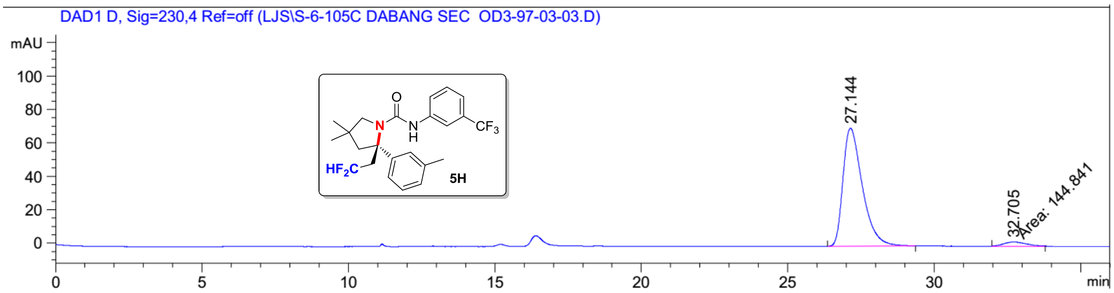

Signal 3: DAD1 D, Sig=230,4 Ref=off

Peak #	RetTime [min]	Type	Width [min]	Area [mAU*s]	Height [mAU]	Area %
1	27.144	BB	0.6937	3243.47607	70.64324	95.7253
2	32.705	MM	0.9172	144.84056	2.63191	4.2747

Totals : 3388.31664 73.27515

Supplementary Figure 199. HPLC traces for racemic and chiral product **5H**

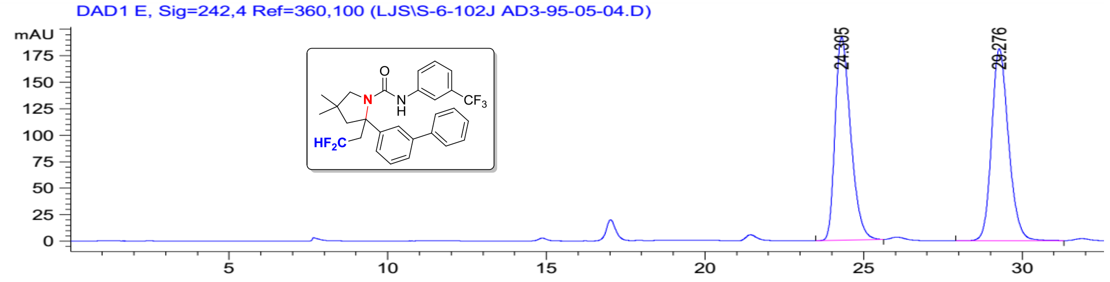

Signal 5: DAD1 E, Sig=242,4 Ref=360,100

Peak #	RetTime [min]	Type	Width [min]	Area [mAU*s]	Height [mAU]	Area %
1	24.305	BB	0.5277	6541.30176	191.88148	49.3372
2	29.276	BB	0.5659	6717.06250	181.43373	50.6628

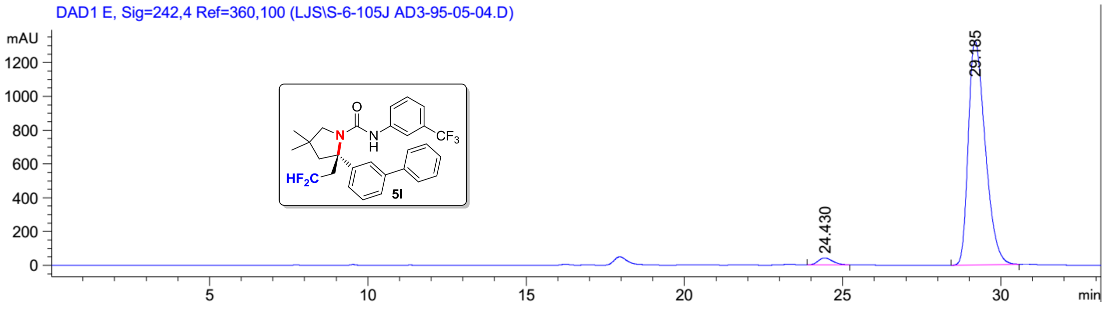

Signal 5: DAD1 E, Sig=242,4 Ref=360,100

Peak #	RetTime [min]	Type	Width [min]	Area [mAU*s]	Height [mAU]	Area %
1	24.430	BB	0.4891	1282.37683	40.97277	2.5406
2	29.185	BB	0.5749	4.91935e4	1325.83667	97.4594

Supplementary Figure 200. HPLC traces for racemic and chiral product **5l**

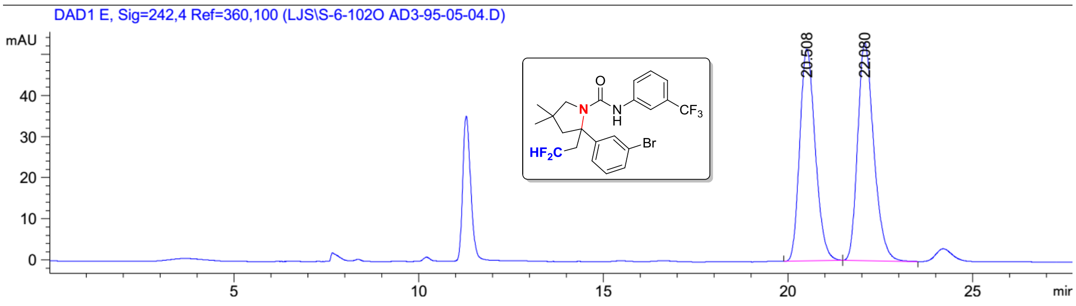

Signal 5: DAD1 E, Sig=242,4 Ref=360,100

Peak #	RetTime [min]	Type	Width [min]	Area [mAU*s]	Height [mAU]	Area %
1	20.508	BB	0.4519	1499.59351	51.47649	48.2506
2	22.080	BB	0.4644	1608.33240	52.94954	51.7494

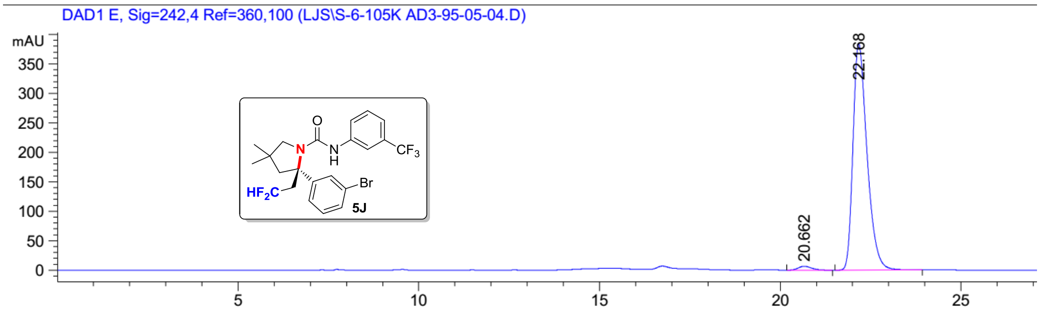

Signal 5: DAD1 E, Sig=242,4 Ref=360,100

Peak #	RetTime [min]	Type	Width [min]	Area [mAU*s]	Height [mAU]	Area %
1	20.662	BB	0.3918	164.43910	6.48494	1.5631
2	22.168	BB	0.4084	1.03558e4	384.22083	98.4369
Totals :				1.05203e4	390.70577	

Supplementary Figure 201. HPLC traces for racemic and chiral product **5J**

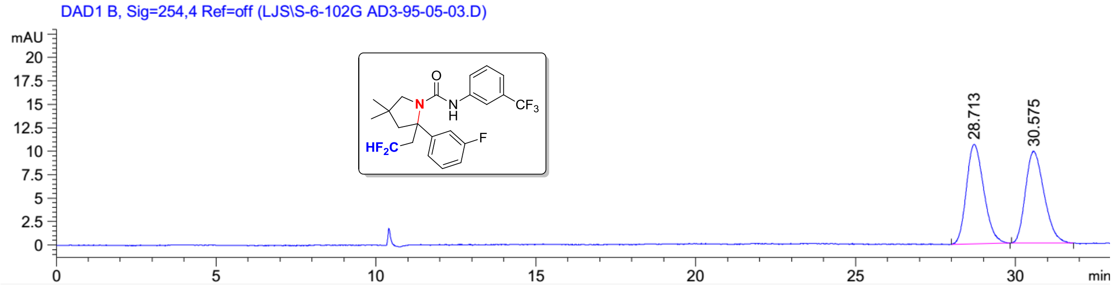

Signal 2: DAD1 B, Sig=254,4 Ref=off

Peak #	RetTime [min]	Type	Width [min]	Area [mAU*s]	Height [mAU]	Area %
1	28.713	BB	0.5417	402.19135	10.62396	50.2056
2	30.575	BB	0.5280	398.89719	9.81741	49.7944
Totals :				801.08853	20.44136	

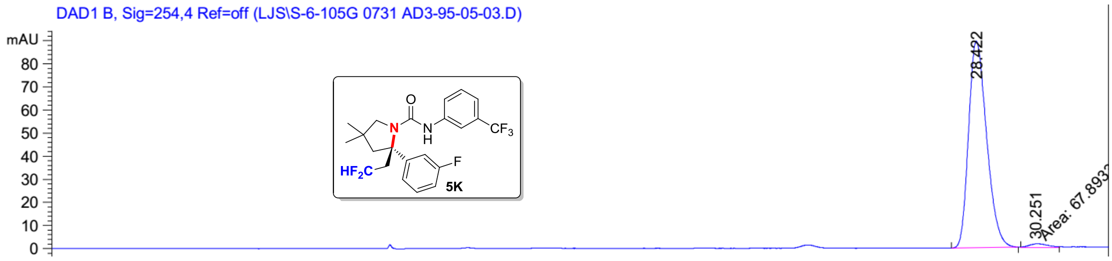

Signal 2: DAD1 B, Sig=254,4 Ref=off

Peak #	RetTime [min]	Type	Width [min]	Area [mAU*s]	Height [mAU]	Area %
1	28.422	BB	0.5897	3449.52417	89.49028	98.0698
2	30.251	MM	0.7044	67.89333	1.60649	1.9302
Totals :				3517.41750	91.09677	

Supplementary Figure 202. HPLC traces for racemic and chiral product **5K**

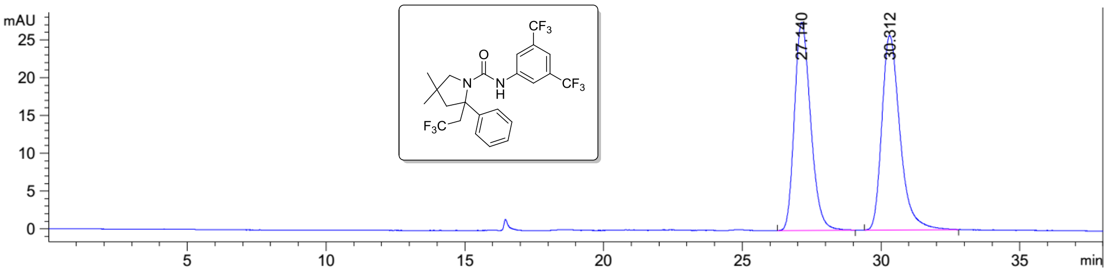

Signal 2: DAD1 B, Sig=254,4 Ref=off

Peak #	RetTime [min]	Type	Width [min]	Area [mAU*s]	Height [mAU]	Area %
1	27.140	BB	0.6191	1120.65088	27.51812	48.9626
2	30.312	BB	0.6759	1168.13635	25.71930	51.0374
Totals :				2288.78723	53.23742	

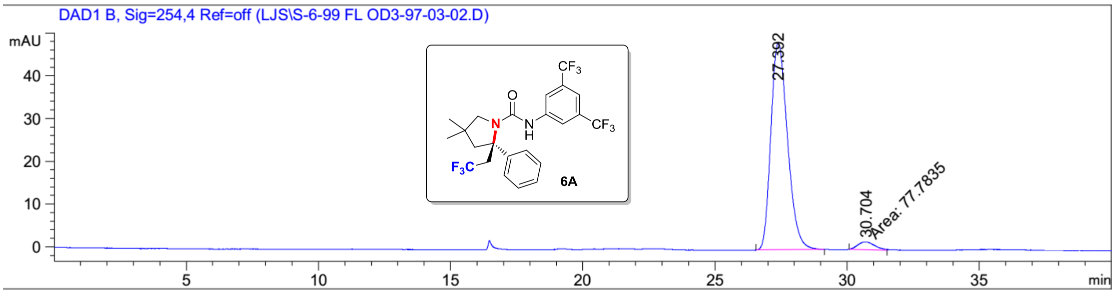

Signal 2: DAD1 B, Sig=254,4 Ref=off

Peak #	RetTime [min]	Type	Width [min]	Area [mAU*s]	Height [mAU]	Area %
1	27.392	BB	0.6755	2122.24951	48.40322	96.4644
2	30.704	MM	0.7180	77.78353	1.80551	3.5356
Totals :				2200.03304	50.20873	

Supplementary Figure 203. HPLC traces for racemic and chiral product **6A**

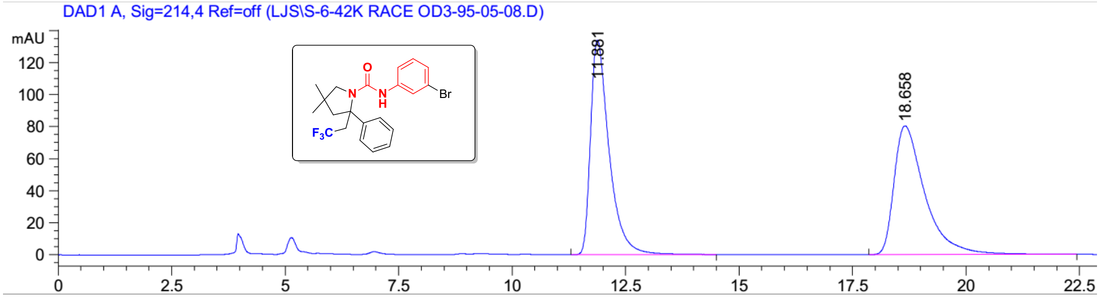

Signal 2: DAD1 B, Sig=254,4 Ref=off

Peak #	RetTime [min]	Type	Width [min]	Area [mAU*s]	Height [mAU]	Area %
1	11.881	BB	0.4268	966.87738	34.09987	50.2905
2	18.658	BB	0.6868	955.70776	20.46592	49.7095

Totals : 1922.58514 54.56579

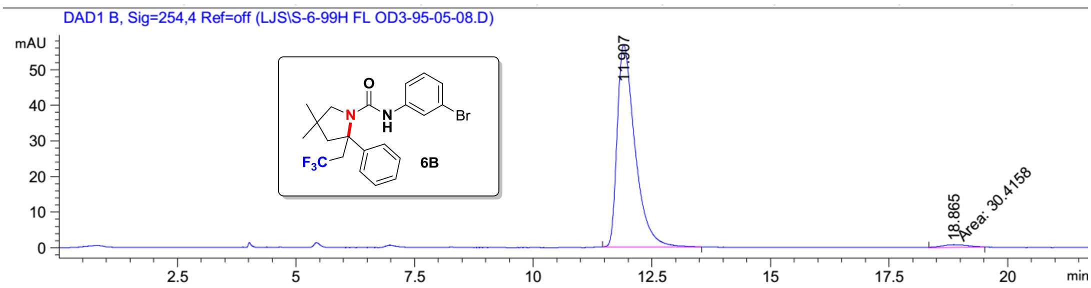

Signal 2: DAD1 B, Sig=254,4 Ref=off

Peak #	RetTime [min]	Type	Width [min]	Area [mAU*s]	Height [mAU]	Area %
1	11.907	BB	0.3899	1476.77991	56.70092	97.9820
2	18.865	MM	0.6654	30.41578	7.61827e-1	2.0180

Totals : 1507.19568 57.46275

Supplementary Figure 204. HPLC traces for racemic and chiral product **6B**

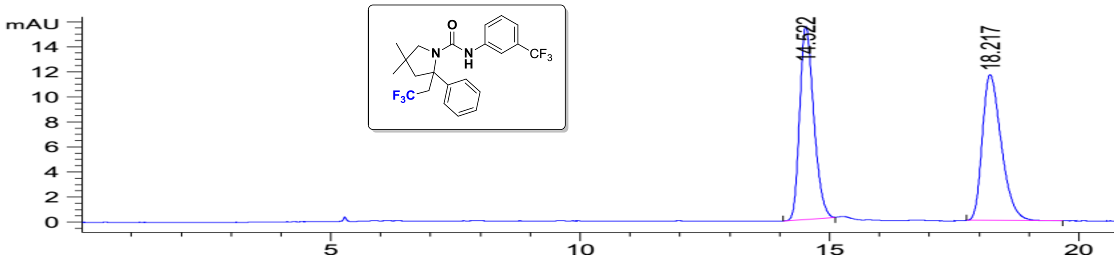

Signal 2: DAD1 B, Sig=254,4 Ref=off

Peak #	RetTime [min]	Type	Width [min]	Area [mAU*s]	Height [mAU]	Area %
1	14.522	BB	0.3010	302.49976	15.41201	49.4126
2	18.217	BB	0.4040	309.69238	11.65386	50.5874
Totals :				612.19214	27.06587	

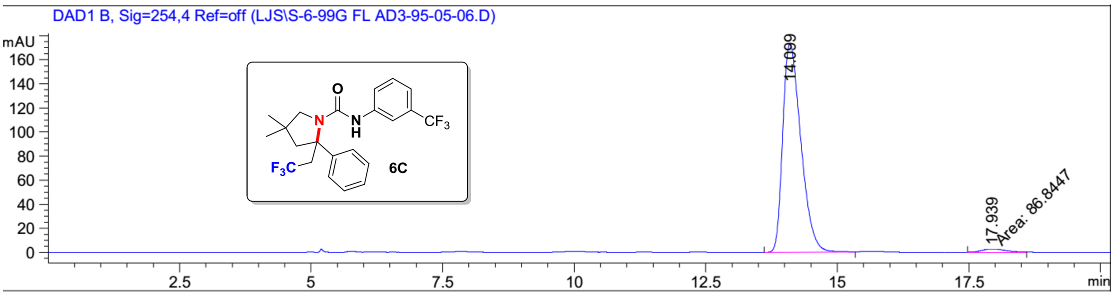

Signal 2: DAD1 B, Sig=254,4 Ref=off

Peak #	RetTime [min]	Type	Width [min]	Area [mAU*s]	Height [mAU]	Area %
1	14.099	BB	0.3725	4135.83594	173.25687	97.9434
2	17.939	FM	0.5076	86.84470	2.85146	2.0566
Totals :				4222.68063	176.10833	

Supplementary Figure 205. HPLC traces for racemic and chiral product **6C**

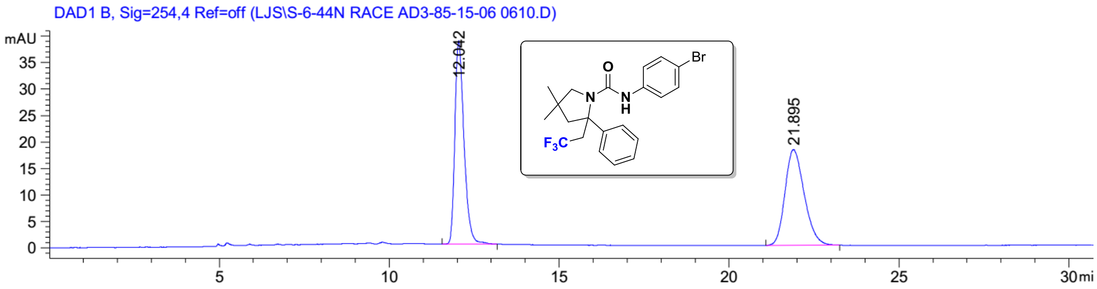

Signal 2: DAD1 B, Sig=254,4 Ref=off

Peak #	RetTime [min]	Type	Width [min]	Area [mAU*s]	Height [mAU]	Area %
1	11.855	BB	0.3346	3096.17554	144.16483	49.5979
2	22.305	BB	0.6841	3146.38086	70.30178	50.4021

Totals : 6242.55640 214.46661

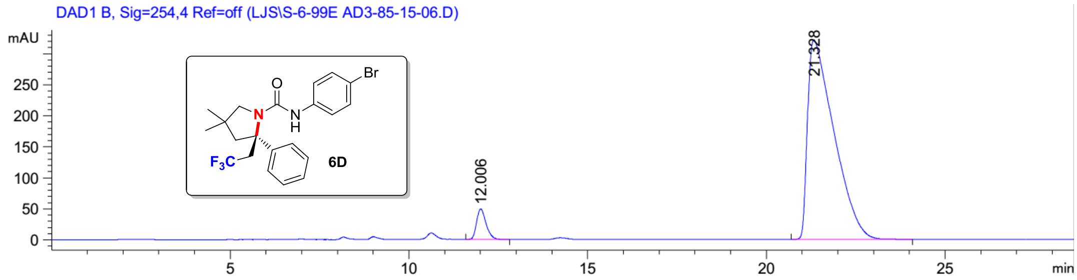

Signal 2: DAD1 B, Sig=254,4 Ref=off

Peak #	RetTime [min]	Type	Width [min]	Area [mAU*s]	Height [mAU]	Area %
1	12.006	BB	0.2817	901.44043	49.22179	5.1912
2	21.328	BB	0.7332	1.64632e4	321.61359	94.8088

Totals : 1.73646e4 370.83537

Supplementary Figure 206. HPLC traces for racemic and chiral product **6D**

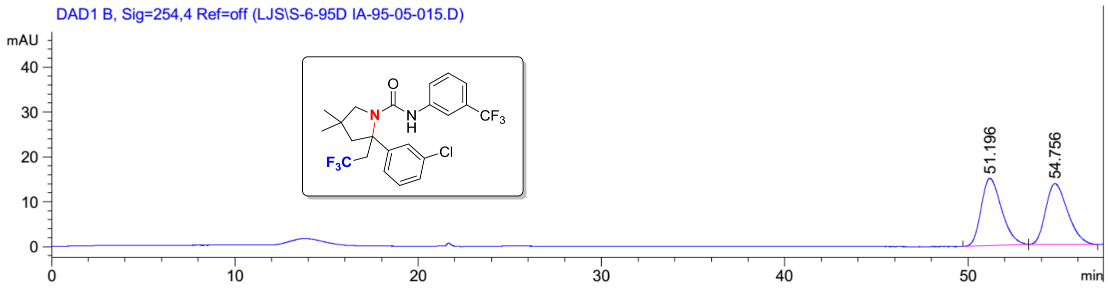

Signal 2: DAD1 B, Sig=254,4 Ref=off

Peak #	RetTime [min]	Type	Width [min]	Area [mAU*s]	Height [mAU]	Area %
1	51.196	BB	1.0740	1157.40601	14.96943	50.6465
2	54.756	BB	1.1334	1127.85547	13.54513	49.3535
Totals :				2285.26147	28.51456	

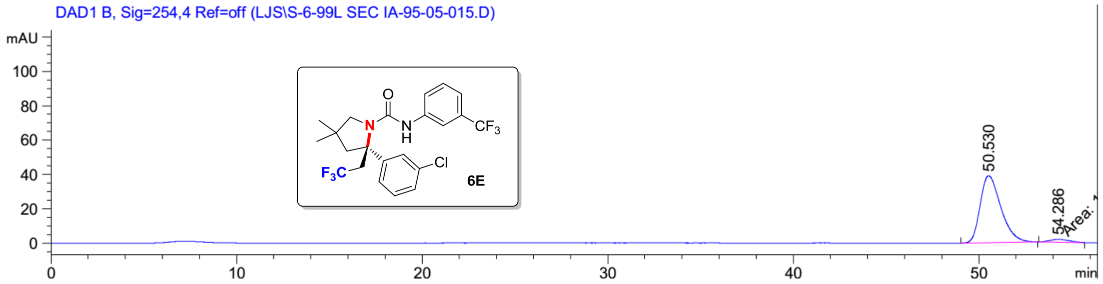

Signal 2: DAD1 B, Sig=254,4 Ref=off

Peak #	RetTime [min]	Type	Width [min]	Area [mAU*s]	Height [mAU]	Area %
1	50.530	BB	1.1314	3018.44897	39.06741	96.0179
2	54.286	MM	1.2550	125.18204	1.66238	3.9821
Totals :				3143.63102	40.72979	

Supplementary Figure 207. HPLC traces for racemic and chiral product **6E**

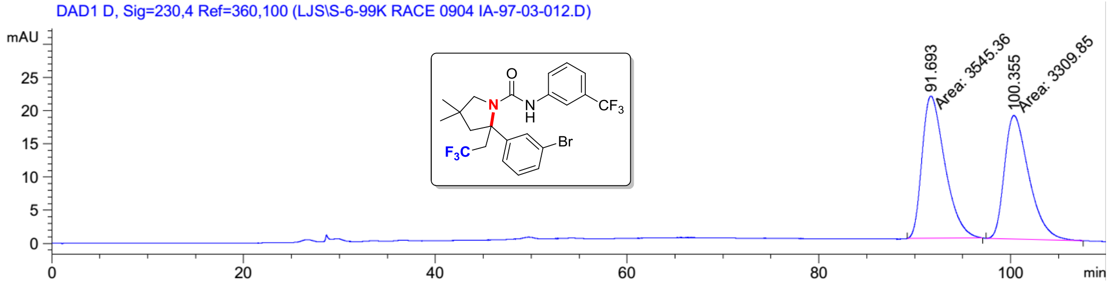

Signal 4: DAD1 D, Sig=230,4 Ref=360,100

Peak #	RetTime [min]	Type	Width [min]	Area [mAU*s]	Height [mAU]	Area %
1	91.693	MM	2.7609	3545.35767	21.40191	51.7177
2	100.355	MM	2.9621	3309.85449	18.62327	48.2823

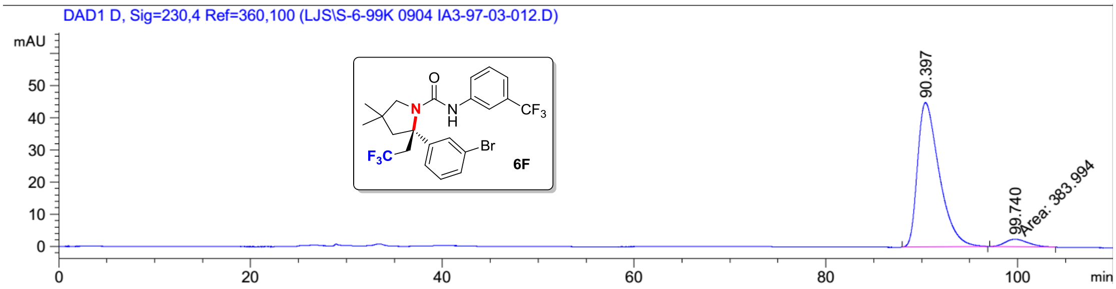

Signal 4: DAD1 D, Sig=230,4 Ref=360,100

Peak #	RetTime [min]	Type	Width [min]	Area [mAU*s]	Height [mAU]	Area %
1	90.397	BB	2.1876	7067.36133	44.91763	94.8467
2	99.740	MM	2.7712	383.99368	2.30940	5.1533
Totals :				7451.35501	47.22704	

Supplementary Figure 208. HPLC traces for racemic and chiral product **6F**

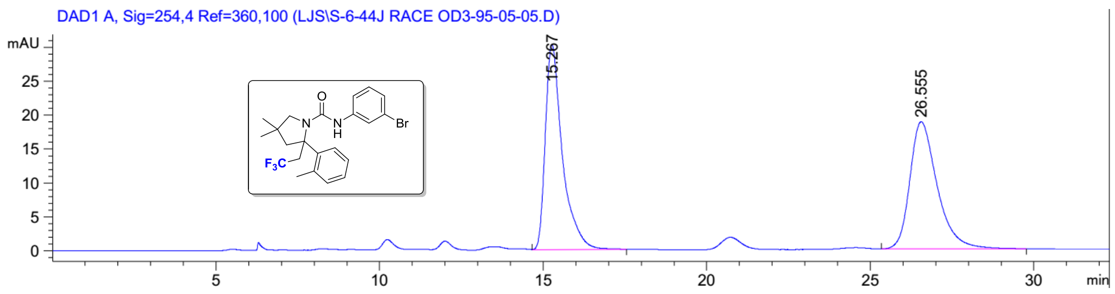

Signal 1: DAD1 A, Sig=254,4 Ref=360,100

Peak #	RetTime [min]	Type	Width [min]	Area [mAU*s]	Height [mAU]	Area %
1	15.267	BB	0.5389	1095.89917	30.23511	50.1119
2	26.555	BB	0.8261	1091.00635	18.77080	49.8881

Totals : 2186.90552 49.00591

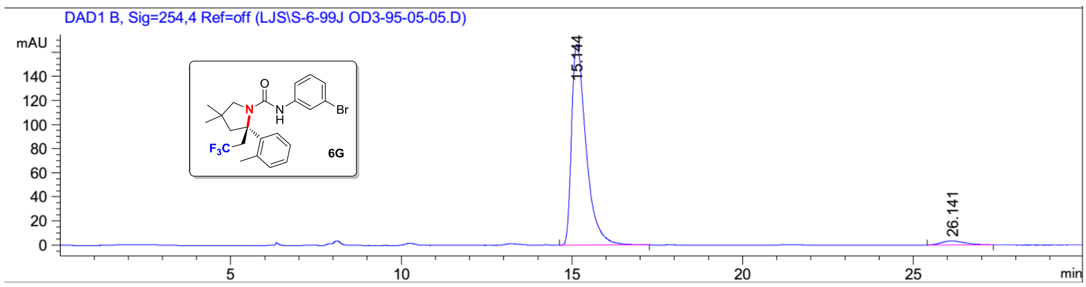

Signal 2: DAD1 B, Sig=254,4 Ref=off

Peak #	RetTime [min]	Type	Width [min]	Area [mAU*s]	Height [mAU]	Area %
1	15.144	BB	0.4477	4937.81982	166.65843	96.9776
2	26.141	BB	0.5554	153.89075	3.25614	3.0224

Totals : 5091.71057 169.91458

Supplementary Figure 209. HPLC traces for racemic and chiral product **6G**

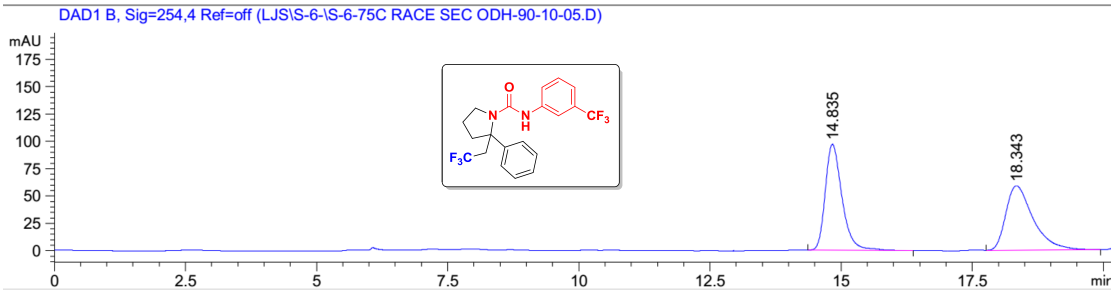

Signal 2: DAD1 B, Sig=254,4 Ref=off

Peak #	RetTime [min]	Type	Width [min]	Area [mAU*s]	Height [mAU]	Area %
1	14.835	BB	0.3396	2155.80127	96.88145	50.3031
2	18.343	BB	0.5438	2129.82007	58.91344	49.6969

Totals : 4285.62134 155.79489

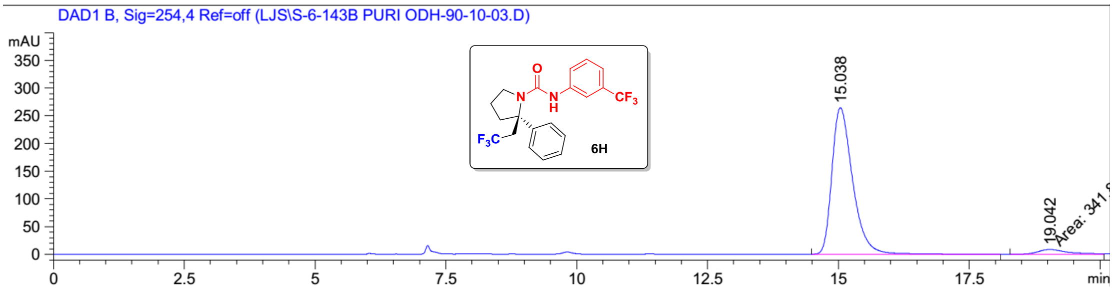

Signal 2: DAD1 B, Sig=254,4 Ref=off

Peak #	RetTime [min]	Type	Width [min]	Area [mAU*s]	Height [mAU]	Area %
1	15.038	BB	0.4191	7270.91748	264.16098	95.5101
2	19.042	MF	0.6711	341.80203	8.48827	4.4899

Totals : 7612.71951 272.64925

Supplementary Figure 210. HPLC traces for racemic and chiral product **6H**

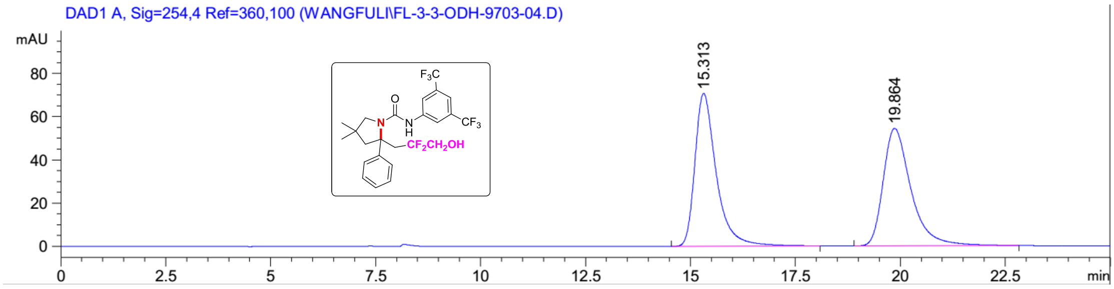

Signal 1: DAD1 A, Sig=254,4 Ref=360,100

Peak #	RetTime [min]	Type	Width [min]	Area [mAU*s]	Height [mAU]	Area %
1	15.313	BB	0.5343	2511.54956	70.72455	50.1288
2	19.864	BB	0.6905	2498.64233	54.33309	49.8712

Totals : 5010.19189 125.05764

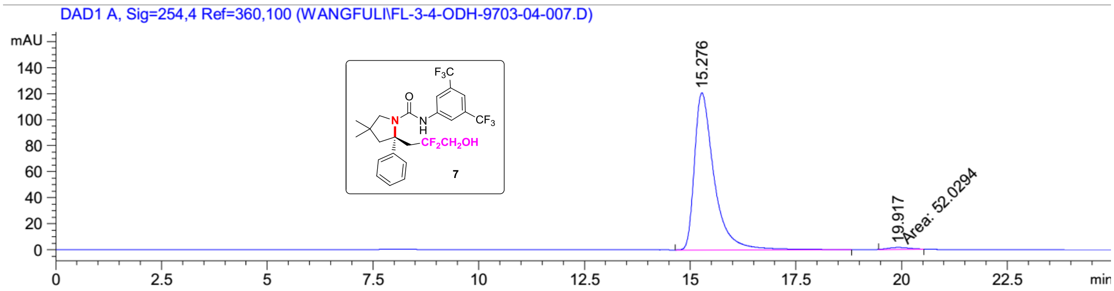

Signal 1: DAD1 A, Sig=254,4 Ref=360,100

Peak #	RetTime [min]	Type	Width [min]	Area [mAU*s]	Height [mAU]	Area %
1	15.276	BB	0.4853	3902.15869	120.64567	98.6842
2	19.917	MM	0.5844	52.02936	1.48379	1.3158

Totals : 3954.18805 122.12946

Supplementary Figure 211. HPLC traces for racemic and chiral product **7**

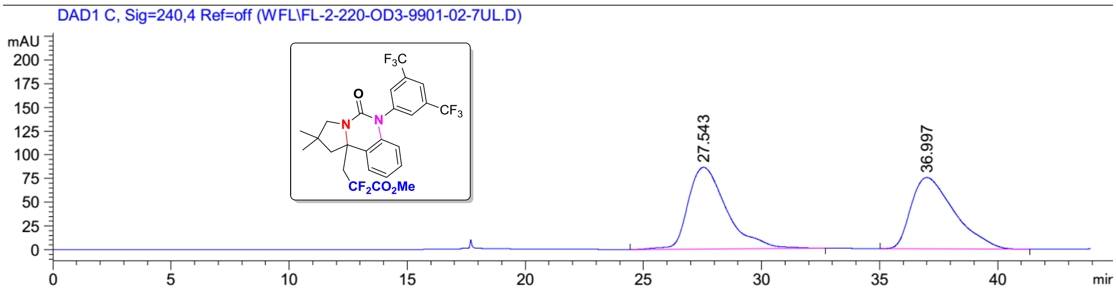

Signal 3: DAD1 C, Sig=240,4 Ref=off

Peak #	RetTime [min]	Type	Width [min]	Area [mAU*s]	Height [mAU]	Area %
1	27.543	BB	1.6996	9819.55664	86.29022	50.6422
2	36.997	BB	1.8452	9570.49902	75.17974	49.3578

Totals : 1.93901e4 161.46996

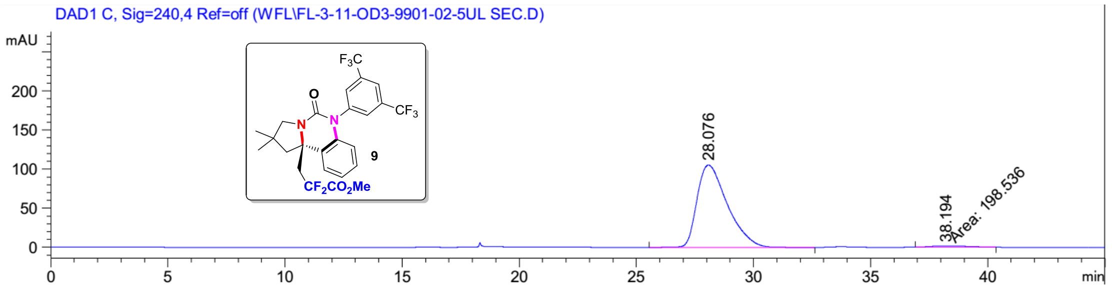

Signal 3: DAD1 C, Sig=240,4 Ref=off

Peak #	RetTime [min]	Type	Width [min]	Area [mAU*s]	Height [mAU]	Area %
1	28.076	BB	1.3581	9523.89648	105.41274	97.9580
2	38.194	MM	1.8988	198.53592	1.74263	2.0420

Totals : 9722.43240 107.15537

Supplementary Figure 212. HPLC traces for racemic and chiral product **9**

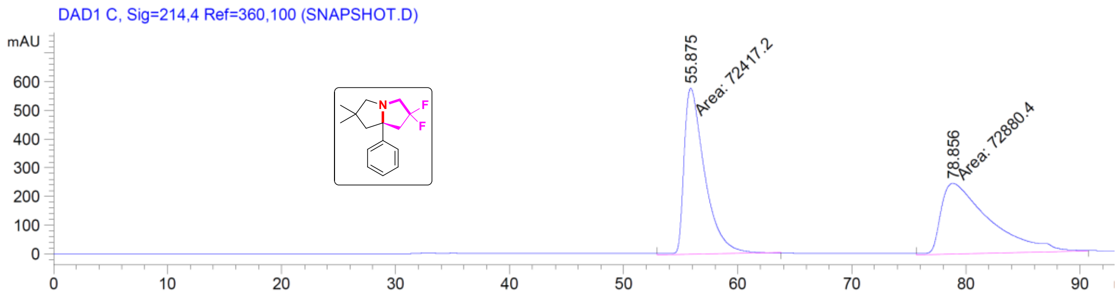

Signal 2: DAD1 C, Sig=214,4 Ref=360,100

Peak #	RetTime [min]	Type	Width [min]	Area [mAU*s]	Height [mAU]	Area %
1	55.875	MM	2.0890	7.24172e4	577.77454	49.8406
2	78.856	MM	4.9435	7.28804e4	245.71243	50.1594

Totals : 1.45298e5 823.48697

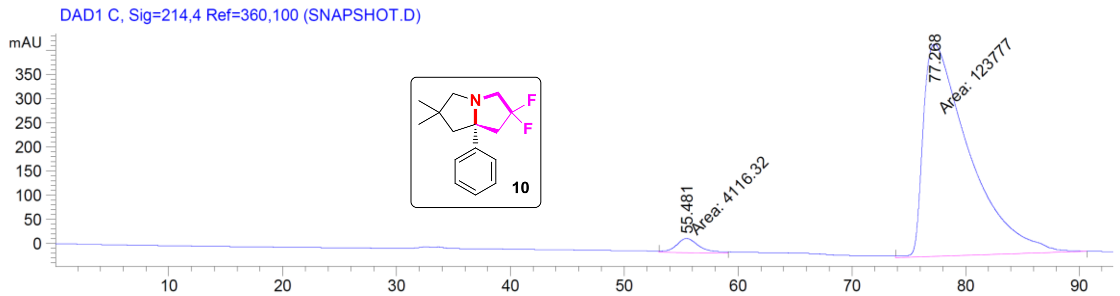

Signal 2: DAD1 C, Sig=214,4 Ref=360,100

Peak #	RetTime [min]	Type	Width [min]	Area [mAU*s]	Height [mAU]	Area %
1	55.481	MM	2.3563	4116.32275	29.11551	3.2185
2	77.268	MM	4.6850	1.23777e5	440.33621	96.7815

Totals : 1.27894e5 469.45172

Supplementary Figure 213. HPLC traces for racemic and chiral product **10**

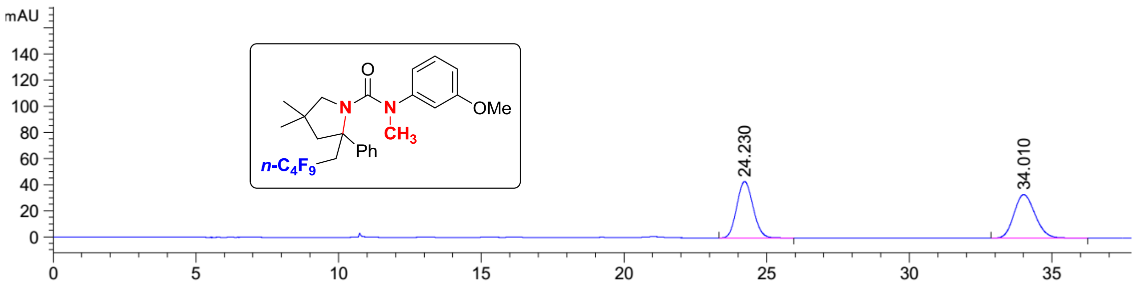

Signal 2: DAD1 B, Sig=254,4 Ref=off

Peak #	RetTime [min]	Type	Width [min]	Area [mAU*s]	Height [mAU]	Area %
1	24.230	BB	0.6180	1708.02612	43.13251	49.9733
2	34.010	BB	0.7892	1709.84998	33.29166	50.0267

Totals : 3417.87610 76.42417

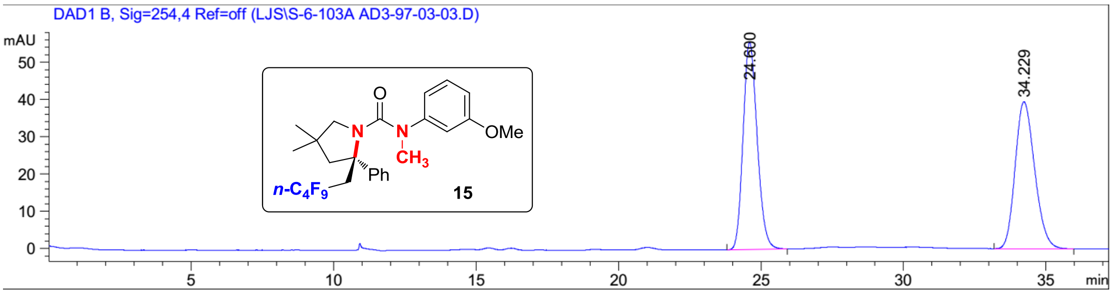

Signal 2: DAD1 B, Sig=254,4 Ref=off

Peak #	RetTime [min]	Type	Width [min]	Area [mAU*s]	Height [mAU]	Area %
1	24.600	BB	0.5368	1908.92712	55.56793	50.3778
2	34.229	BB	0.7339	1880.29944	39.46050	49.6222

Totals : 3789.22656 95.02843

Supplementary Figure 214. HPLC traces for racemic and chiral product **15**

Supplementary Methods

All reactions were carried out under argon atmosphere using Schlenk techniques. Reagents were purchased at the highest commercial quality and used without further purification, unless otherwise stated. CuBr and Ag_2CO_3 were purchased from Sigma-Aldrich. Chiral phosphoric acid (**CPA**) was purchased from Daicel Chiral Technologies (China). Difluoromethylsulfonyl chloride and methyl 2-(chlorosulfonyl) -2,2-difluoroacetate were purchased from 9dingchem (China). Ethyl isobutyrate (*i*-$PrCO_2Et$) was purchased from Adamas-beta® (Product Code: 91931B) and transferred under an argon atmosphere. Analytical thin layer chromatography (TLC) was performed on precoated silica gel 60 GF254 plates. Flash column chromatography was performed using Tsingdao silica gel (60, particle size 0.040-0.063 mm). Visualization on TLC was achieved by use of UV light (254 nm) or iodine. NMR spectra were recorded on Bruker DRX-500 and DPX 400 spectrometer at 400 or 500 MHz for ^{1}H NMR, 100 or 126 MHz for ^{13}C NMR and 376 MHz for ^{19}F NMR in $CDCl_3$, Acetone-d_6 with tetramethylsilane (TMS) as internal standard. The chemical shifts are expressed in ppm and coupling constants are given in Hz. Data for ^{1}H NMR are recorded as follows: chemical shift (ppm), multiplicity (s, singlet; d, doublet; t, triplet; q, quarter; p, pentet, m, multiplet; br, broad), coupling constant (Hz), integration. Data for ^{13}C NMR are reported in terms of chemical shift (δ, ppm). Mass spectrometric data were obtained using Bruker Apex IV RTMS. Enantiomeric excess (ee) was determined using Agilent high-performance liquid chromatography (HPLC) with a Hatachi detector (λ = 254, 242, 230 or 214 nm). Column conditions are reported in the experimental section below. Absolute configuration of a product was determined by X-ray analysis.

General procedure for the synthesis of substrates:

Substrates **1** with *N*-aryl urea groups was synthesized according to the procedures previously reported.[1]

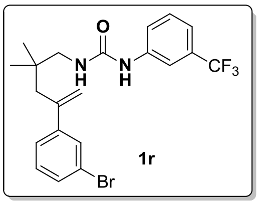

1-(4-(3-bromophenyl)-2,2-dimethylpent-4-en-1-yl)-3-(3-(trifluoromethyl)phenyl)urea (1r)

^{1}H NMR (500 MHz, $CDCl_3$) δ 7.88 (br, 1H), 7.57 (s, 1H), 7.47 (s, 1H), 7.46-7.38 (m, 1H), 7.38-7.33 (m, 1H), 7.29 (d, *J* = 8.0 Hz, 1H), 7.22 (d, *J* = 8.0 Hz, 2H), 7.12 (t, *J* = 8.0 Hz, 1H), 5.87-5.52 (br, 1H), 5.23 (s, 1H), 5.02 (s, 1H), 3.00 (d, *J* = 6.0 Hz, 2H), 2.40 (s, 2H), 0.72 (s, 6H).

^{13}C NMR (126 MHz, $CDCl_3$) δ 156.4, 145.7, 145.2, 134.5, 139.8, 131.6 (q, *J* = 32.5 Hz), 129.9, 129.8, 127.7, 126.8, 124.9, 124.2 (q, *J* = 275.2 Hz), 123.0, 119.7, 118.7, 116.6, 50.5, 45.1, 36.0, 25.7.

^{19}F NMR (376 MHz, $CDCl_3$) δ -62.7 (s, 3F).

HRMS (ESI) m/z calcd. for $C_{21}H_{23}BrF_3N_2O$ $[M+H]^+$ 455.0946, found 455.0940.

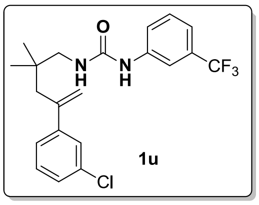

1-(4-(3-chlorophenyl)-2,2-dimethylpent-4-en-1-yl)-3-(3-(trifluoromethyl)phenyl)urea (1u)

^{1}H NMR (500 MHz, $CDCl_3$) δ 7.56-7.41 (m, 3H), 7.36-7.29 (m, 2H), 7.26-7.15 (m, 4H), 5.46 (br s, 1H), 5.25 (s, 1H), 5.04 (s, 1H), 3.01 (d, *J* = 6.0 Hz, 2H), 2.42 (s, 2H), 0.74 (s, 6H).

^{13}C NMR (126 MHz, $CDCl_3$) δ 156.1, 145.1, 145.0, 139.5, 134.2, 131.6 (q, *J* = 34.4 Hz), 129.6, 129.5, 127.3, 126.5, 124.6, 123.9 (q, *J* = 272.4 Hz), 122.7, 119.5, 118.4, 116.3 (d, *J* = 4.6 Hz), 50.2, 44.8, 35.8, 25.4.

^{19}F NMR (376 MHz, $CDCl_3$) δ -62.8 (s, 3F).

HRMS (ESI) m/z calcd. for $C_{21}H_{23}ClF_3N_2O$ $[M+H]^+$ 411.1451, found 411.1454.

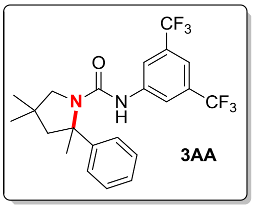

***N*-(3,5-bis(trifluoromethyl)phenyl)-2,4,4-trimethyl-2-phenylpyrrolidine-1-carboxamide (3AA)**

^{1}H NMR (500 MHz, $CDCl_3$) δ 7.55 (br s, 2H), 7.50-7.39 (m, 5H), 7.35 (br s, 1H), 6.34 (br s, 1H), 3.74 (d, *J* = 10.5 Hz, 1H), 3.65 (d, *J* = 10.5 Hz, 1H), 2.28 (d, *J* = 13.5 Hz, 1H), 2.19 (d, *J* = 13.5 Hz, 1H), 1.97 (s, 3H), 1.28 (s, 3H), 1.13 (s, 3H).

^{13}C NMR (126 MHz, $CDCl_3$) δ 153.8, 147.3, 141.1, 132.5 (q, *J* = 33.2 Hz), 129.9, 128.2, 126.1, 123.8 (q, *J* = 273.2 Hz), 119.4, 116.3 (p, *J* = 3.9 Hz), 67.3, 62.5, 60.8, 36.4, 29.5, 29.3, 27.8.

^{19}F NMR (376 MHz, $CDCl_3$) δ -63.1 (s, 6F).

HRMS (ESI) m/z calcd. for $C_{22}H_{23}F_6N_2O$ $[M+H]^+$ 445.1715, found 445.1716.

General procedure A: direct asymmetric intramolecular radical aminoperfluoroalkylation of alkenes

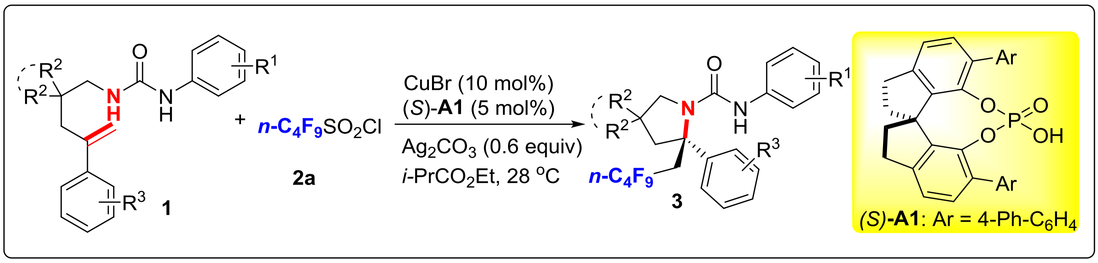

Under argon, an oven-dried resealable Schlenk tube equipped with a magnetic stir bar was charged with urea substrate **1** (0.1 mmol, 1.0 equiv), CuBr (1.43 mg, 0.01 mmol, 10 mol%), Ag_2CO_3 (16.56 mg, 0.06 mmol, 0.6 equiv) chiral phosphoric acid (*S*)-**A1** (3.1 mg, 0.005 mmol, 5 mol%), *n*-$C_4F_9SO_2Cl$ (**2a**) (38.15 mg, 0.12 mmol, 1.2 equiv) and ethyl isobutyrate (1.0 mL) at 28 °C, and the sealed tube was then stirred at 28 °C. Upon completion (monitored by TLC), the reaction mixture was directly purified by a silica gel chromatography [eluent: petroleum ether/EtOAc = 100/0-5/1, using petroleum ether (100%) to remove the solvent (ethyl isobutyrate) at first] to afford the desired product **3**.

Note: Since the reaction is sensitive to water and air, Schlenk tube and the reagents must be dried prior to use.

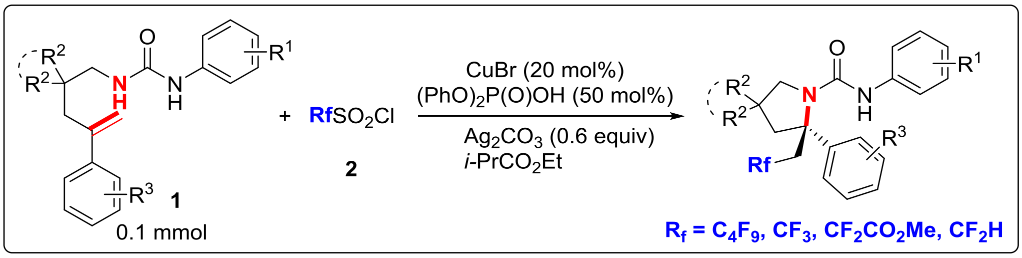

The racemic products were prepared following the same procedure described above using urea substrate **1** (0.1 mmol, 1.0 equiv), CuBr (2.86 mg, 0.02 mmol, 20 mol%), Ag_2CO_3 (16.56 mg, 0.06 mmol, 0.6 equiv) and diphenyl phosphate (12.5 mg, 0.05 mmol, 50 mol%) as catalyst at 28 or 40 °C in ethyl isobutyrate (1.0 mL) for 12-48 h. Upon completion (monitored by TLC), the solvent was removed *in vacuo*, and the residue was purified by a silica gel column chromatography (eluent: petroleum ether/EtOAc = 100/0-5/1) to give the desired products.

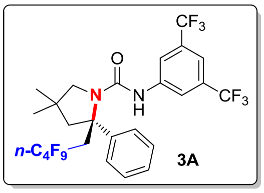

(*R*)-*N*-(3,5-bis(trifluoromethyl)phenyl)-4,4-dimethyl-2-(2,2,3,3,4,4,5,5,5-nonafluoropentyl)-2-phenyl-pyrrolidine-1-carboxamide (3A)

HPLC analysis: Chiralcel ID (hexane/*i*-PrOH = 97/03, flow rate 0.2 mL/min, λ = 230 nm), t_R (major) = 17.57 min, t_R (minor) = 20.36 min.

^{1}H NMR (500 MHz, $CDCl_3$) δ 7.83 (s, 2H), 7.45 (s, 1H), 7.39-7.29 (m, 4H), 7.26-7.24 (m, 1H), 6.82 (s, 1H), 3.82 (dd, *J* = 35.0, 14.0 Hz, 1H), 3.59-3.42 (m, 2H),

2.85-2.83 (m, 1H), 2.78 (d, J = 13.5 Hz, 1H), 2.24 (d, J = 13.5 Hz, 1H), 1.12 (s, 3H), 0.87 (s, 3H).

^{13}C NMR (126 MHz, $CDCl_3$) δ 153.2, 145.5, 140.2, 131.9 (q, J = 33.3 Hz), 128.3, 127.1, 125.7, 123.1 (q, J = 272.7 Hz), 119.6 (d, J = 4.0 Hz), 116.3 (p, J = 3.8 Hz), 121.2-106.8 (m), 68.9, 61.2, 53.1, 36.5, 36.2 (t, J = 18.4 Hz), 28.2.

^{19}F NMR (376 MHz, $CDCl_3$) δ -63.2 (s, 6F), -81.2 (t, J = 9.8 Hz, 3F), -107.3 (AB, d, J_{F-F} = 270.7 Hz, 1F), -117.1 (AB, d, J_{F-F} = 271.9 Hz, 1F), -124.5 (s, 2F), -125.7 ~ -125.8 (m, 2F).

HRMS (ESI) m/z calcd. for $C_{26}H_{22}F_{15}N_2O$ $[M+H]^+$ 663.1493, found 663.1487.

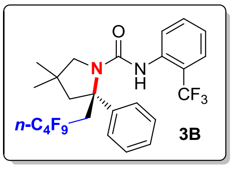

(*R*)-4,4-dimethyl-2-(2,2,3,3,4,4,5,5,5-nonafluoropentyl)-2-phenyl-*N*-(2-(trifluoromethyl)phenyl)pyrrolidine-1-carboxamide (3B)

HPLC analysis: Chiralcel OD3 (hexane/*i*-PrOH = 97/3, flow rate 0.2 mL/min, λ = 254 nm), t_R (major) = 19.23 min, t_R (minor) = 23.88 min.

^{1}H NMR (500 MHz, $CDCl_3$) δ 8.11 (d, J = 8.5 Hz, 1H), 7.61 (d, J = 8.0 Hz, 1H), 7.53 (t, J = 8.0 Hz, 1H), 7.38-7.35 (m, 4H), 7.28-7.23 (m, 1H), 7.17 (t, J = 7.5 Hz, 1H), 6.85 (s, 1H), 3.84 (dd, J = 36.5, 16.0 Hz, 1H), 3.57 (d, J = 8.0 Hz, 1H), 3.51 (d, J = 8.0 Hz, 1H), 2.88-2.71 (m, 2H), 2.27 (d, J = 13.5 Hz, 1H), 1.21 (s, 3H), 0.94 (s, 3H).

^{13}C NMR (126 MHz, $CDCl_3$) δ 153.2, 145.8, 136.6 (d, J = 2.1 Hz), 132.8, 128.3, 126.9, 125.9, 125.8 (q, J = 5.4 Hz), 124.5 (q, J = 272.7 Hz), 124.4, 123.1, 119.6 (q, J = 29.1 Hz), 121.5-106.5 (m), 68.6, 60.8, 53.2, 36.4, 36.2 (t, J = 18.2 Hz), 28.4, 28.3.

^{19}F NMR (376 MHz, $CDCl_3$) δ -60.8 (s, 3F), -77.6 ~ -84.5 (m, 3F), -107.2 (AB, d, J_{F-F} = 269.6 Hz, 1F), -116.9 (AB, d, J_{F-F} = 269.6 Hz, 1F). -124.2 ~ -124.7 (m, 2F), -125.5 ~ -125.9 (m, 2F).

HRMS (ESI) m/z calcd. for $C_{25}H_{23}F_{12}N_2O$ $[M+H]^+$ 595.1619, found 595.1613.

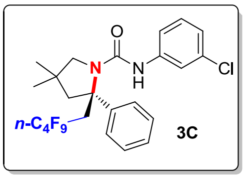

(*R*)-*N*-(3-chlorophenyl)-4,4-dimethyl-2-(2,2,3,3,4,4,5,5,5-nonafluoropentyl)-2-phenylpyrrolidine-1-carboxamide (3C)

HPLC analysis: Chiralcel OD3 (hexane/*i*-PrOH = 90/10, flow rate 0.3 mL/min, λ = 254 nm), t_R (major) = 13.55 min, t_R (minor) = 16.06 min.

^{1}H NMR (500 MHz, $CDCl_3$) δ 7.54 (s, 1H), 7.38-7.36 (m, 4H), 7.28-7.24 (m, 2H), 7.20 (t, J = 8.0 Hz, 1H), 7.03 (d, J = 8.0 Hz, 1H), 6.45 (s, 1H), 3.82 (dd, J = 36.0, 14.5 Hz, 1H), 3.55-3.50 (m, 2H), 2.95-2.73 (m, 2H), 2.25 (d, J = 13.5 Hz, 1H), 1.19 (s, 3H), 0.92 (s, 3H).

^{13}C NMR (126 MHz, $CDCl_3$) δ 153.6, 146.2, 140.4, 134.8, 130.1, 128.6, 127.2, 126.2, 123.4, 120.3, 118.2, 121.2-106.8 (m), 68.9, 61.4, 53.5, 36.8, 36.6 (t, J = 18.5 Hz), 28.7, 28.6.

^{19}F NMR (376 MHz, $CDCl_3$) δ -80.8 ~ -81.3 (m, 3F), -107.7 (AB, d, J_{F-F} = 269.2 Hz, 1F), -116.4 (AB, d, J_{F-F} = 268.8 Hz, 1F), -124.0 (s, 2F), -125.6 ~ -125.7 (m, 2F).

HRMS (ESI) m/z calcd. for $C_{24}H_{23}ClF_9N_2O$ $[M+H]^+$ 561.1355, found 561.1350.

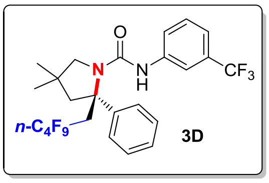

(*R*)-4,4-dimethyl-2-(2,2,3,3,4,4,5,5,5-nonafluoro-pentyl)-2-phenyl-*N*-(3-(trifluoromethyl)phenyl)-pyrrolidine-1-carboxamide (3D)

HPLC analysis: Chiralcel OD3 (hexane/*i*-PrOH = 96/4, flow rate 0.3 mL/min, λ = 230 nm), t_R (major) = 15.55 min, t_R (minor) = 21.13 min.

^{1}H NMR (500 MHz, $CDCl_3$) δ 7.62 (s, 2H), 7.40-7.30 (m, 5H), 7.30-7.19 (m, 2H), 6.56 (s, 1H), 3.88-3.78 (m, 1H), 3.55-3.48 (m, 2H), 2.92-2.62 (m, 2H), 2.24 (d, *J* = 13.5 Hz, 1H), 1.16 (s, 3H), 0.89 (s, 3H).

^{13}C NMR (126 MHz, $CDCl_3$) δ 153.4, 145.8, 139.3, 131.1 (q, *J* = 32.3 Hz), 129.3, 128.2, 126.9, 125.8, 124.0 (q, *J* = 272.4 Hz), 123.1, 119.6 (q, *J* = 3.8 Hz), 116.6 (q, *J* = 4.0 Hz), 121.1-106.5 (m), 68.6, 61.1, 53.1, 36.4, 36.2 (t, *J* = 18.2 Hz), 28.2, 28.2.

^{19}F NMR (376 MHz, $CDCl_3$) δ -62.7 (s, 3F), -81.1 (t, *J* = 9.8 Hz, 3F), -107.4 (AB, d, $J_{F\text{-}F}$ = 270.3 Hz, 1F), -116.7 (AB, d, $J_{F\text{-}F}$ = 270.7 Hz, 1F), -124.5 (s, 2F), -125.1 ~ -126.2 (m, 2F).

HRMS (ESI) m/z calcd. for $C_{25}H_{23}F_{12}N_2O$ $[M+H]^+$ 595.1619, found 595.1613.

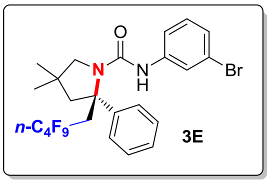

(*R*)-*N*-(3-bromophenyl)-4,4-dimethyl-2-(2,2,3,3,4,4,5,5,5-nonafluoropentyl)-2-phenylpyrrolidine-1-carboxamide (3E)

HPLC analysis: Chiralcel OD3 (hexane/*i*-PrOH = 95/05, flow rate 0.4 mL/min, λ = 214 nm), t_R (major) = 12.96 min, t_R (minor) = 21.52 min.

^{1}H NMR (500 MHz, $CDCl_3$) δ 7.67 (s, 1H), 7.42-7.35 (m, 4H), 7.33-7.31 (m, 1H), 7.28-7.24 (m, 1H), 7.21-7.07 (m, 2H), 6.43 (s, 1H), 3.82 (dd, *J* = 36.5, 15.0 Hz, 1H), 3.58-3.45 (m, 2H), 2.91-2.72 (m, 2H), 2.25 (d, *J* = 13.5 Hz, 1H), 1.19 (s, 3H), 0.92 (s, 3H).

^{13}C NMR (126 MHz, $CDCl_3$) δ 153.6, 146.2, 140.5, 130.4, 128.6, 127.2, 126.4, 126.2, 123.1, 122.8, 118.7, 121.5-106.5 (m), 68.9, 61.4, 53.5, 36.8, 36.6 (t, *J* = 18.3 Hz), 28.7, 28.6.

^{19}F NMR (376 MHz, $CDCl_3$) δ -81.0 (d, *J* = 9.8 Hz, 3F), -107.7 (AB, d, $J_{F\text{-}F}$ = 269.2 Hz, 1F), -116.4 (AB, d, $J_{F\text{-}F}$ = 268.8 Hz, 1F), -122.4 (s, 2F), -124.9 ~ -126.5 (m, 2F).

HRMS (ESI) m/z calcd. for $C_{24}H_{23}BrF_9N_2O$ $[M+H]^+$ 605.0850, found 605.0845.

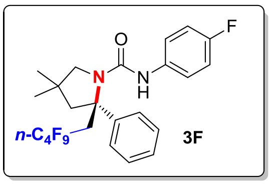

(*R*)-*N*-(4-fluorophenyl)-4,4-dimethyl-2-(2,2,3,3,4,4,5,5,5-nonafluoropentyl)-2-phenylpyrrolidine-1-carboxamide (3F)

HPLC analysis: Chiralcel OD3 (hexane/*i*-PrOH = 96/04, flow rate 0.2 mL/min, λ = 214 nm), t_R (major) = 29.14 min, t_R (minor) = 36.66 min.

^{1}H NMR (500 MHz, $CDCl_3$) δ 7.40-7.32 (m, 6H), 7.27-7.23 (m, 1H), 7.02-6.97 (m, 2H), 6.35 (s, 1H), 3.83 (dd, *J* = 37.5, 15.0 Hz, 1H), 3.58-3.46 (m, 2H), 2.89-2.73 (m, 2H), 2.26 (d, *J* = 13.5 Hz, 1H), 1.19 (s, 3H), 0.92 (s, 3H).

^{13}C NMR (126 MHz, $CDCl_3$) δ 159.3 (d, *J* = 241.8 Hz), 154.1, 146.4, 135.0 (d, *J* = 2.7 Hz), 128.5, 127.1, 126.3, 122.5 (d, *J* = 7.9 Hz), 115.7 (d, *J* = 22.3 Hz), 121.5-106.9 (m), 68.8, 61.4, 53.5, 36.7, 36.6 (t, *J* = 18.9 Hz), 28.7, 28.6.

^{19}F NMR (376 MHz, $CDCl_3$) δ -81.0 ~ -81.1 (m, 3F), -107.5 (AB, d, J_{F-F} = 268.8 Hz, 1F), -116.6 (AB, d, J_{F-F} = 267.7 Hz, 1F), -119.9 (m, 1F), -124.4 (s, 2F), -125.5 ~ -125.8 (m, 2F).
HRMS (ESI) m/z calcd. for$C_{24}H_{23}F_{10}N_2O$ $[M+H]^+$ 545.1651, found 545.1645.

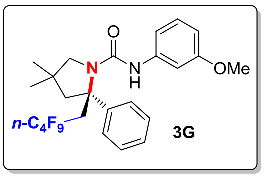

3G

(*R*)-*N*-(3-methoxyphenyl)-4,4-dimethyl-2-(2,2,3,3,4,4,5,5,5-nonafluoropentyl)-2-phenyl-pyrrolidine-1-carboxamide (3G)
HPLC analysis: Chiralcel OD3 (hexane/*i*-PrOH = 90/10, flow rate 0.4 mL/min, λ = 254 nm), t_R (major) = 12.06 min, t_R (minor) = 17. 68 min.
^{1}H NMR (500 MHz, $CDCl_3$) δ 7.42-7.33 (m, 4H), 7.28-7.24 (m, 1H), 7.22-7.17 (m, 2H), 6.89 (d, *J* = 8.0 Hz, 1H), 6.63 (dd, *J* = 8.5, 2.5 Hz, 1H), 6.41 (s, 1H), 3.94-3.80 (m, 4H), 3.60-3.46 (m, 2H), 2.90-2.73 (m, 2H), 2.26 (d, *J* = 13.5 Hz, 1H), 1.20 (s, 3H), 0.92 (s, 3H).
^{13}C NMR (126 MHz, $CDCl_3$) δ 160.2, 153.6, 146.0, 140.2, 129.5, 128.2, 126.8, 126.0, 112.1, 109.2, 121.2-106.2 (m), 105.6, 68.5, 61.1, 55.2, 53.2 (d, *J* = 5.5 Hz), 36.4, 36.3 (t, *J* = 18.2 Hz), 28.4, 28.3.
^{19}F NMR (376 MHz, $CDCl_3$) δ -80.7 ~ -81.4 (m, 3F), -107.2 (AB, d, J_{F-F} = 269.2 Hz, 1F), -116.7 (AB, d, J_{F-F} = 268.8 Hz, 1F), -124.4 (s, 2F), -125.3 ~ -125.9 (m, 2F).
HRMS (ESI) m/z calcd. for $C_{25}H_{26}F_9N_2O_2$ $[M+H]^+$ 557.1851, found 557.1845.

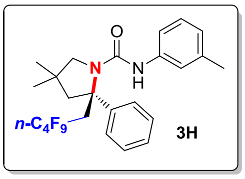

3H

(*R*)-4,4-dimethyl-2-(2,2,3,3,4,4,5,5,5-nonafluoropentyl)-2-phenyl-*N*-(m-tolyl)pyrrolidine-1-carboxamide (3H)
HPLC analysis: Chiralcel OD3 (hexane/*i*-PrOH = 97/03, flow rate 0.3 mL/min, λ = 230 nm), t_R (major) = 19.19 min, t_R (minor) = 36.68 min.
^{1}H NMR (500 MHz, $CDCl_3$) δ 7.41-7.32 (m, 5H), 7.26 (tt, *J* = 7.0, 3.5 Hz 1H), 7.22-7.17 (m, 2H), 6.93-6.86 (m, 1H), 6.36 (s, 1H), 3.97-3.75 (m, 1H), 3.63-3.45 (m, 2H), 2.92-2.74 (m, 2H), 2.35 (s, 3H), 2.26 (d, *J* = 13.5 Hz, 1H), 1.20 (s, 3H), 0.93 (s, 3H).
^{13}C NMR (126 MHz, $CDCl_3$) δ 154.0, 146.5, 139.1, 139.1, 129.0, 128.5, 127.1, 126.3, 124.3, 121.0, 117.3, 121.2-106.3 (m), 68.8, 61.4, 53.5 (d, *J* = 5.6 Hz), 36.7, 36.6 (t, *J* = 18.2 Hz), 28.7, 28.6, 21.8.
^{19}F NMR (376 MHz, $CDCl_3$) δ -81.0 (t, *J* = 9.8 Hz, 3F), -107.5 (AB, d, J_{F-F} = 267.7 Hz, 1F), -116.3 (AB, d, J_{F-F} = 270.0 Hz, 1F), -124.4 (s, 2F), -125.5 ~ -125.7 (m, 2F).
HRMS (ESI) m/z calcd. for $C_{25}H_{26}F_9N_2O$ $[M+H]^+$ 541.1901, found 541.1896.

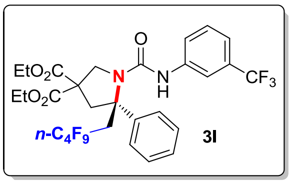

3I

(*R*)-diethyl 5-(2,2,3,3,4,4,5,5,5-nonafluoropentyl)-5-phenyl-1-((3-(trifluoromethyl)phenyl)carbamoyl)-pyrrolidine-3,3-dicarboxylate (3I)
HPLC analysis: Chiralcel OD3 (hexane/*i*-PrOH = 90/10, flow rate 0.3 mL/min, λ = 242 nm), t_R (major) = 15.15 min, t_R (minor) = 19.16 min.

^{1}H NMR (500 MHz, $CDCl_3$) δ 7.67 (s, 1H), 7.63 (d, J = 8.0 Hz, 1H), 7.41 (t, J = 8.0 Hz, 1H), 7.37-7.30 (m, 3H), 7.28-7.22 (m, 3H), 6.70 (br s, 1H), 4.70 (d, J = 9.0 Hz, 1H), 4.31-4.18 (m, 2H), 4.02-3.86 (m, 2H), 3.85-3.76 (m, 1H), 3.55-3.43 (m, 2H), 3.08 (d, J = 14.0 Hz, 1H), 2.92 (ddd, J = 31.0, 16.0, 7.5 Hz, 1H), 1.28 (t, J = 7.0 Hz, 3H), 0.88 (t, J = 7.0 Hz, 3H).
^{13}C NMR (126 MHz, $CDCl_3$) δ 168.7, 168.5, 152.6, 143.0, 139.1, 131.2 (q, J = 32.4 Hz), 129.4, 128.4, 127.5, 125.6, 124.0 (q, J = 272.2 Hz), 123.3, 119.9 (q, J = 3.8 Hz), 116.8 (q, J = 3.9 Hz), 121.1-106.5 (m), 67.6, 62.8, 62.4, 57.1, 52.4, 45.3 (d, J = 6.2 Hz), 35.3 (t, J = 18.4 Hz), 13.8, 13.4.
^{19}F NMR (376 MHz, $CDCl_3$) δ -62.8 (s, 3F), -81.0 ~ -81.1 (m, 3F), -107.4 (AB, d, $J_{F\text{-}F}$ = 268.1 Hz, 1F), -117.0 (AB, d, $J_{F\text{-}F}$ = 269.8 Hz, 1F). -124.4 (s, 2F), -125.5 ~ -125.8 (m, 2F).
HRMS (ESI) m/z calcd. for $C_{29}H_{27}F_{12}N_2O_5$ $[M+H]^+$ 711.1728, found 711.1723.

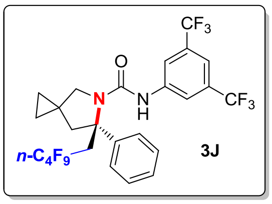

(*R*)-*N*-(3,5-bis(trifluoromethyl)phenyl)-6-(2,2,3,3,4,4,5,5,5-nonafluoropentyl)-6-phenyl-5-azaspiro[2.4]heptane-5-carboxamide (3J)

HPLC analysis: Chiralcel AD3 (hexane/*i*-PrOH = 97/3, flow rate 0.15 mL/min, λ = 254 nm), t_R (major) = 29.85 min, t_R (minor) = 34.01 min.
^{1}H NMR (500 MHz, $CDCl_3$) δ 7.69 (s, 2H), 7.50-7.26 (m, 6H), 6.83 (br s, 1H), 3.96-3.70 (m, 2H), 3.34 (d, J = 8.5 Hz, 1H), 3.16-2.95 (m, 2H), 1.80 (d, J = 13.0 Hz, 1H), 0.64-0.54 (m, 2H), 0.55-0.49 (m, 1H), 0.24-0.16 (m, 1H).
^{13}C NMR (126 MHz, $CDCl_3$) δ 152.5, 145.1, 140.2, 131.7 (q, J = 33.3 Hz), 128.6, 127.3, 124.6, 123.1 (q, J = 272.6 Hz), 119.5 (d, J = 3.0 Hz), 116.1 (q, J = 3.8 Hz), 122.0-106.5 (m), 67.9, 55.9, 48.3, 34.6 (t, J = 18.7 Hz), 18.2, 15.6, 5.8.
^{19}F NMR (376 MHz, $CDCl_3$) δ -63.3 (s, 6F), -79.0 ~ -86.1 (m, 3F), -108.8 (AB, d, $J_{F\text{-}F}$ = 246.5 Hz, 1F), -115.7 (AB, d, $J_{F\text{-}F}$ = 241.3 Hz, 1F). -124.5 (s, 2F), -125.7 ~ -125.8 (m, 2F).
HRMS (ESI) m/z calcd. for $C_{26}H_{20}F_{15}N_2O$ $[M+H]^+$ 661.1336, found 661.1331.

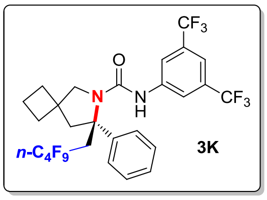

(*R*)-*N*-(3,5-bis(trifluoromethyl)phenyl)-7-(2,2,3,3,4,4,5,5,5-nonafluoropentyl)-7-phenyl-6-azaspiro[3.4]octane-6-carboxamide (3K)

HPLC analysis: Chiralcel AD3 (hexane/*i*-PrOH = 97/3, flow rate 0.3 mL/min, λ = 254 nm), t_R (major) = 13.42 min, t_R (minor) = 17.47 min.
^{1}H NMR (500 MHz, $CDCl_3$) δ 7.85 (s, 2H), 7.48 (s, 1H), 7.40-7.32 (m, 2H), 7.31-7.24 (m, 3H), 6.79 (br s, 1H), 4.02-3.78 (m, 2H), 3.64 (d, J = 8.5 Hz, 1H), 2.91-2.81 (m, 1H), 2.76 (d, J = 13.0 Hz, 1H), 2.50 (d, J = 13.0 Hz, 1H), 2.09-2.00 (m, 2H), 1.85-1.70 (m, 3H), 1.29 (s, 1H).
^{13}C NMR (126 MHz, $CDCl_3$) δ 153.1, 144.6, 140.6, 132.3 (q, J = 33.2 Hz), 128.7, 127.6, 125.7, 123.5 (q, J = 272.8 Hz), 119.8 (d, J = 3.9 Hz), 116.7-116.4 (m), 121.5-106.8 (m), 68.5, 60.3, 51.8, 42.7, 36.0, 35.5 (t, J = 18.4 Hz), 30.2, 16.6.

^{19}**F NMR** (376 MHz, $CDCl_3$) δ -63.2 (s, 6F), -81.0 ~ -81.5 (t, *J* = 9.8 Hz, 3F), -106.8 (AB, d, $J_{F\text{-}F}$ = 270.0 Hz, 1F), -117.7 (AB, d, $J_{F\text{-}F}$ = 269.6 Hz, 1F), -124.5 (d, *J* = 10.4 Hz, 2F), -125.5 ~ -126.0 (m, 2F).

HRMS (ESI) m/z calcd. for $C_{27}H_{22}F_{15}N_2O$ $[M+H]^+$ 675.1493, found 675.1487.

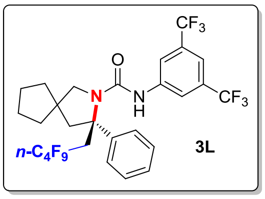

(*R*)-*N*-(3,5-bis(trifluoromethyl)phenyl)-3-(2,2,3,3,4,4,5,5,5-nonafluoropentyl)-3-phenyl-2-azaspiro[4.4]nonane-2-carboxamide (3L)

HPLC analysis: Chiralcel AD3 (hexane/*i*-PrOH = 96/4, flow rate 0.2 mL/min, λ = 254 nm), t_R (major) = 17.33 min, t_R (minor) = 20.87 min.

^{1}**H NMR** (500 MHz, $CDCl_3$) δ 7.84 (s, 2H), 7.46 (s, 1H), 7.37-7.31 (m, 4H), 7.26 (t, *J* = 7.0 Hz, 1H), 6.79 (br s, 1H), 3.96-3.76 (m, 1H), 3.66-3.52 (m, 2H), 2.99-2.68 (m, 2H), 2.30 (d, *J* = 13.0 Hz, 1H), 1.62 (s, 2H), 1.53 (s, 4H), 1.44-1.37 (m, 1H), 0.97-0.95 (m, 1H).

^{13}**C NMR** (126 MHz, $CDCl_3$) δ 153.3, 145.2, 140.7, 132.1 (q, *J* = 33.2 Hz), 128.6, 127.5, 126.1, 123.5 (q, *J* = 272.6 Hz), 120.0, 116.7-116.5 (m), 121.5-106.5 (m), 68.6, 60.4, 51.6, 47.4, 39.2, 38.6, 36.2 (t, *J* = 18.3 Hz), 25.3, 24.4.

^{19}**F NMR** (376 MHz, $CDCl_3$) δ -63.1 (s, 6F), -81.13 (t, *J* = 9.9 Hz, 3F), -106.6 (AB, d, $J_{F\text{-}F}$ = 270.9 Hz, 1F), -117.6 (AB, d, $J_{F\text{-}F}$ = 269.6 Hz, 1F), -124.52 (s, 2F), -125.6 ~ -125.8 (m, 2F).

HRMS (ESI) m/z calcd. for $C_{28}H_{24}F_{15}N_2O$ $[M+H]^+$ 689.1649, found 689.1644.

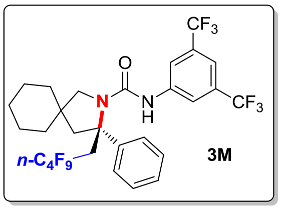

(*R*)-*N*-(3,5-bis(trifluoromethyl)phenyl)-3-(2,2,3,3,4,4,5,5,5-nonafluoropentyl)-3-phenyl-2-azaspiro[4.5]decane-2-carboxamide (3M)

HPLC analysis: Chiralcel AD3 (hexane/*i*-PrOH = 97/3, flow rate 0.2 mL/min, λ = 230 nm), t_R (major) = 16.43 min, t_R (minor) = 24.66 min.

^{1}**H NMR** (500 MHz, $CDCl_3$) δ 7.88 (s, 2H), 7.50 (s, 1H), 7.40-7.30 (m, 4H), 7.27 (t, *J* = 7.5 Hz, 1H), 6.81 (br s, 1H), 3.81 (dd, *J* = 35.0, 14.0 Hz, 1H), 3.70 (d, *J* = 8.5 Hz, 1H), 3.49 (d, *J* = 8.5 Hz, 1H), 2.94-2.77 (m, 1H), 2.66 (d, *J* = 13.5 Hz, 1H), 2.44 (d, *J* = 13.5 Hz, 1H), 1.53-1.20 (m, 10H).

^{13}**C NMR** (126 MHz, $CDCl_3$) δ 153.5, 146.0, 140.6, 132.34 (q, *J* = 33.3 Hz), 128.8, 127.4, 125.8, 123.5 (q, *J* = 273.2 Hz), 120.0, 116.8-116.5 (m), 121.5-106.8 (m), 68.5, 59.4, 51.2, 40.7, 38.7, 36.6 (t, *J* = 18.2 Hz), 36.2, 25.8, 24.2, 22.9.

^{19}**F NMR** (376 MHz, $CDCl_3$) δ -63.1 (s, 6F), -81.2 (t, *J* = 9.7 Hz, 3F), -107.5 (AB, d, $J_{F\text{-}F}$ = 268.9 Hz, 1F), -116.8 (AB, d, $J_{F\text{-}F}$ = 270.4 Hz, 1F), -124.5 (s, 2F), -125.77 (d, *J* = 11.4 Hz, 2F).

HRMS (ESI) m/z calcd. for $C_{30}H_{28}F_{15}N_2O$ $[M+H]^+$ 703.1806, found 703.1800.

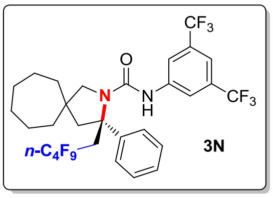

(*R*)-*N*-(3,5-bis(trifluoromethyl)phenyl)-3-(2,2,3,3,4,4,5,5,5-nonafluoropentyl)-3-phenyl-2-azaspiro[4.6]undecane-2-carboxamide (3N)

HPLC analysis: Chiralcel AD3 (hexane/*i*-PrOH = 95/5, flow rate 0.2 mL/min, λ = 254 nm), t_R (major) = 16.45 min, t_R (minor) = 19.99 min.

^{1}H NMR (500 MHz, $CDCl_3$) δ 7.88 (s, 2H), 7.49 (s, 1H), 7.41-7.33 (m, 4H), 7.31-7.24 (m, 1H), 6.84 (br s, 1H), 3.84 (dd, *J* = 32.0, 11.0 Hz, 1H), 3.60 (d, *J* = 8.5 Hz, 1H), 3.47 (d, *J* = 8.5 Hz, 1H), 2.94-2.67 (m, 2H), 2.45 (d, *J* = 13.5 Hz, 1H), 1.73-1.54 (m, 5H), 1.45-1.10 (m, 7H).

^{13}C NMR (126 MHz, $CDCl_3$) δ 153.2, 145.4, 140.3, 132.0 (q, *J* = 33.2 Hz), 128.3, 127.0, 125.8, 124.3 (q, *J* = 272.6 Hz), 119.6 (q, *J* = 2.8 Hz), 116.3-116.2 (m), 121.2-106.8 (m), 68.7, 61.1, 52.1, 43.7, 41.1, 39.0, 36.2 (t, *J* = 18.2 Hz), 29.2, 28.6, 24.6, 22.8.

^{19}F NMR (376 MHz, $CDCl_3$) δ -63.1 (s, 6F), -81.1 (t, *J* = 9.9 Hz, 3F), -107.0 (AB, d, $J_{F\text{-}F}$ = 269.7 Hz, 1F), -117.2 (AB, d, $J_{F\text{-}F}$ = 269.5 Hz, 1F), -124.5 (d, *J* = 4.9 Hz, 2F), -125.5 ~ -126.0 (m, 2F).

HRMS (ESI) m/z calcd. for $C_{30}H_{28}F_{15}N_2O$ $[M+H]^+$ 717.1962, found 717.1970.

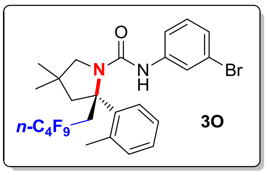

(*R*)-*N*-(3-bromophenyl)-4,4-dimethyl-2-(2,2,3,3,4,4,5,5,5-nonafluoropentyl)-2-(o-tolyl)pyrrolidine-1-carboxamide (3O)

HPLC analysis: Chiralcel AD3 (hexane/*i*-PrOH = 96/4, flow rate 0.2 mL/min, λ = 254 nm), t_R (major) = 28.24 min, t_R (minor) = 39.69 min.

^{1}H NMR (500 MHz, $CDCl_3$) δ 7.69 (s, 1H), 7.46-7.45 (m, 1H), 7.34 (d, *J* = 8.0 Hz, 1H), 7.24-7.10 (m, 5H), 6.45 (s, 1H), 4.19-3.94 (m, 1H), 3.62-3.38 (m, 2H), 2.96 (ddd, *J* = 31.5, 15.5, 8.0 Hz, 1H), 2.84 (d, *J* = 13.0 Hz, 1H), 2.48 (s, 3H), 2.32 (d, *J* = 13.0 Hz, 1H), 1.21 (s, 3H), 0.90 (s, 3H).

^{13}C NMR (126 MHz, $CDCl_3$) δ 154.0, 143.2, 140.8, 133.6, 133.3, 130.7, 128.0, 128.0, 126.7, 126.1, 123.4, 123.1, 119.0, 122.1-107.1 (m), 70.1, 61.4, 51.6, 37.3, 33.5 (t, *J* = 17.8 Hz), 29.4, 29.1, 23.1.

^{19}F NMR (376 MHz, $CDCl_3$) δ -80.9 ~ -81.0 (m, 3F), -107.1 (AB, d, $J_{F\text{-}F}$ = 268.8 Hz, 1F), -116.8 (AB, d, $J_{F\text{-}F}$ = 268.5 Hz, 1F). -124.6 (d, *J* = 8.3 Hz, 2F), -125.4 ~ -125.7 (m, 2F).

HRMS (ESI) m/z calcd. for $C_{25}H_{25}BrF_9N_2O$ $[M+H]^+$ 619.1007, found 619.1017.

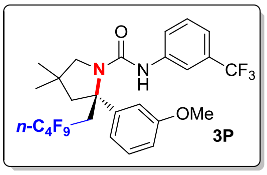

(*R*)-2-(3-methoxyphenyl)-4,4-dimethyl-2-(2,2,3,3,4,4,5,5,5-nonafluoropentyl)-*N*-(3-(trifluoromethyl)phenyl)-pyrrolidine-1-carboxamide (3P)

HPLC analysis: Chiralcel OD3 (hexane/*i*-PrOH = 95/5, flow rate 0.4 mL/min, λ = 230 nm), t_R (major) = 13.14 min, t_R (minor) = 16.75 min.

^{1}H NMR (500 MHz, $CDCl_3$) δ 7.64-7.62 (m, 2H), 7.38 (t, *J* = 8.0 Hz, 1H), 7.32-7.24 (m, 2H), 6.99-6.94 (m, 1H), 6.93 (t, *J* = 2.0 Hz, 1H), 6.80 (dd, *J* = 8.0, 2.0 Hz, 1H),

6.54 (s, 1H), 3.90-3.78 (m, 4H), 3.59-3.48 (m, 2H), 2.89-2.73 (m, 2H), 2.26 (d, *J* = 13.5 Hz, 1H), 1.19 (s, 3H), 0.96 (s, 3H).
^{13}C NMR (126 MHz, $CDCl_3$) δ 159.4, 153.3, 147.6, 139.3, 131.1 (q, *J* = 32.3 Hz), 129.3, 124.0 (d, *J* = 272.8 Hz), 123.0, 119.6 (q, *J* = 3.8 Hz), 118.3, 116.5 (q, *J* = 4.0 Hz), 113.1, 111.1, 120.7-106.5 (m), 68.5, 61.2, 55.2, 53.1, 36.4, 36.2 (t, *J* = 18.2 Hz), 29.0-28.4 (m).
^{19}F NMR (376 MHz, $CDCl_3$) δ -62.8 (s, 3F), -81.1 (t, *J* = 9.8 Hz, 3F), -107.5 (AB, d, $J_{F\text{-}F}$ = 287.9 Hz, 1F), -116.7 (AB, d, $J_{F\text{-}F}$ = 273.3 Hz, 1F), -124.5 (s, 2F), -125.5 ~ -125.8 (m, 2F).
HRMS (ESI) m/z calcd. for $C_{26}H_{25}F_{12}N_2O_2$ $[M+H]^+$ 625.1724, found 625.1719.

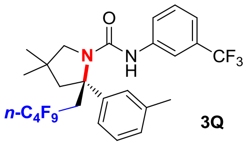

3Q

(*R*)-4,4-dimethyl-2-(2,2,3,3,4,4,5,5,5-nonafluoropentyl)-2-(m-tolyl)-*N*-(3-(trifluoromethyl)phenyl)pyrrolidine-1-carboxamide (3Q)

HPLC analysis: Chiralcel OD3 (hexane/*i*-PrOH = 95/5, flow rate 0.2 mL/min, λ = 242 nm), t_R (major) = 20.11 min, t_R (minor) = 22.04 min.
^{1}H NMR (500 MHz, $CDCl_3$) δ 7.60 (s, 2H), 7.35 (t, *J* = 8.5 Hz, 1H), 7.28-7.18 (m, 2H), 7.17-7.09 (m, 2H), 7.04 (d, *J* = 7.5 Hz, 1H), 6.51 (s, 1H), 3.79 (dd, *J* = 35.0, 14.5 Hz, 1H), 3.59-3.43 (m, 2H), 2.87-2.68 (m, 2H), 2.35 (s, 3H), 2.22 (d, *J* = 13.5 Hz, 1H), 1.15 (s, 3H), 0.90 (s, 3H).
^{13}C NMR (126 MHz, $CDCl_3$) δ 153.4, 146.2, 139.8, 138.2, 131.4 (q, *J* = 32.2 Hz), 129.7, 128.5, 128.1, 126.4, 124.0 (q, *J* = 272.8 Hz), 123.1, 123.0, 119.6 (q, *J* = 3.8 Hz), 116.6 (q, *J* = 3.9 Hz), 121.2-106.6 (m), 68.9, 61.5, 53.6, 36.8, 36.6 (t, *J* = 18.2 Hz), 28.7, 28.7, 22.1.
^{19}F NMR (376 MHz, $CDCl_3$) δ -62.8 (s, 3F), -81.1 (t, *J* = 9.8 Hz, 3F), -107.4 (AB, d, $J_{F\text{-}F}$ = 266.9 Hz, 1F), -116.8 (AB, d, $J_{F\text{-}F}$ = 272.2 Hz, 1F), -124.4 (s, 2F), -125.4 ~ -126.0 (m, 2F).
HRMS (ESI) m/z calcd. for $C_{26}H_{25}F_{12}N_2O$ $[M+H]^+$ 609.1775, found 609.1770.

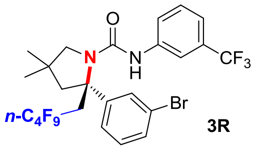

3R

(*R*)-2-(3-bromophenyl)-4,4-dimethyl-2-(2,2,3,3,4,4,5,5,5-nonafluoropentyl)-*N*-(3-(trifluoromethyl)phenyl)-pyrrolidine-1-carboxamide (3R)

HPLC analysis: Chiralcel OD3 (hexane/*i*-PrOH = 95/5, flow rate 0.2 mL/min, λ = 230 nm), t_R (major) = 23.79 min, t_R (minor) = 27.71 min.
^{1}H NMR (500 MHz, $CDCl_3$) δ 7.68-7.62 (m, 2H), 7.48 (t, *J* = 2.0 Hz, 1H), 7.43-7.38 (m, 2H), 7.31 (d, *J* = 8.0 Hz, 2H), 7.23 (t, *J* = 8.0 Hz, 1H), 6.54 (s, 1H), 3.86 (ddd, *J* = 36.0, 16.0, 4.0 Hz, 1H), 3.59-3.46 (m, 2H), 2.84-2.66 (m, 2H), 2.22 (d, *J* = 13.5 Hz, 1H), 1.20 (s, 3H), 0.93 (s, 3H).
^{13}C NMR (126 MHz, $CDCl_3$) δ 153.3, 148.4, 139.2, 131.2 (q, *J* = 32.3 Hz), 130.1, 129.7, 129.4, 129.1, 124.7, 124.0 (q, *J* = 272.3 Hz), 123.2, 122.5, 119.9 (q, *J* = 4.0 Hz), 116.7 (q, *J* = 3.9 Hz), 121.1-106.5 (m), 68.4, 61.0, 53.0 (d, *J* = 5.9 Hz), 36.7, 36.0 (t, *J* = 18.3 Hz), 28.3, 28.2.

^{19}F NMR (376 MHz, $CDCl_3$) δ -62.76 (s, 3F), -81.04 (t, *J* = 9.8 Hz, 3F), -107.3 (AB, d, $J_{F\text{-}F}$ = 267.6 Hz, 1F), -115.89 ~ -117.79 (m, 1F), -124.38 (s, 2F), -124.79 ~ -125.92 (m, 2F).
HRMS (ESI) m/z calcd. for $C_{25}H_{22}BrF_{12}N_2O$ $[M+H]^+$ 673.0724, found 673.0740.

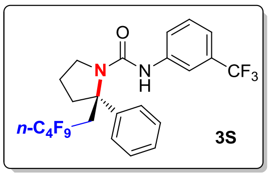

(*R*)-2-(2,2,3,3,4,4,5,5,5-nonafluoropentyl)-2-phenyl-*N*-(3-(trifluoromethyl)phenyl)pyrrolidine-1-carboxamide (3S)
HPLC analysis: Chiralcel AD3 (hexane/*i*-PrOH = 95/5, flow rate 0.5 mL/min, λ = 230 nm), t_R (major) = 15.80 min, t_R (minor) = 34.41 min.
^{1}H NMR (500 MHz, Acetone-d_6) δ 8.16 (s, 1H), 8.01 (s, 1H), 7.82 (d, *J* = 8.5 Hz, 1H), 7.48-7.40 (m, 3H), 7.36 (t, *J* = 8.0 Hz, 2H), 7.30-7.22 (m, 2H), 4.12-3.94 (m, 2H), 3.87-3.76 (m, 1H), 3.24 (ddd, *J* = 32.0, 15.5, 8.0 Hz, 1H), 2.77-2.66 (m, 1H), 2.20 (ddd, *J* = 13.0, 6.5, 3.0 Hz, 1H), 2.05-1.94 (m, 1H), 1.88-1.71 (m, 1H).
^{13}C NMR (126 MHz, Acetone-d_6) δ 153.4, 146.0, 141.4, 130.1 (q, *J* = 31.6 Hz), 129.2, 128.2, 126.6, 125.2, 124.5 (q, *J* = 272.0 Hz), 122.9, 118.2 (q, *J* = 3.9 Hz), 115.9 (q, *J* = 4.1 Hz), 122.8-106.7 (m), 67.4, 47.8, 39.9 (d, *J* = 5.2 Hz), 34.1 (t, *J* = 18.0 Hz), 21.4.
^{19}F NMR (376 MHz, $CDCl_3$) δ -62.8 (s, 3F), -81.1 (t, *J* = 9.7 Hz, 3F), -108.6 (AB, d, *J* = 259.8 Hz, 1F), -116.5 (AB, d, *J* = 272.1 Hz, 1F), -124.4 (s, 2F), -125.5 ~ -126.0 (m, 2F).
HRMS (ESI) m/z calcd. for $C_{23}H_{19}F_{12}N_2O$ $[M+H]^+$ 567.1306, found 567.1300.

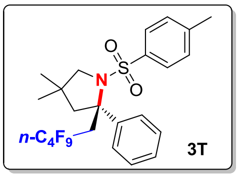

(*R*)-4,4-dimethyl-2-(2,2,3,3,4,4,5,5,5-nonafluoropentyl)-2-phenyl-1-tosylpyrrolidine (3T)
HPLC analysis: Chiralcel ID (hexane/*i*-PrOH = 98/2, flow rate 0.4 mL/min, λ = 230 nm), t_R (major) = 21.03 min, t_R (minor) = 24.07 min.
^{1}H NMR (500 MHz, $CDCl_3$) δ 7.34-7.28 (m, 2H), 7.27-7.15 (m, 3H), 7.04 (s, 4H), 4.23 (ddd, *J* = 33.0, 16.5, 4.5 Hz, 1H), 3.50 (d, *J* = 9.0 Hz, 1H), 3.15 (d, *J* = 9.0 Hz, 1H), 3.11-2.92 (m, 1H), 2.59 (d, *J* = 14.5 Hz, 1H), 2.50 (d, *J* = 14.5 Hz, 1H), 2.36 (s, 3H), 1.30 (s, 3H), 1.16 (s, 3H).
^{13}C NMR (126 MHz, $CDCl_3$) δ 142.6, 141.9, 136.3, 129.0, 127.9, 127.5, 127.0, 126.8, 120.8-106.6 (m), 69.7, 61.8, 53.7 (d, *J* = 4.3 Hz), 39.8 (t, *J* = 19.8 Hz), 36.8, 28.7, 27.3, 21.4.
^{19}F NMR (376 MHz, $CDCl_3$) δ -81.0 (t, *J* = 2.9 Hz, 3F), -104.0 (AB, dd, *J* = 274.5, 15.7 Hz, 1F), -108.0 (AB, d, *J* = 273.4 Hz, 1F), -124.1 (d, *J* = 8.6 Hz, 2F), -125.5 (dd, *J* = 17.6, 9.3 Hz, 1F), -125.7 (t, *J* = 14.2 Hz, 1F).
HRMS (ESI) m/z calcd. for $C_{24}H_{25}F_9NO_2S$ $[M+H]^+$ 562.1462, found 562.1471.

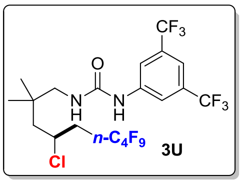

1-(3,5-Bis(trifluoromethyl)phenyl)-3-(4-chloro-6,6,7,7,8,8,9,9,9-nonafluoro-2,2-dimethylnonyl)urea (3U)
^{1}H NMR (500 MHz, $CDCl_3$) δ 7.85 (s, 1H), 7.75 (s, 2H), 7.43 (s, 1H), 5.66 (t, *J* = 6.0 Hz, 1H), 4.33-4.28 (m, 1H), 3.32 (dd, *J* =

14.0, 7.5 Hz, 1H), 3.13 (dd, J = 14.0, 5.5 Hz, 1H), 2.67-2.39 (m, 2H), 1.90 (dd, J = 15.5, 9.5 Hz, 1H), 1.75-1.73 (m, 1H), 0.99 (s, 3H), 0.97 (s, 3H).
^{13}C NMR (125 MHz, $CDCl_3$) δ 155.7, 140.3, 132.3 (q, J = 33.4 Hz), 123.03 (q, J = 272.7 Hz), 118.6, 116.0, 119.8-108.2 (m), 49.6, 49.3, 47.8, 40.8 (t, J = 20.4 Hz), 35.0, 25.9, 24.7.
^{19}F NMR (376 MHz, $CDCl_3$) δ -63.5 (s, 6F), -81.3 (t, J = 9.4 Hz, 3F), -113.5 (s, 2F), -124.9 (s, 2F), -126.2 (s, 2F).
HRMS (ESI) m/z calcd. for $C_{20}H_{19}ClF_{15}N_2O$ [M+H]$^+$ 623.0941, found 623.0933.

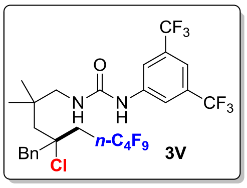

1-(4-Benzyl-4-chloro-6,6,7,7,8,8,9,9,9-nonafluoro-2,2-dimethylnonyl)-3-(3,5-bis(trifluoromethyl)phenyl)urea (3V)
^{1}H NMR (500 MHz, $CDCl_3$) δ 7.76 (s, 2H), 7.45 (s, 1H), 7.31 (s, 5H), 7.23 (s, 1H), 5.21 (t, J = 6.0 Hz, 1H), 3.37-3.30 (m, 3H), 3.21 (d, J = 14.0 Hz, 1H), 2.72-2.60 (m, 2H), 2.06 (s, 2H), 1.12 (s, 3H), 1.09 (s, 3H).
^{13}C NMR (125 MHz, $CDCl_3$) δ 155.2, 140.5, 135.0, δ 132.2 (q, J = 33.3 Hz), 131.6, 128.1, 127.4, 123.1 (q, J = 272.7 Hz), 118.4, 115.8, 112.8, 118.4-106.2 (m), 71.6, 53.4, 50.4, 49.4, 47.7, 41.3 (t, J = 19.0 Hz), 36.7, 31.1, 27.5, 26.7.
^{19}F NMR (376 MHz, $CDCl_3$) δ -63.4 (s, 6F), -81.2 (t, J = 9.5 Hz, 3F), -107.6 (AB, d, $J_{F\text{-}F}$ = 271.5 Hz, 1F), -111.2 (AB, d, $J_{F\text{-}F}$ = 274.1 Hz, 1F), -124.5 (s, 2F), -125.8 ~ -126.0 (m, 2F).
HRMS (ESI) m/z calcd. for $C_{27}H_{25}ClF_{15}N_2O$ [M+H]$^+$ 713.1410, found 713.1414.

General procedure B: direct asymmetric intramolecular radical amino-difluoro(methoxycarbonyl)methylation of alkenes

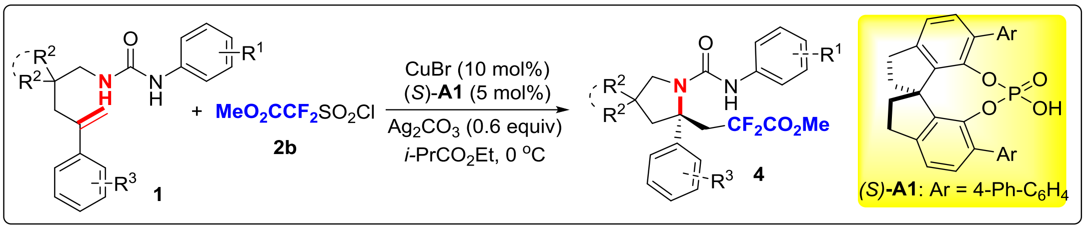

Under argon, an oven-dried resealable Schlenk tube equipped with a magnetic stir bar was charged with urea substrate **1** (0.1 mmol, 1.0 equiv), CuBr (1.43 mg, 0.01 mmol, 10 mol%), Ag_2CO_3 (16.56 mg, 0.06 mmol, 0.6 equiv) chiral phosphoric acid (*S*)-**A1** (3.1 mg, 0.005 mmol, 5 mol%), $MeO_2CCF_2SO_2Cl$ (**2b**) (25 mg, 0.12 mmol, 1.2 equiv) and ethyl isobutyrate (1.0 mL) at 0 °C, and the sealed tube was then stirred at 0 °C. Upon completion (monitored by TLC), the reaction mixture was directly purified by a silica gel chromatography [eluent: petroleum ether/EtOAc = 100/0-5/1, using petroleum ether (100%) to remove the solvent (ethyl isobutyrate) at first] to afford the desired product **4**.

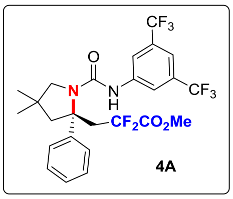

(*R*)-methyl 3-(1-((3,5-bis(trifluoromethyl)phenyl)-carbamoyl)-4,4-dimethyl-2-phenylpyrrolidin-2-yl)-2,2-difluoropropanoate (4A)

HPLC analysis: Chiralcel AD3 (hexane/*i*-PrOH = 95/5, flow rate 0.2 mL/min, λ = 254 nm), t_R (major) = 20.82 min, t_R (minor) = 19.01 min.

^{1}H NMR (500 MHz, $CDCl_3$) δ 7.91 (s, 2H), 7.49 (s, 1H), 7.39-7.30 (m, 4H), 7.27-7.22 (m, 1H), 6.82 (br s, 1H), 3.74-3.57 (m, 5H), 3.53 (d, *J* = 8.5 Hz, 1H), 2.95 (q, *J* = 16.0 Hz, 1H), 2.83 (d, *J* = 13.5 Hz, 1H), 2.22 (d, *J* = 13.5 Hz, 1H), 1.17 (s, 3H), 0.89 (s, 3H).

^{13}C NMR (126 MHz, $CDCl_3$) δ 164.7 (t, *J* = 33.0 Hz), 153.4, 145.7, 140.5, 131.9 (q, *J* = 33.4 Hz), 128.3, 126.9, 125.6, 123.2 (q, *J* = 272.5 Hz), 119.5, 116.1, 115.8 (t, *J* = 253.0 Hz), 68.8, 61.3, 53.8, 53.4, 41.7 (t, *J* = 20.6 Hz), 36.4, 28.4, 28.3.

^{19}F NMR (376 MHz, $CDCl_3$) δ -63.0 (s, 6F), -95.8 (d, *J* = 262.1 Hz, 1F), -108.4 (d, *J* = 260.8 Hz, 1F).

HRMS (ESI) m/z calcd. for $C_{25}H_{25}F_8N_2O_3$ $[M+H]^+$ 553.1737, found 553.1732.

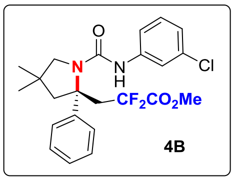

(*R*)-methyl 3-(1-((3-chlorophenyl)carbamoyl)-4,4-dimethyl-2-phenylpyrrolidin-2-yl)-2,2-difluoropropanoate (4B)

HPLC analysis: Chiralcel OD3 (hexane/*i*-PrOH = 90/10, flow rate 0.45 mL/min, λ = 254 nm), t_R (major) = 13.13 min, t_R (minor) = 16.31 min.

^{1}H NMR (500 MHz, $CDCl_3$) δ 7.59 (s, 1H), 7.39-7.30 (m, 4H), 7.28-7.15 (m, 3H), 7.06-6.98 (m, 1H), 6.37 (s, 1H), 3.77-3.56 (m, 5H), 3.49 (d, *J* = 8.0 Hz, 1H), 2.94 (q, *J* = 16.0 Hz, 1H), 2.82 (d, *J* = 13.5 Hz, 1H), 2.20 (d, *J* = 13.5 Hz, 1H), 1.19 (s, 3H), 0.90 (s, 3H).

^{13}C NMR (126 MHz, $CDCl_3$) δ 164.69 (dd, *J* = 33.6, 32.4 Hz), 153.4, 146.1, 140.2, 134.5, 129.7, 128.2, 126.7, 125.8, 123.0, 119.9, 117.8, 115.8 (dd, *J* = 254.4, 250.7 Hz), 68.5 (d, *J* = 4.0 Hz), 61.3, 53.8, 53.5, 42.0 (dd, *J* = 22.0, 19.7 Hz), 36.3, 28.6, 28.5.
^{19}F NMR (376 MHz, $CDCl_3$) δ -95.6 (d, *J* = 261.2 Hz, 1F), -106.7 (d, *J* = 260.9 Hz, 1F).
HRMS (ESI) m/z calcd. for $C_{23}H_{26}ClF_2N_2O_3$ $[M+H]^+$ 451.1600, found 451.1595..

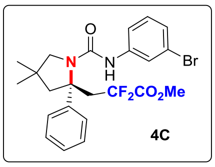

(*R*)-methyl 3-(1-((3-bromophenyl)carbamoyl)-4,4-dimethyl-2-phenylpyrrolidin-2-yl)-2,2-difluoropropanoate (4C)
HPLC analysis: Chiralcel AD3 (hexane/*i*-PrOH = 90/10, flow rate 0.5 mL/min, λ = 254 nm), t_R (major) = 21.38 min, t_R (minor) = 20.34 min.
^{1}H NMR (500 MHz, $CDCl_3$) δ 7.74 (s, 1H), 7.38-7.21 (m, 6H), 7.20-7.11 (m, 2H), 6.36 (s, 1H), 3.72 (s, 3H), 3.65 (q, *J* = 17.0 Hz, 1H), 3.58 (d, *J* = 8.0 Hz, 1H), 3.48 (d, J = 8.5 Hz, 1H), 2.95 (q, *J* = 16.0 Hz, 1H), 2.82 (d, *J* = 13.5 Hz, 1H), 2.20 (d, *J* = 13.5 Hz, 1H), 1.19 (s, 3H), 0.90 (s, 3H).
^{13}C NMR (126 MHz, $CDCl_3$) δ 164.7 (dd, *J* = 33.9, 32.1 Hz), 153.4, 146.1, 140.3, 130.0, 128.2, 126.7, 126.0, 125.8, 122.7, 122.5, 118.3, 115.8 (dd, *J* = 253.9, 250.5 Hz), 68.5 (d, *J* = 4.0 Hz), 61.3, 53.8, 53.5, 42.0 (dd, *J* = 22.0, 19.8 Hz), 36.3, 28.6, 28.5.
^{19}F NMR (376 MHz, $CDCl_3$) δ -95.6 (d, *J* = 263.2 Hz, 1F), -106.8 (d, *J* = 260.2 Hz, 1F).
HRMS (ESI) m/z calcd. for $C_{23}H_{26}BrF_2N_2O_3$ $[M+H]^+$ 495.1095, found 495.1089.

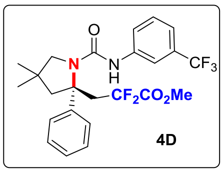

(*R*)-methyl 3-(4,4-dimethyl-2-phenyl-1-((3-(trifluoromethyl)phenyl)carbamoyl)pyrrolidin-2-yl)-2,2-difluoropropanoate (4D)
HPLC analysis: Chiralcel AD3 (hexane/*i*-PrOH = 95/5, flow rate 0.4 mL/min, λ = 230 nm), t_R (major) = 29.76 min, t_R (minor) = 37.29 min.
^{1}H NMR (500 MHz, $CDCl_3$) δ 7.74 (s, 1H), 7.60 (d, *J* = 8.0 Hz, 1H), 7.42-7.32 (m, 5H), 7.29 (d, *J* = 8.0 Hz, 1H), 7.24 (t, *J* = 7.0 Hz, 1H), 6.50 (s, 1H), 3.76-3.57 (m, 5H), 3.51 (d, *J* = 8.5 Hz, 1H), 2.95 (q, *J* = 16.0 Hz, 1H), 2.83 (d, *J* = 13.0 Hz, 1H), 2.21 (d, *J* = 13.0 Hz, 1H), 1.19 (s, 3H), 0.91 (s, 3H).
^{13}C NMR (126 MHz, $CDCl_3$) δ 164.7 (dd, *J* = 33.9, 32.1 Hz), 153.5, 146.0, 139.6, 131.1 (q, *J* = 32.2 Hz), 129.3, 128.2, 126.8, 125.7, 124.0 (q, *J* = 273.0 Hz), 123.0, 119.6 (q, *J* = 3.9 Hz), 116.5 (q, *J* = 3.9 Hz), 115.8 (dd, *J* = 253.9, 250.7 Hz), 68.6 (d, *J* = 3.9 Hz), 61.3, 53.8, 53.4, 42.0 (dd, *J* = 22.0, 19.7 Hz), 36.4, 28.5, 28.4.
^{19}F NMR (376 MHz, $CDCl_3$) δ -62.6 (s, 3F), -95.5 (d, *J* = 261.7 Hz, 1F), -107.2 (d, *J* = 261.4 Hz, 1F).
HRMS (ESI) m/z calcd. for $C_{24}H_{26}F_5N_2O_3$ $[M+H]^+$ 485.1864, found 485.1858.

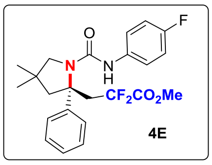

(*R*)-methyl 2,2-difluoro-3-(1-((4-fluorophenyl)carbamoyl)-4,4-dimethyl-2-phenylpyrrolidin-2-yl)propanoate (4E)

HPLC analysis: Chiralcel AD3 (hexane/*i*-PrOH = 90/10, flow rate 0.6 mL/min, λ = 242 nm), t_R (major) = 29.30 min, t_R (minor) = 20.67 min.
^{1}H NMR (500 MHz, $CDCl_3$) δ 7.41-7.30 (m, 6H), 7.26-7.20 (m, 1H), 6.98 (t, *J* = 8.5 Hz, 2H), 6.31 (s, 1H), 3.75-3.52 (m, 5H), 3.48 (d, *J* = 8.5 Hz, 1H), 2.94 (q, *J* = 16.0 Hz, 1H), 2.81 (d, *J* = 13.0 Hz, 1H), 2.19 (d, *J* = 13.5 Hz, 1H), 1.19 (s, 3H), 0.90 (s, 3H).
^{13}C NMR (126 MHz, $CDCl_3$) δ 164.7 (dd, *J* = 34.0, 31.9 Hz), 158.9 (d, *J* = 241.6 Hz), 154.0, 146.2, 134.8 (d, *J* = 2.7 Hz), 128.2, 126.6, 125.8, 122.1 (d, *J* = 7.7 Hz), 115.9 (dd, *J* = 253.7, 250.5 Hz), 115.3 (d, *J* = 22.4 Hz), 68.4 (d, *J* = 4.1 Hz), 61.2, 53.8, 53.5, 42.1 (dd, *J* = 22.0, 19.7 Hz), 36.3, 28.6, 28.5.
^{19}F NMR (376 MHz, $CDCl_3$) δ -95.9 (d, *J* = 261.6 Hz, 1F), -106.5 (dt, *J* = 261.6, 17.7 Hz, 1F), -119.99 ~ -120.12 (m, 1F).
HRMS (ESI) m/z calcd. for $C_{23}H_{26}F_3N_2O_3$ $[M+H]^+$ 435.1896, found 435.1890.

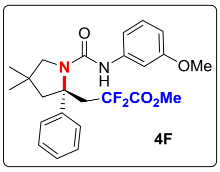

(*R*)-methyl 2,2-difluoro-3-(1-((3-methoxyphenyl)-carbamoyl)-4,4-dimethyl-2-phenylpyrrolidin-2-yl)propanoate (4F)
HPLC analysis: Chiralcel AD3 (hexane/*i*-PrOH = 90/10, flow rate 0.6 mL/min, λ = 254 nm), t_R (major) = 26.75 min, t_R (minor) = 18.79 min.
^{1}H NMR (500 MHz, $CDCl_3$) δ 7.35-7.33 (m, 4H), 7.25-7.16 (m, 3H), 6.92 (d, *J* = 8.0 Hz, 1H), 6.61 (dd, *J* = 8.5, 2.5 Hz, 1H), 6.34 (s, 1H), 3.80 (s, 3H), 3.75-3.62 (m, 4H), 3.57 (d, *J* = 8.5 Hz, 1H), 3.49 (d, *J* = 8.0 Hz, 1H), 2.94 (q, *J* = 15.5 Hz, 1H), 2.83 (d, *J* = 13.5 Hz, 1H), 2.19 (d, *J* = 13.5 Hz, 1H), 1.19 (s, 3H), 0.89 (s, 3H).
^{13}C NMR (126 MHz, $CDCl_3$) δ 164.7 (dd, *J* = 34.0, 32.0 Hz), 160.1, 153.6, 146.2, 140.3, 129.5, 128.2, 126.7, 125.9, 115.9 (dd, *J* = 253.8, 250.3 Hz), 112.1, 108.8, 105.6, 68.4 (d, *J* = 4.4 Hz), 61.3, 55.3, 53.7, 53.5, 42.1 (dd, *J* = 22.2, 19.7 Hz), 36.2, 28.6, 28.5.
^{19}F NMR (376 MHz, $CDCl_3$) δ -95.6 (d, *J* = 261.3 Hz, 1F), -106.0 (dt, *J* = 261.7, 17.9 Hz, 1F).
HRMS (ESI) m/z calcd. for $C_{24}H_{29}F_2N_2O_4$ $[M+H]^+$ 447.2095, found 447.2090.

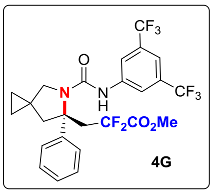

(*R*)-methyl 3-(5-((3,5-bis(trifluoromethyl)phenyl)-carbamoyl)-6-phenyl-5-azaspiro[2.4]heptan-6-yl)-2,2-difluoropropanoate (4G)
HPLC analysis: Chiralcel AD3 (hexane/*i*-PrOH = 97/3, flow rate 0.3 mL/min, λ = 230 nm), t_R (major) = 21.65 min, t_R (minor) = 18.27 min.
^{1}H NMR (500 MHz, Acetone-*d*$_6$) δ 8.34 (s, 1H), 8.31 (d, *J* = 1.5 Hz, 2H), 7.59 (s, 1H), 7.48-7.42 (m, 2H), 7.33 (t, *J* = 7.5 Hz, 2H), 7.24 (d, *J* = 7.5 Hz, 1H), 4.00 (d, *J* = 9.0 Hz, 1H), 3.87 (dt, *J* = 22.5, 15.5 Hz, 1H), 3.78 (s, 3H), 3.67 (d, *J* = 9.0 Hz, 1H), 3.25 (q, *J* = 14.0 Hz, 1H), 3.08 (d, *J* = 13.0 Hz, 1H), 1.72 (d, *J* = 13.0 Hz, 1H), 0.66-0.49 (m, 3H), 0.11-0.04 (m, 1H).
^{13}C NMR (126 MHz, Acetone-*d*$_6$) δ 164.4 (dd, *J* = 34.1, 31.6 Hz), 153.0, 146.1, 142.5, 131.2 (q, *J* = 32.9 Hz), 127.8, 126.4, 125.3, 123.7 (q, *J* = 272.4 Hz), 119.0 (d, *J* = 3.9

Hz), 117.0 (q, J = 258.9, 252.0 Hz), 114.5 (p, J = 3.9 Hz), 68.2 (d, J = 6.4 Hz), 55.8, 52.9, 47.7 (dd, J = 4.0, 1.8 Hz), 40.3 (dd, J = 23.4, 20.2 Hz), 18.0, 15.7, 4.6.
^{19}F NMR (376 MHz, Acetone-d_6) δ -63.5 (s, 6F), -95.80 (dt, J = 259.4, 14.9 Hz, 1F), -103.2 (ddd, J = 259.4, 22.9, 15.9 Hz, 1F).
HRMS (ESI) m/z calcd. for $C_{25}H_{23}F_8N_2O_3$ $[M+H]^+$ 551.1581, found 551.1575.

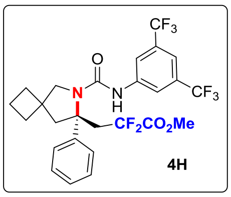

(*R*)-methyl 3-(6-((3,5-bis(trifluoromethyl)phenyl)-carbamoyl)-7-phenyl-6-azaspiro[3.4]octan-7-yl)-2,2-difluoropropanoate (4H)
HPLC analysis: Chiralcel OD3 (hexane/*i*-PrOH = 97/3, flow rate 0.2 mL/min, λ = 254 nm), t_R (major) = 25.59 min, t_R (minor) = 29.51 min.
^{1}H NMR (500 MHz, $CDCl_3$) δ 7.90 (s, 2H), 7.49 (s, 1H), 7.35 (t, J = 8.0 Hz, 2H), 7.29-7.20 (m, 3H), 6.78 (br s, 1H), 3.92 (d, J = 8.5 Hz, 1H), 3.82-3.61 (m, 5H), 3.01 (q, J = 16.0 Hz, 1H), 2.79 (d, J = 13.0 Hz, 1H), 2.44 (d, J = 13.0 Hz, 1H), 2.13-1.99 (m, 2H), 1.85-1.73 (m, 3H), 1.41-1.32 (m, 1H).
^{13}C NMR (126 MHz, $CDCl_3$) δ 165.0 (t, J = 32.9 Hz), 153.2, 144.9, 140.8, 132.2 (q, J = 33.3 Hz), 128.7, 127.4, 125.5, 123.5 (d, J = 272.8 Hz), 119.6 (d, J = 4.3 Hz), 116.3, 116.1 (t, J = 252.3 Hz), 68.4, 60.6, 53.8, 52.5, 42.6, 41.0 (t, J = 21.1 Hz), 36.2, 30.4, 16.6.
^{19}F NMR (376 MHz, $CDCl_3$) δ -63.0 (s, 6F), -95.4 (d, J = 269.3 Hz, 1F), -107.6 (d, J = 264.6 Hz, 1F).
HRMS (ESI) m/z calcd. for $C_{26}H_{25}F_8N_2O_3$ $[M+H]^+$ 565.1737, found 565.1732.

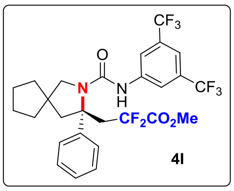

(*R*)-methyl 3-(2-((3,5-bis(trifluoromethyl)phenyl)-carbamoyl)-3-phenyl-2-azaspiro[4.4]nonan-3-yl)-2,2-difluoropropanoate (4I)
HPLC analysis: Chiralcel OD3 (hexane/*i*-PrOH = 97/3, flow rate 0.2 mL/min, λ = 254 nm), t_R (major) = 23.11 min, t_R (minor) = 25.99 min.
^{1}H NMR (500 MHz, $CDCl_3$) δ 7.92 (s, 2H), 7.50 (s, 1H), 7.39-7.24 (m, 5H), 6.74 (s, 1H), 3.80-3.53 (m, 6H), 3.07-2.90 (m, 2H), 2.27 (d, J = 13.0 Hz, 1H), 1.76-1.63 (m, 2H), 1.60-1.52 (m, 4H), 1.49-1.39 (m, 1H), 1.06-0.93 (m, 1H).
^{13}C NMR (126 MHz, $CDCl_3$) δ 164.7 (t, J = 32.9 Hz), 153.1, 145.1, 140.5, 132.0 (q, J = 33.5 Hz), 128.3, 127.0, 125.6, 123.2 (q, J = 273.1 Hz), 119.4, 116.1, 115.8 (t, J = 252.1 Hz), 68.4, 60.3, 53.5, 52.4, 47.0, 41.4 (t, J = 20.8 Hz), 39.1, 38.4, 25.0, 24.0.
^{19}F NMR (376 MHz, $CDCl_3$) δ -63.0 (s, 6F), -95.3 (d, J = 260.5 Hz, 1F), -107.9 (d, J = 263.1 Hz, 1F).
HRMS (ESI) m/z calcd. for $C_{27}H_{27}F_8N_2O_3$ $[M+H]^+$ 579.1894, found 579.1888.

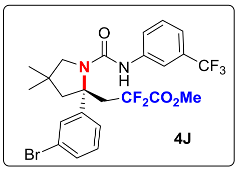

(*R*)-methyl 3-(2-(3-bromophenyl)-4,4-dimethyl-1-((3-(trifluoromethyl)phenyl)carbamoyl)pyrrolidin-2-yl)-2,2-difluoropropanoate (4J)
HPLC analysis: Chiralcel AD3 (hexane/*i*-PrOH = 95/5, flow rate

0.45 mL/min, λ = 240 nm), t_R (major) = 24.22 min, t_R (minor) = 22.07 min.
^{1}H NMR (500 MHz, Acetone-d_6) δ 8.06 (s, 2H), 7.87 (d, *J* = 8.0 Hz, 1H), 7.64 (t, *J* = 2.0 Hz, 1H), 7.49-7.44 (m, 2H), 7.41-7.38 (m, 1H), 7.29 (dd, *J* = 16.0, 8.0 Hz, 2H), 3.77- 3.57 (m, 5H), 3.57 (d, *J* = 9.0 Hz, 1H), 3.11-3.01 (m, 1H), 2.76 (d, *J* = 13.5 Hz, 1H), 2.20 (d, *J* = 13.5 Hz, 1H), 1.15 (s, 3H), 0.87 (s, 3H).
^{13}C NMR (126 MHz, Acetone-d_6) δ 164.5 (dd, *J* = 33.5, 32.1 Hz) 153.9, 149.7, 141.3, 130.1 (q, *J* = 31.8 Hz), 129.7, 129.4, 129.3, 129.3, 125.4, 124.5 (q, *J* = 271.9 Hz), 123.1, 121.5, 118.4 (q, *J* = 3.9 Hz), 116.1 (q, *J* = 4.1 Hz), 116.1 (t, *J* = 252.6 Hz), 68.3 (d, *J* = 4.2 Hz), 60.9, 53.1 (t, *J* = 3.3 Hz), 53.0, 41.3 (dd, *J* = 22.2, 19.5 Hz), 36.0, 27.7.
^{19}F NMR (376 MHz, Acetone-d_6) δ -63.1 (s, 3F), -97.2 (ddd, *J* = 259.9, 18.7, 14.4 Hz, 1F), -104.8 (dt, *J* = 259.9, 18.2 Hz, 1F).
HRMS (ESI) m/z calcd. for $C_{24}H_{25}BrF_5N_2O_3$ $[M+H]^+$ 563.0969, found 563.0976.

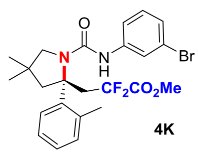

4K

(*R*)-methyl 3-(1-((3-bromophenyl)carbamoyl)-4,4-dimethyl -2-(o-tolyl)pyrrolidin-2-yl)-2,2-difluoropropanoate (4K)
HPLC analysis: Chiralcel AD3 (hexane/*i*-PrOH = 95/5, flow rate 0.6 mL/min, λ = 230 nm), t_R (major) = 25.70 min, t_R (minor) = 16.58 min.
^{1}H NMR (500 MHz, $CDCl_3$) δ 7.75 (s, 1H), 7.37 (t, *J* = 4.5 Hz, 1H), 7.34-7.25 (m, 1H), 7.24-7.10 (m, 5H), 6.38 (s, 1H), 3.85 (q, *J* = 17.5 Hz, 1H), 3.74 (s, 3H), 3.56 (d, *J* = 8.0 Hz, 1H), 3.47 (d, *J* = 8.0 Hz, 1H), 3.09 (q, *J* = 16.5 Hz, 1H), 2.82 (d, *J* = 13.0 Hz, 1H), 2.50 (s, 3H), 2.29 (d, *J* = 13.0 Hz, 1H), 1.21 (s, 3H), 0.88 (s, 3H).
^{13}C NMR (126 MHz, $CDCl_3$) δ 164.7 (t, *J* = 33.0 Hz), 153.5, 142.7, 140.3, 133.1, 133.0, 130.0, 127.2, 127.1, 126.0, 125.4, 122.8, 122.5, 118.3, 116.2 (dd, *J* = 251.2, 250.2 Hz), 69.5, 61.0, 53.5, 51.4, 39.3 (t, *J* = 21.7 Hz), 36.6, 29.0, 28.5, 22.8.
^{19}F NMR (376 MHz, $CDCl_3$) δ -95.0 (d, *J* = 262.5 Hz, 1F), -107.2 (d, *J* = 256.1 Hz, 1F).
HRMS (ESI) m/z calcd. for $C_{24}H_{28}BrF_2N_2O_3$ $[M+H]^+$ 509.1251, found 509.1258.

General procedure C: direct asymmetric intramolecular radical aminodifluoro -methylation of alkenes

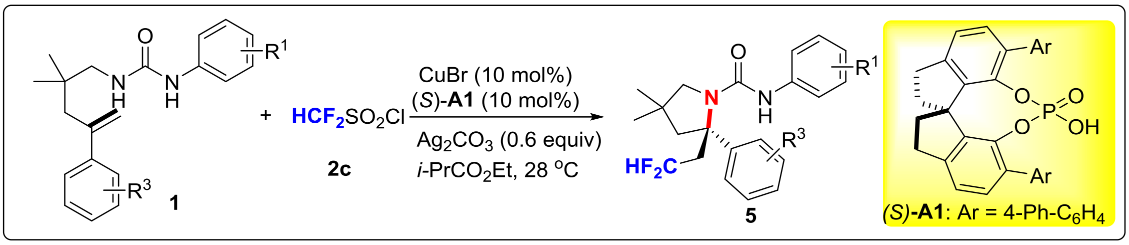

Under argon, an oven-dried resealable Schlenk tube equipped with a magnetic stir bar was charged with urea substrate **1** (0.1 mmol, 1.0 equiv), CuBr (1.43 mg, 0.01 mmol, 10 mol%), Ag_2CO_3 (16.56 mg, 0.06 mmol, 0.6 equiv), chiral phosphoric acid (*S*)-**A1** (6.2 mg, 0.01 mmol, 10 mol%), HCF_2SO_2Cl (**2c**) (18.0 mg, 0.12 mmol, 1.2 equiv) and ethyl isobutyrate (1.0 mL) at 28 °C, and the sealed tube was then stirred at 28 °C. Upon completion (monitored by TLC), the reaction mixture was directly purified by a silica gel chromatography [eluent: petroleum ether/EtOAc = 100/0-5/1, using petroleum ether (100%) to remove the solvent (ethyl isobutyrate) at first] to afford the desired product **5**.

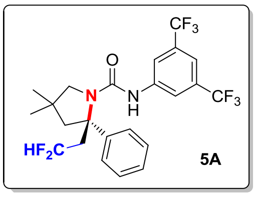

(*R*)-*N*-(3,5-bis(trifluoromethyl)phenyl)-2-(2,2-difluoroethyl)-4,4-dimethyl-2-phenylpyrrolidine-1-carboxamide (5A)

HPLC analysis: Chiralcel OD3 (hexane/*i*-PrOH = 97/3, flow rate 0.2 mL/min, λ = 254 nm), t_R (major) = 30.55 min, t_R (minor) = 33.88 min.

^{1}H NMR (500 MHz, $CDCl_3$) δ 7.88 (s, 2H), 7.52 (s, 1H), 7.37 (t, *J* = 7.5 Hz, 2H), 7.33-7.23 (m, 3H), 6.74 (s, 1H), 6.11-5.84 (m, 1H), 3.61-3.53 (m, 2H), 3.22-3.04 (m, 1H), 2.84-2.72 (m, 1H), 2.64 (d, *J* = 13.5 Hz, 1H), 2.25 (d, *J* = 13.5 Hz, 1H), 1.19 (s, 3H), 0.92 (s, 3H).

^{13}C NMR (126 MHz, $CDCl_3$) δ 153.0, 145.6, 140.2, 132.1 (q, *J* = 33.3 Hz), 128.5, 127.0, 125.3, 123.2 (d, *J* = 272.8 Hz), 119.4 (t, *J* = 4.0 Hz), 116.4 (t, *J* = 241.2 Hz), 116.4-116.3 (m), 68.4, 61.8, 54.8, 43.1 (t, *J* = 19.9 Hz), 36.3, 28.8, 28.4.

^{19}F NMR (376 MHz, $CDCl_3$) δ -63.0 (s, 6F), -112.2 (ddt, *J* = 290.7, 55.8, 12.8 Hz, 1F), -114.3 (d, *J* = 290.1 Hz, 1F).

HRMS (ESI) m/z calcd. for $C_{23}H_{23}F_8N_2O$ $[M+H]^+$ 495.1683, found 495.1677.

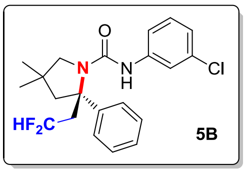

(*R*)-*N*-(3-chlorophenyl)-2-(2,2-difluoroethyl)-4,4-dimethyl-2-phenylpyrrolidine-1-carboxamide (5B)

HPLC analysis: Chiralcel AD3 (hexane/*i*-PrOH = 95/05, flow rate 0.4 mL/min, λ = 230 nm), t_R (major) = 36.46 min, t_R (minor) = 32.54 min.

^{1}H NMR (500 MHz, Acetone-d_6) δ 7.90 (s, 1H), 7.83 (t, *J* = 2.0 Hz, 1H), 7.49 (dd, *J* = 8.0, 2.0 Hz, 1H), 7.44-7.38 (m, 2H), 7.32 (t, *J* = 8.0 Hz, 2H), 7.24 (t, *J* = 8.0 Hz, 1H), 7.20 (t, *J* = 7.5 Hz, 1H), 6.99 (dd, *J* = 8.0, 2.0 Hz, 1H), 6.09 (tdd, *J* = 56.5, 6.0, 3.0 Hz, 1H), 3.75 (d, *J* = 9.5 Hz, 1H), 3.65 (d, *J* = 9.5 Hz, 1H), 3.23-3.11 (m, 1H),

2.75-2.68 (m, 1H), 2.60 (d, J = 13.0 Hz, 1H), 2.23 (d, J = 13.5 Hz, 1H), 1.16 (s, 3H), 0.84 (s, 3H).
^{13}C NMR (126 MHz, Acetone-d_6) δ 153.8, 146.9, 142.0, 133.5, 129.7, 127.8, 126.2, 125.9, 121.8, 119.4, 117.9, 117.4 (t, J = 237.4 Hz), 68.3 (t, J = 5.8 Hz), 61.2, 54.1, 43.2 (t, J = 20.1 Hz), 35.8, 28.2, 27.6.
^{19}F NMR (376 MHz, Acetone-d_6) δ -112.9 (ddd, J = 56.5, 19.7, 13.8 Hz, 2F).
HRMS (ESI) m/z calcd. for $C_{21}H_{24}ClF_2N_2O$ $[M+H]^+$ 393.1545, found 393.1540.

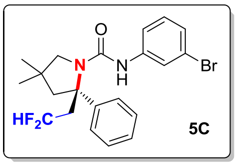

(*R*)-*N*-(3-bromophenyl)-2-(2,2-difluoroethyl)-4,4-dimethyl-2-phenylpyrrolidine-1-carboxamide (5C)
HPLC analysis: Chiralcel OD3 (hexane/*i*-PrOH = 95/5, flow rate 0.5 mL/min, λ = 254 nm), t_R (major) = 23.95 min, t_R (minor) = 32.38 min.
^{1}H NMR (500 MHz, $CDCl_3$) δ 7.68 (s, 1H), 7.37 (t, J = 7.5 Hz, 2H), 7.33-7.25 (m, 4H), 7.22-7.11 (m, 2H), 6.38 (s, 1H), 5.96 (tdd, J = 57.0, 6.5, 3.0 Hz, 1H), 3.63-3.48 (m, 2H), 3.13-3.07 (m, 1H), 2.74 (qd, J = 14.0, 6.5 Hz, 1H), 2.64 (d, J = 13.5 Hz, 1H), 2.23 (d, J = 13.0 Hz, 1H), 1.19 (s, 3H), 0.91 (s, 3H).
^{13}C NMR (126 MHz, $CDCl_3$) δ 153.3, 146.0, 140.0, 130.2, 128.5, 126.9, 126.2, 125.4, 122.6, 122.6, 118.2, 116.6 (t, J = 239.3 Hz), 68.1, 61.8, 54.9, 43.4 (t, J = 19.5 Hz), 36.2, 28.9, 28.5.
^{19}F NMR (376 MHz, $CDCl_3$) δ -111.93 (dddd, J = 290.1, 55.0, 13.9, 11.3 Hz, 1F), -114.3 (d, J = 291.9 Hz, 1F).
HRMS (ESI) m/z calcd. for $C_{21}H_{24}BrF_2N_2O$ $[M+H]^+$ 437.1040, found 437.1046.

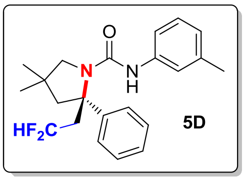

(*R*)-2-(2,2-difluoroethyl)-4,4-dimethyl-2-phenyl-*N*-(m-tolyl)pyrrolidine-1-carboxamide (5D)
HPLC analysis: Chiralcel OD3 (hexane/*i*-PrOH = 95/5, flow rate 0.5 mL/min, λ = 230 nm), t_R (major) = 28.73 min, t_R (minor) = 32.12 min.
^{1}H NMR (500 MHz, $CDCl_3$) δ 7.41-7.30 (m, 5H), 7.26 (t, J = 7.0 Hz, 1H), 7.18 (t, J = 7.5 Hz, 1H), 7.14 (d, J = 7.5 Hz, 1H), 6.89 (d, J = 7.5 Hz, 1H), 6.36 (s, 1H), 6.14-5.84 (m, 1H), 3.60-3.49 (m, 2H), 3.19-3.04 (m, 1H), 2.73 (qd, J = 14.0, 6.5 Hz, 1H), 2.65 (d, J = 13.0 Hz, 1H), 2.35 (s, 3H), 2.22 (d, J = 13.0 Hz, 1H), 1.19 (s, 3H), 0.90 (s, 3H).
^{13}C NMR (126 MHz, $CDCl_3$) δ 153.8, 146.3, 138.9, 138.6, 128.8, 128.4, 126.7, 125.5, 124.1, 120.6, 116.9, 116.8 (t, J = 239.3 Hz), 67.9 (d, J = 9.1 Hz), 61.8, 55.0, 43.6 (t, J = 19.9 Hz), 36.1, 28.9, 28.5, 21.5.
^{19}F NMR (376 MHz, $CDCl_3$) δ -111.9 (ddt, J = 289.3, 54.0, 11.7 Hz, 1F), -114.3 (d, J = 285.4 Hz, 1F).
HRMS (ESI) m/z calcd. for $C_{22}H_{27}F_2N_2O$ $[M+H]^+$ 373.2091, found 273.2086.

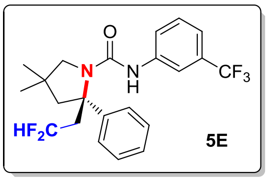

(*R*)-2-(2,2-difluoroethyl)-4,4-dimethyl-2-phenyl-*N*-(3-(trifluoromethyl)phenyl)pyrrolidine-1-carboxamide (5E)

HPLC analysis: Chiralcel AD3 (hexane/*i*-PrOH = 95/5, flow rate 0.4 mL/min, λ = 254 nm), t_R (major) = 23.55 min, t_R (minor) = 25.59 min.

^{1}H NMR (500 MHz, $CDCl_3$) δ 7.66 (s, 1H), 7.61 (d, *J* = 7.0 Hz, 1H), 7.44-7.34 (m, 3H), 7.34-7.24 (m, 4H), 6.54 (s, 1H), 5.98 (td, *J* = 56.5, 3.0 Hz, 1H), 3.65-3.49 (m, 2H), 3.18-3.01 (m, 1H), 2.75 (qd, *J* = 14.0, 6.5 Hz, 1H), 2.65 (d, *J* = 13.0 Hz, 1H), 2.24 (d, *J* = 13.0 Hz, 1H), 1.19 (s, 3H), 0.91 (s, 3H).

^{13}C NMR (126 MHz, $CDCl_3$) δ 153.4, 145.9, 139.3, 131.3 (q, *J* = 32.3 Hz), 129.4, 128.5, 126.9, 125.4, 124.0 (q, *J* = 272.4 Hz), 123.0, 119.8 (q, *J* = 4.0 Hz), 116.6 (t, *J* = 239.4 Hz), 116.4 (q, *J* = 3.9 Hz), 68.1, 61.8, 54.9, 43.4 (t, *J* = 19.8 Hz), 36.2, 28.9, 28.4.

^{19}F NMR (376 MHz, $CDCl_3$) δ -62.6 (s, 3F), -112.0 (ddd, *J* = 290.1, 55.1, 14.3 Hz, 1F), -114.2 (d, *J* = 294.3 Hz, 1F).

HRMS (ESI) m/z calcd. for $C_{22}H_{24}F_5N_2O$ $[M+H]^+$ 427.1809, found 427.1803.

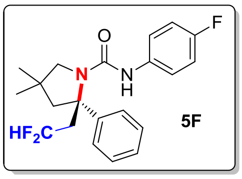

(*R*)-2-(2,2-difluoroethyl)-*N*-(4-fluorophenyl)-4,4-dimethyl-2-phenylpyrrolidine-1-carboxamide (5F)

HPLC analysis: Chiralcel OD3 (hexane/*i*-PrOH = 95/5, flow rate 0.5 mL/min, λ = 254 nm), t_R (major) = 21.79 min, t_R (minor) = 25.51 min.

^{1}H NMR (500 MHz, $CDCl_3$) δ 7.41-7.29 (m, 6H), 7.25 (t, *J* = 7.0 Hz, 1H), 7.00 (t, *J* = 8.7 Hz, 2H), 6.34 (s, 1H), 6.10-5.82 (m, 1H), 3.56-3.52 (m, 2H), 3.17-3.02 (m, 1H), 2.75 (qd, *J* = 14.0, 6.5 Hz, 1H), 2.64 (d, *J* = 13.5 Hz, 1H), 2.23 (d, *J* = 13.0 Hz, 1H), 1.18 (s, 3H), 0.90 (s, 3H).

^{13}C NMR (126 MHz, $CDCl_3$) δ 159.0 (d, *J* = 242.4 Hz), 153.9, 146.1, 134.6 (d, *J* = 2.7 Hz), 128.4, 126.8, 125.5, 122.0 (d, *J* = 8.0 Hz), 116.7 (t, *J* = 239.3 Hz), 115.5 (d, *J* = 22.4 Hz), 68.0, 61.7, 54.9, 43.5 (t, *J* = 19.6 Hz), 36.2, 28.9, 28.5.

^{19}F NMR (376 MHz, $CDCl_3$) δ -111.9 (dddd, *J* = 289.7, 54.9, 13.9, 10.9 Hz, 1F), -114.08 (d, *J* = 291.4 Hz, 1F), -119.6~ -119.7 (m, 1F).

HRMS (ESI) m/z calcd. for $C_{21}H_{24}F_3N_2O$ $[M+H]^+$ 377.1841, found 377.1835.

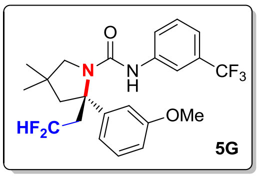

(*R*)-2-(2,2-difluoroethyl)-2-(3-methoxyphenyl)-4,4-dimethyl-*N*-(3-(trifluoromethyl)phenyl)pyrrolidine-1-carboxamide (5G)

HPLC analysis: Chiralcel AD3 (hexane/*i*-PrOH = 95/5, flow rate 0.3 mL/min, λ = 230 nm), t_R (major) = 33.85 min, t_R (minor) = 39.98 min.

^{1}H NMR (500 MHz, $CDCl_3$) δ 7.65 (s, 1H), 7.60 (d, *J* = 8.5 Hz, 1H), 7.40 (t, *J* = 8.0 Hz, 1H), 7.34-7.23 (m, 2H), 6.91 (d, *J* = 8.0 Hz, 1H), 6.87 (s, 1H), 6.81 (d, *J* = 8.0 Hz, 1H), 6.50 (s, 1H), 6.12-5.83 (m, 1H), 3.82 (s, 3H), 3.59-3.54 (m, 2H), 3.19-3.02 (m, 1H), 2.73 (qd, *J* = 14.0, 6.5 Hz, 1H), 2.63 (d, *J* = 13.0 Hz, 1H), 2.24 (d, *J* = 13.0 Hz, 1H), 1.19 (s, 3H), 0.95 (s, 3H).

^{13}**C NMR** (126 MHz, $CDCl_3$) δ 159.6, 153.4, 147.7, 139.2, 131.3 (q, *J* = 32.3 Hz), 129.5, 129.4, 123.9 (q, *J* = 272.4 Hz), 122.9, 119.8 (q, *J* = 4.0 Hz), 117.8, 116.6 (t, *J* = 239.5 Hz), 116.4 (q, *J* = 3.9 Hz), 112.5, 111.3, 68.0, 61.8, 55.3, 54.9, 43.3 (t, *J* = 19.8 Hz), 36.2, 28.9, 28.6.

^{19}**F NMR** (376 MHz, $CDCl_3$) δ -62.6 (s, 3F), -112.0 (ddt, *J* = 289.9, 55.0, 12.9 Hz, 1F), -114.18 (d, *J* = 276.7 Hz, 1F).

HRMS (ESI) m/z calcd. for $C_{23}H_{26}F_5N_2O_2$ $[M+H]^+$ 457.1914, found 457.1909.

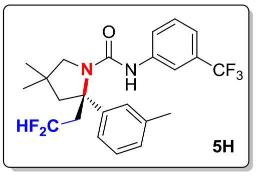

(*R*)-2-(2,2-difluoroethyl)-4,4-dimethyl-2-(m-tolyl)-*N*-(3-(trifluoromethyl)phenyl)pyrrolidine-1-carboxamide (5H)

HPLC analysis: Chiralcel OD3 (hexane/*i*-PrOH = 97/3, flow rate 0.3 mL/min, λ = 230 nm), t_R (major) = 27.14 min, t_R (minor) = 32.71 min.

^{1}**H NMR** (500 MHz, $CDCl_3$) δ 7.70-7.53 (m, 2H), 7.40 (t, *J* = 8.0 Hz, 1H), 7.33-7.22 (m, 2H), 7.16-7.05 (m, 3H), 6.49 (s, 1H), 5.97 (tdd, *J* = 56.5, 6.0, 3.0 Hz, 1H), 3.65-3.52 (m, 2H), 3.17-3.00 (m, 1H), 2.75 (qd, *J* = 14.0, 6.5 Hz, 1H), 2.63 (d, *J* = 13.0 Hz, 1H), 2.38 (s, 3H), 2.23 (d, *J* = 13.0 Hz, 1H), 1.19 (s, 3H), 0.94 (s, 3H).

^{13}**C NMR** (126 MHz, $CDCl_3$) δ 153.4, 145.9, 139.3, 138.2, 131.3 (q, *J* = 32.3 Hz), 129.4, 128.4, 127.8, 126.1, 123.9 (q, *J* = 272.5 Hz), 122.9, 122.5, 119.8 (q, *J* = 3.8 Hz), 116.6 (t, *J* = 239.6 Hz), 116.4 (q, *J* = 3.9 Hz), 67.9, 61.9, 55.1, 43.4 (t, *J* = 19.8 Hz), 36.2, 28.9, 28.6, 21.8.

^{19}**F NMR** (376 MHz, $CDCl_3$) δ -63.1 (s, 3F), -112.9 (ddd, *J* = 56.2, 19.6, 13.9 Hz, 2F).

HRMS (ESI) m/z calcd. for $C_{23}H_{26}F_5N_2O$ $[M+H]^+$ 441.1965, found 441.1960.

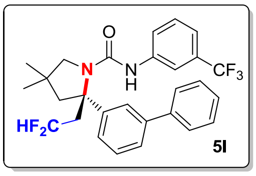

(*R*)-2-([1,1'-biphenyl]-3-yl)-2-(2,2-difluoroethyl)-4,4-dimethyl-*N*-(3-(trifluoromethyl)phenyl)pyrrolidine-1-carboxamide (5I)

HPLC analysis: Chiralcel AD3 (hexane/*i*-PrOH = 95/5, flow rate 0.4 mL/min, λ = 242 nm), t_R (major) = 24.43 min, t_R (minor) = 29.19 min.

^{1}**H NMR** (500 MHz, $CDCl_3$) δ 7.72 (s, 1H), 7.62-7.59 (m, 3H), 7.54-7.35 (m, 7H), 7.32 (t, *J* = 6.5 Hz, 2H), 6.57 (s, 1H), 6.20-5.80 (m, 1H), 3.66-3.50 (m, 2H), 3.27-3.10 (m, 1H), 2.81 (qd, *J* = 14.0, 6.0 Hz, 1H), 2.69 (d, *J* = 13.0 Hz, 1H), 2.31 (d, *J* = 13.0 Hz, 1H), 1.21 (s, 3H), 0.95 (s, 3H).

^{13}**C NMR** (126 MHz, $CDCl_3$) δ 153.4, 146.5, 141.5, 141.2, 139.3, 131.3 (q, *J* = 32.3 Hz), 129.5, 128.8, 127.5, 127.3, 125.9, 124.5, 124.4, 124.0 (q, *J* = 272.5 Hz), 123.0, 119.9 (q, *J* = 3.8 Hz), 116.6 (t, *J* = 239.4 Hz), 116.5 (q, *J* = 3.9 Hz), 68.3, 61.8, 54.9, 43.4 (t, *J* = 19.8 Hz), 36.3, 28.8, 28.5.

^{19}**F NMR** (376 MHz, $CDCl_3$) δ -62.6 (s, 3F), -112.0 (dddd, *J* = 290.2, 55.0, 14.6, 11.2 Hz, 1F), -114.34 (d, *J* = 300.3 Hz, 1F).

HRMS (ESI) m/z calcd. for $C_{28}H_{28}F_5N_2O$ $[M+H]^+$ 503.2122, found 503.2132.

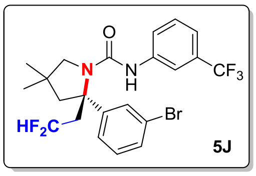

(*R*)-2-(3-bromophenyl)-2-(2,2-difluoroethyl)-4,4-dimethyl-*N*-(3-(trifluoromethyl)phenyl)pyrrolidine-1-carboxamide (5J)

HPLC analysis: Chiralcel AD3 (hexane/*i*-PrOH = 95/5, flow rate 0.4 mL/min, λ = 242 nm), t_R (major) = 22.17 min, t_R (minor) = 20.66 min.

^{1}H NMR (500 MHz, $CDCl_3$) δ 7.68 (s, 1H), 7.65 (d, *J* = 8.5 Hz, 1H), 7.44-7.36 (m, 3H), 7.33 (d, *J* = 8.0 Hz, 1H), 7.27-7.19 (m, 2H), 6.57 (s, 1H), 6.08-5.81 (m, 1H), 3.54 (s, 2H), 3.21-3.08 (m, 1H), 2.75-2.56 (m, 2H), 2.20 (d, *J* = 13.0 Hz, 1H), 1.19 (s, 3H), 0.92 (s, 3H).

^{13}C NMR (126 MHz, $CDCl_3$) δ 153.3, 148.6, 139.1, 131.3 (q, *J* = 32.4 Hz), 129.9, 129.8, 129.5, 128.8, 124.2, 123.9 (q, *J* = 272.4 Hz), 123.1, 122.6, 120.0 (q, *J* = 4.0 Hz), 116.6 (q, *J* = 3.9 Hz), 116.4 (t, *J* = 239.7 Hz), 68.0 (d, *J* = 8.8 Hz), 61.6, 54.6 (d, *J* = 3.7 Hz), 43.0 (dd, *J* = 21.3, 18.4 Hz), 36.5, 28.7, 28.4.

^{19}F NMR (376 MHz, $CDCl_3$) δ -62.6 (s, 3F), -112.2 (dddd, *J* = 290.8, 54.8, 14.3, 10.8 Hz, 1F), -114.0~ -115.4 (m, 1F).

HRMS (ESI) m/z calcd. for $C_{22}H_{23}BrF_5N_2O$ $[M+H]^+$ 505.0914, found 505.0908.

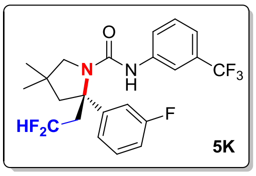

(*R*)-2-(2,2-difluoroethyl)-2-(3-fluorophenyl)-4,4-dimethyl-*N*-(3-(trifluoromethyl)phenyl)pyrrolidine-1-carboxamide (5K)

HPLC analysis: Chiralcel AD3 (hexane/*i*-PrOH = 95/5, flow rate 0.3 mL/min, λ = 254 nm), t_R (major) = 28.42 min, t_R (minor) = 30.25 min.

^{1}H NMR (500 MHz, $CDCl_3$) δ 7.68 (s, 1H), 7.64 (d, *J* = 8.0 Hz, 1H), 7.41 (t, *J* = 8.0 Hz, 1H), 7.35-7.27 (m, 2H), 7.08 (d, *J* = 8.0 Hz, 1H), 7.01 (d, *J* = 10.5 Hz, 1H), 6.95 (td, *J* = 8.0, 2.0 Hz, 1H), 6.59 (s, 1H), 6.10-5.80 (m, 1H), 3.54 (s, 2H), 3.26-3.07 (m, 1H), 2.75-2.60 (m, 2H), 2.21 (d, *J* = 13.5 Hz, 1H), 1.19 (s, 3H), 0.91 (s, 3H).

^{13}C NMR (126 MHz, $CDCl_3$) δ 162.8 (d, *J* = 245.5 Hz), 153.3, 149.0 (d, *J* = 6.4 Hz), 139.1, 131.3 (q, *J* = 32.3 Hz), 129.9 (d, *J* = 8.3 Hz), 129.5, 123.9 (q, *J* = 272.4 Hz), 123.1, 121.1 (d, *J* = 2.8 Hz), 120.0 (q, *J* = 3.9 Hz), 116.5 (q, *J* = 3.9 Hz), 116.5 (t, *J* = 239.6 Hz), 113.7 (d, *J* = 21.0 Hz), 112.9 (d, *J* = 23.1 Hz), 68.1 (d, *J* = 8.7 Hz), 61.6, 54.6 (d, *J* = 3.5 Hz), 43.1 (dd, *J* = 21.2, 18.5 Hz), 36.4, 28.7, 28.4.

^{19}F NMR (376 MHz, $CDCl_3$) δ -62.6 (s, 3F), -112.1 (dddd, *J* = 290.6, 54.9, 14.5, 11.0 Hz, 2F), -113.8~ -115.3 (m, 1F).

HRMS (ESI) m/z calcd. for $C_{22}H_{23}F_6N_2O$ $[M+H]^+$ 445.1715, found 445.1709.

General procedure D: direct asymmetric intramolecular radical aminotrifluoromethylation of alkenes

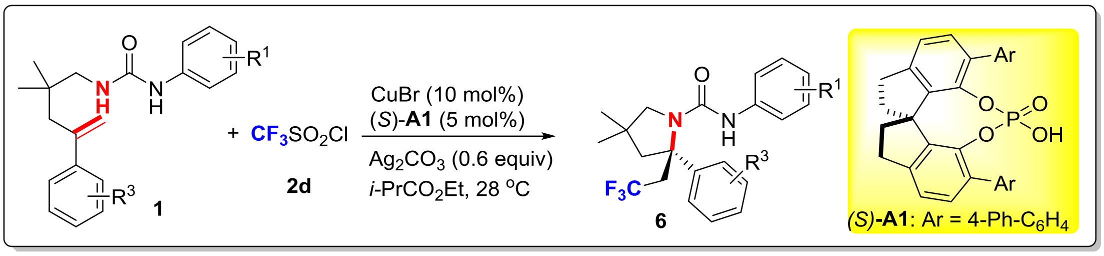

Under argon, an oven-dried resealable Schlenk tube equipped with a magnetic stir bar was charged with urea substrate **1** (0.1 mmol, 1.0 equiv), CuBr (1.43 mg, 0.01 mmol, 10 mol%), Ag_2CO_3 (16.56 mg, 0.06 mmol, 0.6 equiv), chiral phosphoric acid (*S*)-**A1** (3.1 mg, 0.005 mmol, 5 mol%), CF_3SO_2Cl (**2d**) (20.16 mg, 0.12 mmol, 1.2 equiv) and ethyl isobutyrate (1.0 mL) at 0 °C or 28 °C, and the sealed tube was then stirred at 0 °C or 28 °C. Upon completion (monitored by TLC), the reaction mixture was directly purified by a silica gel chromatography [eluent: petroleum ether/EtOAc = 100/0-5/1, using petroleum ether (100%) to remove the solvent (ethyl isobutyrate) at first] to afford the desired product **6**.

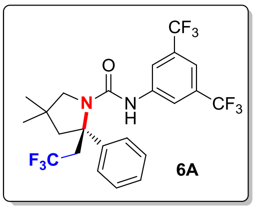

(*R*)-*N*-(3,5-bis(trifluoromethyl)phenyl)-4,4-dimethyl-2-phenyl-2-(2,2,2-trifluoroethyl)pyrrolidine-1-carboxamide (6A)

HPLC analysis: Chiralcel OD3 (hexane/*i*-PrOH = 97/3, flow rate 0.2 mL/min, λ = 254 nm), t_R (major) = 27.39 min, t_R (minor) = 30.70 min.

Spectra matches previously reported spectra.[1]

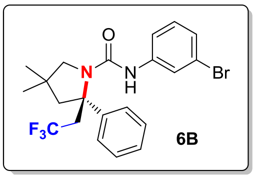

(*R*)-*N*-(3-bromophenyl)-4,4-dimethyl-2-phenyl-2-(2,2,2-trifluoroethyl)pyrrolidine-1-carboxamide (6B)

HPLC analysis: Chiralcel OD3 (hexane/*i*-PrOH = 95/5, flow rate 0.8 mL/min, λ = 254 nm), t_R (major) = 11.91 min, t_R (minor) = 18.87 min.

Spectra matches previously reported spectra.[1]

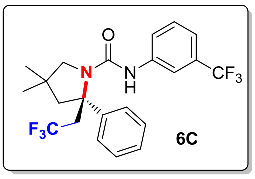

(*R*)-4,4-dimethyl-2-phenyl-2-(2,2,2-trifluoroethyl)-*N*-(3-(trifluoromethyl)phenyl)pyrrolidine-1-carboxamide (6C)

HPLC analysis: Chiralcel AD3 (hexane/*i*-PrOH = 95/5, flow rate 0.6 mL/min, λ = 254 nm), t_R (major) = 14.01 min, t_R (minor) = 17.94 min.

Spectra matches previously reported spectra.[1]

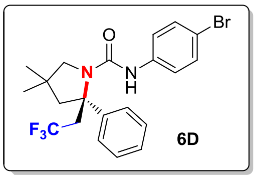

(*R*)-*N*-(4-bromophenyl)-4,4-dimethyl-2-phenyl-2-(2,2,2-trifluoroethyl)pyrrolidine-1-carboxamide (6D)

HPLC analysis: Chiralcel AD3 (hexane/*i*-PrOH = 85/15, flow rate 0.6 mL/min, λ = 254 nm), t_R (major) = 21.33 min, t_R (minor) = 12.01 min.
Spectra matches previously reported spectra.[1]

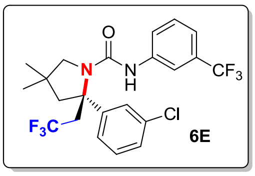

(*R*)-2-(3-chlorophenyl)-4,4-dimethyl-2-(2,2,2-trifluoroethyl)-*N*-(3-(trifluoromethyl)phenyl)pyrrolidine-1-carboxamide (6E)

HPLC analysis: Chiralcel IA (hexane/*i*-PrOH = 95/5, flow rate 0.15 mL/min, λ = 254 nm), t_R (major) = 50.53 min, t_R (minor) = 54.29 min.

^{1}H NMR (500 MHz, $CDCl_3$) δ 7.69-7.60 (m, 2H), 7.41 (t, *J* = 8.0 Hz, 1H), 7.34-7.26 (m, 3H), 7.26-7.21 (m, 2H), 6.54 (s, 1H), 3.86-3.68 (m, 1H), 3.52 (s, 2H), 2.88-2.76 (m, 1H), 2.74 (d, *J* = 13.5 Hz, 1H), 2.17 (d, *J* = 13.5 Hz, 1H), 1.19 (s, 3H), 0.95 (s, 3H).

^{13}C NMR (126 MHz, $CDCl_3$) δ 153.4, 147.9, 139.2, 134.2, 131.2 (q, *J* = 32.3 Hz), 129.5, 129.4, 127.1, 126.1, 124.9 (q, *J* = 278.8 Hz), 124.1, 124.0 (q, *J* = 272.4 Hz), 123.3, 119.9 (q, *J* = 3.9 Hz), 116.7 (q, *J* = 3.9 Hz), 67.8 (d, *J* = 2.4 Hz), 61.2, 52.6, 40.1 (q, *J* = 25.8 Hz), 36.6, 28.4, 28.2.

^{19}F NMR (376 MHz, $CDCl_3$) δ -59.6 (t, *J* = 10.9 Hz, 3F), -62.6 (s, 3F).

HRMS (ESI) m/z calcd. for $C_{22}H_{22}ClF_6N_2O$ $[M+H]^+$ 479.1325, found 479.1319.

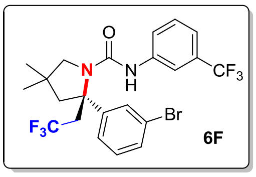

(*R*)-2-(3-bromophenyl)-4,4-dimethyl-2-(2,2,2-trifluoroethyl)-*N*-(3-(trifluoromethyl)phenyl)pyrrolidine-1-carboxamide (6F)

HPLC analysis: Chiralcel IA (hexane/*i*-PrOH = 97/3, flow rate 0.12 mL/min, λ = 230 nm), t_R (major) = 90.40 min, t_R (minor) = 99.74 min.

^{1}H NMR (500 MHz, Acetone-d_6) δ 8.13 (s, 1H), 8.05 (s, 1H), 7.88 (dd, *J* = 8.0, 2.0 Hz, 1H), 7.70 (t, *J* = 2.0 Hz, 1H), 7.54-7.46 (m, 2H), 7.45-7.40 (m, 1H), 7.34-7.27 (m, 2H), 3.86-3.68 (m, 2H), 3.58 (d, *J* = 9.0 Hz, 1H), 3.27-3.11 (m, 1H), 2.73 (d, *J* = 13.5 Hz, 1H), 2.24 (d, *J* = 13.5 Hz, 1H), 1.17 (s, 3H), 0.93 (s, 3H).

^{13}C NMR (126 MHz, Acetone-d_6) δ 153.9, 149.2, 141.2, 130.2 (q, *J* = 31.8 Hz), 129.7, 129.5, 129.4, 129.3, 127.7 (q, *J* = 274.5 Hz), 125.4, 124.8 (q, *J* = 272.0 Hz), 123.1, 121.6, 118.5 (q, *J* = 3.9 Hz), 116.1 (q, *J* = 4.1 Hz), 67.7 (d, *J* = 2.2 Hz), 60.8, 52.5 (d, *J* = 2.2 Hz), 39.4 (q, *J* = 25.4 Hz), 36.1, 27.6, 27.5.

^{19}F NMR (376 MHz, Acetone-d_6) δ -60.0 (t, *J* = 11.2 Hz, 3F), -63.1 (s, 3F).

HRMS (ESI) m/z calcd. for $C_{22}H_{22}BrF_6N_2O$ $[M+H]^+$ 523.0814, found 523.0804.

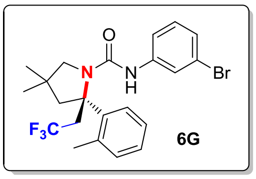

(*R*)-*N*-(3-bromophenyl)-4,4-dimethyl-2-(o-tolyl)-2-(2,2,2-trifluoroethyl)pyrrolidine-1-carboxamide (6G)

HPLC analysis: Chiralcel OD3 (hexane/*i*-PrOH = 95/5, flow rate 0.5 mL/min, λ = 254 nm), t_R (major) = 15.14 min, t_R (minor) = 26.14 min.

Spectra matches previously reported spectra[1].

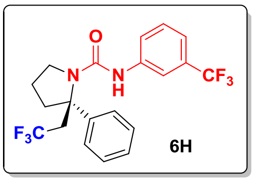

(*R*)-2-phenyl-2-(2,2,2-trifluoroethyl)-*N*-(3-(trifluoromethyl)phenyl)pyrrolidine-1-carboxamide (6H)

HPLC analysis: Chiralcel ODH (hexane/*i*-PrOH = 90/10, flow rate 0.5 mL/min, λ = 254 nm), t_R (major) = 15.04 min, t_R (minor) = 19.04 min.

Spectra matches previously reported spectra[1].

Procedure for synthetic application:

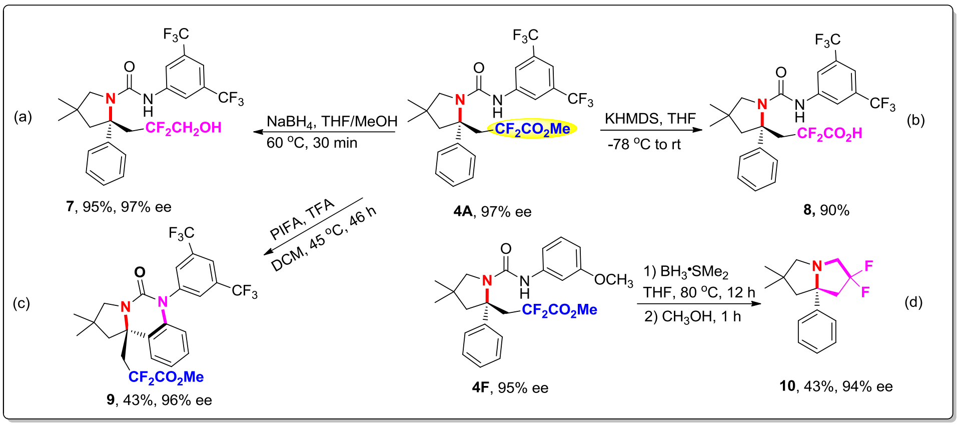

Synthesis of **7**: Sodium borohydride (9.5 mg, 0.25 mmol) was added to a solution of **4A** (27.6 mg, 0.05 mmol) in THF (2 mL) in a sealed flask. The resulting mixture was stirred at 60 °C for 15 min. Methanol (2 mL) was then added by syringe and stirring was maintained for a further period of 15 min at the same temperature. After that, the solvent was removed *in vacuo*, and the residue was purified by a silica gel column chromatography (eluent: petroleum ether/EtOAc = 8/1) to give **7** (24.9 mg, 95%).

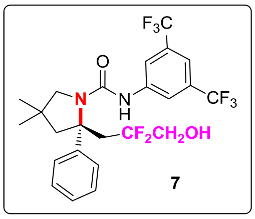

7

(*R*)-*N*-(3,5-bis(trifluoromethyl)phenyl)-2-(2,2-difluoro-3-hydroxypropyl)-4,4-dimethyl-2-phenylpyrrolidine-1-carboxamide (7)

HPLC analysis: Chiralcel ODH (hexane/*i*-PrOH = 97/3, flow rate 0.4 mL/min, λ = 254 nm), t_R (major) = 15.28 min, t_R (minor) = 19.92 min.

^{1}H NMR (500 MHz, $CDCl_3$) δ 7.86 (s, 2H), 7.49 (s, 1H), 7.39-7.28 (m, 4H), 7.26-7.19 (m, 1H), 6.67 (s, 1H), 3.73-3.54 (m, 3H), 3.51 (d, *J* = 7.5 Hz, 1H), 3.46-3.30 (br s, 1H), 2.87 (d, *J* = 13.0 Hz, 1H), 2.76-2.59 (m, 1H), 2.52 (s, 1H), 2.18 (d, *J* = 13.2 Hz, 1H), 1.16 (s, 3H), 0.89 (s, 3H).

^{13}C NMR (126 MHz, $CDCl_3$) δ 153.8, 146.3, 140.5, 132.2 (q, *J* = 33.3 Hz), 128.4, 127.0, 125.8, 123.4 (t, *J* = 246.0 Hz), 123.3 (q, *J* = 273.3 Hz), 119.5 (d, *J* = 3.2 Hz), 116.4 (p, *J* = 3.7 Hz), 69.3, 65.6 (t, *J* = 31.6 Hz), 61.6, 53.6, 39.8 (t, *J* = 20.4 Hz), 36.5, 28.5, 28.5.

^{19}F NMR (376 MHz, $CDCl_3$) δ -63.0 (s, 6F), -98.2 (d, *J* = 242.8 Hz, 1F), -113.4 (d, *J* = 248.2 Hz, 1F).

HRMS (ESI) m/z calcd. for $C_{24}H_{25}F_8N_2O_2$ $[M+H]^+$ 525.1788, found 525.1795.

Synthesis of **8**: KHMDS (7.5 μL, 1.0 M in THF, 0.075 mmol) was added to a stirred solution of **4A** (27.6 mg, 0.05 mmol) in dry THF (0.5 mL) in an oven-dried Schlenk tube at −78 °C. After stirring at −78 °C for 1 h, the reaction mixture was allowed to warm to room temperature over a period of 11 h, quenched with saturated NH_4Cl solution and stirred for 15 min. The mixture was extracted three times with ethyl acetate, dried over Na_2SO_4, and concentrated *in vacuo*. The crude product was purified by flash column chromatography on

silica gel (eluent: EtOAc/MeOH = 20/1) to give **8** (12.1 mg, 90%).

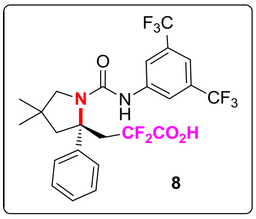

(*R*)-3-(1-((3,5-bis(trifluoromethyl)phenyl)carbamoyl)-4,4-dimethyl-2-phenylpyrrolidin-2-yl)-2,2-difluoropropanoic acid (8)

$[\alpha]_D^{20}$ = –67.50 (*c* = 0.10 in CH_2Cl_2).

^{1}H NMR (500 MHz, CD_3OD) δ 8.14 (s, 2H), 7.49 (s, 1H), 7.37 (d, *J* = 7.5 Hz, 2H), 7.30 (t, *J* = 8.0 Hz, 2H), 7.18 (t, *J* = 7.5 Hz, 1H), 3.66 (d, *J* = 9.0 Hz, 1H), 3.60 (d, *J* = 9.0 Hz, 1H), 3.53-3.38 (m, 1H), 3.00 (dd, *J* = 34.0, 16.0 Hz, 1H), 2.89 (d, *J* = 13.0 Hz, 1H), 2.04 (d, *J* = 13.0 Hz, 1H), 1.14 (s, 3H), 0.86 (s, 3H).

^{13}C NMR (126 MHz, CD_3OD) δ 155.9, 148.5, 143.5, 132.7 (q, *J* = 33.0 Hz), 128.8, 127.3, 127.2, 124.9 (q, *J* = 272.3 Hz), 121.2 (d, *J* = 3.3 Hz), 116.0 (p, *J* = 3.7 Hz), 70.5, 62.4, 54.0, 41.9 (t, *J* = 22.6 Hz), 37.3, 28.7, 28.3.

^{19}F NMR (376 MHz, CD_3OD) δ -64.4 (s, 6F), -100.0 (d, *J* = 252.0 Hz, 1F), -104.3 (d, *J* = 250.8 Hz, 1F).

HRMS (ESI) m/z calcd. for $C_{24}H_{21}F_8N_2O_3$ $[M-H]^-$ 537.1424, found 537.1432.

Synthesis of **9**: To a stirred solution of **4A** (27.6 mg, 0.05 mmol) and $PhI(OTFA)_2$ (PIFA, 75.3 mg, 0.175 mmol) in dry CH_2Cl_2 (0.5 mL) in an oven-dried resealable Schlenk tube 2,2,2-trifluoroacetic acid (TFA, 11.2 μL, 0.15 mmol) was added. The reaction mixture was stirred at 45 °C for 46 h. Then cooled to room temperature, CH_2Cl_2 (2.0 mL) was added and the solution was washed successively with saturated solutions of $NaHCO_3$, brine, dried over Na_2SO_4, filtered and concentrated *in vacuo*. The residue was purified by a silica gel column chromatography (eluent: petroleum ether/EtOAc = 20/1) to give **9** (11.8 mg, 43%).

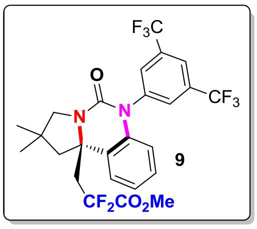

(*R*)-methyl 3-(6-(3,5-bis(trifluoromethyl)phenyl)-2,2-dimethyl-5-oxo-1,2,3,5,6,10b-hexahydropyrrolo[1,2-c]quinazolin-10b-yl)-2,2-difluoropropanoate (9)

HPLC analysis: Chiralcel OD3 (hexane/*i*-PrOH = 99/1, flow rate 0.2 mL/min, λ = 240 nm), t_R (major) = 28.08 min, t_R (minor) = 38.19 min.

^{1}H NMR (500 MHz, $CDCl_3$) δ 7.97 (s, 2H), 7.93 (s, 1H), 7.18-7.10 (m, 2H), 7.06 (td, *J* = 7.5, 1.0 Hz, 1H), 6.20 (d, *J* = 8.0 Hz, 1H), 3.79 (d, *J* = 11.5 Hz, 1H), 3.53 (s, 3H), 3.28 (d, *J* = 11.5 Hz, 1H), 3.01-2.86 (m, 1H), 2.65-2.55 (m, 1H), 2.53 (d, *J* = 13.0 Hz, 1H), 2.43 (d, *J* = 13.0 Hz, 1H), 1.29 (s, 3H), 1.00 (s, 3H).

^{13}C NMR (126 MHz, $CDCl_3$) δ 163.6 (t, *J* = 32.2 Hz), 150.8, 139.8, 138.9, 133.1 (q, *J* = 33.9 Hz), 130.9, 128.9, 126.4, 125.6, 123.3, 123.1 (q, *J* = 275.8 Hz), 122.2-121.9 (m), 115.4, 114.9 (dd, *J* = 254.0, 251.1 Hz), 62.9 (dd, *J* = 5.1, 3.2 Hz), 58.8, 54.4, 53.8, 45.0 (t, *J* = 21.5 Hz), 36.9, 29.4, 27.4.

^{19}F NMR (376 MHz, $CDCl_3$) δ -62.8 (s, 6F), -99.9 (d, *J* = 277.5 Hz, 1F), -103.7 (d, *J* = 277.5 Hz, 1F).

HRMS (ESI) m/z calcd. for $C_{25}H_{23}F_8N_2O_3$ $[M+H]^+$ 551.1581, found 551.1586.

Synthesis of **10:** To an oven-dried resealable Schlenk tube equipped with a magnetic stir bar were added **4F** (22.3 mg, 0.05 mmol), $BH_3{\cdot}SMe_2$ (0.125 mL, 2.0 M in THF, 0.25 mmol) and THF (0.5 mL) under argon. The reaction mixture was refluxed for 12 h, then cooled to room temperature, quenched with CH_3OH (0.5 mL) at 0 °C (carefully addition), and stirred for an additional 60 min. The solvent was removed *in vacuo*, and the residue was purified by a silica gel column chromatography (eluent: petroleum ether/EtOAc = 50/1) to afford **10** (5.4 mg, 43%).

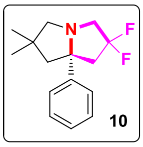

(*R*)-2,2-difluoro-6,6-dimethyl-7a-phenylhexahydro-1H-pyrrolizine (10)

HPLC analysis: Chiralcel OJ3 (hexane/*i*-PrOH = 100/0, flow rate 0.1 mL/min, λ = 214 nm), t_R (major) = 77.27 min, t_R (minor) = 55.48 min.

^{1}H NMR (400 MHz, $CDCl_3$) δ 7.49-7.42 (m, 2H), 7.34-7.27 (m, 2H), 7.22-7.15 (m, 1H), 3.41-3.16 (m, 2H), 3.02 (d, *J* = 8.6 Hz, 1H), 2.91 (d, *J* = 8.5 Hz, 1H), 2.76-2.58 (m, 1H), 2.56-2.41 (m, 1H), 2.18 (d, *J* = 12.7 Hz, 1H), 2.10 (d, *J* = 12.7 Hz, 1H), 1.09 (s, 3H), 0.89 (s, 3H).

^{13}C NMR (126 MHz, $CDCl_3$) δ 149.6, 133.0 (dd, *J* = 256.2, 252.5 Hz), 128.4, 126.2, 125.4, 75.1, 68.7, 60.1 (dd, *J* = 27.1, 24.8 Hz), 56.5, 51.3 (t, *J* = 22.4 Hz), 39.8, 28.1, 27.9.

^{19}F NMR (376 MHz, $CDCl_3$) δ -90.8 (d, *J* = 230.3 Hz, 1F), -97.1 (d, *J* = 230.3 Hz, 1F).

HRMS (ESI) m/z calcd. for $C_{15}H_{20}F_2N$ $[M+H]^+$ 252.1564, found 330.1563.

Mechanistic study

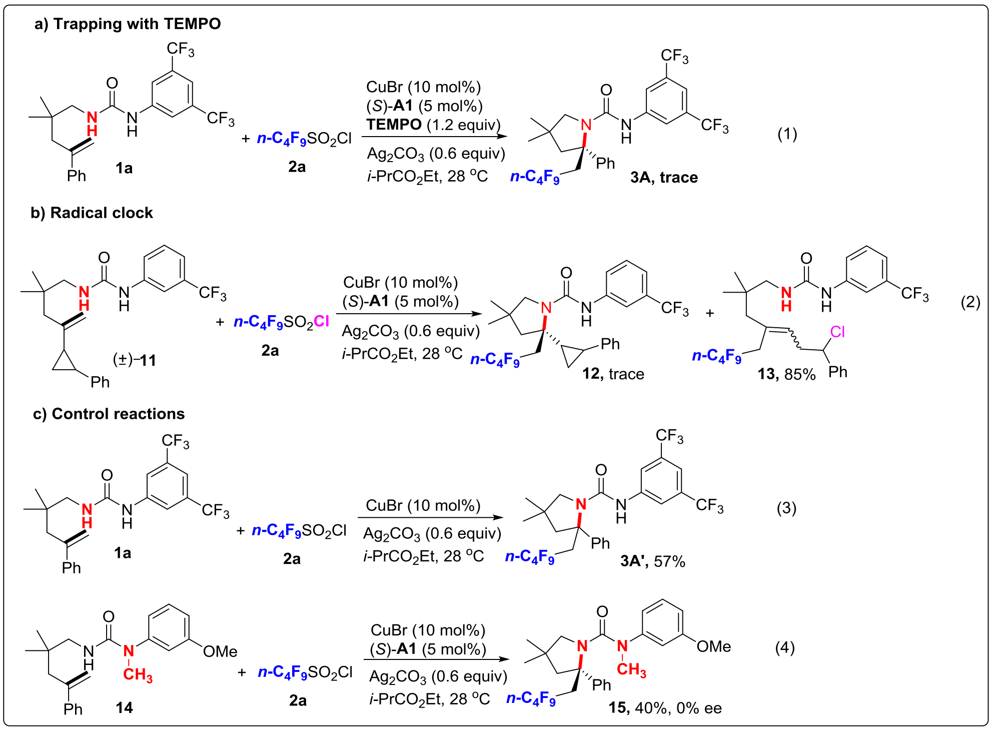

a) Trapping with TEMPO

Under argon, an oven-dried resealable Schlenk tube equipped with a magnetic stir bar was charged with urea substrate **1a** (0.05 mmol, 1.0 equiv), CuBr (0.72 mg, 0.005 mmol, 10 mol%), chiral phosphoric acid (*S*)-**A1** (1.6 mg, 0.0025 mmol, 5 mol%), *n*-$C_4F_9SO_2Cl$ **2a** (19.07 mg, 0.06 mmol, 1.2 equiv), 2,2,6,6-tetramethyl-1-piperidinyloxy (TEMPO, 9.4 mg, 0.06 mmol, 1.2 equiv) and ethyl isobutyrate (0.5 mL) at 28 °C, and the sealed tube was then stirred at 28 °C for 36 h. $PhOCF_3$ (internal standard, 0.05 mmol, 1.0 equiv) was added to the reaction mixture. Yield was based on ^{19}F NMR analysis of the crude product.

Note: Since the reaction is sensitive to water and air, Schlenk tube and the reagents must be dried prior to use.

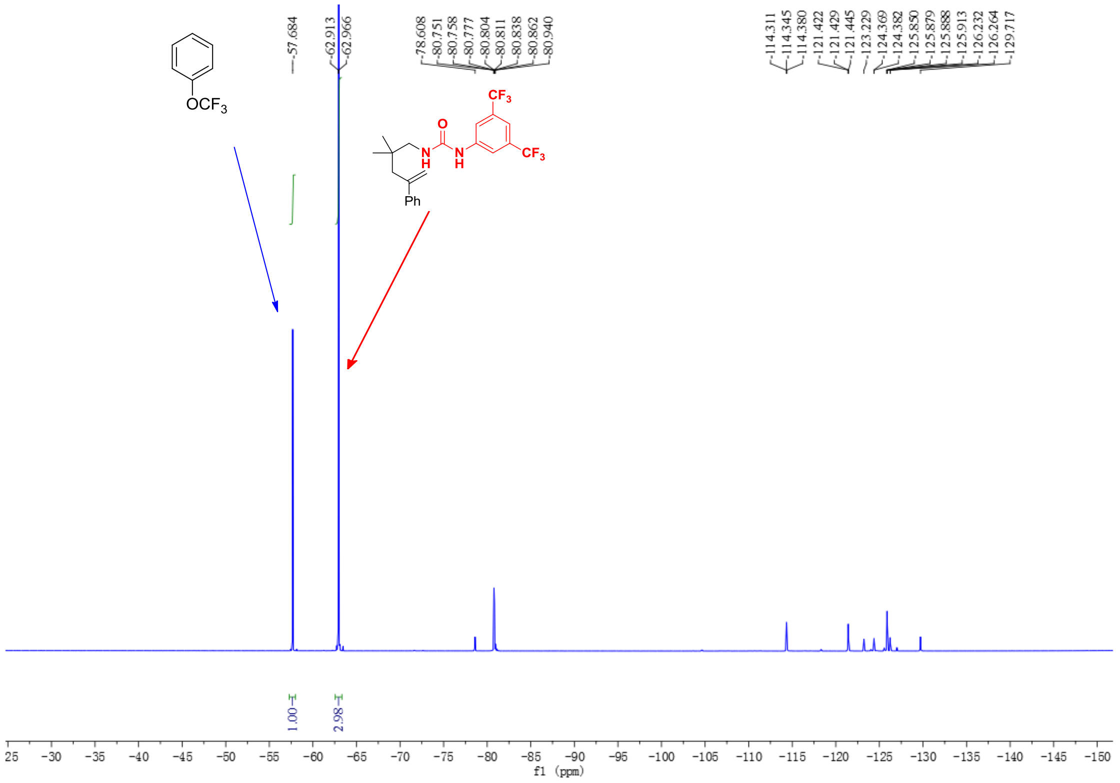

b) Radical clock

Procedure for synthesis of substrate **11**

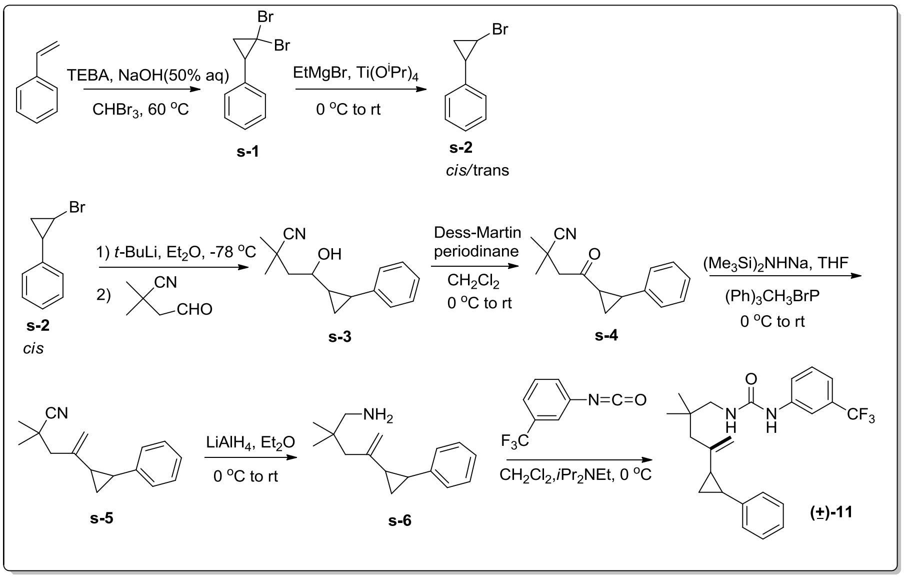

According to the procedures previously reported[2], styrene (5.2 g, 50.0 mmol), $CHBr_3$ (50.5 g, 200.0 mmol) and triethylbenzylammonium chloride (TEBA, 1.1 g, 5.0 mmol) were added to a 250 mL flask under nitrogen atmosphere. A solution of sodium hydroxide (6.0 g in 6.0 mL of H_2O) was added dropwise to the mixture at 0 °C. After addition, the reaction mixture was stirred for 2 hours at 60 °C. The resulting mixture was diluted by 50 mL of water and extracted with dichloromethane (3 × 50 mL). The combined organic layers were washed with brine, and dried with anhydrous Na_2SO_4.

The solvent was removed *in vacuo*, and distillation (120 ^oC (12 mmHg)) of the residue afforded (2,2-dibromocyclopropyl)benzene (**s-1**) (11.5 g, 83%) as a colorless liquid.

According to the procedures previously reported,[3] to a solution of **s-1** (2.75 g, 10.0 mmol) and titanium isopropoxide (0.14 g, 0.5 mmol) in dry THF (50 mL), ethylmagnesium bromide (5.4 mL, 2.4 M in 2-Methyltetrahydrofuran, 13.0 mmol) was slowly added by syringe at 0 ^oC. The reaction mixture was stirred at 0 °C for 20 min and then at room temperature for 20 min. Then the reaction was quenched by saturated NH_4Cl solution (10 mL), and extracted with EtOAc (3 × 30 mL). The combined organic layers were brined, dried over Na_2SO_4, filtered and concentrated *in vacuo*. The residue was purified by a silica gel chromatography (eluent: petroleum ether) to afford the desired product **s-2** (0.89 g, 45%).

To a solution of **s-2** (1.97 g, 10.0 mmol) in dry Et_2O (20 mL) was added *t*-BuLi (17.0 mL, 1.3 M in *n*-pentane, 22.0 mmol) at -78 ^oC. The reaction mixture was stirred at -78 ^oC for 30 min. 2,2-Dimethyl-4-oxobutanenitrile (1.11 g, 10.0 mmol) in dry Et_2O (5 mL) was slowly added, and the reaction mixture was stirred for 2 h. Saturated NH_4Cl solution was added and the solution was extracted with EtOAc (3 × 20 mL). The organic layer was dried over anhydrous Na_2SO_4, filtered and concentrated *in vacuo* to afford the crude product **s-3**. To a stirred suspension of Dess-Martin periodinane (5.09 g, 12.0 mmol) and $NaHCO_3$ (1.00 g, 12.0 mmol) in CH_2Cl_2 (40 mL) was added a solution of **s-3** in CH_2Cl_2 (10 mL) at 0 ^oC. The reaction mixture was stirred at room temperature for 2 h. The resulting solution was washed with saturated aqueous $NaHCO_3$ (2 × 30 mL) and saturated aqueous NaS_2O_3 (2 × 30 mL), dried over anhydrous Na_2SO_4, and concentrated *in vacuo* to afford crude product, which was purified by a silica gel chromatography (eluent: petroleum ether/EtOAc = 10/1-4/1) to afford **s-4** (0.52 g, 23% over two steps).

Sodium bis(trimethylsilyl)amide (2.6 mL, 2.0 M in THF) was added to a solution of methyltriphenylphosphonium bromide (1.86 g, 5.2 mmol) in dry THF (10 mL) at 0 ^oC. After stirring for 30 min, a solution of **s-4** (0.91 g, 4 mmol) in THF (5 mL) was added and the reaction mixture was gradually warmed up to room temperature and stirred for an additional 8 h. The mixture was filtered through silica-pad (Et_2O was used as an eluent). The eluate was concentrated *in vacuo* then purified by a silica gel chromatography (eluent: petroleum ether/EtOAc = 40/1-20/1) to afford **s-5** (0.78 g, 87%).

To a suspension of $LiAlH_4$ (228 mg, 6.0 mmol) in Et_2O (5 mL) at 0 °C was slowly added a solution of **s-5** (676 mg, 3.0 mmol) in Et_2O (3.0 mL), then the mixture was warmed to room temperature, stirred for an additional 2 h. The reaction mixture was quenched by slow, sequential addition of water (0.1 mL) in Na_2SO_4 (1.0 g) at 0 °C. The reaction mixture was warmed to room temperature, stirred for an additional 30 min, filtered and concentrated *in vacuo* to afford **s-6**, which was used in the next reaction without further purification.

1-Isocyanato-3-(trifluoromethyl)benzene (2.0 mmol) was slowly added to a stirred solution of **s-6** (2.0 mmol) and ethyldiisopropylamine (*i*Pr_2NEt, 2.0 mmol) in dry CH_2Cl_2 (5.0 mL) at 0 ^oC. The reaction mixture was stirred for an additional 30 min at

0 ^{o}C. After complete conversion (monitored by TLC), the crude mixture was directly purified by silica gel column chromatography (eluent: petroleum ether/CH_2Cl_2 = 100:1-1:5, using petroleum ether (100%) to remove CH_2Cl_2 and *i*Pr_2NEt at first) to give urea substrate **11**.

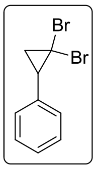

(2,2-dibromocyclopropyl)benzene (s-1)

^{1}H NMR (500 MHz, $CDCl_3$) δ 7.41-7.32 (m, 3H), 7.31-7.26 (m, 2H), 2.98 (dd, *J* = 10.5, 8.5 Hz, 1H), 2.15 (dd, *J* = 10.5, 8.5 Hz, 1H), 2.07-2.00 (m, 1H).

^{13}C NMR (126 MHz, $CDCl_3$) δ 136.1, 129.0, 128.4, 127.7, 36.0, 28.6, 27.3.

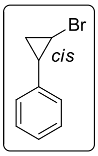

((*cis*)-2-bromocyclopropyl)benzene (s-2)

^{1}H NMR (400 MHz, $CDCl_3$) δ 7.40-7.26 (m, 5H), 3.37-3.29 (m, 1H), 2.38-2.29 (m, 1H), 1.64-1.56 (m, 1H), 1.39-1.32 (m, 1H).

^{13}C NMR (100 MHz, CDCl3) δ 137.3, 129.3, 128.1, 126.9, 24.2, 22.2, 14.3.

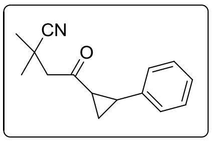

2,2-dimethyl-4-oxo-4-(2-phenylcyclopropyl)butanenitrile (s-4)

^{1}H NMR (500 MHz, $CDCl_3$) δ 7.29-7.13 (m, 5H), 2.77 (q, *J* = 8.5 Hz, 1H), 2.67 (d, *J* = 16.5 Hz, 1H), 2.50 (d, *J* = 16.5 Hz, 1H), 2.48-2.43 (m, 1H), 1.98-1.91 (m, 1H), 1.40-1.33 (m, 1H), 1.15 (s, 3H), 1.12 (s, 3H).

^{13}C NMR (126 MHz, $CDCl_3$) δ 201.3, 135.3, 129.2, 128.1, 127.0, 124.5, 53.0, 30.4, 29.4, 29.2, 26.7, 26.1, 11.7.

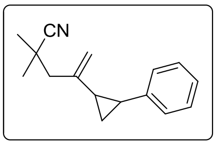

2,2-dimethyl-4-oxo-4-(2-phenylcyclopropyl)butanenitrile (s-5)

^{1}H NMR (400 MHz, $CDCl_3$) δ 7.26-7.07 (m, 5H), 4.96-4.91 (m, 2H), 2.45-2.32 (m, 1H), 2.10 (q, 8.0 Hz, 1H), 1.94 (d, *J* = 14.0 Hz, 1H), 1.88 (d, *J* = 14.0 Hz, 1H), 1.25-1.23 (m, 8H).

^{13}C NMR (100 MHz, $CDCl_3$) δ 140.7, 138.3, 128.4, 127.8, 125.8, 117.2, 48.0, 31.2, 27.8, 27.1, 26.7, 26.7, 23.7, 10.7.

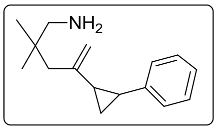

2,2-dimethyl-4-(2-phenylcyclopropyl)pent-4-en-1-amine (s-6)

^{1}H NMR (500 MHz, $CDCl_3$) δ 7.23-7.18 (m, 2H), 7.16-7.06 (m, 3H), 4.77 (t, *J* = 1.5 Hz, 1H), 4.73 (s, 1H), 2.41 (s, 2H), 2.32-2.24 (m, 1H), 1.91 (q, 8.0 Hz, 1H), 1.71 (d, *J* = 13.5 Hz, 1H), 1.59 (d, *J* = 13.5 Hz, 1H), 1.19 (t, *J* = 7.5 Hz, 2H), 0.83 (s, 3H), 0.83 (s, 3H).

^{13}C NMR (126 MHz, $CDCl_3$) δ 142.9, 138.7, 128.3, 127.6, 125.6, 115.3, 53.1, 46.5, 28.0, 25.4, 25.2, 23.5, 10.8.

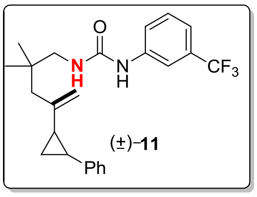

1-(2,2-dimethyl-4-(2-phenylcyclopropyl)pent-4-en-1-yl)-3-(3-(trifluoromethyl)phenyl)urea (11)

^{1}H NMR (500 MHz, $CDCl_3$) δ 7.55 (s, 1H), 7.50 (d, *J* = 8.5 Hz, 1H), 7.34 (t, *J* = 8.0 Hz, 1H), 7.27-7.19 (m, 3H), 7.18-7.12 (m,

2H), 7.11-7.05 (m, 2H), 5.15 (t, J = 6.0 Hz, 1H), 4.78 (s, 1H), 4.74 (s, 1H), 3.05 (d, J = 6.0 Hz, 2H), 2.28 (dd, J = 15.5, 8.5 Hz, 1H), 1.88 (dd, J = 16.0, 8.0 Hz, 1H), 1.73 (d, J = 13.5 Hz, 3H), 1.63 (d, J = 13.5 Hz, 1H), 0.88 (s, 3H), 0.86 (s, 3H).
^{13}C NMR (126 MHz, $CDCl_3$) δ 155.8, 142.4, 139.6, 138.7, 131.3 (q, J = 32.2 Hz) 129.5, 128.6, 127.6, 125.6, 123.9 (q, J = 272.9 Hz), 122.7, 119.5 (q, J = 3.8 Hz), 116.2 (q, J = 3.8 Hz), 115.6, 50.1, 47.1, 28.0, 26.9, 25.7, 25.3, 23.6, 10.9.
^{19}F NMR (376 MHz, $CDCl_3$) δ -62.8 (s, 3F).

b) Radical clock experiment:

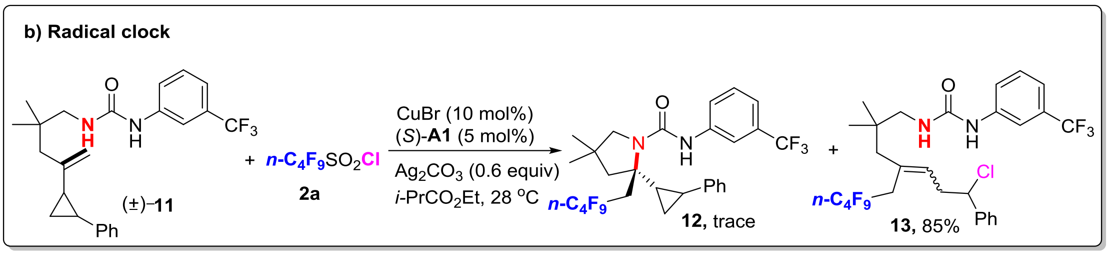

Under argon, an oven-dried resealable Schlenk tube equipped with a magnetic stir bar was charged with urea substrate **11** (0.1 mmol, 1.0 equiv), CuBr (1.43 mg, 0.01 mmol, 10 mol%), chiral phosphoric acid (S)-**A1** (3.1 mg, 0.005 mmol, 5 mol%), n-$C_4F_9SO_2Cl$ (**2a**) (38.15 mg, 0.12 mmol, 1.2 equiv) and ethyl isobutyrate (1.0 mL) at 28 °C, and the sealed tube was then stirred at 28 °C for 72 h. Upon completion (monitored by TLC), the reaction mixture was directly purified by a silica gel chromatography [eluent: petroleum ether/EtOAc = 100/0-5/1, using petroleum ether (100%) to remove the solvent (ethyl isobutyrate) at first] to afford the desired product **13** in 85% yield (ca.9:1 mixture of alkene geometric isomers by **^{1}H NMR**).

Note: Since the reaction is sensitive to water and air, Schlenk tube and the reagents must be dried prior to use.

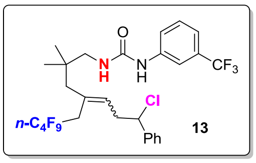

^{1}H NMR (500 MHz, $CDCl_3$) δ 7.58 (s, 1H, major + minor), 7.51 (d, J = 8.0 Hz, 1H, major + minor), 7.43-7.20 (m, 8H, major + minor), 7.03 (s, 1H, minor), 6.94 (d, J = 21.0 Hz, 1H, major), 6.80 (dd, J = 15.5, 11.0 Hz, 1H, minor), 6.57 (d, J = 15.5 Hz, 1H, minor), 6.31 (d, J = 11.1 Hz, 1H, minor), 5.56 (t, J = 7.2 Hz, 1H, major), 5.03 (t, J = 6.0 Hz, 1H, major), 4.89 (dd, J = 8.5, 5.5 Hz, 1H, major), 3.18-2.98 (m, 2H, major + minor), 2.98-2.65 (m, 4H, major + minor), 2.12-1.97 (m, 2H, major + minor), 0.99-0.76 (m, 7H, major + minor).
^{19}F NMR (376 MHz, $CDCl_3$) δ -62.7 (s, 3F, major + minor), -80.9 (t, J = 9.4 Hz, 3F, major + minor), -112.5 (s, 2F, major + minor), -124.1 (s, 2F, major + minor), -125.7 (t, J = 12.5 Hz, 2F, major + minor).
HRMS (ESI) m/z calcd. for $C_{28}H_{28}ON_2ClF_{12}$ $[M+H]^+$: 671.1699, found 671.1704.

c) Control reactions

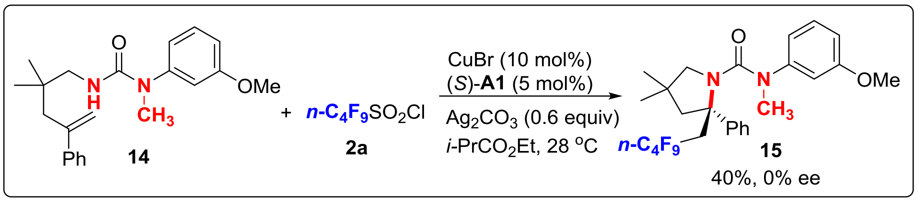

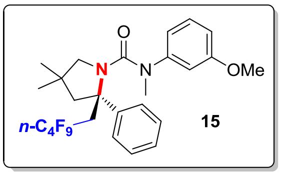

(*R*)-*N*-(3-methoxyphenyl)-*N*,4,4-trimethyl-2-(2,2,3,3,4,4,5,5,5-nonafluoropentyl)-2-phenylpyrrolidine-1-carboxamide (15)

HPLC analysis: Chiralcel AD3 (hexane/*i*-PrOH = 98/2, flow rate 0.2 mL/min, λ = 214 nm), t_R (major) = 25.61 min, t_R (minor) = 28.56 min.

^{1}H NMR (500 MHz, $CDCl_3$) δ 7.38-7.30 (m, 4H), 7.29-7.19 (m, 2H), 6.73 (dd, *J* = 8.7, 2.0 Hz, 1H), 6.69-6.63 (m, 2H), 3.77 (s, 3H), 3.68-3.52 (m, 1H), 3.46-3.30 (m, 1H), 3.15 (s, 3H), 2.94-2.83 (m, 2H), 2.49 (d, *J* = 13.5 Hz, 1H), 2.14 (d, *J* = 13.5 Hz, 1H), 0.92 (s, 3H), 0.71 (s, 3H).

^{13}C NMR (126 MHz, $CDCl_3$) δ 160.5, 159.2, 147.6, 145.6, 130.1, 127.9, 126.7, 126.3, 117.6, 111.3, 110.7, 121.4-105.9 (m), 69.0, 62.3, 55.3, 52.4, 41.0, 37.0 (t, *J* = 18.7 Hz), 36.2, 29.0, 27.9.

^{19}F NMR (376 MHz, $CDCl_3$) δ -80.3 ~ -81.5 (m, 3F), -107.7 ~ -112.0 (m, 2F), -124.3 (q, *J* = 9.7 Hz, 2F), -125.3 ~ -126.7 (m, 2F).

HRMS (ESI) m/z calcd. for $C_{26}H_{28}F_9N_2O_2$ $[M+H]^+$ 571.2007, found 571.2013.

Supplementary References:

1 Lin, J. S. *et al.* A Dual-Catalytic Strategy To Direct Asymmetric Radical Aminotrifluoromethylation of Alkenes. *J. Am. Chem. Soc.* **138,** 9357-9360 (2016).

2 Clavier, H., Le Jeune, K., de Riggi, I., Tenaglia, A. & Buono, G. Highly Selective Cobalt-Mediated 6+2 Cycloaddition of Cycloheptatriene and Allenes. *Org. Lett.* **13,** 308-311 (2011).

3 Al Dulayymi, J. a. R., Baird, M. S., Bolesov, I. G., Nizovtsev, A. V. & Tverezovsky, V. V. Hydrodehalogenation of 1,1-dibromocyclopropanes by Grignard reagents promoted by titanium compounds. *J. Chem. Soc, Perkin Trans. 2***,** 1603-1618 (2000).
